# 26th Annual Computational Neuroscience Meeting (CNS*2017): Part 3

**DOI:** 10.1186/s12868-017-0372-1

**Published:** 2017-08-18

**Authors:** Adam J. H. Newton, Alexandra H. Seidenstein, Robert A. McDougal, Alberto Pérez-Cervera, Gemma Huguet, Tere M-Seara, Caroline Haimerl, David Angulo-Garcia, Alessandro Torcini, Rosa Cossart, Arnaud Malvache, Kaoutar Skiker, Mounir Maouene, Gianmarco Ragognetti, Letizia Lorusso, Andrea Viggiano, Angelo Marcelli, Rosa Senatore, Antonio Parziale, S. Stramaglia, M. Pellicoro, L. Angelini, E. Amico, H. Aerts, J. Cortés, S. Laureys, D. Marinazzo, S. Stramaglia, I. Bassez, L. Faes, Hannes Almgren, Adeel Razi, Frederik Van de Steen, Ruth Krebs, Hannelore Aerts, Lida Kanari, Pawel Dlotko, Martina Scolamiero, Ran Levi, Julian Shillcock, Christiaan P.J. de Kock, Kathryn Hess, Henry Markram, Cheng Ly, Gary Marsat, Tom Gillespie, Malin Sandström, Mathew Abrams, Jeffrey S. Grethe, Maryann Martone, Robin De Gernier, Sergio Solinas, Christian Rössert, Marc Haelterman, Serge Massar, Valentina Pasquale, Vito Paolo Pastore, Sergio Martinoia, Paolo Massobrio, Cristiano Capone, Núria Tort-Colet, Maria V. Sanchez-Vives, Maurizio Mattia, Ali Almasi, Shaun L. Cloherty, David B. Grayden, Yan T. Wong, Michael R. Ibbotson, Hamish Meffin, Luke Y. Prince, Krasimira Tsaneva-Atanasova, Jack R. Mellor, Alberto Mazzoni, Manuela Rosa, Jacopo Carpaneto, Luigi M. Romito, Alberto Priori, Silvestro Micera, Rosanna Migliore, Carmen Alina Lupascu, Francesco Franchina, Luca Leonardo Bologna, Armando Romani, Sára Saray, Werner Van Geit, Szabolcs Káli, Alex Thomson, Audrey Mercer, Sigrun Lange, Joanne Falck, Eilif Muller, Felix Schürmann, Dmitrii Todorov, Robert Capps, William Barnett, Yaroslav Molkov, Federico Devalle, Diego Pazó, Ernest Montbrió, Gabriela Mochol, Habiba Azab, Benjamin Y. Hayden, Rubén Moreno-Bote, Pragathi Priyadharsini Balasubramani, Srinivasa V. Chakravarthy, Vignayanandam R. Muddapu, Medorian D. Gheorghiu, Bartul Mimica, Jonathan Withlock, Raul C. Mureșan, Jennifer L. Zick, Kelsey Schultz, Rachael K. Blackman, Matthew V. Chafee, Theoden I. Netoff, Nicholas Roberts, Vivek Nagaraj, Andrew Lamperski, Theoden I. Netoff, Logan L. Grado, Matthew D. Johnson, David P. Darrow, Davide Lonardoni, Hayder Amin, Stefano Di Marco, Alessandro Maccione, Luca Berdondini, Thierry Nieus, Marcel Stimberg, Dan F. M. Goodman, Thomas Nowotny, Veronika Koren, Valentin Dragoi, Klaus Obermayer, Samy Castro, Mariano Fernandez, Wael El-Deredy, Kesheng Xu, Jean Paul Maidana, Patricio Orio, Weiliang Chen, Iain Hepburn, Francesco Casalegno, Adrien Devresse, Aleksandr Ovcharenko, Fernando Pereira, Fabien Delalondre, Erik De Schutter, Peter Bratby, Andrew R. Gallimore, Guido Klingbeil, Criseida Zamora, Yunliang Zang, Patrick Crotty, Eric Palmerduca, Alberto Antonietti, Claudia Casellato, Csaba Erö, Egidio D’Angelo, Marc-Oliver Gewaltig, Alessandra Pedrocchi, Ilja Bytschok, Dominik Dold, Johannes Schemmel, Karlheinz Meier, Mihai A. Petrovici, Hui-An Shen, Simone Carlo Surace, Jean-Pascal Pfister, Baptiste Lefebvre, Olivier Marre, Pierre Yger, Athanasia Papoutsi, Jiyoung Park, Ryan Ash, Stelios Smirnakis, Panayiota Poirazi, Richard A. Felix, Alexander G. Dimitrov, Christine Portfors, Silvia Daun, Tibor I. Toth, Joanna Jędrzejewska-Szmek, Nadine Kabbani, Kim T. Blackwel, Bahar Moezzi, Natalie Schaworonkow, Lukas Plogmacher, Mitchell R. Goldsworthy, Brenton Hordacre, Mark D. McDonnell, Nicolangelo Iannella, Michael C. Ridding, Jochen Triesch, Reinoud Maex, Karen Safaryan, Volker Steuber, Rongxiang Tang, Yi-Yuan Tang, Darya V. Verveyko, Alexey R. Brazhe, Andrey Yu Verisokin, Dmitry E. Postnov, Cengiz Günay, Gabriella Panuccio, Michele Giugliano, Astrid A. Prinz, Pablo Varona, Mikhail I. Rabinovich, Jack Denham, Thomas Ranner, Netta Cohen, Maria Reva, Nelson Rebola, Tekla Kirizs, Zoltan Nusser, David DiGregorio, Eirini Mavritsaki, Panos Rentzelas, Nikul H. Ukani, Adam Tomkins, Chung-Heng Yeh, Wesley Bruning, Allison L. Fenichel, Yiyin Zhou, Yu-Chi Huang, Dorian Florescu, Carlos Luna Ortiz, Paul Richmond, Chung-Chuan Lo, Daniel Coca, Ann-Shyn Chiang, Aurel A. Lazar, Bahar Moezzi, Jennifer L. Creaser, Congping Lin, Peter Ashwin, Jonathan T. Brown, Thomas Ridler, Daniel Levenstein, Brendon O. Watson, György Buzsáki, John Rinzel, Rodica Curtu, Anh Nguyen, Sahand Assadzadeh, Peter A. Robinson, Paula Sanz-Leon, Caroline G. Forlim, Lírio O. B. de Almeida, Reynaldo D. Pinto, Francisco B. Rodríguez, Ángel Lareo, Caroline Garcia Forlim, Francisco B. Rodríguez, Aaron Montero, Thiago Mosqueiro, Ramon Huerta, Francisco B. Rodriguez, Vinicio Changoluisa, Francisco B. Rodriguez, Vinícius L. Cordeiro, César C. Ceballos, Nilton L. Kamiji, Antonio C. Roque, William W. Lytton, Andrew Knox, Joshua J. C. Rosenthal, Silvia Daun, Svitlana Popovych, Liqing Liu, Bin A. Wang, Tibor I. Tóth, Christian Grefkes, Gereon R. Fink, Nils Rosjat, Abraham Perez-Trujillo, Andres Espinal, Marco A. Sotelo-Figueroa, Ivan Cruz-Aceves, Horacio Rostro-Gonzalez, Martin Zapotocky, Martina Hoskovcová, Jana Kopecká, Olga Ulmanová, Evžen Růžička, Matthias Gärtner, Sevil Duvarci, Jochen Roeper, Gaby Schneider, Stefan Albert, Katharina Schmack, Michiel Remme, Susanne Schreiber, Michele Migliore, Carmen A. Lupascu, Luca L. Bologna, Stefano M. Antonel, Jean-Denis Courcol, Felix Schürmann, Sami Utku Çelikok, Eva M. Navarro-López, Neslihan Serap Şengör, Rahmi Elibol, Neslihan Serap Sengor, Mustafa Yasir Özdemir, Tianyi Li, Angelo Arleo, Denis Sheynikhovich, Akihiro Nakamura, Masanori Shimono, Youngjo Song, Sol Park, Ilhwan Choi, Jaeseung Jeong, Hee-sup Shin, Sadra Sadeh, Padraig Gleeson, R. Angus Silver, Alexandra Pierri Chatzikalymniou, Frances K. Skinner, Lazaro M. Sanchez-Rodriguez, Roberto C. Sotero, Loreen Hertäg, Owen Mackwood, Henning Sprekeler, Steffen Puhlmann, Simon N. Weber, David Higgins, Laura B. Naumann, Simon N. Weber, Ramakrisnan Iyer, Stefan Mihalas, Valentina Ticcinelli, Tomislav Stankovski, Peter V. E. McClintock, Aneta Stefanovska, Predrag Janjić, Dimitar Solev, Gerald Seifert, Ljupčo Kocarev, Christian Steinhäuser, Mehrdad Salmasi, Stefan Glasauer, Martin Stemmler, Danke Zhang, Chi Zhang, Armen Stepanyants, Julia Goncharenko, Lieke Kros, Neil Davey, Chris de Zeeuw, Freek Hoebeek, Ankur Sinha, Roderick Adams, Michael Schmuker, Maria Psarrou, Maria Schilstra, Benjamin Torben-Nielsen, Christoph Metzner, Achim Schweikard, Tuomo Mäki-Marttunen, Bartosz Zurowski, Daniele Marinazzo, Luca Faes, Sebastiano Stramaglia, Henry O. C. Jordan, Simon M. Stringer, Elżbieta Gajewska-Dendek, Piotr Suffczyński, Nicoladie Tam, George Zouridakis, Luca Pollonini, Yi-Yuan Tang, Mojtaba Madadi Asl, Alireza Valizadeh, Peter A. Tass, Andreas Nold, Wei Fan, Sara Konrad, Heiko Endle, Johannes Vogt, Tatjana Tchumatchenko, Juliane Herpich, Christian Tetzlaff, Jannik Luboeinski, Timo Nachstedt, Manuel Ciba, Andreas Bahmer, Christiane Thielemann, Eric S. Kuebler, Joseph S. Tauskela, Jean-Philippe Thivierge, Rembrandt Bakker, María García-Amado, Marian Evangelio, Francisco Clascá, Paul Tiesinga, Christopher L. Buckley, Taro Toyoizumi, Alexis M. Dubreuil, Rémi Monasson, Alessandro Treves, Davide Spalla, Sophie Rosay, Florence I. Kleberg, Willy Wong, Bruno de Oliveira Floriano, Toshihiko Matsuo, Tetsuya Uchida, Domenica Dibenedetto, Kâmil Uludağ, Abdorreza Goodarzinick, Maximilian Schmidt, Claus C. Hilgetag, Markus Diesmann, Sacha J. van Albada, Michael Fauth, Mark van Rossum, Manuel Reyes-Sánchez, Rodrigo Amaducci, Carlos Muñiz, Pablo Varona, Irene Elices, David Arroyo, Rafael Levi, Ben Cohen, Carson Chow, Shashaank Vattikuti, Elena Bertolotti, Raffaella Burioni, Matteo di Volo, Alessandro Vezzani, Bayar Menzat, Tim P. Vogels, Nobuhiko Wagatsuma, Susmita Saha, Reena Kapoor, Robert Kerr, John Wagner, Luis C. Garcia del Molino, Guangyu Robert Yang, Jorge F. Mejias, Xiao-Jing Wang, Hanbing Song, Joseph Goodliffe, Jennifer Luebke, Christina M. Weaver, John Thomas, Nishant Sinha, Nikhita Shaju, Tomasz Maszczyk, Jing Jin, Sydney S. Cash, Justin Dauwels, M. Brandon Westover, Maryam Karimian, Michelle Moerel, Peter De Weerd, Thomas Burwick, Ronald L. Westra, Romesh Abeysuriya, Jonathan Hadida, Stamatios Sotiropoulos, Saad Jbabdi, Mark Woolrich, Chama Bensmail, Borys Wrobel, Xiaolong Zhou, Zilong Ji, Xiao Liu, Yan Xia, Si Wu, Xiao Wang, Mingsha Zhang, Si Wu, Netanel Ofer, Orit Shefi, Gur Yaari, Ted Carnevale, Amit Majumdar, Subhashini Sivagnanam, Kenneth Yoshimoto, Elena Y. Smirnova, Dmitry V. Amakhin, Sergey L. Malkin, Aleksey V. Zaitsev, Anton V. Chizhov, Margarita Zaleshina, Alexander Zaleshin, Victor J. Barranca, George Zhu, Quinton M. Skilling, Daniel Maruyama, Nicolette Ognjanovski, Sara J. Aton, Michal Zochowski, Jiaxing Wu, Sara Aton, Scott Rich, Victoria Booth, Maral Budak, Salvador Dura-Bernal, Samuel A. Neymotin, Benjamin A. Suter, Gordon M. G. Shepherd, Melvin A. Felton, Alfred B. Yu, David L. Boothe, Kelvin S. Oie, Piotr J. Franaszczuk, Sergey A. Shuvaev, Batuhan Başerdem, Anthony Zador, Alexei A. Koulakov, Víctor J. López-Madrona, Ernesto Pereda, Claudio R. Mirasso, Santiago Canals, Stefano Masoli, Udaya B. Rongala, Alberto Mazzoni, Anton Spanne, Henrik Jorntell, Calogero M. Oddo, Alexander V. Vartanov, Anastasia K. Neklyudova, Stanislav A. Kozlovskiy, Andrey A. Kiselnikov, Julia A. Marakshina, Maria Teleńczuk, Bartosz Teleńczuk, Alain Destexhe, Paula T. Kuokkanen, Anna Kraemer, Thomas McColgan, Catherine E. Carr, Richard Kempter

**Affiliations:** 1 Department of Neuroscience, Yale University, New Haven, CT 06520 USA; 2 Department Physiology & Pharmacology, SUNY Downstate, Brooklyn, NY 11203 USA; 3 NYU School of Engineering, 6 MetroTech Center, Brooklyn, NY 11201 USA; 4 Kings County Hospital Center, Brooklyn, NY 11203 USA; 5 Departament de Matemàtica Aplicada, Universitat Politècnica de Catalunya, Barcelona, 08028 Spain; 6 Institut de Neurobiologie de la Méditerrannée (INMED), INSERM, UMR901, Aix-Marseille Univ, Marseille, France; 7 Center of Neural Science, New York University, New York, NY USA; 8 Aix-Marseille Univ, INSERM, INS, Inst Neurosci Syst, Marseille, France; 9 Laboratoire de Physique Théorique et Modélisation, CNRS UMR 8089, Université de Cergy-Pontoise, 95300 Cergy-Pontoise Cedex, France; 10 Department of Mathematics and Computer Science, ENSAT, Abdelmalek Essaadi’s University, Tangier, Morocco; 11 Laboratory of Natural Computation, Department of Information and Electrical Engineering and Applied Mathematics, University of Salerno, 84084 Fisciano, SA Italy; 12 Department of Medicine, University of Salerno, 84083 Lancusi, SA Italy; 13 Dipartimento di Fisica, Università degli Studi Aldo Moro, Bari, and INFN, Sezione Di Bari, Italy; 14 Data Analysis Department, Ghent University, Ghent, Belgium; 15 Coma Science Group, University of Liège, Liège, Belgium; 16 Cruces Hospital and Ikerbasque Research Center, Bilbao, Spain; 17 BIOtech, Department of Industrial Engineering, University of Trento, and IRCS-PAT FBK, 38010 Trento, Italy; 18 Department of Data Analysis, Ghent University, Ghent, 9000 Belgium; 19 The Wellcome Trust Centre for Neuroimaging, University College London, London, WC1N 3BG UK; 20 Department of Electronic Engineering, NED University of Engineering and Technology, Karachi, Pakistan; 21 Blue Brain Project, École Polytechnique Fédérale de Lausanne, Lausanne, Switzerland; 22 Departement of Mathematics, Swansea University, Swansea, Wales, UK; 23 Laboratory for Topology and Neuroscience at the Brain Mind Institute, École polytechnique fédérale de Lausanne, Lausanne, Switzerland; 24 Institute of Mathematics, University of Aberdeen, Aberdeen, Scotland, UK; 25 Department of Integrative Neurophysiology, Center for Neurogenomics and Cognitive Research, VU Universiteit Amsterdam, Amsterdam, The Netherlands; 26 Department of Statistical Sciences and Operations Research, Virginia Commonwealth University, Richmond, VA 23284 USA; 27 Biology Department, West Virginia University, Morgantown, WV 26506 USA; 28 Center for Research in Biological Systems, UCSD, La Jolla, CA 92093 USA; 29 INCF Secretariat, Karolinska Institute, Nobels väg 15A, 17177 Stockholm, Sweden; 30 Neurosciences, UCSD, La Jolla, CA 92093 USA; 31 École polytechnique de Bruxelles, Université libre de Bruxelles, Brussels, 1050 Belgium; 32 Department of Biomedical Science, University of Sassari, 07100 Sassari, Italy; 33 Blue Brain Project, École polytechnique fédérale de Lausanne, Biotech Campus, Geneva, 1202 Switzerland; 34 Neuroscience and Brain Technologies Department, Istituto Italiano di Tecnologia (IIT), Genoa, Italy; 35 Department of Informatics, Bioengineering, Robotics, System Engineering (DIBRIS), University of Genova, Genoa, Italy; 36 Istituto Superiore di Sanità (ISS), 00161 Rome, Italy; 37 Sapienza University, 00185 Rome, Italy; 38 Institut d’Investigacions Biomèdiques August Pi i Sunyer (IDIBAPS), 08036 Barcelona, Spain; 39 Institució Catalana de Recerca i Estudis Avançats (ICREA), 08010 Barcelona, Spain; 40 National Vision Research Institute, Australian College of Optometry, Melbourne, Australia; 41 NeuroEngineering Laboratory, Department Biomedical Eng., University of Melbourne, Melbourne, Australia; 42 Department of Electrical & Computer Systems Eng., Monash University, Melbourne, Australia; 43 Department of Physiology, Monash University, Melbourne, Australia; 44 ARC Centre of Excellence for Integrative Brain Function, University of Melbourne, Melbourne, Australia; 45 Centre for Synaptic Plasticity, School of Physiology, Pharmacology, and Neuroscience, University of Bristol, Bristol, BS8 1TD UK; 46 Department of Mathematic, College of Engineering, Mathematics and Physical Sciences, University of Exeter, Exeter, EX4 4QF UK; 47 EPRSC Centre for Predictive Modelling in Healthcare, University of Exeter, Exeter, EX4 4QJ UK; 48 Translational Neural Engineering, The Biorobotics Institute, Scuola Superiore Sant’Anna, 56025 Pontedera, Italy; 49 Clinical Center for Neurostimulation, Neurotechnology and Movement Disorders Fondazione, IRCCS Ca’ Granda Ospedale Maggiore Policlinico, 20122 Milan, Italy; 50 Movement Disorders Department, Neurological Institute Carlo Besta, 20133 Milan, Italy; 51 Department of Health Sciences, University of Milan & ASST Santi Paolo e Carlo, 20142 Milan, Italy; 52 Bertarelli Foundation Chair in Translational NeuroEngineering, Institute of Bioengineering and Center for Neuroprosthetics, Ecole Polytechnique Federale De Lausanne, Lausanne, 1015 Switzerland; 53 Institute of Biophysics, National Research Council (CNR), Palermo, Italy; 54 Institute of Experimental Medicine, Hungarian Academy of Sciences, Budapest, Hungary; 55 University College London, London, UK; 56 Department of Mathematics and Statistics, Georgia State University, Atlanta, GA 30303-3083 USA; 57 Center for Brain and Cognition, Universitat Pompeu Fabra, 08018 Barcelona, Spain; 58 Department of Physics, Lancaster University, Lancaster, LA1 4YB UK; 59 Instituto de Fisica de Cantabria (IFCA), CSIC-Universidad de Cantabria, 39005 Santander, Spain; 60 Center for Brain and Cognition and Department of Information and Communications Technologies, University Pompeu Fabra, Barcelona, 08005 Spain; 61 Department of Brain and Cognitive Sciences and Center for Visual Sciences, University of Rochester, Rochester, NY 14618 USA; 62 Brain and Cognitive Sciences, University of Rochester, Rochester, NY 14627 USA; 63 Bhupat and Jyoti Mehta School of Biosciences, Department of Biotechnology, IIT- Madras, Chennai, TN India; 64 Romanian Institute of Science and Technology, Cluj-Napoca, Cluj, 400552 Romania; 65 Centre for Neural Computation, Kavli Institute for Systems Neuroscience, Trondheim, 7491 Norway; 66 Graduate Program in Neuroscience, University of Minnesota, Minneapolis, MN 55455 USA; 67 Medical Scientist Training Program, University of Minnesota, Minneapolis, MN 55455 USA; 68 Department of Biomedical Engineering, University of Minnesota, Minneapolis, MN 55455 USA; 69 Brain Sciences Center, VA Medical Center, Minneapolis, MN 55417 USA; 70 Department of Electrical and Computer Engineering, University of Minnesota, Minneapolis, MN 55455 USA; 71 Neuroscience and Brain Technology Department, Fondazione Istituto Italiano di Tecnologia, 16163 Genoa, Italy; 72 Scienze cliniche applicate e biotecnologiche, Università dell’Aquila, 67100 L’Aquila, Italy; 73 Department of Biomedical and Clinical Sciences, “Luigi Sacco”, University of Milan, Milan, Italy; 74 Sorbonne Universités, UPMC Univ Paris 06, INSERM, CNRS, Institut de la Vision, Paris, France; 75 Department of Electrical and Electronic Engineering, Imperial College, London, UK; 76 School of Engineering and Informatics, University of Sussex, Brighton, UK; 77 Neural Information Processing Group, Institute of Software Engineering and Theoretical Computer Science, Technische Universität Berlin, 10587 Berlin, Germany; 78 Bernstein Center for Computational Neuroscience Berlin, Berlin, Germany; 79 Department of Neurobiology and Anatomy, University of Texas Medical School, Houston, TX 77030 USA; 80 Centro Interdisciplinario de Neurociencia de Valparaíso, Universidad de Valparaíso, 2360102 Valparaíso, Chile; 81 Programa de Doctorado en Ciencias, mención en Neurociencia, Facultad de Ciencias, Universidad de Valparaíso, 2360102 Valparaíso, Chile; 82 Laboratorio de Electrónica Industrial, Control e Instrumentación, Universidad Nacional de La Plata, La Plata, Argentina; 83 Escuela de Ingeniería Biomédica, Universidad de Valparaíso, 2362905 Valparaíso, Chile; 84 Instituto de Neurociencia, Facultad de Ciencias, Universidad de Valparaíso, 2360102 Valparaíso, Chile; 85 Computational Neuroscience Unit, Okinawa Institute of Science and Technology Graduate University, Onna-son, Okinawa, 904-0895 Japan; 86 Okinawa Institute of Science and Technology Graduate University, 1919-1 Tancha, Onna-son, Kunigami-gun, Okinawa, 904-0495 Japan; 87 Computational Neuroscience Unit, Okinawa Institute of Science and Technology, 1919-1 Tancha, Onna-son, Kunigami-gun, Okinawa, 904-0495 Japan; 88 Department of Physics and Astronomy, Colgate University, Hamilton, NY 13346 USA; 89 Department of Electronics, Information and Bioengineering, Politecnico di Milano, Milan, Italy; 90 Department of Brain and Behavioural Science, Neurophysiology and Neurocomputation Unit, University of Pavia, Via Forlanini 6, 27100 Pavia, Italy; 91 Kirchhoff-Institute for Physics, Heidelberg University, Im Neuenheimer Feld 227, 69120 Heidelberg, Germany; 92 Department of Physiology, University of Bern, Bühlplatz 5, 3012 Bern, Switzerland; 93 Institute of Neuroinformatics, UZH and ETHZ, Zurich, 8057 Switzerland; 94 Institut de la Vision, INSERM UMRS 968, CNRS UMR, 7210 Paris, France; 95 IMBB, FORTH, 70013 Heraklion, Crete, Greece; 96 Neurology, Baylor College of Medicine, Houston, TX 77030 USA; 97 Department of Integrative Biology and Neuroscience, Washington State University Vancouver, Vancouver, WA 98686 USA; 98 Department of Mathematics and Statistics, Washington State University Vancouver, Vancouver, WA 98686 USA; 99 Department of Animal Physiology, Institute of Zoology, University of Cologne, 50674 Cologne, Germany; 100 Cognitive Neuroscience, Institute of Neuroscience and Medicine (INM-3), Research Center Juelich, 52425 Juelich, Germany; 101 Krasnow Institute, George Mason University, Fairfax, VA 22030 USA; 102 School of Systems Biology, George Mason University, Fairfax, VA 22030 USA; 103 Bioengineering Department, George Mason University, Fairfax, VA 22030 USA; 104 Computational and Theoretical Neuroscience Laboratory, School of Information Technology and Mathematical Sciences, University of South Australia, Adelaide, Australia; 105 Robinson Research Institute, School of Medicine, University of Adelaide, Adelaide, Australia; 106 Frankfurt Institute for Advanced Studies, Frankfurt, Germany; 107 Discipline of Psychiatry, School of Medicine, University of Adelaide, Adelaide, Australia; 108 School of Mathematical Sciences, University of Nottingham, Nottingham, UK; 109 Department of Cognitive Sciences, Ecole Normale Supérieure, rue d’Ulm 25, 75005 Paris, France; 110 Department of Physics and Astronomy, Knudsen Hall, University of California, Los Angeles, CA 90095-0001 USA; 111 Centre for Computer Science and Informatics Research, University of Hertfordshire, College Lane, Hatfield AL10 9AB UK; 112 Department of Psychological & Brain Sciences, Washington University in St. Louis, St. Louis, MO 63130 USA; 113 Department of Psychological Sciences, Texas Tech University, Lubbock, TX 79409 USA; 114 Department of Theoretical Physics, Kursk State University, Kursk, Russian Federation 305000; 115 Department of Biophysics, Lomonosov Moscow State University, Moscow, Russian Federation 119991; 116 Department of Physics, Saratov State National Research University, Saratov, Russian Federation 410012; 117 Department Biology, Emory University, Atlanta, GA 30322 USA; 118 Grupo de Neurocomputación Biológica, Departamento de Ingeniería Informática, Escuela Politécnica Superior, Universidad Autónoma de Madrid, Madrid, Spain; 119 BioCircuits Institute, University of California, San Diego, CA USA; 120 School of Computing, University of Leeds, Leeds, LS2 9JT UK; 121 Laboratory of Dynamic Neuronal Imaging, Neuroscience Department, Institute Pasteur, 75015 Paris, France; 122 Department of Psychology, Birmingham City University, Birmingham, UK; 123 School of Psychology, University of Birmingham, Birmingham, UK; 124 Department of Electrical Engineering, Columbia University, New York, NY 10027 USA; 125 Department of Automatic Control & Systems Engineering, The University of Sheffield, Sheffield, S1 3JD UK; 126 Department of Computer Science, Columbia University, New York, NY 10027 USA; 127 Data Science Institute, Columbia University, New York, NY 10027 USA; 128 Brain Research Center, National Tsing Hua University, Hsinchu, 30013 Taiwan; 129 Department of Computer Science, The University of Sheffield, Sheffield, S1 4DP UK; 130 Department of Mathematics, University of Exeter, Exeter, EX4 4QD UK; 131 Institute of Biomedical and Clinical Sciences, University of Exeter Medical School, Exeter, EX4 4PS UK; 132 Center for Neural Science, New York University, New York, NY 10003 USA; 133 NYU Neuroscience Institute, New York University, New York, NY 10016 USA; 134 Department of Psychiatry, Weill Cornell Medical Center, New York, NY 10065 USA; 135 Courant Institute for Mathematical Sciences, New York University, New York, NY 10012 USA; 136 Department of Mathematics, The University of Iowa, Iowa City, IA 52242 USA; 137 School of Physics, The University of Sydney, Sydney, NSW 2006 Australia; 138 Center for Integrative Brain Function, The University of Sydney, Sydney, NSW 2006 Australia; 139 Clinic and Policlinic for Psychiatry and Psychotherapy, University Medical Center Hamburg-Eppendorf, Hamburg, 20246 Germany; 140 Departamento de Física Geral, Universidade de Sao Paulo, Sao Paulo, 05508-090 Brazil; 141 Instituto de Física de Sao Carlos, Universidade de Sao Paulo, Sao Carlos, 13560-970 Brazil; 142 Clinic and Policlinic for Psychiatry and Psychotherapy, University Medical Center, Hamburg-Eppendorf, Hamburg, Germany; 143 Lab. Neurodynamics/Neurobiophysics - Department Physics and Interdisciplinary Sciences - Institute of Physics of São Carlos, Universidade de São Paulo, São Paulo, Brazil; 144 BioCircuits Institute, University of California, San Diego, La Jolla, CA 92093-0402 USA; 145 Universidad Politécnica Salesiana, Quito, Ecuador; 146 Departamento de Física-FFCLRP, Universidade de São Paulo, Ribeirão Preto, São Paulo, SP 14040-901 Brazil; 147 Department of Neurology, Kings County Hospital, Brooklyn, NY 11203 USA; 148 Department of Neurology, University of Wisconsin, Madison, WI 53705 USA; 149 Department of Neurobiology, Marine Biological Laboratory, Woods Hole, MA 02543 USA; 150 Heisenberg Research Group of Computational Neuroscience – Modeling Neural Network Function, Department of Animal Physiology, Institute of Zoology, University of Cologne, 50674 Cologne, Germany; 151 Department of Neurology, University Hospital Cologne, 50937 Cologne, Germany; 152 Department of Electronics, University of Guanajuato, Salamanca, Guanajuato, 36885 Mexico; 153 Department of Organizational Studies, University of Guanajuato, 3625 Guanajuato, Mexico; 154 CONACYT, Mathematics Research Center (CIMAT), Guanajuato, 36000 Mexico; 155 Institute of Physiology, Czech Academy of Sciences, 14220 Prague, Czech Republic; 156 Department of Neurology, First Faculty of Medicine, Charles University, 120 00 Prague, Czech Republic; 157 Institute of Mathematics, Goethe-University, Frankfurt, Germany; 158 Neuroscience Center, Institute of Neurophysiology, Goethe-University, Frankfurt, Germany; 159 Department of Psychiatry and Psychotherapy, Charité Universitätsmedizin, Berlin, Germany; 160 Institute for Theoretical Biology, Humboldt University, 10115 Berlin, Germany; 161 Bernstein Center for Computational Neuroscience, Berlin, Germany; 162 Biomedical Engineering Department, Boğaziçi University, Istanbul, 34342 Turkey; 163 School of Computer Science, The University of Manchester, Manchester, M13 9PL UK; 164 Electronics and Communication Engineering Department, Istanbul Technical University, Istanbul, 34469 Turkey; 165 Sorbonne Universités, UPMC Univ Paris 06, INSERM, CNRS, Institut de la Vision, 17 rue Moreau, 75012 Paris, France; 166 Osaka University, Toyonaka, Osaka Japan; 167 Riken Brain Science Institute, Saitama, Japan; 168 Department of Bio and Brain Engineering, KAIST, Daejeon, 34141 Republic of Korea; 169 Center for Cognition and Sociality, IBS, Daejeon, 34047 Republic of Korea; 170 Program of Brain and Cognitive Engineering, KAIST, Daejeon, 34141 Republic of Korea; 171 Department of Neuroscience, Physiology and Pharmacology, University College London, London, WC1E 6BT UK; 172 Krembil Research Institute, University Health Network, Toronto, ON Canada; 173 Department of Physiology, University of Toronto, Toronto, ON Canada; 174 Department of Medicine (Neurology), University of Toronto, Toronto, ON Canada; 175 Hotchkiss Brain Institute and Department of Radiology, University of Calgary, Calgary, AB T2N 1N4 Canada; 176 Modelling of Cognitive Processes, Berlin Institute of Technology and Bernstein Center for Computational Neuroscience, 10587 Berlin, Germany; 177 Bernstein Center for Computational Neuroscience, 10115 Berlin, Germany; 178 Modelling of Cognitive Processes, TU Berlin, 10587 Berlin, Germany; 179 Allen Institute for Brain Science, Seattle, WA 98109 USA; 180 Department of Physics, Lancaster University, LA1 4YB Lancaster, UK; 181 Faculty of Medicine, Ss Cyril and Methodius University, Skopje, 1000 Macedonia; 182 Laboratory for Complex Systems and Networks, Macedonian Academy of Sciences and Arts, Skopje, Macedonia; 183 Macedonian Academy of Sciences and Arts, Skopje, Macedonia; 184 Institute of Cellular Neurosciences, University of Bonn Medical School, Bonn, Germany; 185 Graduate School of Systemic Neurosciences, Ludwig-Maximilian University, Munich, Germany; 186 Bernstein Center for Computational Neuroscience, Munich, Germany; 187 German Center for Vertigo and Balance Disorders, Ludwig-Maximilian University, Munich, Germany; 188 Department of Neurology, Ludwig-Maximilian University, Munich, Germany; 189 Department of Biology II, Ludwig-Maximilian University, Munich, Germany; 190 Department of Physics and Center for Interdisciplinary Research on Complex Systems, Northeastern University, Boston, MA 02115 USA; 191 Department of Neuroscience, Erasmus MC, Wytemaweg 80, 3015 CN Rotterdam, The Netherlands; 192 UH Biocomputation Group, University of Hertfordshire, Hatfield, AL10 9AB UK; 193 Institute for Robotics and Cognitive Systems, University of Luebeck, 23562 Luebeck, Germany; 194 NORMENT, Institute of Clinical Medicine, University of Oslo, Oslo, Norway; 195 Department of Psychiatry, University of Luebeck, Schleswig-Holstein, 23562 Luebeck, Germany; 196 Dipartimento di Fisica, Università degli Studi Aldo Moro, Bari, and INFN, Sezione di Bari, 70123 Bari, Italy; 197 OFTNAI, Department Experimental Psychology, University of Oxford, South Parks Road, Oxford, OX1 3UD UK; 198 Department of Biomedical Physics, Institute of Experimental Physics, University of Warsaw, Warsaw, 02-093 Poland; 199 Department of Biological Sciences, University of North Texas, Denton, TX 76203 USA; 200 Departments of Engineering Technology, Computer Science, and Electrical and Computer Engineering, University of Houston, Houston, TX 77204 USA; 201 Department of Physics, Institute for Advanced Studies in Basic Sciences (IASBS), Zanjan, 45195-1159 Iran; 202 School of Cognitive Sciences, Institute for Research in Fundamental Sciences (IPM), Tehran, 19395-5746 Iran; 203 Department of Neurosurgery, School of Medicine, Stanford University, Stanford, CA 94305 USA; 204 Theory of Neural Dynamics, Max Planck Institute for Brain Research, 60438 Frankfurt, Germany; 205 Institute for Microscopic Anatomy and Neurobiology, University Medical Center, Johannes Gutenberg University, 55131 Mainz, Germany; 206 Third Institute of Physics – Biophysics, Department of Computational Neuroscience, University of Goettingen, 37077 Goettingen, Germany; 207 Bernstein Center for Computational Neuroscience, University of Goettingen, 37077 Goettingen, Germany; 208 Biomems Lab, Faculty of Engineering, UAS Aschaffenburg, 63743 Aschaffenburg, Germany; 209 Comprehensive Hearing Center, University ENT-Clinic Würzburg, 97080 Würzburg, Germany; 210 School of Psychology, Faculty of Social Science, University of Ottawa, Ottawa, ON Canada; 211 Human Health Therapeutics, National Research Council of Canada, Ottawa, ON Canada; 212 Neuroinformatics Department, Donders Institute for Brain, Cognition and Behaviour, Radboud University Nijmegen, Nijmegen, The Netherlands; 213 Institute of Neuroscience and Medicine (INM-6) and Institute for Advanced Simulation (IAS-6) and JARA BRAIN Institute I, Jülich Research Centre, Jülich, Germany; 214 Departamento de Anatomía, Histología y Neurociencia, Facultad de Medicina, Universidad Autónoma de Madrid, Madrid, Spain; 215 Informatics, University of Sussex, Brighton, BN1 9RH UK; 216 Brain Science Institute, RIKEN, Wako, Saitama 351-0106 Japan; 217 Laboratoire de Physique Théorique, Ecole Normale Supérieure, Paris, France; 218 Cognitive Neuroscience Sector, SISSA, 34136 Trieste, Italy; 219 Neuroscience Lab, Frankfurt Institute for Advanced Studies, 60438 Frankfurt Am Main, Hessen, Germany; 220 Department of Electrical and Computer Engineering, University of Toronto, Toronto, ON M5S3G4 Canada; 221 Institute of Biomaterials and Biomedical Engineering, University of Toronto, Toronto, ON M5S3G9 Canada; 222 Ophthalmology, Okayama University Medical School, Okayama, 700-8558 Japan; 223 Polymer Materials Science, Faculty of Engineering, Okayama University, Okayama, 700-8530 Japan; 224 Maastricht Centre for Systems Biology (MaCSBio), Maastricht University, Maastricht, The Netherlands; 225 Maastricht Brain Imaging Centre (MBIC), Faculty of Psychology & Neuroscience, Maastricht University, Maastricht, The Netherlands; 226 Department of Physics, Institute for Advanced Studies in Basic Sciences (IASBS), Zanjan, 45137-66731 Iran; 227 Institute of Neuroscience and Medicine (INM-6) and Institute for Advanced Simulation (IAS-6), Jülich Research Centre and JARA, Jülich, Germany; 228 Laboratory for Neural Circuit Theory, RIKEN Brain Science Institute, Wako, Japan; 229 Department of Computational Neuroscience, University Medical Center Eppendorf, Hamburg, Germany; 230 Department of Health Sciences, Boston University, Boston, MA USA; 231 Department of Psychiatry, Psychotherapy and Psychosomatics, Medical Faculty, RWTH Aachen University, Aachen, Germany; 232 Department of Physics, Faculty 1, RWTH Aachen University, Aachen, Germany; 233 School of Informatics, University of Edinburgh, Edinburgh, UK; 234 Department of Biological Sciences, University of Southern California, Los Angeles, CA USA; 235 Lab of Biological Modeling, NIDDK/NIH, Bethesda, MD 20814 USA; 236 Department of Mathematical, Physical and Computer Sciences, University of Parma, 43124 Parma, Italy; 237 INFN, Gruppo Collegato di Parma, 43124 Parma, Italy; 238 Group for Neural Theory, Departément des Etudes Cognitives, Ecole Normale Supérieure, Paris, France; 239 Centro Interdipartimentale per lo Studio delle Dinamiche Complesse, 1-50019 Sesto Fiorentino, Italy; 240 Indiana University–Purdue University, Indianapolis, IN 46202 USA; 241 IMEM-CNR, 43124 Parma, Italy; 242 Centre for Neural Circuits and Behaviour, University of Oxford, Oxford, UK; 243 School of Science and Engineering, Tokyo Denki University, Hiki, Saitama 350-0394 Japan; 244 IBM Research Australia, Melbourne, VIC 3006 Australia; 245 NeuroEngineering Laboratory, Electrical & Electronic Engineering, The University of Melbourne, Melbourne, Australia; 246 NYU-ECNU Institute of Brain and Cognitive Science, NYU Shanghai, Shanghai, 200122 China; 247 Department of Mathematics, Franklin and Marshall College, Lancaster, PA 17604 USA; 248 Department of Anatomy and Neurobiology, Boston University School of Medicine, Boston, MA 02118 USA; 249 School of Electrical and Electronic Engineering, Nanyang Technological University, 639798 Singapore, Singapore; 250 Institute of Neuroscience, Faculty of Medical Sciences, Newcastle University, Newcastle upon Tyne, UK; 251 School of Computing Science, Newcastle University, Newcastle upon Tyne, UK; 252 Department of Electrical and Electronics Engineering, Vellore Institute of Technology, Vellore, Tamil Nadu India; 253 Neurology Department, Massachusetts General Hospital and Harvard Medical School, Boston, MA USA; 254 Department of Cognitive Neuroscience, Maastricht University, 6229 ER Maastricht, The Netherlands; 255 Frankfurt Institute for Advanced Studies, Goethe University Frankfurt, 60438 Frankfurt Am Main, Germany; 256 Department of Data Science and Knowledge Engineering, Maastricht University, 6211 LH Maastricht, The Netherlands; 257 Oxford Centre for Human Brain Activity, Oxford, UK; 258 Oxford Centre for Functional MRI of the Brain, Oxford, UK; 259 Biology, Adam Mickiewicz University, 61-712 Poznan, Poland; 260 Systems Modelling IOPAN, 81-701 Sopot, Poland; 261 School of Systems Science, Beijing Normal University, Beijing, 100875 China; 262 State Key Laboratory of Cognitive Neuroscience & Learning, IDG/McGovern Institute for Brain Research, Beijing Normal University, Beijing, 100875 China; 263 Faculty of Engineering, Bar Ilan University, Ramat Gan, 52900 Israel; 264 Institute for Nanotechnology and Advanced Materials, Bar Ilan University, Ramat Gan, 5290002 Israel; 265 Neuroscience Department, Yale University, New Haven, CT 06510 USA; 266 San Diego Supercomputer Center, University of California San Diego, San Diego, CA 92093-0505 USA; 267 Ioffe Institute, St.-Petersburg, Russia 194021; 268 Sechenov Institute of Evolutionary Physiology and Biochemistry of RAS, St.-Petersburg, Russia 194223; 269 Moscow Institute of Physics and Technology, Moscow, Russia 117303; 270 Department of Mathematics and Statistics, Swarthmore College, Swarthmore, PA 19081 USA; 271 Biophysics Program, University of Michigan, Ann Arbor, MI 48109 USA; 272 Department of Physics, University of Michigan, Ann Arbor, MI 48109 USA; 273 Department of Molecular, Cellular, and Developmental Biology, University of Michigan, Ann Arbor, MI 48109 USA; 274 Applied Physics Program, University of Michigan, Ann Arbor, MI 48109 USA; 275 Applied and Interdisciplinary Mathematics Program, University of Michigan, Ann Arbor, MI 48104 USA; 276 Departments of Anesthesiology and Mathematics, University of Michigan, Ann Arbor, MI 48104 USA; 277 Departments of Physics and Biophysics, University of Michigan, Ann Arbor, MI 48104 USA; 278 Department Neuroscience, Brown University, Providence, RI 02912 USA; 279 Institute of Science and Technology (IST) Austria, 3400 Klosterneburg, Austria; 280 Department Physiology, Northwestern University, Chicago, IL 60611 USA; 281 U. S. Army Research Laboratory, Adelphi, MD 20783 USA; 282 U. S. Army Research Laboratory, Aberdeen Proving Ground, MD 21005 USA; 283 Department of Neurology, Johns Hopkins University School of Medicine, Baltimore, MD 21287 USA; 284 Cold Spring Harbor Laboratory, Cold Spring Harbor, NY 11724 USA; 285 Instituto de Neurociencias, Consejo Superior de Investigaciones Científicas, Universidad Miguel Hernández, Sant Joan d’Alacant, 03550 Spain; 286 Departamento de Ingeniería Industrial, Escuela Superior de Ingeniería y Tecnología, Universidad de La Laguna Avda, Astrofísico Fco. Sanchez, s/n, La Laguna, Tenerife, 38205 Spain; 287 Instituto de Física Interdisciplinar y Sistemas Complejos, CSIC-UIB, Campus Universitat de les Illes Balears, 07122 Palma De Mallorca, Spain; 288 Brain Connectivity Center, Istituto Neurologico IRCCS C. Mondino, Pavia, 27100 Italy; 289 The BioRobotics Institute, Scuola Superiore Sant’Anna, Pontedera, 56025 Pisa, Italy; 290 Neural Basis of Sensorimotor Control, Department of Experimental Medical Science, Lund University, Lund, Sweden; 291 Department of Psychology, Lomonosov Moscow State University, Moscow, Russia; 292 Psychological Institute of Russian Academy of Education, Moscow, Russia; 293 European Institute for Theoretical Neuroscience, Paris, France; 294 Unité de Neurosciences, Information et Complexité, Gif-Sur-Yvette, France; 295 Inst. For Theoretical Biology, Humboldt-Universitaet Zu Berlin, 10115 Berlin, Germany; 296 Bernstein Center for Computational Neuroscience Berlin, 10115 Berlin, Germany; 297 Department of Biology, University of Maryland, College Park, MD 20742 USA

## P156 Multiscale modeling of ischemic stroke with the NEURON reaction-diffusion module

### Adam J. H. Newton^1,2^, Alexandra H. Seidenstein^2,3^, Robert A. McDougal^1^, William W. Lytton^2,4^

#### ^1^Department of Neuroscience, Yale University, New Haven, CT 06520, USA; ^2^Department Physiology & Pharmacology, SUNY Downstate, Brooklyn, NY 11203, USA; ^3^NYU School of Engineering, 6 MetroTech Center, Brooklyn, NY 11201, USA; ^4^Kings County Hospital Center, Brooklyn, NY 11203, USA

##### Correspondence: Adam J. H. Newton (adam.newton@yale.edu)

*BMC Neuroscience* 2017, **18** (**Suppl 1**):P156

Ischemic stroke is fundamentally a multiscale phenomenon [1]. Occlusion of blood vessels in the brain triggers a cascade of changes including: 1. synaptic glutamate release, related to excitotoxicity; 2. elevated extracellular potassium, leading to spreading depression; 3. cell swelling, reducing the extracellular volume and diffusion; 4. production of reactive oxygen species, which give rise to inflammation. These cascades occur over multiple time-scales, with the initial rapid changes in cell metabolism and ionic concentrations trigging several damaging agents that may ultimately leads to cell death. Tissue affected by ischemic stroke is divided into three regions; 1. a core where cells suffer irreparable damage and death, 2. a penumbra where cells may recover with reperfusion, 3. a further region of edema where spontaneous recovery is expected. Multiscale modeling and multiphysics modeling is essential to capture this cascade. Such modeling requires coupling complex intracellular molecular alterations with electrophysiology, and consideration of network properties in the context of bulk tissue alterations mediated by extracellular diffusion.

Spreading depression is a wave of depolarization that propagates through tissue and causes cells in the penumbra to expend energy by repolarization, increasing their vulnerability to cell death. We modeled the spreading depression seen in ischemic stroke by coupling a detailed biophysical model of cortical pyramidal neurons equipped with Na^+^/K^+^-ATPase pumps with reaction-diffusion of ions in the extracellular space (ECS). A macroscopic view of the ECS is characterised by its tortuosity (a reduction in the diffusion coefficient due to obstructions) and its free volume fraction (typically ~20%). The addition of reactions allows the ECS be modeled as an active medium glial buffering of K^+^. Ischemia impedes ATP production which results in a failure of the Na^+^/K^+^-ATPase pump and a rise in extracellular K^+^. Once extracellular K^+^ exceeds a threshold it will cause neurons to depolarize, further increasing extracellular K^+^.

NEURON’s reaction-diffusion module NRxD [2] provides a platform where detailed neurons models can be embedded in a macroscopic model of tissue. This is demonstrated with a multiscale biophysical model of ischemic stroke where the rapid intracellular changes are coupled with the slower diffusive signaling.

**Acknowledgements**

Research supported by NIH grant 5R01MH086638

**References**

1. Newton, AJH, and Lytton, WW: Computer modeling of ischemic stroke. *Drug Discovery Today: Disease Models.* 2017.

2. McDougal RA, Hines ML, Lytton WW: Reaction-diffusion in the NEURON simulator. *Frontiers in neuroinformatics.* 2013, **7**(28).

## P157 Accelerating NEURON reaction-diffusion simulations

### Robert A. McDougal^1^, William W. Lytton^2,3^

#### ^1^Neuroscience, Yale University, New Haven, CT 06520, USA; ^2^Physiology & Pharmacology, SUNY Downstate Medical Center, Brooklyn, NY 11203, USA; ^3^Kings County Hospital, Brooklyn, NY 11203, USA

##### Correspondence: Robert A. McDougal (robert.mcdougal@yale.edu)

*BMC Neuroscience* 2017, **18** (**Suppl 1**):P157

A neuron’s electrical activity is governed not just by presynaptic activity, but also by its internal state. This state is a function of history including prior synaptic input (e.g. cytosolic calcium concentration, protein expression in SCN neurons), cellular health, and routine biological processes. The NEURON simulator [1], like much of computational neuroscience, has traditionally focused on electrophysiology. NEURON has included NRxD to give standardized support for reaction-diffusion (i.e. intracellular) modeling for the past 5 years [2], facilitating studies into the role of electrical-chemical interactions. The original reaction-diffusion support was written in vectorized Python, which offered limited performance, but ongoing improvements have now significantly reduced run-times, making larger-scale studies more practical.

New accelerated reaction-diffusion methods are being developed as part of a separate NEURON module, crxd. This new module will ultimately be a fully compatible replacement for the existing NRxD module (rxd). Developing it as a separate module allows us to make it available to the community before it supports the full functionality of NRxD. The interface code for crxd remains in Python, but it now transfers model structure to C code via ctypes, which performs all run-time calculations; Python is no longer invoked during simulation. Dynamic code generation allows arbitrary reaction schemes to run at full compiled speed. Thread-based parallelization accelerates extracellular reaction-diffusion simulations.

Preliminary tests suggest an approximately 10x reduction in 1D run-time using crxd instead of the Python-based rxd. Like rxd, crxd uses the Hines method [3] for O(n) 1D reaction-diffusion simulations. Using 4 cores for extracellular diffusion currently reduces the runtime by a factor of 2.3. Additionally, using the crxd module simplifies setup relative to rxd-based simulations since it does not require installing scipy.

Once crxd supports the entire documented NRxD interface and has been thoroughly tested, it will replace the rxd module and thus become NEURON’s default module for specifying reaction-diffusion kinetics.

**Acknowledgements**

Research supported by NIH R01 MH086638.

**References**

1. NEURON | for empirically based simulations of neurons and networks of neurons [http://neuron.yale.edu]

2. McDougal RA, Hines ML, Lytton WW: Reaction-diffusion in the NEURON simulator. *Front. Neuroinform* 2013, **7:**28.

3. Hines M: Efficient computation of branched nerve equations. *Int. J. Bio*-*Medical Computing* 1984, **15**:69–76.

## P158 Computation of invariant objects in the analysis of periodically forced neural oscillators

### Alberto Pérez-Cervera, Gemma Huguet, Tere M-Seara

#### Departament de Matemàtica Aplicada, Universitat Politècnica de Catalunya, Barcelona, E-08028, Spain

##### Correspondence: Alberto Pérez-Cervera (alberto.perez@upc.edu)

*BMC Neuroscience* 2017, **18** (**Suppl 1**):P158

Background oscillations, reflecting the excitability of neurons, are ubiquitous in the brain. Some studies have conjectured that when spikes sent by one population reach the other population in the peaks of excitability, then information transmission between two oscillating neuronal groups is more effective [1]. In this context, the phase relationship between oscillating neuronal populations may have implications in neuronal communication between brain areas [2, 3]. The Phase Response Curve (PRC) of a neural oscillator measures the phase-shift resulting from perturbing the oscillator at different phases of the cycle. It provides useful information to understand how phase-locking relationships between neural oscillators emerge but only when perturbations are weak and amplitude is not taken into account.

In this work, we consider a population rate model [4] and perturb it with a time-dependent input. In order to study the phase-locking relationships that emerge, we use the stroboscopic map to perform a bifurcation analysis as a function of the amplitude and frequency of the perturbation. We observe the existence of bistable solutions for some regions of the parameters space, suggesting that, for a given input, populations may operate in different regimes. Furthermore, we apply powerful computational methods [5] to compute the invariant objects for the stroboscopic map, providing a framework that enlarges the PRC comprehension of the perturbative effects in the phase dynamics.

**References**

1. Fries P: A mechanism for cognitive dynamics: neuronal communication through neuronal coherence. *Trends in cognitive sciences* 2005, **9(10):**474–48

2. Tiesinga PH, Sejnowski TJ: Mechanisms for phase shifting in cortical networks and their role in communication through coherence. *Frontiers in human neuroscience* 2010, **4:**196.

3. Canavier CC: Phase-resetting as a tool of information transmission. *Current opinion in neurobiology* 2015, **31:** 206–213.

4. Wilson HR, Cowan JD: Excitatory and inhibitory interactions in localized populations of model neurons. *Biophysical journal* 1972, **12.1:**1–24.

5. Haro À, Canadell M, Figueras JL, Luque A, Mondelo JM: *The Parameterization Method for Invariant Manifolds* 2016. Springer.

## P159 Computational model of spatio-temporal coding in CA3 with speed-dependent theta oscillation

### Caroline Haimerl^1,2^, David Angulo-Garcia^1,3^, Alessandro Torcini^1,3,4^, Rosa Cossart^1^, Arnaud Malvache^1^

#### ^1^Institut de Neurobiologie de la Méditerrannée (INMED), INSERM, UMR901, Aix-Marseille Univ, Marseille, France; ^2^Center of Neural Science, New York University, New York, NY, USA; ^3^Aix-Marseille Univ, INSERM, INS, Inst Neurosci Syst, Marseille, France; ^4^Laboratoire de Physique Théorique et Modélisation, CNRS UMR 8089, Université de Cergy-Pontoise, F-95300 Cergy-Pontoise Cedex, France

##### Correspondence: Caroline Haimerl (david.angulo-garcia@univ-amu.fr)

*BMC Neuroscience* 2017, **18** (**Suppl 1**):P159

Recent studies have demonstrated the capacity of hippocampal sequences associated with theta oscillation, to encode spatio-temporal information. In particular, cells in CA1 become active sequentially in a stable unidirectional order during spontaneous run periods and under minimal external cues [1]. This sequential activity seems to integrate either the distance that the animal has run or the time that has elapsed, two related coding states that can be separated through the change in cellular dynamics with the animals’ speed. Other studies indicate that these cell sequences depend on theta oscillation from the medial septum and may reflect input from CA3 [2–4].

Running speed of the animal has also shown to influence theta oscillation frequency and amplitude. This oscillation could thereby carry the spatio-temporal information input required to determine distance/time coding. Inspired by [2], we modeled a circular recurrent network of excitatory cells with short-term synaptic plasticity [5] and global inhibition. By applying speed-dependent theta oscillation, we reproduced the dynamics of spatio-temporal coding observed in experimental data and propose a mechanism of switching between the two coding states through a change in integration of theta input. In particular, our firing rate model reproduces the sequence properties (recurrence, unidirectionality, sparse activity, memory) based on the network characteristics of CA3 and allows exploring the dynamics of the sequential activity. Simulations with this model show a non-trivial relationship between sequence slope and the frequency/amplitude of the oscillatory input: depending on the amplitude range of the theta oscillation, sequence dynamics can either be independent of speed (time coding) or linearly dependent on speed (distance coding). Therefore, the model proposes a network structure that could give rise to two basic and possibly default, self-referenced coding states observed in the hippocampus.

This model provides insights into how a recurrent network operates in the absence of spatially specific input, but still allows for such input to modulate sequential activity towards place field representation [2]. We will next explore further the mechanisms of sequence generation and coding correlates in both theoretical and experimental work.

**References**

1. Villete V, Malvache A, Tressard T, Dupuy N, Cossart R: Internally Recurring Hippocampal Sequences as a Population Template of Spatiotemporal Information. *Neuron* 2015, **88(2):**357–366.

2. Wang Y, Romani S, Lustig B, Leonardo A, Pastalkova E: Theta sequences are essential for internally generated hippocampal firing fields. *Nature Neuroscience* 2015 **18(2)**:282–290.

3. Salz DM., Tigany Z, Khasnabish S, Kohley A, Sheehan D, Howard MW, Eichenbaum H: Time Cells in Hippocampal Area CA3. *J. Neurosci.* 2016, **36:**7476–7484.

4. Guzman SJ, Schlögl A, Frotscher M, Jonas P: Synaptic mechanisms of pattern completion in the hippocampal CA3 network. *Science* 2016, **353**:1117–1123.

5. Mongillo G, Barak, O, Tsodyks M: Synaptic theory of working memory. *Science* 2008**, 319:**1543–1546.

## P160 The effect of progressive degradation of connectivity between brain areas on the brain network structure

### Kaoutar Skiker, Mounir Maouene

#### Department of mathematics and computer science, ENSAT, Abdelmalek Essaadi’s University, Tangier, Morocco

##### Correspondence: Kaoutar Skiker (skiker.kaoutar85@gmail.com)

*BMC Neuroscience* 2017, **18** (**Suppl 1**):P160

Neurodegenerative diseases such as Alzheimer and Schizophrenia are characterized by the progressive decline of cognitive functions such as memory, language and consciousness with take the form of memory loss, deficits in verbal and non-verbal communication and so on. Cognitive deficits are interpreted in terms of damage in the network of brain areas, instead of damage to specific brain areas [1]. Many studies combining network theory and neuroimaging data have shown that brain networks, known to have a small world structure [2], are disorganized in people with neurodegenerative diseases indicating that the connectivity between brain areas is altered by the disease [1]. The disorganization of brain networks can be a consequence of the vulnerability of hub areas to diseases or from the abnormal connectivity between brain areas.

In this paper, we assess how the progressive degradation of connectivity between brain areas affects the brain network structure. We propose an algorithm building on the idea that the connections between brain areas are weakened as the disease progress in time. We apply the algorithm on a functional connectivity matrix freely available for download from the Brain Connectivity Toolbox consisting of nodes representing brain areas and edges representing the functional links between two brain areas [3]. The network is weighted, with weights wij reflect the correlations between two brain areas Ai and Aj. At a given threshold t, the new weights are given by wij-t; with t indicates the progression of disease in time. The structure of the new network is analyzed using graph theoretical measures including clustering coefficient and path length. After damage, the functional brain network shows the properties of high clustering and low path length indicting that the network presents a small world structure necessary for the proper cognitive functioning. The progressive degradation of links doesn’t change the network’s properties dramatically, clustering coefficient are slightly modified until t = 0.25 (see Figure 1 for clustering coefficient). At this stage, the functional network shifts from high organization to randomness.

In sum, cognitive deficits in neurodegenerative diseases can be understood in the scope of the progressive degradation of the connectivity between brain areas within the network.
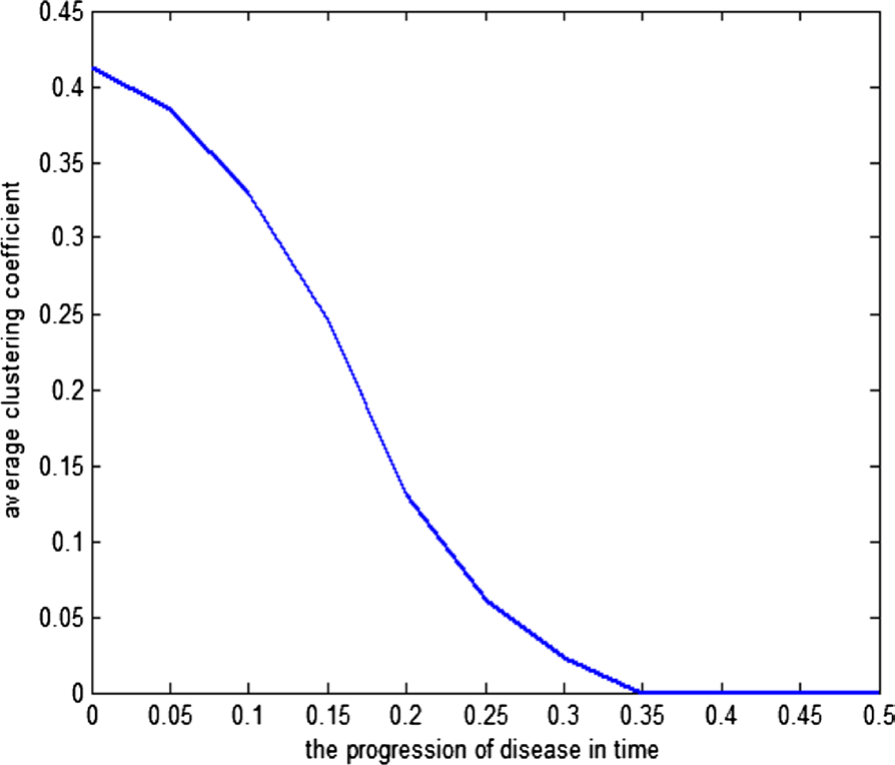


**Figure 1.** The average clustering coefficient of the network decreases following the progressive degradation of the connectivity between brain areas

**References**

1. DS Bassett, ET Bullmore: Human Brain Networks in Health and Disease. Current Opinion in Neurology 2009, **22**: 340–47.

2. O Sporns: Network Attributes for Segregation and Integration in the Human Brain. Current Opinion in Neurobiology 2013, **23**: 162–71.

3. M Rubinov, O Sporns: Complex network measures of brain connectivity: Uses and interpretations. Neuroimage 2010, **52**:1059–1069.

## P161 A network architecture for comparing the behavior of a neurocomputational model of reward-based learning with human

### Gianmarco Ragognetti^1^, Letizia Lorusso^2^, Andrea Viggiano^2^ and Angelo Marcelli^1^

#### ^1^Laboratory of Natural Computation, Department of Information and Electrical Engineering and Applied Mathematics, University of Salerno, 84084 Fisciano (SA), Italy; ^2^Department of Medicine, University of Salerno, 84083 Lancusi (SA), Italy

##### Correspondence: Gianmarco Ragognetti (gragognetti@unisa.it)

*BMC Neuroscience* 2017, **18** (**Suppl 1**):P161

Neuro computational models represent a powerful tool for bridging the gap between functions of the neural circuits and observable behaviors [1]. Once the model has been built, its output is compared with the observations either to validate the model itself or to propose new hypotheses. This approach has led to building a multi-scale model of the sensorimotor system from muscles, proprioceptors to skeletal joints, spinal regulating centers and central control circuits [2–6].

In this framework, we propose a neural network architecture to simulate the selection of actions performed by the motor cortex in response to a sensory input during a reward-based movement learning. The network has as many input nodes as the number of different stimuli, each node being a combination of the sensory inputs, and as many output nodes as the number of different actions that can be performed, each node being a combination of the motor commands. The network is fully connected, so that each stimulus concurs to the selection of each action and each action is selected concurrently by all the stimuli. The weights are updated by taking into account both the expected reward and the actual reward, as suggested in [7]. By adopting this architecture, the *percept* is represented by a combination of sensory inputs, while the *action* is represented by a combination of motor commands. Thus, it reproduces faithfully the condition of experiments of motor learning when a set of sensory inputs, such as semantically neutral visual stimuli, are presented to the subject whose response is merely a motor action, such as pushing a button. Under such conditions, it then becomes possible to fit the data provided by the experiments with the model to both estimate the validity of the model and to infer the role of the parameter on behavioral traits.

The simulations were compared to the behaviors of human subjects while learning which out of two buttons to press in response to a collection of visual stimuli containing edges and geometric shapes in a reward based setting. The results showed that the behavior of the complete system is the one expected under the hypothesis that the reward acts by modulating the action selection triggered by the input stimuli during motor learning. Moreover, differently from most literature models, the learning rate varies with the complexity of the task, i.e. the number of input stimuli. It can be argued that the decrease in learning rate seen in humans learning large set of stimuli could be due to an attenuation of memory traces in real synapses over time. In our future investigations, we will work to improve the model by adding such an effect in our network.

**References**

1. Lan, N., Cheung, V. and Gandevia, S.C.: EDITORIAL - Neural and Computational Modeling of Movement Control. *Front. in Comp. Neurosc*. 2016, **10**: 1–5.

2. Cheng, E. J., Brown, I.E., and Loeb, G. E.: Virtual muscle: a computational approach to understanding the effects of muscle properties on motor control. *J. Neurosci. Methods* 2000, **101**: 117–130.

3. Mileusnic, M. P., Brown, I.E., Lan, N., and Loeb, G. E.: Mathematical models of proprioceptors. I. Control and transduction in the muscle spindle. *J. Neurophysiol.* 2006, **96:** 1772–1788.

4. Song, D., Raphael, G., Lan, N., and Loeb, G. E.: Computationally efficient models of neuromuscular recruitment and mechanics. *J. Neural Eng.* 2008, **5**: 175–184.

5. Song, D., Lan, N., Loeb, G. E., and Gordon, J.: Model-based sensorimotor integration for multi-joint control, development of a virtual arm model. *Ann. Biomed. Eng.* 2008, **36**: 1033–1048.

6. He, X., Du, Y. F., and Lan, N.: Evaluation of feedforward and feedback contributions to hand stiffness and variability in multi joint arm control. *IEEE Trans. Neural Syst. Rehabil. Eng*. 2013, **21**: 634–647.

7. Sutton, R. S., and Barto A.G.: *Reinforcement learning: An introduction*. Cambridge: MIT press, 1998.

## P162 Distributed plasticity in the cerebellum: how do cerebellar cortex and nuclei plasticity cooperate for learning?

### Rosa Senatore, Antonio Parziale, Angelo Marcelli

#### Laboratory of Natural Computation, Department of Information and Electrical Engineering and Applied Mathematics, University of Salerno, 84084 Fisciano (SA), Italy

##### Correspondence: Rosa Senatore (rsenatore@unisa.it)

*BMC Neuroscience* 2017, **18** (**Suppl 1**):P162

Different forms of synaptic plasticity have been revealed within the cerebellum (CB), and many hypothesis about their role have been proposed [1]. We used a model-based analysis for investigating the role of these forms of plasticity in three behaviors: phase reversal of the vestibule-ocular reflex, acquisition of conditioned responses and learning a novel limb movement. We investigated these behaviors since they involve different forms of learning: phase reversal requires to modify a preexistent stimulus-response (S-R) association according to the feedback signal provided by climbing fibers (CFs); conditioning involves learning a new S-R association according to a preexistent one between the stimulus coming from the CFs and a motor response; learning novel motor behaviors corresponds to create new S-R associations according to the CF feedback. The analysis was carried through a CB model that incorporates plasticity mechanisms at different stages of the CB processing, both in cortex and nuclei [2]. Synaptic plasticity has been simulated in both granular (Gr) and Purkinje (PC) network: granule cells show intrinsic plasticity depending on mossy fibers (MFs) activity, and MF-Gr synapses undergo both Long Term Depression (LTD) and Long Term Potentiation (LTP)[3]; PF-PC synapses undergo both LTD and LTP, depending on PF and CF activity [4]. The model also includes synaptic plasticity involving the molecular interneurons (MLI) at PF-MLI synapses [5] and Rebound potentiation at MLI-PC synapses [6]. Within the CB nuclei, LTD occurs in MF-NC synapses during inhibition from PCs, whereas LTP occurs during release from inhibition [7]. Our results suggest that the main contribution to CB learning is provided by the synaptic plasticity at PF-PC and MF-NC synapses. Indeed, excluding the plasticity at PF–PC site caused strong impairment in learning all the considered behaviors, while excluding the plasticity at MF–NC site induced mild impairment in acquiring conditioned responses and novel limb movements, and strong impairment was observed in phase reversal and motor adaptation. Removal of other forms of synaptic plasticity only induced slower learning. Our results also suggest that LTP at PF-PC underlies the extinction phenomenon observed in conditioning, and that saving phenomenon could be ascribed to a residual plasticity within the CB cortex rather than within the CB nucleus, since saving was observed even after removal of MF-NC plasticity before reconditioning. Finally, model simulations support the view that learned associations are transferred from the CB cortex to the CB nuclei, due to the combined effect of plasticity at PF-PC synapses in early stage of learning, and MF-NC synapses in late learning. Indeed, lesions at PCs layer or removal of PF-PC synaptic plasticity in late learning stage did not induced any impairment in the behavior of the model, whereas removal of PF-PC synaptic plasticity in early learning impaired learning capabilities of the model.

**References**

1. Gao Z, van Beugen BJ, De Zeeuw CI: Distributed synergistic plasticity and cerebellar learning. *Nat Rev Neurosci* 2012, **13**:619–635.

2. Senatore R, Parziale A, Marcelli A: A computational model for investigating the role of cerebellum in acquisition and retention of motor behavior. *25th Annual Computational Neuroscience Meeting: CNS*-*2016*. *BCM Neurosci* 2016, **17**: 64–64.

3. Gall D, Prestori F, Sola E, D’Errico A, Roussel C, Forti L, Rossi P, D’Angelo E: Intracellular calcium regulation by burst discharge determines bidirectional long-term synaptic plasticity at the cerebellum input stage. *J Neurosci* 2005, **25**:4813–4822.

4. Coesmans M, Weber JT, De Zeeuw CI, Hansel C: Bidirectional parallel fiber plasticity in the cerebellum under climbing fiber control. *Neuron* 2004, **44**:691–700.

5. Rancillac A, Crépel F: Synapses between parallel fibres and stellate cells express long-term changes in synaptic efficacy in rat cerebellum. *J Physiol* 2004, **554**:707–720.

6. Kano M, Rexhausen U, Dreessen J, Konnerth A: Synaptic excitation produces a long-lasting rebound potentiation of inhibitory synaptic signals in cerebellar Purkinje cells. *Nature* 1992, **356**:601–604.

7. Aizenman CD, Linden DJ: Rapid, synaptically driven increases in the intrinsic excitability of cerebellar deep nuclear neurons. *Nat Neurosci* 2000, **3**:109–111.

## P163 Ising Model with conserved magnetization on the Human Connectome: implications on the relation structure-function in wakefulness and anesthesia

### S. Stramaglia^1^, M. Pellicoro^1^, L. Angelini^1^, E. Amico^2,3^, H. Aerts^2^, J. Cortés^4^, S. Laureys^3^, D. Marinazzo^2^

#### ^1^Dipartimento di Fisica, Università degli Studi Aldo Moro, Bari, and INFN, Sezione di Bari, Italy; ^2^Data Analysis Department, Ghent University, Ghent, Belgium; ^3^Coma Science Group, University of Liège, Liège, Belgium; ^4^Cruces Hospital and Ikerbasque Research Center, Bilbao, Spain

##### Correspondence: S. Stramaglia (sebastiano.stramaglia@ba.infn.it)

*BMC Neuroscience* 2017, **18** (**Suppl 1**):P163

Dynamical models implemented on the large-scale architecture of the human brain may shed light on how function arises from the underlying structure. This is the case notably for simple abstract models, such as the Ising one. We compare the spin correlations of the Ising model and the empirical functional brain correlations, both at the single link level and at the modular level, and show that the prediction is better in anesthesia than in wakefulness, in agreement with recent experiments. We show that conserving the magnetization in the Ising model dynamics (Kawasaki dynamics) leads to an improved prediction of the empirical correlations in anesthetised brains, see Figure 1. Moreover, we show that at the peak of the specific heat (the *critical state*) the spin correlations are minimally shaped by the underlying structural network, explaining how the best match between structure and function is obtained at the onset of criticality, as previously observed.

These findings could open the way to novel perspectives when the conserved magnetization is interpreted in terms of a homeostatic principle imposed to neural activity.
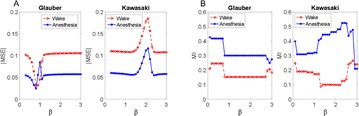


**Figure 1. A.** Mean Squared Error in Wakefulness and Anesthesia between the empirical connectivity and the one simulated by Glauber and Kawasaki dynamics. **B.** Mutual Information between the modular partitions of the empirical and modelled functional networks. These quantities are depicted as a function of the inverse temperature β

**Conclusions:** In agreement with recent theoretical frameworks [1], our results suggest that a wide range of temperatures correspond to *criticality* of the dynamical Ising system on the connectome, rather than a narrow interval centered in a critical state. In such conditions, the correlational pattern is minimally shaped by the underlying structural network. It follows that, assuming that the human brain operates close to a *critical* regime [2], there is an intrinsic limitation in the relationship between structure and function that can be observed in data. We show that empirical correlations among brain areas are better reproduced at the modular level using a model which conserves the global magnetization. The most suitable way to compare functional and structural patterns is to contrast them at the network level, using, e.g., the mutual information between partitions like in the present work.

**References**

1. Moretti P. and Muñoz M.A.: Griffiths phases and the stretching of criticality in brain networks, *Nature communications* 2013, **4:** 2521.

2. Chialvo D.: Emergent complex neural dynamics, *Nature Physics* 2010, **6:** 744–750.

## P164 Multiscale Granger causality analysis by *à trous* wavelet transform

### S. Stramaglia^1^, I. Bassez^2^, L. Faes^3^, D. Marinazzo^2^

#### ^1^Dipartimento di Fisica, Università degli Studi Aldo Moro, Bari, and INFN, Sezione di Bari, Italy; ^2^Data Analysis Department, Ghent University, Ghent, Belgium; ^3^BIOtech, Dept. of Industrial Engineering, University of Trento, and IRCS-PAT FBK, Trento, Italy

##### Correspondence: S. Stramaglia (sebastiano.stramaglia@ba.infn.it)

*BMC Neuroscience* 2017, **18** (**Suppl 1**):P164

Great attention has been devoted in the last years to the identification of information flows in human brains. Since interactions occur across multiple temporal scales, it is likely that information flow will exhibit a multiscale structure: high-frequency activity, reflecting local domains of cortical processing, and low-frequency activity dynamically spread across the brain regions by both external sensory input and internal cognitive events. In order to detect information flow at multiple scale the decomposition of the signals in the wavelet space has been proposed in [1]; an analytical frame for linear multivariate stochastic processes explored at different time scales has been proposed in [2]. However, the computation of multiscale measures of information dynamics may be complicated by theoretical and practical issues such as filtering and undersampling: to overcome this problems, we propose here another wavelet-based approach for multiscale causality analysis, which is characterized by the following properties: (i) only the candidate driver variable is wavelet transformed (ii) the decomposition is performed using the *à trous* wavelet transform with cubic B-spline filter [3]. The use of the *à trous* transform is suggested by its interesting properties, indeed it satisfies the shift invariance, and its coefficients at time t are a linear combination of the time series values; no decimation of the time series, as in the discrete wavelet transform, is done. Granger causality examines how much the predictability of the target from its past improves when the driver variables’ past values are included in the regression, where m is the order of the model. We propose here to measure the causality at scale s by including w(t-1,s), w(t-2,s),…,w(t-m,s) in the regression model of the target, where w(t,s) are the *à trous* wavelet coefficients of the driver. In figure 1 we depict the multiscale causality evaluated by the proposed approach on a simulated two-dimensional linear system unidirectionally coupled with lag equal to 8 and strength a: it increases with the strength and peaks in correspondence of the lag. We have applied the proposed algorithm to scalp EEG signals [4], and we found that the global amount of causality among signals is significantly decreasing as the scale s is increased. Furthermore, comparing signals corresponding to resting conditions with closed eyes and with open eyes, we found that at large scales the effective connectivity, in terms of the proposed measure, is significantly lower with eyes open.
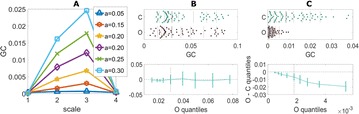


**Figure 1. A.** Granger causality in an unidirectionally coupled system is depicted as a function of the scale for several values of the coupling. **B.** GC values for eyes open and closed conditions from regular time series. **C.** GC values in the same conditions from wavelet coefficients (scale 4)

**References**

1. Lungarella M, Pitti A, Kuniyoshi K: Information transfer at multiple scales. *Phys. Rev. E* 2007, **76**: 056117

2. Faes, L., Montalto, A., Stramaglia, S., Nollo, G., Marinazzo, D.: Multiscale analysis of information dynamics for linear multivariate processes, *Proceedings of the Annual International Conference of the IEEE Engineering in Medicine and Biology Society, EMBS* 2016.

3. Renaud O, Starck, J-L, Murtagh, F: Wavelet-Based Combined Signal Filtering and Prediction. *IEEE Transactions on Systems, Man, and Cybernetics, Part B: Cybernetics.* 2005, vol. 35, no. 6, p. 1241–1251

4. http://www.physionet.org/pn4/eegmmidb

## P165 New (spectral) dynamic causal modeling scheme improves effective connectivity estimation within resting state networks in longitudinal data

### Hannes Almgren^1^, Frederik Van De Steen^1^, Adeel Razi^2,3^, Daniele Marinazzo^1^

#### ^1^Department of Data Analysis, Ghent University, Ghent, 9000, Belgium; ^2^The Wellcome Trust Centre for Neuroimaging, University College London, London, WC1 N 3BG, UK; ^3^Department of Electronic Engineering, NED University of Engineering and Technology, Karachi, Pakistan

##### Correspondence: Hannes Almgren (Hannes.Almgren@ugent.be)

*BMC Neuroscience* 2017, **18** (**Suppl 1**):P165

Effective connectivity within resting state networks has been estimated using spectral dynamic causal modeling (spDCM) [1]. Since its initial release, spDCM has been updated to improve performance and to render it applicable to larger networks. The objective of the present study is to assess the impact of these changes on parameter estimates and stability. We therefore compared performance between an early version of DCM (v6303) and a newer version of DCM (v6801) in combination with the parametric empirical Bayesian (PEB) framework [2]. Both were compared regarding (1) ability to explain observed cross spectral densities (CSD), (2) estimated network structure, and (3) stability of parameter estimates. An extensive single-subject longitudinal dataset, including 101 resting state fMRI sessions, was analyzed (myconnectome.org/wp) [3]. Eight resting state sessions were chosen for our analyses: occipital and lateral visual, auditory, somatomotor, left and right frontoparietal, default mode, and executive control network. Results showed that the newer spDCM-PEB combination explained the data (i.e., CSDs) far better than the older spDCM (95.31% versus 68.31% explained variance, respectively). Furthermore, the older version often failed to yield proper estimates (i.e., because of low proportion explained variance or estimated connection strengths near zero) in networks consisting of two- or three regions, while the newer version showed less such problems. Concerning average network structure across sessions, the newer spDCM-PEB combination detected asymmetric influences within networks consisting of two regions (see Figure 1). Furthermore, regions located in the medial part of the brain showed larger in- versus out-connectivity. For the default mode network, consisting of four regions in the present study, both versions yielded largely similar network structures (i.e., reciprocal influences between bilateral parietal cortices, and larger in- versus out-connectivity for medial areas). However, the older version of spDCM showed a positive influence (0.21 Hz) from precuneus to medial prefrontal cortex, which was much smaller (0.05 Hz) for the newer DCM-PEB combination. Stability depended profoundly on the size of the network: parameter estimates showed higher stability in two-region networks than in larger networks for both versions.
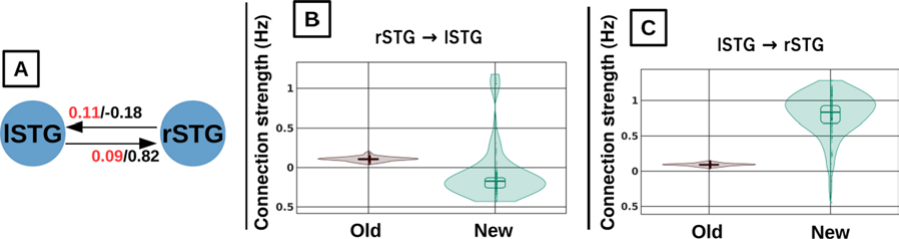


**Figure 1.** Comparison of posterior parameter estimates within the auditory network. **A.** median posterior parameter estimates for the older version (shown in red) and the newer spDCM-PEB combination (shown in black). **B and C.** distribution of these parameter estimates over sessions, together with the bootstrapped high density intervals, for both the older and newer scheme

**References**

1. Friston KJ, Kahan J, Biswal B, Razi, A: A DCM for resting state fMRI. *NeuroImage* 2014, **94:**396–407.

2. Friston KJ, Litvak V, Oswal A, Razi A, Stephan KE, van Wijk BC, Ziegler G, Zeidman P: Bayesian model reduction and empirical Bayes for group (DCM) studies. *NeuroImage* 2016, **128:**413–431.

3. Laumann TO, Gordon EM, Adeyemo B, Snyder AZ, Joo SJ, Chen MY, Gilmore AW, McDermott KB, Nelson SM, Dosenbach NU, et al.: Functional system and areal organization of a highly sampled individual human brain. *Neuron* 2015, **87(3):**657–670.

## P166 Effective connectivity modulations of win-and loss feedback: A dynamic causal modeling study of the human connectome gambling task

### Frederik Van de Steen^1^, Ruth Krebs^2^, Daniele Marinazzo^1^

#### ^1^Department of data analysis, Ghent University, Ghent, 9000, Belgium; ^2^Department of experimental psychology, Ghent University, Ghent, 9000, Belgium

##### Correspondence: Frederik Van de Steen (frederik.vandesteen@ugent.be)

*BMC Neuroscience* 2017, **18** (**Suppl 1**):P166

The main goal of this study was to investigate changes in effective connectivity associated with reward and punishment. More specifically, changes in connectivity between the ventral striatum (VS), anterior insula (aI), anterior cingulate cortex (ACC) and occipital cortex (OCC) that are related to win- and loss- feedback were studied.

Here, fMRI data from the human connectome project [1] was used for our study purposes. Data from 369 unrelated subjects performing a gambling task was analyzed. In short, participants played a card game where they had to guess whether the upcoming card would be higher or less than 5 (range was between 1 and 9). After the gamble, feedback was provided indicating a reward, punishment or neutral trial. The minimally preprocessed data was used and extra spatially smoothed with a 5-mm FWHM Gaussian kernel. The images were then entered in a first level general linear model (GLM) and summary statistic images of the first level GLM were entered in a second level GLM. The following two contrasts were used to identify the relevant brain regions at the group level: [Win - Neut] AND [Loss-Neut] (i.e. conjunction), and [Win-neut]. Based on the group level results, time-series of VS, aI, ACC and OCC were extracted for every subject and used in further dynamic causal modeling (DCM, [2]) analysis. We specified a fully connected model (i.e. all nodes are reciprocally connected) where the win and loss events were allowed to modulate all connections. The driving input consisted of all feedback events (win, loss and neutral events) and entered the DCM’s via OCC. The fully connected model was estimated for every subject and then used in the recently proposed parametric empirical Bayesian (PEB, [3]) framework for estimating DCM parameters at the group level. Finally, we used Bayesian model reduction to obtain the best 255 nested models. Since there was no clear winning model, Bayesian model averaging (BMA) of the 256 model (full + 255 nested models) parameters was performed. Figure 1. shows the group level BMA modulatory parameters with a posterior probability >.95.

**Conclusion:** Overall, both win- and loss- feedback have a general increasing effect on effective connectivity. The main difference between win and loss can be observed for the connection from aI and OCC with loss-feedback having a decreased effect. In addition, only win-feedback increases the connection from VS to aI. Overall, the VS appears as a key region in conveying loss and win information across the network.
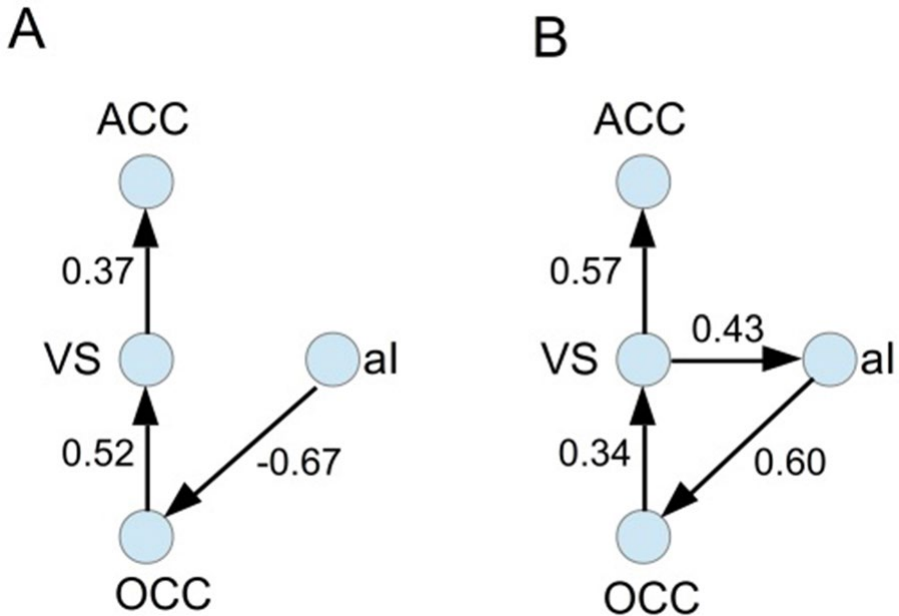


**Figure 1.** BMA modulatory parameters at the group level are shown for **A.** loss feedback; **B.** win feedback

**Acknowledgements**

This research was supported by the Fund for Scientific Research-Flanders (FWO-V), Grant FWO16/ASP_H/255.

**References**

1. Van Essen, D. et al. The WU-Minn Human Connectome Project: An overview. NeuroImage, 2013, **80:** 62–79.

2. Friston, Karl J., Lee Harrison, and Will Penny. Dynamic causal modelling. *Neuroimage*, 2003, **19(4):** 1273–1302.

3. Friston, Karl J., et al. Bayesian model reduction and empirical Bayes for group (DCM) studies. *Neuroimage*

## P167 Modeling global brain dynamics in brain tumor patients using the Virtual Brain

### Hannelore Aerts, Daniele Marinazzo

#### Department of Data Analysis, Ghent University, Ghent, Belgium

Correspondence: Hannelore Aerts (hannelore.aerts@ugent.be)

*BMC Neuroscience* 2017, **18** (**Suppl 1**):P167

Increasingly, computational models of brain activity are applied to investigate the relation between structure and function. In addition, biologically interpretable dynamical models may be used as unique predictive tools to investigate the impact of structural connectivity damage on brain dynamics. That is, individually modeled biophysical parameters could inform on alterations in patients’ local and large-scale brain dynamics, which are invisible to brain-imaging devices. In this study, we compared global biophysical model parameters between brain tumor patients and healthy controls. To this end, we used The Virtual Brain (TVB; [1]), a neuroinformatics platform that utilizes empirical structural connectivity data to create dynamic models of an individual’s brain.

Ten glioma patients (WHO grade II and III, mean age 41.1yo, 4 females; 5 from open access dataset [2]), 13 meningioma patients (mean age 60.23y, 11 females), three pseudo-meningioma patients (subtentorial brain tumors, mean age 58yo, 2 females) and 11 healthy partners (mean age 58.6y, 4 females) were included in this study. From all participants, diffusion MRI, resting-state fMRI and T1-weighted MRI data were acquired. Data were preprocessed and converted to a subject-specific structural and functional connectivity matrix using a modified version of the TVB preprocessing pipeline [3].

In order to simulate brain dynamics, the reduced Wong-Wang model [4] was used. This is a dynamical mean field model that consistently summarizes the realistic dynamics of a detailed spiking and conductance-based synaptic large-scale network. A subject-specific parameter space exploration was conducted to obtain an optimal correspondence between the individual’s simulated and empirical functional connectivity matrix. To this end, values of the global scaling factor *G* and the local feedback inhibitory synaptic coupling *J*_*i*_ were varied. Values of *G* and *J*_*i*_ yielding optimal correspondence were then compared between the brain tumor patient groups and healthy controls.

The distribution of optimal values for *G* and *J*_*i*_ per group is depicted in Figure 1. Visually, no clear group differences are apparent. In future studies, larger sample sizes will be utilized, as data collection is still ongoing and more efforts to data sharing across labs are undertaken. In addition, local model parameter alterations in the vicinity of the lesion will be examined, since global model parameters might not be sufficiently sensitive to capture local lesion effects.
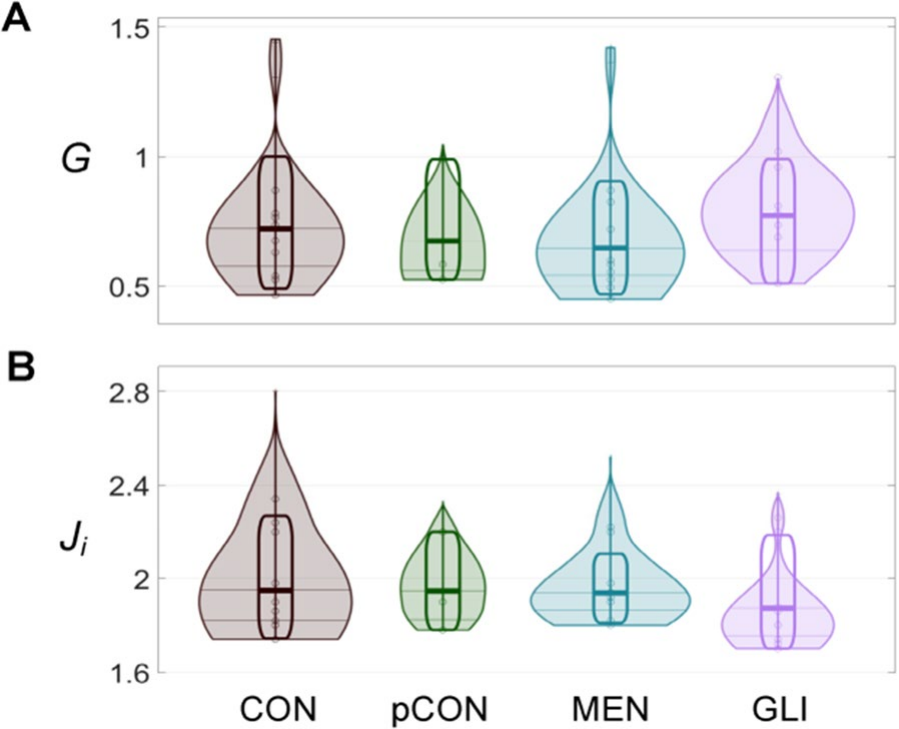


**Figure 1.** Distribution of optimal model parameter values per group: control subjects (CON), pseudo control subjects with subtentorial brain tumor (pCON), meningioma patients (MEN), and glioma WHO grade II and III patients (GLI). **A.** Global scaling factor (*G*); **B.** Local feedback inhibitory synaptic coupling (*J*_*i*_)

**References**

1. P Sanz Leon, S A Knock, M M Woodman, L Domide, J Mersmann, A R McIntosh, V K Jirsa. The Virtual Brain: A simulator of primate brain network dynamics. *Frontiers in Neuroinformatics* 2013, **7**:1–23.

2. C Pernet, K Gorgolewski, I Whittle. UK Data Archive. [http://dx.doi.org/10.5255/UKDA-SN-851861]

3. M Schirner, S Rothmeier, V K Jirsa, A R McIntosh, P Ritter. An automated pipeline for constructing personalized virtual brains from multimodal neuroimaging data. *NeuroImage* 2015, **117:**343–357.

4. G Deco, A Ponce-Alvarez, P Hagmann, G L Romani, D Martini, M Corbetta. How local excitation-inhibition ratio impacts the whole brain dynamics. *The Journal of Neuroscience* 2014, **34:**7886–7898.

## P168 Representation of Neuronal Morphologies

### Lida Kanari^1^, Pawel Dlotko^2^, Martina Scolamiero^3^, Ran Levi^4^, Julian Shillcock^1^, Christiaan P.J. de Kock^5^, Kathryn Hess^3^ and Henry Markram^1^

#### ^1^Blue Brain Project, École polytechnique fédérale de Lausanne, Lausanne, Switzerland; ^2^Departement of Mathematics, Swansea University, Swansea, Wales, UK; ^3^Laboratory for Topology and Neuroscience at the Brain Mind Institute, École polytechnique fédérale de Lausanne, Lausanne, Switzerland; ^4^Institute of Mathematics, University of Aberdeen, Aberdeen, Scotland, UK; ^5^Department of Integrative Neurophysiology, Center for Neurogenomics and Cognitive Research, VU Universiteit Amsterdam, Amsterdam, the Netherlands

##### Correspondence: Lida Kanari (lida.kanari@epfl.ch)

*BMC Neuroscience* 2017, **18** (**Suppl 1**):P168

The shape of neuronal arborizations defines amongst other aspects their physical connectivity and functionality. Yet an efficient method for quantitatively analyzing the spatial structure of such trees has been difficult to establish. The wide diversity of neuronal morphologies in the brain, even for cells identified by experts as of the same type, renders an objective classification scheme a challenging task.

We propose a Topological Morphology Descriptor [1], inspired by Topological Data Analysis, to quantitatively analyze the branching shapes of neurons, which overcomes the limitations of existing techniques. The TMD algorithm maps the branches of a tree (Fig 1A) into a “barcode” (Fig 1B). The TMD encodes the morphology of the tree into a simplified topological representation that preserves sufficient information to be useful for the comparison and the distinction of different branching patterns.
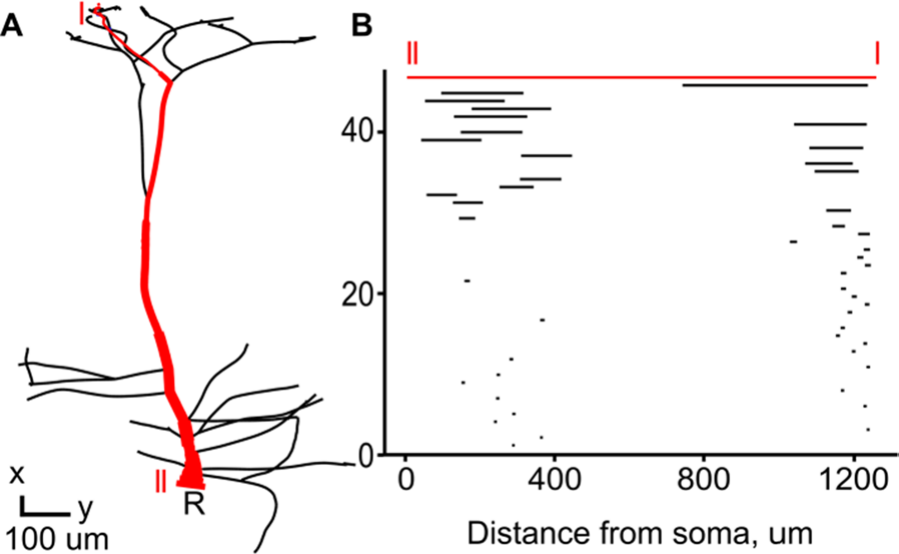


**Figure 1.** Topological morphology descriptor. **A.** The neuronal tree is mapped into a barcode. **B.** Each bar represents the lifetime of a branch; its start and end distance from the soma

This method is applicable to any tree-like structure, and we demonstrate its generality by applying it to groups of mathematical random trees and neuronal morphologies. We identify the structural differences between known morphological types [2-3] as well as subtypes for human temporal cortex L2/3 pyramidal cells [4]. Our results show that the TMD of tree shapes reliably and efficiently distinguishes different shapes of trees and neurons. Therefore, the TMD provides an objective benchmark test of the quality of any grouping of branching trees into discrete morphological classes. Our results demonstrate that the TMD can enhance our understanding of the anatomy of neuronal morphologies.

**References**

1. Kanari L, Dłotko P, Scolamiero M, Levi R, Shillcock J, Hess K, Markram H, Quantifying topological invariants of neuronal morphologies, arxiv.org 2016, [https://arxiv.org/abs/1603.08432]

2. Ascoli G.A., Donohue D.E. and Halavi M., NeuroMorpho.Org: A Central Resource for Neuronal Morphologies, *J. Neurosc.* 2007, **27 (35):** 9247–9251.

3. Markram H. Muller E., Ramaswamy S., Reimann M.W. et al., Reconstruction and Simulation of Neocortical Microcircuitry, *Cell* 2015, **163 (2):** 456–492.

4. Mohan H., de Kock C.P.J., et al. Dendritic and Axonal Architecture of Individual Pyramidal Neurons across Layers of Adult Human Neocortex, *Cereb Cortex* 2015, **25 (12):** 4839–4853.

## P169 Firing Rate Heterogeneity and Consequences for Stimulus Estimation in the Electrosensory System

### Cheng Ly^1^, Gary Marsat^2^

#### ^1^Department of Statistical Sciences and Operations Research, Virginia Commonwealth University, Richmond, VA 23284, USA; ^2^Biology Department, West Virginia University, Morgantown, WV 26506, USA

##### Correspondence: Cheng Ly (CLy@vcu.edu)

*BMC Neuroscience* 2017, **18** (**Suppl 1**):P169

Heterogeneity of neural attributes is recognized as a crucial feature in neural processing. Thus, we have developed theoretical methods (based on [1]) to characterize the firing rate distribution of spiking neural networks with intrinsic and network heterogeneity [2], both of which have been widely reported in experiments. This relationship (intrinsic and network) can lead to various levels of firing rate heterogeneity, depending on regime.

Next we adapt our theory to a delayed feedforward spiking network model of the electrosensory system of the weakly electric fish. Experimental recordings indicate that feedforward network input can mediate response heterogeneity of pyramidal cells [3]. We demonstrate that structured connectivity rules, derived from our theory, can lead to qualitatively similar statistics as the experimental data. Thus, the model demonstrates that intrinsic and network attributes do not interact in a linear manner but rather in a complex stimulus-dependent fashion to increase or decrease neural heterogeneity and thus shape population codes.

As evidence for heterogeneity shaping population codes, we also present some preliminary work using recordings from electric fish subject to noisy stimuli. We use a GLM model for each neuron, fit the parameters to the data using standard maximum likelihood methods, and perform Bayesian estimation of the stimuli. We find that firing rate heterogeneity is a signature of optimal (Bayesian) stimulus estimation of noisy stimuli. Interestingly, the firing rate correlation is not an indicator of decoding performance for a given population of neurons.

**References**

1. W. Nicola, C. Ly, S.A. Campbell: One-Dimensional Population Density Approaches to Recurrently Coupled Networks of Neurons with Noise. *SIAM Journal on Applied Mathematics* 2015, **75:**2333–2360.

2. C. Ly: Firing Rate Dynamics in Recurrent Spiking Neural Networks with Intrinsic and Network Heterogeneity. *Journal of Computational Neuroscience* 2015, **39:**311–327.

3. G. Marsat, G.J. Hupe, K.M. Allen: Heterogeneous response properties in a population of sensory neurons are structured to efficiently code naturalistic stimuli. Program # 181.20 *Neuroscience Meeting Planner* 2014.

## P170 Knowledge Space: a community encyclopedia linking brain research concepts to data, models and literature

### Tom Gillespie^3^, Willy Wong^3^, Malin Sandström^1^, Mathew Abrams^1^, Jeffrey S. Grethe^3^, Maryann Martone^4^

#### ^1^INCF Secretariat, Karolinska Institute, Nobels väg 15A, 17177 Stockholm, Sweden; ^2^Campus Biotech, EPFL, CH-1202 Genève, Switzerland; ^3^Center for Research in Biological Systems, UCSD, La Jolla 92093, CA, USA; ^4^Neurosciences, UCSD, La Jolla 92093, CA, USA

##### Correspondence: Malin Sandström (malin.sandstrom@incf.org)

*BMC Neuroscience* 2017, **18** (**Suppl 1**):P170

KnowledgeSpace [1] is a community encyclopedia platform currently under development where neuroscience data and knowledge are synthesized. KnowledgeSpace aims to provide a global interface between current brain research concepts and the data, models and literature about them. It is an open project that welcomes participation and contributions from members of the global research community.

KnowledgeSpace version 1.0 was launched at Neuroscience 2016 in San Diego, November 12-16, with three modes of search - keyword, category and atlas-based (so far only for mouse brain). During the pre-launch phase, work focused on linking concepts to data, models, and literature from existing community resources. Current data sources include NeuroLex, Allen Institute for Brain Sciences, The Blue Brain Project, NeuroMorpho, NeuroElectro, Cell Image Library, NIF Integrated Connectivity, Ion Channel Genealogy, ModelDB, Open Source Brain, GenSat, BrainMaps, NeuronDB, The Human Brain Atlas, and PubMed. Initial content included in KnowledgeSpace covers ion channels, neuron types, and microcircuitry. For each content type, physiology, gene expression, anatomy, models, and morphology data sources are available.

Going forward we will enhance atlas representations of the mouse brain linking concepts to data, models, and literature, and an atlas representation of the human brain that links to available data, models, and literature will be implemented. Links to analysis tools will also be integrated into the KnowledgeSpace data section. The project will also develop protocols, standards, and mechanisms that allow the community to add data, analysis tools, and model content to KnowledgeSpace.

The initial development of KnowledgeSpace has been driven and supported by the International Neuroinformatics Coordinating Facility (INCF; incf.org), the Neuroscience Information Framework (NIF; neuinfo.org) and the Blue Brain Project (BBP; bluebrain.epfl.ch). The KnowledgeSpace also represents an important component of the Neuroinformatics Platform being deployed in the Human Brain Project web portal. KnowledgeSpace is currently transitioning to a shared governance model, with a Governing Board composed of members of the neuroscience community who are currently funded to generate or share data and/or code as part of a lab, project or organization, and who will rotate off the board when their project ends.

**Reference**

1. KnowledgeSpace website [https://knowledge-space.org/index.html]

## P171 Evaluating the computational capacity of a cerebellum model

### Robin De Gernier^1^, Sergio Solinas^2^, Christian Rössert^3^, Marc Haelterman^1^, Serge Massar^1^

#### ^1^École polytechnique de Bruxelles, Université libre de Bruxelles, Brussels, Belgium, 1050; ^2^Department of Biomedical Science, University of Sassari, Sassari, Italia, 07100; ^3^Blue Brain Project, École polytechnique fédérale de Lausanne, Geneva, CH-1202, Switzerland

##### Correspondence: Robin De Gernier (rdegerni@ulb.ac.be)

*BMC Neuroscience* 2017, **18** (**Suppl 1**):P171

The cerebellum plays an essential role in tasks ranging from motor control to higher cognitive functions (such as language processing) and receives input from many brain areas. A general framework for understanding cerebellar function is to view it as an adaptive-filter [1]. Within this framework, understanding, from computational and experimental studies, how the cerebellum processes information and what kind of computations it performs is a complex task, yet to be fully accomplished. In the case of computational studies, this reflects a need for new systematic methods to characterize the computational capacities of cerebellum models. In the present work, to fulfill this need, we apply a method borrowed from the field of machine learning to evaluate the computational capacity of a prototypical model of the cerebellum cortical network. Using this method, we find that the model can perform both linear operations on input signals –which is expected from previous work-, and –more surprisingly- highly nonlinear operations on input signals.

The model that we study is a simple rate model of the cerebellar granular layer in which granule cells inhibit each other via a single-exponential synaptic connection. The resulting recurrent inhibition is an abstraction of the inhibitory feedback circuit composed of granule and Golgi cells. Purkinje cells are modelled as linear trainable readout neurons. The model was originally introduced in [2, 3] to demonstrate that models of the cerebellum that include recurrence in the granular layer are suited for timing-related tasks. Further studies carried out in [4] showed how the recurrent dynamics of the network can provide the basis for constructing temporal filters.

The method, described in detail in [5], and developed in the context of the artificial intelligence algorithm known as reservoir computing [6], consists in feeding the network model with a random time dependent input signal and then quantifying how well a complete set of functions (each function representing a different type of computation) of the input signal can be reconstructed by taking a linear combination of the neuronal activations. The result is a quantitative estimate of the number of different computations that can be carried out by the model. We conducted simulations with 1000 granule cells. Our results show that the cerebellum prototypical model has the capability to compute both linear and highly nonlinear functions of its input. Specifically, the model is able to reconstruct Legendre polynomial functions up to the 10th degree. Moreover, the model can internally maintain a delayed representation of the input with delays of up to 100 ms, and perform operations on that delayed representation. Despite their abstract nature, these two properties are essential to perform typical cerebellar functions, such as learning the timing of conditioned reflexes or fine-tuning nonlinear motor control tasks or, we believe, even higher cognitive functions.

In future work, we hope to confirm these abstract results by applying our cerebellum model to typical cerebellar tasks. Additionally, we will compare our results with a very recent work which studied how a model of the cerebellum could solve several machine learning tasks [7].

**References**

1. Dean P, Porril J: The cerebellar microcircuit as an adaptive filter: experimental and computational evidence. *Nat Rev Neurosci* 2010, **11(1):** 30–43.

2. Yamazaki T, Tanaka S: Neural Modeling of an Internal Clock. *Neural Comput* 2005, **17(5):** 1032–1058.

3. Yamazaki T, Tanaka S: The cerebellum as a liquid state machine. *Neural Netw* 2007, **20(3):** 290–297.

4. Rössert C, Dean P, Porrill J: At the Edge of Chaos: How Cerebellar Granular Layer Network Dynamics Can Provide the Basis for Temporal Filters. *PLOS Comput Biol* 2015, **11(10):**e1004515.

5. Dambre J, Verstraeten D, Schrauwen B, Massar S: Information processing capacity of dynamical systems. *Sci Rep* 2012, **2:**514.

6. Lukoševičius M, Jaeger H: Reservoir computing approaches to recurrent neural network training. *Computer Science Review* 2009, **3:**127–149.

7. Hausknecht M, Li WK, Mauk M, Stone P: Machine Learning Capabilities of a Simulated Cerebellum. *IEEE Trans Neural Netw Learn Syst* 2017, **28(3):**510–522.

## P172 Complexity of cortical connectivity promotes self-organized criticality

### Valentina Pasquale^1^, Vito Paolo Pastore^2^, Sergio Martinoia^2^, Paolo Massobrio^2^

#### ^1^Neuroscience and Brain Technologies Department, Istituto Italiano di Tecnologia (IIT), Genova, Italy; ^2^Department of Informatics, Bioengineering, Robotics, System Engineering (DIBRIS), University of Genova, Genova, Italy

##### Correspondence: Valentina Pasquale (valentina.pasquale@iit.it)

*BMC Neuroscience* 2017, **18** (**Suppl 1**):P172

Large-scale in vitro cortical networks spontaneously exhibit recurrent events of propagating spiking and bursting activity, usually termed as *neuronal avalanches*, since their size (and lifetime) distribution can be approximated by a power law, as in critical sand pile models [1, 2] (Figure 1). However, neuronal avalanches in cultures of dissociated cortical neurons can distribute according to three different dynamic states, namely sub-critical, critical, or super-critical, depending on several factors like developmental stage, excitation/inhibition balance, cell density, etc. [3]. In this work, we investigated the role of connectivity in driving spontaneous activity towards critical, sub-critical or super-critical regimes, by combining both experimental and computational investigations.

Our experimental model consists of mature networks (third week of in vitro development) of cortical dissociated neurons coupled to High-Density Micro-Electrode Arrays (HD-MEAs) (3Brain, Wadenswill, Switzerland). These devices, containing 4’096 microelectrodes, 81 µm-spaced, allow to follow the emergence and propagation of neuronal avalanches with high spatio-temporal resolution. We estimated the functional connectivity of cortical networks by using cross-correlation based methods, collected in the software ToolConnect [4]. In particular, our cross-correlation algorithm is able to reliably and accurately infer functional and effective excitatory and inhibitory links in ex vivo neuronal networks, while guaranteeing high computational performances necessary to process large-scale population recordings. To support our experimental investigations, we also developed a computational model of neuronal network, made up of Izhikevich neurons [5] structurally connected by following well defined topologies of connectivity (e.g., random, scale-free, small-world).

Simulations of the model demonstrated that the presence of hubs, the physiological balance between excitation and inhibition, and the concurrent presence of scale-free and small-world features are necessary to induce critical dynamics. We then confirmed the predictions of the model by analyzing medium/high density cortical cultures coupled to HD-MEAs, finding that networks featuring both scale-free and small-world properties (as computed from functional connectivity graphs) display critical behavior.
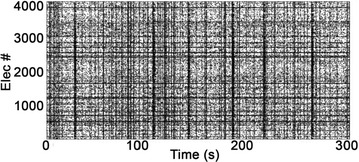


**Figure 1.** Example of electrophysiological activity of a cortical network coupled to a High-Density Micro-Electrode Arrays (HD-MEAs)

**References**

1. Beggs JM, Plenz D: Neuronal avalanches in neocortical circuits. *J Neurosci* 2003, **23**(35):11167–11177.

2. Bak P: How nature works. Oxford (UK): Oxford University Press; 1997.

3. Pasquale V, Massobrio P, Bologna LL, Chiappalone M, Martinoia S: Self-organization and neuronal avalanches in networks of dissociated cortical neurons. *Neuroscience* 2008, **153**(4):1354–1369.

4. Pastore VP, Poli D, Godjoski A, Martinoia S, Massobrio P: ToolConnect: a functional connectivity toolbox for in vitro networks. *Front Neuroinform* 2016, **10**(13).

5. Izhikevich EM: Simple model of spiking neurons. *IEEE Trans Neur Net* 2003, **14**:1569–1572.

## P173 Attractor dynamics of cortical assemblies underlying brain awakening from deep anesthesia

### Cristiano Capone^1,2^, Núria Tort-Colet^3^, Maria V. Sanchez-Vives^3,4^, Maurizio Mattia^1^

#### ^1^Istituto Superiore di Sanità (ISS), 00161 Rome, Italy; ^2^PhD Program in Physics, Sapienza University, 00185 Rome, Italy; ^3^Institut d’Investigacions Biomèdiques August Pi i Sunyer (IDIBAPS), 08036 Barcelona, Spain; ^4^Institució Catalana de Recerca i Estudis Avançats (ICREA), 08010 Barcelona, Spain

##### Correspondence: Cristiano Capone (cristiano0capone@gmail.com)

*BMC Neuroscience* 2017, **18** (**Suppl 1**):P173

Slow rhythms of activity (~1 Hz) and slow-wave activity [1, 2] are a remarkably reproducible dynamical activity pattern with a low degree of complexity which opens a window on the brain multiscale organization, on top of which cognitive functions emerge during wakefulness. Understanding how such transition takes place might shade light on the emergence of the rich repertoire of neuronal dynamics underlying brain computation. Sleep-wake transition is a widely-studied phenomenon ranging in experimental, computational and theoretical frameworks [3–5], however it is still debated how brain state changes occur. In our previous work [6] we showed from intracortical recordings in anesthetized rats, that sleep-like rhythms fade out when wakefulness is approached giving rise to an alternation between slow Up/Down oscillations and awake-like (AL) activity periods. We also shown how this phase of activity pattern bistability is captured by a mean-field rate-based model of a cortical column. Guided by this mean-field model, spiking neuron networks are devised to reproduce the electrophysiological changes displayed during the transition. Also, the model gave us hints on the mechanistic and dynamical nature of the patterns of activity observed, suggesting that the AL periods appearance is due to a Hopf-like transition from a limit cycle to a stable fixed point at a high level of activity, and that AL-SO alternation is related to the presence of a slow oscillating (∼ 0.2 Hz) level of excitation probably due to populations of neurons in deeper regions of the brain.

We extended our previous findings by performing a stability analysis of the competing attractors, observing a modulation of their stability, that affect the dynamics of the Down-to-AL transition and the residence dynamics within the AL state. Moreover, we found that the mean-field model remarkably matches the stability modulation observed in experiments. This match between theory and experiments further strengthens our claim that cortical assemblies of neurons display a Hopf bifurcation when anesthesia fades out.

Such observation gives important information on intrinsic dynamical properties of the system, suggesting that it does not respond in a passive way but rather it is a strongly nonlinear component, capable to drastically change its dynamics under small changes of relevant parameters. This can provide a computational advantage in terms of the capability of producing a rich repertoire of network states during wakefulness.

**Acknowledgements**

Supported by EC FET Flagship HBP SGA1 (720270) to MM and MVSV

**References**

1. Sanchez-Vives MV, & Mattia M: Slow wave activity as the default mode of the cerebral cortex. *Arch Ital Biol* 2014, **152:**147–155.

2. Capone Cristiano, Mattia Maurizio: Speed hysteresis and noise shaping of traveling fronts in neural fields: role of local circuitry and nonlocal connectivity. *Scientific Reports* 2016, **7:**39611 doi: 10.1038/srep39611

3. Bettinardi RG, Tort-Colet N, Ruiz-Mejias M, Sanchez-Vives MV, & Deco G: Gradual emergence of spontaneous correlated brain activity during fading of general anesthesia in rats: evidences from fMRI and local field potentials. *Neuroimage* 2015, **114:**185–198.

4. G. Deco, P. Hagmann, A. G. Hudetz, and G. Tononi: Modeling resting-stat state functional networks when the cortex falls asleep: local and global changes., *Cereb. Cortex* 2014, vol. 24, no. 12, pp. 3180–3194.

5. Steyn-Ross ML, Steyn-Ross DA, and Sleigh JW: Interacting Turing-Hopf instabilities drive symmetry-breaking transitions in a mean-field model of the cortex: a mechanism for the slow oscillation, *Phys. Rev. X*, vol. 3, no. 2, p. 21005, 2013.

6. Capone C, Tort-Colet N, Mattia M, Sanchez-Vives MV (2016) Multistable attractor dynamics in columnar cortical networks transitioning from deep anesthesia to wakefulness. Bernstein Conference 2016.

## P174 Are receptive fields in visual cortex quantitatively consistent with efficient coding?

### Ali Almasi^1,2^, Shaun L. Cloherty^4^, David B. Grayden^2^, Yan T. Wong^3,4^, Michael R. Ibbotson^1,5^, Hamish Meffin^1,5^

#### ^1^National Vision Research Institute, Australian College of Optometry, Melbourne, Australia; ^2^NeuroEngineering Laboratory, Dept. Biomedical Eng., University of Melbourne, Melbourne, Australia; ^3^Dept. of Physiology, Monash University, Melbourne, Australia; ^4^Dept. of Electrical & Computer Systems Eng., Monash University, Melbourne, Australia; ^5^ARC Centre of Excellence for Integrative Brain Function, University of Melbourne, Melbourne, Australia

##### Correspondence: Hamish Meffin (hmeffin@unimelb.edu.au)

*BMC Neuroscience* 2017, **18** (**Suppl 1**):P174

Numerous studies, across different sensory modalities, suggest that the neural code employed in early stages of the cortical hierarchy can be explained in terms of Efficient Coding. This principle states that information is represented in a neural population so as to minimize redundancy. This is achieved when the features to which neurons are tuned occur in a statistically independent fashion in the sensory environment. The “statistically independent features” can be rigorously identified through methods of statistical inference, and can be associated with a cell’s receptive field (RF). Several studies using these methods have shown a *qualitative* similarity between predicted RFs and those found in primary visual cortex, for simple and complex cells (with linear and non-linear RF structures, respectively).

Recent methods allow direct experimental estimation of RFs. Using these methods, we report on the first *quantitative* evaluation of the Efficient Coding Hypothesis at the level of RF structures, including both simple and complex cells.

Experimental RF structures were estimated from recordings of single-units in the primary visual cortex of anaesthetized cats in response to presentation of Gaussian white noise. RFs were estimated from recordings assuming a General Quadratic Model for spike rate and performing maximum likelihood estimation on the response given the stimulus. Theoretical Efficient Coding RF structures were inferred by performing unsupervised learning on a set of natural images, under the assumption of Efficient Coding that evoked spike rates were statistically independent and sparsely distributed, and using the same General Quadratic Model as for the experimental RFs.

We recovered spatial RF structures from 94 well isolated single-units in 3 cats, of which 26 were classified as simple cells, 38 as complex cells and 30 as a mixed cell class.

The results confirmed the qualitatively similarity of theoretical RF structures from Efficient Coding with those estimated experimentally. However, quantitatively a number of discrepancies were observed as well as similarities. (1) RF orientation tuning was wider experimentally than theoretically (bandwidth was most frequently between 60° and 90° experimentally, while theoretically, it was mostly between 30° and 60°). (2) Spatial frequency tuning was wider experimentally than theoretically (bandwidth was most frequently 2 ± 0.5 octaves experimentally, but only 1 ± 0.5 octaves theoretically). (3) For cells with more than one sub-RF it was possible to compare the tuning to orientation and spatial frequency between different sub-RFs. The difference in orientation tuning between sub-RFs showed that experimentally around 60% cells had precisely matched orientation preferences (<15°), while in the theoretical population this proportion dropped to around 40%. (4) Experimentally, the spatial frequency preference of sub-RFs in the same cell were also tightly matched for the majority of cells (<0.5 octaves), with a similar result in the theoretical population (<0.5 octaves). (5) Finally, the spatial phase relationships of sub-RFs were compared: experimentally a large majority (80%) of cells that had two quadratic sub-RFs that were 90° ± 15° out of phase. In the theoretical population, this spatial phase relationship was common but less prevalent (50%).

The quantitative discrepancies we found were robust to changes in meta-parameters, such as the degree of image compression in pre-processing or the source of natural images. The results suggest that the experimental RFs are sub-optimal in terms of coding efficiency. However, it is important to note that we used a deterministic model of spike rate in response to an image stimulus: a stochastic model is more realistic and may limit the coding efficiency of the theoretical result, bringing it in closer quantitative agreement with experiment.

**Acknowledgements**

AA acknowledges a Melbourne University Postgraduate Research Award. HM and MI acknowledge support from the Australian Research Council Centre of Excellence for Integrative Brain function.

## P175 Cholinergic Modulation of DG-CA3 microcircuit dynamics and function

### Luke Y. Prince^1^, Krasimira Tsaneva-Atanasova^2,3^, Jack R. Mellor^1^

#### ^1^Centre for Synaptic Plasticity, School of Physiology, Pharmacology, and Neuroscience, University of Bristol, Bristol, BS8 1TD, UK; ^2^Department of Mathematic, College of Engineering, Mathematics and Physical Sciences, University of Exeter, Exeter, UK, EX4 4QF; ^3^EPRSC Centre for Predictive Modelling in Healthcare, University of Exeter, Exeter, UK, EX4 4QJ

##### Correspondence: Luke Y. Prince (l.y.prince@bristol.ac.uk)

*BMC Neuroscience* 2017, **18** (**Suppl 1**):P175

Dentate gyrus granule cells provide powerful feedforward excitatory drive onto a local circuit of CA3 pyramidal cells and inhibitory interneurons, and is believed to selectively activate subsets of pyramidal cells in the CA3 recurrent network for encoding and recall of memories. Cholinergic receptors provide a key means to modulate this circuit, increasing cellular excitability and altering synaptic release, but the combined action of these changes on information processing between the dentate gyrus and CA3 remains unknown. We recorded evoked monosynaptic EPSCs and disynaptic IPSCs in CA3 pyramidal cells in response to a range of frequencies and stimulation patterns and in the presence and absence of the cholinergic receptor agonist carbachol (5 μM). We found that carbachol strongly reduced IPSC amplitudes but only mildly reduced EPSC amplitudes. The short-term plasticity dynamics of these responses were used to constrain a computational model of mossy fibre driven transmission across a range of stimulation patterns. This model was then used to analyse how aceytlcholine influences encoding and recall in a spiking neural network model of CA3 to study encoding and recall of neuronal ensembles driven by mossy fibre input. We found that acetylcholine lowers the requirements for encoding neuronal ensembles and increases memory storage in CA3.

## P176 Subthalamic nucleus low frequency fluctuations carry information about future economic decisions in parkinsonian gamblers

### Alberto Mazzoni^1†^, Manuela Rosa^2†^, Jacopo Carpaneto^1^, Luigi M. Romito^3^, Alberto Priori^2,4^, Silvestro Micera^1,5^

#### ^1^Translational Neural Engineering, The Biorobotics Institute, Scuola Superiore Sant’Anna, Pontedera, 56025, Italy; ^2^Clinical Center for Neurostimulation, Neurotechnology and Movement Disorders Fondazione IRCCS Ca’ Granda Ospedale Maggiore Policlinico, Milan, 20122, Italy; ^3^Movement Disorders Department, Neurological Institute Carlo Besta, Milan, 20133, Italy; ^4^Department of Health Sciences, University of Milan & ASST Santi Paolo e Carlo, Milan, 20142, Italy; ^5^Bertarelli Foundation Chair in Translational NeuroEngineering, Institute of Bioengineering and Center for Neuroprosthetics, Ecole Polytechnique Federale De Lausanne, Lausanne, CH-1015, Switzerland

##### Correspondence: Alberto Mazzoni (alberto.mazzoni@santannapisa.it)

^†^equal first author contribution

*BMC Neuroscience* 2017, **18** (**Suppl 1**):P176

Dopamine replacement therapy for the treatment for Parkinson Disease (PD) has been related to an increased risk of occurrence of Impulse Control Disorders (ICD), such as Gambling Disorder (GD) [1]. Previous experimental and modeling studies [2] have shown a link between ICD and specific activity of the subthalamic nucleus (STN), a standard target for Deep Brain Stimulation (DBS) therapy for advanced PD. Several brain areas involved in decision making, impulsivity and reward valuation, such as the prefrontal cortex and striatum, are interconnected to the STN, and activity in these areas might be modulated by STN DBS. Understanding the relationship between STN functioning and ICD would help developing better therapies for PD while shedding light on the mechanisms of human decision making.

To study how STN activity is modulated by gambling, we analyzed low-frequency ([1–12] Hz) fluctuations of STN LFP recorded by DBS electrodes from PD patients during an economic decision making task. All patients were under dopamine replacement therapy, and half of them were affected by GD. In the task patients were asked to decide between a high risk (HR) and low risk (LR) option, the first being associated to a negative expected value, but to a high reward in case of win. Reaction times were strongly affected by trial type, with GD patients and non-GD patients quicker in taking HR and LR decisions respectively, suggesting that decision is actually determined before options presentation. Analyzing low frequency STN LFP we found that amplitude of fluctuations, recorded during specific intervals preceding option presentation, carried significant information about future choices on single trials in patients affected by GD but not in those not affected.

These results complement previous studies about the role of inhibiting impulsive behavior displayed by the STN activity. Beta-range STN fluctuations were found to be modulated by the level of conflict in decisions [3], while our results suggest that the lower frequencies, which are functionally correlated with different cortical areas [4], play instead a role to prevent pathological risk attraction.

**Acknowledgements**

This work was supported by institutional funds from Scuola Superiore Sant’Anna, by the Italian Ministry of Health (GR-2009-1594645 grant), by the Aldo Ravelli Donation for Research on Parkinson Disease, by the Bertarelli Foundation, and by institutional funds from École Polytechnique Federale de Lausanne.

**References**

1. Weintraub D, David AS, Evans AH, Grant JE, Stacy M: Clinical spectrum of impulse control disorders in Parkinson’s disease. *Mov. Disord.* 2015 **30**: 121–127.

2. Frank MJ, Samanta J, Moustafa AA, Sherman SJ: Hold Your Horses: Impulsivity, Deep Brain Stimulation, and Medication in Parkinsonism. *Science* 2007 **318**: 1309–1312.

3. Brittain JS, Watkins KE, Joundi RA, Ray NJ, Holland P, Green AL, Aziz TZ, Jenkinson N A Role for the Subthalamic Nucleus in Response Inhibition during Conflict. *J. Neurosci*. 2012 **32:** 13396–13401.

4. Herz DM, Tan H, Brittain JS, Fischer P, Cheeran B, Green AL, FitzGerald J, Aziz TZ, Ashkan K, Little S, et al. Distinct mechanisms mediate speed-accuracy adjustments in cortico-subthalamic networks. *eLife* 2017 **6**: 10.7554/eLife.21481

## P177 Data-driven computational modeling of CA1 hippocampal principal cells and interneurons

### Rosanna Migliore^1^, Carmen Alina Lupascu^1^, Francesco Franchina^1^, Luca Leonardo Bologna^1^, Armando Romani^2^, Christian Rössert^2^, Sára Saray^3^, Jean-Denis Courcol^2^, Werner Van Geit^2^, Szabolcs Káli^3^, Alex Thomson^4^, Audrey Mercer^4^, Sigrun Lange^4,5^, Joanne Falck^4^, Eilif Muller^2^, Felix Schürmann^2^, and Michele Migliore^1^

#### ^1^Institute of Biophysics, National Research Council, Palermo, Italy; ^2^Blue Brain Project, École Polytechnique Fédérale de Lausanne Biotech Campus, Geneva, Switzerland; ^3^Institute of Experimental Medicine, Hungarian Academy of Sciences, Budapest, Hungary; ^4^University College London, London, United Kingdom; ^5^University of Westminster, London, United Kingdom

##### Correspondence: Rosanna Migliore (rosanna.migliore@cnr.it)

*BMC Neuroscience* 2017, **18** (**Suppl 1**):P177

We present and discuss data-driven models of biophysically detailed hippocampal CA1 pyramidal cells and interneurons of a rat. The results have been obtained by using the Brain Simulation Platform (BSP) of the Human Brain Project and two open-source packages, the Electrophys Feature Extraction Library (eFEL, https://github.com/BlueBrain/eFEL) and the Blue Brain Python Optimization Library (BluePyOpt) [1]. They have been integrated into the BSP in an intuitive graphical user interface guiding the user through all steps, from selecting experimental data to constrain the model, to run the optimization generating a model template and, finally, to explore the model with in silico experiments. Electrophysiological features were extracted from somatic traces obtained from intracellular paired recordings performed using sharp electrodes on CA1 principal cells and interneurons with classical accommodating (cAC), bursting accommodating (bAC) and classical non-accommodating (cNAC) firing patterns. The extracted features, together with user selections for realistic morphological reconstructions and ion channel kinetics, were then used to automatically configure and run the BluePyOpt on the Neuroscience Gateway and/or on one of the HPC systems supporting the BSP operations, such as CINECA (Bologna, Italy) and JSC (Jülich, Germany) in this case. The resulting optimized ensembles of peak conductances for the ionic currents, were used to explore and validate the model behavior during interactive in silico experiments carried out within the HBP Collaboratory. Such a modelling effort has been undertaken in the context of the Human Brain Project and constitutes one of the major steps in the workflow that is being used to build a cellular level model of a rodent hippocampus.

**Acknowledgements**

This project has received funding from the European Union’s Horizon 2020 research and innovation programme under grant agreement No 720270

**Reference**

1. Van Geit W, Gevaert M, Chindemi G, Rössert C, Courcol J-D, Muller EB, Schürmann F, Segev I and Markram H (2016) BluePyOpt: Leveraging Open Source Software and Cloud Infrastructure to Optimise Model Parameters in Neuroscience. *Front. Neuroinform*. **10:**17. doi: 10.3389/fninf.2016.00017

## P178 The interplay between basal ganglia and cerebellum in motor adaptation

### Dmitrii Todorov, Robert Capps, William Barnett, Yaroslav Molkov

#### Department of Mathematics and Statistics, Georgia State University, Atlanta, Georgia 30303-3083, USA

##### Correspondence: Dmitrii Todorov (dtodorov@gsu.edu)

*BMC Neuroscience* 2017, **18** (**Suppl 1**):P178

It is widely accepted that the cerebellum and basal ganglia (BG) and play key roles in motor adaptation (in error based and non-error based one, respectively) [1]. However, despite considerable number of studies, the interactions between BG and cerebellum are not completely understood [1]. In particular, in the experiments it is difficult to dissociate the adaptation performed by cerebellum and by BG. To do so, some studies [2] introduced perception perturbations that were suggested to impair cerebellum’s ability to adapt to errors, and, thus, promoted the BG-based mechanisms. To our knowledge no mathematical model exists that explains the conditions in which visual perturbations make reinforcement learning in the BG the main mechanism of motor adaptation.

We have developed a model that integrates a phenomenological representation of the cerebellum and a previously published firing rate-based description of BG network [3], and mimics the trial-to-trial motor adaptation in 2D reaching arm movements. Cerebellum is implemented as an artificial neural network performing corrections of the motor program, descending from motor cortex to spinal cord, via supervised learning.

Figure 1 below shows the model architecture. Stimulus signal comes from prefrontal cortex (PFC) and is sent to direct and indirect pathways of BG. The strength of PFC → BG connections changes due to reinforcement learning mediated by substantia nigra pars compacta (SNc) dopaminergic input, whose activity is defined by the reward prediction error (RPE) signal. Direct and indirect pathways converge at globus pallidus internus (GPi)/substantia nigra pars reticulata (SNr), which together project to premotor cortex (PMC)/Thalamus to perform action selection. There are also direct PFC → PMC connections representing habitual cue-action associations. The PMC/Thalamus then project to the motor cortex (MC) and to the cerebellum. Cerebellum output represents a correction, which adds to the motor command descending from the MC to the spinal cord. This correction is calculated as a linear transformation of the motor command. The transformation matrix is updated by the supervised learning algorithm, accounting for the vector error provided by the visual feedback. The corrected signal goes to the spinal cord neuron network that controls a two-joint arm to perform center-out reaching movements. The perceived movement endpoint of the is used to compute the vector error and/or the reward.
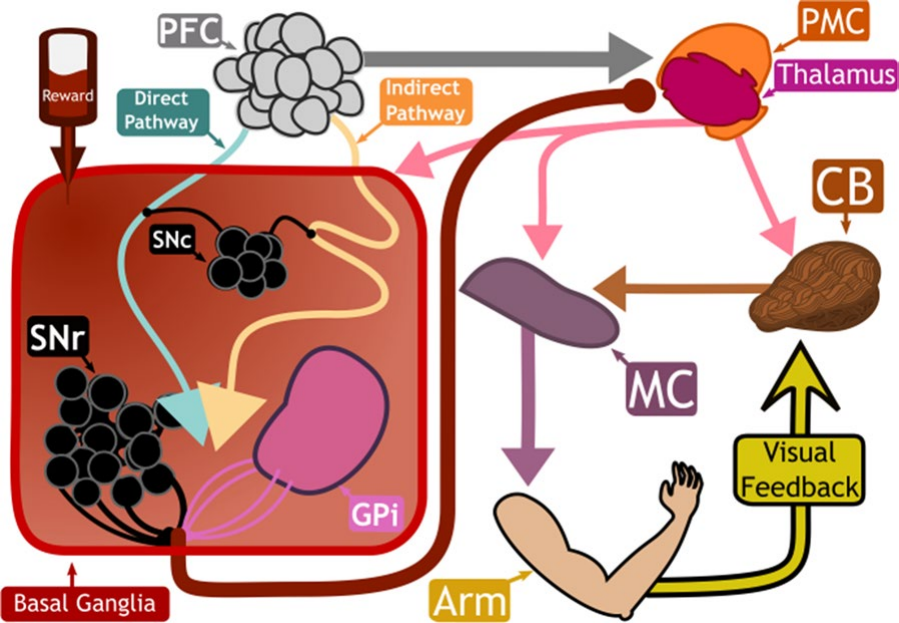


**Figure 1.** Model architecture

Our model simulations suggest that when the perception of the vector error provided to the cerebellum is significantly perturbed, the faulty cerebellar corrections adversely affect or even completely destroy motor adaptation. We speculate and show via simulations that error-based learning in cerebellum has an adaptive critic component which effectively suppresses error-based mechanisms to enable reinforcement-based motor adaptation.

**References**

1. Izawa J, Shadmehr R. Learning from sensory and reward prediction errors during motor adaptation. PLoS Comput Biol. 2011; **7(3):**e1002012.

2. Gutierrez‐Garralda JM, Moreno‐Briseño P, Boll MC, Morgado‐Valle C, Campos‐Romo A, Diaz R, Fernandez‐Ruiz J. The effect of Parkinson’s disease and Huntington’s disease on human visuomotor learning. European *Journal of Neuroscience* 2013;**38(6):**2933–40.4.

3. Kim T, Hamade KC, Todorov D, Barnett WH, Capps RA, Latash EM, Markin SN, Rybak IA, Molkov YI. Reward based motor adaptation mediated by basal ganglia. Frontiers in Computational Neuroscience. 2017;**11**.

## P179 Microscopic and macroscopic dynamics of neural populations with delays

### Federico Devalle^1,2^, Diego Pazó^3^, Ernest Montbrió^1^

#### ^1^Center for Brain and Cognition, Universitat Pompeu Fabra, 08018 Barcelona, Spain; ^2^Department of Physics, Lancaster University, LA1 4YB Lancaster, UK; ^3^Instituto de Fisica de Cantabria (IFCA), CSIC-Universidad de Cantabria, 39005 Santander, Spain

##### Correspondence: Federico Devalle (federico.devalle@upf.edu)

*BMC Neuroscience* 2017, **18** (**Suppl 1**):P179

Bridging descriptions of brain activity across different scales is a major challenge for theoretical neuroscience. Numerous experimental techniques are available to measure brain activity, ranging from single cells recordings to population measurements of the average activity of large ensembles of neurons. It is often in these population-level recordings (e.g. EEG, MEG…), that important phenomena are observed. A particularly relevant example are gamma oscillations, a temporal coherent activity with frequency between 30 and 100 Hz. A large body of experimental and computational works indicates that the interplay between synaptic processing and recurrent inhibition is the key ingredient to generate such oscillations, in a mechanism commonly referred to as Interneuronal Gamma oscillations (ING) [1, 2]. Here, we analyse the dynamics of a network of quadratic integrate-and-fire neurons with time-delayed synaptic interactions, both in their excitable and self-oscillatory regime. Time delays have been indeed shown to approximate the effect of synaptic kinetics [3]. Using the so-called Lorentzian ansatz [4, 5], we derive a set of two delayed firing rate equations (FREs). Due to their analytical tractability, the FREs allow us to find exact boundaries of stability for the parameters regions of oscillatory (collective synchrony-CS) and asynchronous dynamics. Moreover, for inhibitory coupling, we observe a more complex oscillatory state, the so-called quasiperiodic partially synchronized state (QPS). Here, neurons are quasiperiodic, and have a mean frequency different from the global frequency of the entire population, which corresponds to fast brain oscillations (f ~ 80 Hz). Interestingly, macroscopically this state strongly resembles the sparsely synchronized state observed in networks of leaky integrate-and-fire neurons subjected to strong recurrent inhibition and noise [6]. However, microscopically, these two states have qualitatively different dynamics, suggesting a dichotomy between microscopic and macroscopic dynamics. For a certain region of parameters, the QPS coexists also with the CS. Moreover, sufficiently increasing inhibition, the QPS undergoes a series of period doubling bifurcation that eventually leads to chaos. Notably, only the collective dynamics is chaotic, while microscopically neurons are non-chaotic. Finally, we find that while excitation always leads to collective synchronous oscillations, inhibition fails to synchronize neural activity when a precise degree of heterogeneity is exceeded, consistently with previous numerical studies of heterogeneous, inhibitory spiking neural networks [7].

**Acknowledgements**

We acknowledge support by MINECO (Spain) under project No. ~FIS2014-59462-P, and the project COSMOS of the European Union’s Horizon 2020 research and innovation programme under the Marie Sklodowska-Curie grant agreement No.642563.

**References**

1. Whittington MA, Traub RD, Jefferys JG: Synchronized oscillations in interneuron networks driven by metabotropic glutamate receptor activation. *Nature* 1995, **373:**612–615.

2. Whittington MA, Traub RD, Kopell N, Ermentrout B, Buhl EH: Inhibition-based rhythms: experimental and mathematical observations on network dynamics. *Int J Psychophysiol* 2000, **38**:315–336

3. Roxin A, Montbrió E: How effective delays shape oscillatory dynamics in neuronal networks. *Physica D* 2011, **240:** 323–345.

4. Montbrió E, Pazó D, Roxin A: Macroscopic description for Networks of Spiking Neurons. *Phys Rev X* 2015, **5:** 021028

5. Pazó D, Montbrió E: From Quasiperiodic Partial Synchronization to Collective Chaos in Populations of Inhibitory Neurons with Delay. *Phys Rev Lett* 2016, **116:** 238101

6. Brunel N, Hakim V: Fast global oscillations in networks of integrate-and-fire neurons with low firing rates. *Neural Comput* 1999, **11**:1621.

7. Wang XJ, Buzsáki G: Gamma Oscillations by Synaptic inhibition in a Hippocampal Interneuronal Network Model. *J Neurosci* 1996, **16(20)**:6402–6413.

## P180 Motivation signal in anterior cingulate cortex during economic decisions

### Gabriela Mochol^1^, Habiba Azab^2^, Benjamin Y. Hayden^2^, Rubén Moreno-Bote^1^

#### ^1^Center for Brain and Cognition and Department of Information and Communications Technologies, University Pompeu Fabra, Barcelona, 08005, Spain; ^2^Department of Brain and Cognitive Sciences and Center for Visual Sciences, University of Rochester, Rochester, NY 14618, USA

##### Correspondence: Gabriela Mochol (gabriela.mochol@upf.edu)

*BMC Neuroscience* 2017, **18** (**Suppl 1**):P180

Anterior cingulate cortex (ACC) plays regulatory and cognitive roles. Its functions are associated with conflict and performance monitoring, regulation of strategy and response selection, all of which depend on reward monitoring and its anticipation [1]. It has been shown previously that in the condition when the reward was certain and its proximity was cued, animal’s error rate decreases together with the number of trial remaining to the reward [2]. Concurrently, the firing rate of ACC neurons gradually increased or decreased along with reward expectancy. It happened when the reward was certain and correct decisions could only bring animal closer to the reward. However, when certainty about outcome was removed and no notion of reward proximity was provided the progressive modulation of behavior and ACC activity disappeared.

Here we tested whether such motivation signal can be also found in the circumstances when the reward is no longer certain and the animal choices brings reward closer or further away but the information about reward closeness reminds - the situation more common in the economic decisions of everyday life. We recorded single unit activity from dorsal ACC while monkey performed token gambling task. On each trial, monkeys gambled to gain certain number of tokens, but they could also lose tokens. The collection of six tokens resulted in a jackpot reward delivery. The number of collected tokens was displayed on the monitor and was known to the animal. The animal learnt the task and exhibited risk seeking behavior as previously reported [3]. The analysis of behavioral data revealed that animal performance (percent of correct responses) depended on the number of previously collected tokens. The relation was not monotonic with the drop of performance after reward administration. At the same time, the significant fraction of recorded neurons exhibited tuning towards the number of previously collected tokens.

Our preliminary results suggest that ACC monitors rewards in risky conditions, and that neuronal signals could be directly related to the motivation of the animal.

**Acknowledgements**

The Spanish Ministry of Economy and Competitiveness IJCI-2014-21937 grant (to G. M.); the Marie Curie FP7-PEOPLE-2010-IRG grant PIRG08-GA - 2010-276795, and the Spanish Ministry of Economy and Competitiveness PSI2013-44811-P grant (to R. M. B.)

**References**

1. Heilbronner SR, Hayden BY: Dorsal Anterior Cingulate Cortex: A Bottom-Up View. *Annu Rev Neurosci* 2016, **39**: 149–170.

2. Shidara M, Richmond BJ: Anterior Cingulate: Single Neuronal Signals Related to Degree of Reward Expectancy. *Science* 2002, **296(5573):**1483–1490.

3. Azab H, Hayden BY: Shared roles of dorsal and subgenual anterior cingulate cortices in economic decisions. bioRxiv 2016. [http://biorxiv.org/content/early/2016/09/09/074484].

## P181 A simple computational model of altered neuromodulation in cortico-basal ganglia dynamics underlying bipolar disorder

### Pragathi Priyadharsini Balasubramani^1^, Srinivasa V. Chakravarthy^2^, Vignayanandam R. Muddapu^2^

#### ^1^Brain and Cognitive Sciences, University of Rochester, Rochester, New York 14627, USA; ^2^Bhupat and Jyoti Mehta School of Biosciences, Department of Biotechnology, IIT- Madras, Chennai, TN, India

##### Correspondence: Srinivasa V. Chakravarthy (schakra@iitm.ac.in)

*BMC Neuroscience* 2017, **18** (**Suppl 1**):P181

Bipolar disorder (BPD) is characterized by oscillations alternating between manic and depressive episodes causing swings in moods. The length of an episode in a patient’s mood cycle (time period) can vary from hours to years. Some medications popularly used for stabilizing mood include selective serotonin reuptake inhibitors and lithium therapy. This computational study focuses on the serotonergic system dysfunction, and particularly, understanding their contribution to cortico-basal ganglia network (CGBN) dynamics for stability and recurrence of moods. To this end, we try to model the disorder in a decision-making framework that tries to choose between actions of positive or negative affects. We propose a computational model that explores the effects of impaired serotonergic neuromodulation on the dynamics of CBGN and relate this impairment to the manic and depressive episodes of BPD. The proposed model of BPD is derived from an earlier model, that describes the roles of dopamine and serotonin in the action selection dynamics of CBGN. In that model, rewarding actions are selected based on the Utility function, which combines Value and Risk functions as follows (eqn. 1).

1 $$ U_{t} (s_{t} ,a_{t} ) = Q_{t} (s_{t} ,a_{t} ) - \alpha \;sign(Q_{t} (s_{t} ,a_{t} ))\;\sqrt {h_{t} (s_{t} ,a_{t} )} $$

where U, Q and h represent Utility, Value and Risk respectively, for a given state, *s*, and action, *a*, at time, *t*. The parameter *α*, which represents risk preference, is associated with serotonin action in CBGN. Value and Risk are trained by Reinforcement Learning using the Temporal Difference (TD) error, which represents dopamine in CBGN. The lumped model was later extended to a detailed network model of BG. In those models, *α* was a constant, whereas in the current model it varies as per the following dynamics:

2 $$ \dot{\alpha } = \tau_{\alpha } ( - \alpha + A_{r} \bar{r} + \alpha_{k} ) $$

3 $$ \dot{\bar{r}} = \tau_{r} \left( {r - \bar{r}} \right) $$

The variable r-bar tracks the average rewards ‘*r*’ gained through time, and α-dot defines serotonin dynamics with *α*_k_ constant (eqns. 2, 3) indicating basal risk sensitivity levels. The parameter *A*_r_ denotes the amplitude of reward sensitivity, and thus the reward history is proposed to modulate *α* dynamics. When the model is run in a simple two arm bandit task - one rewarding (+ve reward) and the other punitive (-ve reward) with probability 0.5, under normal conditions the network shows high preference for rewarding actions. But for certain ranges of reward sensitivity (*A*_r_) and basal risk sensitivity (*α*_k_) the model exhibits oscillations reminiscent of BPD mood oscillations (Fig. 1). There exists clinical and experimental evidence supporting abnormality in serotonin levels and reward sensitivity in case of BPD. Specifically, high reward sensitivity with medium levels of risk sensitivity (serotonin activity correlate, as tonic/basal levels or that induced by medication), can trigger bipolar mood oscillations. This preliminary model can be extended to a detailed network model. Future work will include expanding CBGN with neural models of limbic system, and predicting plausible treatment strategies for effectively dealing with the onset and progression of BPD symptoms.
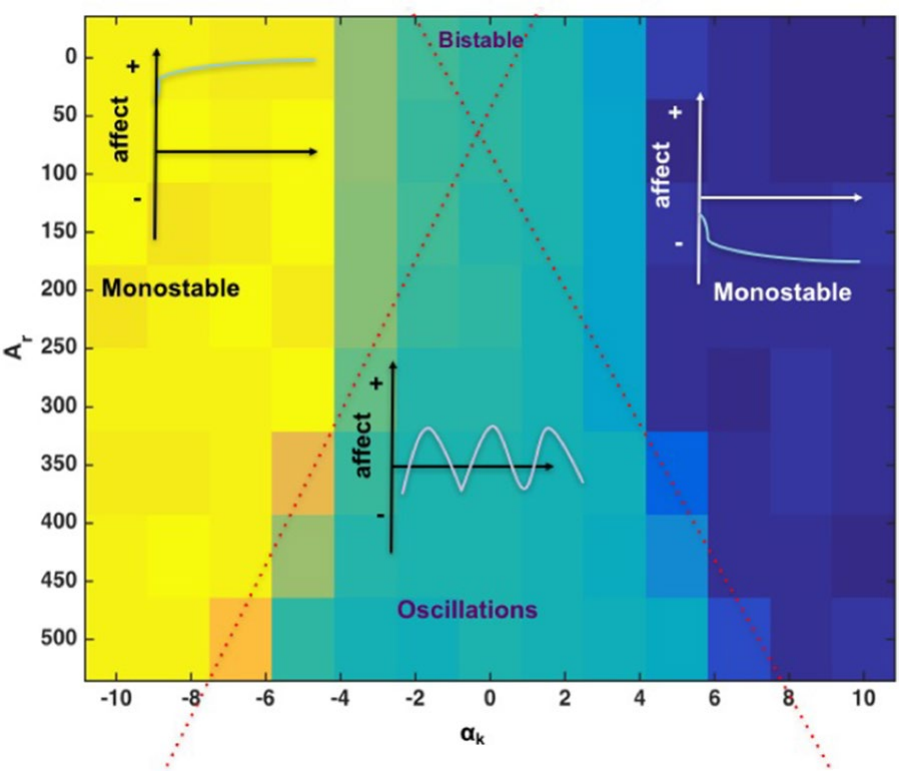


**Figure 1**. Action (positive or negative affect) selection in CBGN model: Yellow: rewarding (+ve) action selection as in healthy controls; Green: Oscillations between +ve and –ve actions as in BPD; Blue: -ve action selection as in depression

## P182 Theta/alpha coordination of pre-motor and parietal networks during free behavior in rats

### Medorian D. Gheorghiu^1^, Bartul Mimica^2^, Jonathan Withlock^2^, Raul C. Mureșan^1^

#### ^1^Romanian Institute of Science and Technology, Cluj-Napoca, Cluj 400552, Romania; ^2^Centre for Neural Computation, Kavli Institute for Systems Neuroscience, Trondheim, NO-7491, Norway

##### Correspondence: Medorian D. Gheorghiu (medorian@gmail.com)

*BMC Neuroscience* 2017, **18** (**Suppl 1**):P182

Activity of posterior parietal cortex (PPC) neurons exhibits self-motion tuning to both ongoing and impeding movements, which may reflect behavioral planning [1]. A major input to PPC originates from the frontal medial agranular cortex (AGm), which is believed to be involved in complex motor planning. In the monkey, Pesaran and colleagues [2] showed that fronto-parietal coherence is stronger in free-choice tasks than in instructed trials, probably activating different decision-related circuits in these areas. Therefore, we hypothesize that in the rat the interaction between AGm and PPC may be instrumental in coordinating decision making and motor planning. Here, we are investigating the coupling strength between PPC and AGm in the theta/alpha frequency band by computing pairwise spectral coherence and phase delays across the two areas (see Figure 1) during goal-directed spatial navigation in rats. Two tasks were implemented: an instructed or “known” task where the rat had to run straight to a fixed well named “Home”; an “exploratory” task where the rat had to search for reward delivered in “Target” wells located randomly across the arena and then run back to the Home well.

**Results:** As the rat stopped running and started licking at the target well, there was an increase in theta coupling strength accompanied by a gradual decrease in frequency (Figure 1A). Using the phase information, we computed the delay of PPC relative to AGm. The delay decreased sharply from ~5.5 to ~2.5 ms when the rat arrived at the target location (see Figure 1B), and it was gradually resetting in the last 5 s that the rat spent at that location (see Figure 1D). As suggested by anatomical evidence, AGm was leading PPC indicating a causal interaction where AGm coordinates the activity in PPC.

**Conclusions:** Our results indicate a complex regulation of oscillatory behavior in PPC and AGm during free behavior in rats. In particular, a pronounced ongoing oscillation in the theta/alpha band is expressed throughout the task and seems to be coordinated across the two areas. AGm leads PPC and both the frequency of the oscillation and the time delay between the two areas change as a function of behavioral events.
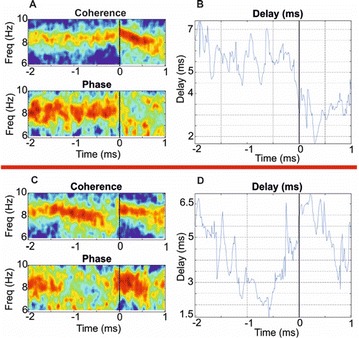


**Figure 1. A and C.** Time-resolved spectral coherence between PPC and AGm in the 6-10 Hz frequency band, aligned to the initiation (A) and cessation (C) of licking at the target well. **B and D**. phase delays in ms between PPC and AGm aligned to the initiation (B) and cessation (D) of licking at the target well

**Acknowledgements**

This work was supported by CNCS - UEFISCDI (PN-II-RU-TE-2014-4-0406 and PN-III-P3-3.6-H2020-2016-0012).

**References**

1. Withlock J., Robert J. Sutherland, Menno P. Witter, May-Britt Moser, Edvard I. Moser: Navigating from hippocampus to parietal cortex. *PNAS 2008 vol. 105.39*: 14755–14762;

2. Pesaran B, Nelson MJ, Andersen RA: Free choice activates a decision circuit between frontal and parietal cortex. *Nature* 2008: 406–409.

## P183 Information theoretic approach towards identifying changes in cellular-level functional connectivity and synchrony across animal models of schizophrenia

### Jennifer L. Zick^1,2^, Kelsey Schultz^4^, Rachael K. Blackman^1,2,3^, Matthew V. Chafee^1,3^, Theoden I. Netoff^1,4^

#### ^1^Graduate Program in Neuroscience, University of Minnesota, Minneapolis, MN 55455 USA; ^2^Medical Scientist Training Program (MD/PhD), University of Minnesota, Minneapolis, MN 55455 USA; ^3^Brain Sciences Center, VA Medical Center, Minneapolis, MN 55417 USA; ^4^Department of Biomedical Engineering, University of Minnesota, Minneapolis, MN 55455 USA

##### Correspondence: Jennifer L. Zick (zick@umn.edu)

*BMC Neuroscience* 2017, **18** (**Suppl 1**):P183

Schizophrenia has long been described as a syndrome of disordered connectivity in the brain. While originally based on clinical symptomatology, neurophysiological evidence for this concept has been found in imaging studies in humans with schizophrenia. It has also been found that cortical pyramidal neurons have a reduced density of the synaptic spines necessary for cellular communication in postmortem brain tissue recovered from people with schizophrenia. However, functional evidence for disconnectivity at the level of local neuronal circuits is limited. To address this question, we characterized neuronal dynamics between groups of simultaneously recorded cortical neurons in data obtained from both primate and mouse models of schizophrenia. Neural data were obtained from multielectrode recording arrays inserted into the parietal and prefrontal cortices of macaque monkeys while the animals performed a cognitive control task that measures a specific cognitive impairment in human patients with schizophrenia. Phencyclidine, an NMDA receptor (NMDAR) antagonist that has long been used as a pharmacological model of psychosis, was administered systemically on alternating days with injections of saline. In the mouse experiments, analogous data were obtained from medial prefrontal cortex in awake head-fixed mice during locomotion. Data from Nestin-promoted Dgcr8^+/−^ mutant mice (*DiGeorge syndrome critical region 8*; a gene strongly associated with schizophrenia in humans and shown to produce schizophrenia-like symptomatology in mice) is compared with that obtained from wildtype littermate controls.

Cross-correlation analysis was performed on spike trains from pairs of simultaneously recorded neurons to characterize changes in synchrony between conditions. In the primate neural data, cross correlations frequently displayed a prominent “zero-lag” peak representing a large number of coincident action potentials between cells in the control condition that could be a result of common input. In the phencyclidine condition, there was a reduction in synchronous firing between pairs of cells. A similar rate-independent reduction in precise synchrony was also found in medial prefrontal cortical neuronal ensemble recordings obtained from Dgcr8 mice as compared to controls, suggesting that this is may be a consistent finding related to the root pathophysiology of schizophrenic processes.

To characterize deficits in synaptic communication between neurons in the disease state, we employed higher-order transfer entropy (TE) metrics to identify pairs of cells that exhibited effective connectivity (Ito et al, 2011, PLOS One). Consistent with the disconnection hypothesis of schizophrenia, we found that acute administration of PCP resulted in a reduction in the percent of cell pairs identified as significantly functionally connected by TE analysis, as well as a reduction in the overall distribution of population shared information. This result suggests a cellular basis for the reduced information-processing capabilities seen in schizophrenics performing prefrontal cortex-dependent tasks, as well as synaptic disconnection. Furthermore, this result is supported by a similar reduction in both number of functionally connected cell pairs and overall shared information in prefrontal cortex in the Dgcr8^+/−^ mouse genetic model of schizophrenia.

In summary, these results display a reduction in both zero-lag synchrony and cellular-level functional connectivity in two very distinct animal models of schizophrenia. It is well known that coincident firing of action potentials facilitates connectivity between neurons, and asynchrony results in disconnection. Thus, the results presented here support the notion that alterations in precise spike timing may be an underlying driving factor towards reduced functional connectivity in schizophrenia, providing a new mechanistic model for disease pathophysiology.

**Acknowledgements**

This material is based upon work supported by the NIH (R01 MH1107491; Chafee); NRSA F30 MH108205-01A1 (Zick); NSF Career Award (TIN); Medical Scientist Training Program NIH T32-008244

## P184 Neural Suppression with Deep Brain Stimulation using a Linear Quadratic Regulator

### Nicholas Roberts^1^, Vivek Nagaraj^2,^, Andrew Lamperski^3^, Theoden I. Netoff^1^

#### ^1^Department of Biomedical Engineering, University of Minnesota, Minneapolis, MN 55455, USA; ^2^Graduate Program in Neuroscience, University of Minnesota, Minneapolis, MN 55455, USA; ^3^Department of Electrical and Computer Engineering, University of Minnesota, Minneapolis, MN 55455, USA

##### Correspondence: Nicholas Roberts (robe1521@umn.edu)

*BMC Neuroscience* 2017, **18** (**Suppl 1**):P184

Current neuromodulation techniques for seizure suppression, such as vagus nerve or deep brain stimulation, have shown some clinical efficacy. Yet their application is complicated by the large parameter space of electrical stimulation settings inherent to these systems. A physician must skillfully choose stimulation parameters such as frequency, amplitude, and pulse width for each individual patient in order to effectively reduce their incidence of seizures. We demonstrate an algorithm capable of automatically generating a continuous stimulation waveform to suppress neural activity and minimize total stimulation energy.

We treat the suppression of neural activity as a linear-quadratic-Gaussian (LQG) control problem. The resulting optimal controller consists of a Kalman filter and a linear-quadratic regulator (LQR). The effectiveness of the LQG controller in suppressing seizure biomarkers was first verified in a computational model of epilepsy called Epileptor [1], which simulates local field potential (LFP) recordings within a seizure focus. We built a model of the generated LFPs using the Ho-Kalman algorithm [2] for subspace system identification. The Kalman filter estimated the state of the system and a feedback control signal provided by the LQR successfully prevented seizures during stimulation, even while varying the Epileptor model parameters.

We then implemented the LQG controller in an in vivo rodent model. We stimulated the ventral hippocampal commissure while recording in the hippocampus. The Ho-Kalman algorithm was again used to build a dynamical systems model of the LFP activity based on the evoked response to Gaussian white noise stimulation. We used a three-phase experiment to test the LQG controller: 2 min of baseline activity; 2 min of closed-loop neural stimulation; and 2 min post-stimulation to check if LFPs return to baseline levels. This same stimulation waveform was then replayed in “open-loop,” without state estimation from the Kalman filter. The LFP power from 1-100 Hz was used to measure performance. Our results show a significant decrease in LFP power during closed-loop stimulation. Open-loop stimulation produced negligible change in LFP power. The LQG controller was confirmed to be an effective tool for minimizing LFP activity within a selected frequency band. The mathematical models of neural dynamics it uses are subject specific and determine stimulation waveforms based on state to suppress neural activity.

**References**

1. Jirsa VK, Stacey WC, Quilichini PP, Ivanov AI, Bernard C: On the nature of seizure dynamics. *Brain* 2014, **137** (pt. 8):2210–2230.

2. Miller DN, & de Callafon RA: Identification of linear time-invariant systems via constrained step-based realization. *IFAC Proceedings Volumes* 2012, ***45*** **(16):** 1155–1160.

## P185 Reinforcement learning for phasic disruption of pathological oscillations in a computational model of Parkinson’s disease

### Logan L. Grado^1^, Matthew D. Johnson^1,2^, Theoden I. Netoff^1^

#### ^1^Department of Biomedical Engineering, University of Minnesota, Minneapolis, MN, 55455, United States; ^2^Institute for Translational Neuroscience, University of Minnesota, Minneapolis, MN, 55455, United States

##### Correspondence: Logan L. Grado (grado@umn.edu)

*BMC Neuroscience* 2017, **18** (**Suppl 1**):P185

Deep brain stimulation (DBS) is an effective therapy for motor symptoms of PD, and is often used as a complement to medication in patients who have progressed to severe stages of PD. However, programming these devices is difficult and time consuming, and DBS therapy is limited by side effects and partial efficacy [1]. Furthermore, traditional continuous DBS (cDBS) does not account for fluctuations in motor symptoms caused by factors such as sleep, attention, stress, cognitive and motor load, and current drug therapy [2], and as the patient’s state changes, so does the need for stimulation. Current cDBS strategies are incapable of adapting to the needs of patients: once the clinician sets the parameters, they do not change until the next programming visit. *In this study, we have created a reinforcement learning (RL) algorithm capable of learning online how best to stimulate to reduce pathological oscillations* in silico. We have developed the reinforcement learning DBS (RL-DBS) algorithm for tuning DBS parameters, and have tested it on a biophysically realistic mean-field model of the basal ganglia-thalamocortical system (BGTCs) [3], simulating parkinsonian neural activity. The RL-DBS algorithm decides when to deliver stimulus pulses based upon the real-time amplitude and phase of the pathological oscillation in order to reduce the amplitude of that oscillation. The algorithm learns which actions lead to the highest cumulative reward (i.e. reduction of oscillation amplitude). After training on the model, the RL-DBS algorithm is able to learn both phase and amplitude selectivity to optimally reduce the pathological oscillation. The algorithm learns the expected reward for both actions (not stimulating and stimulating) as a function of the phase/amplitude of the oscillation (Figure. 1A, Figure. 1B). The algorithm then decides which action to execute based upon the action difference (Figure. 1C). Additionally, the algorithm learns to deliver bursts of stimulation phase-locked to the oscillation.

We created an adaptive RL-DBS algorithm capable of learning on-line how to reduce the power of a pathological oscillation in a computation model of PD. The algorithm has the potential to deliver individualized, adaptive DBS therapy that can improve the quality of life for PD patients.
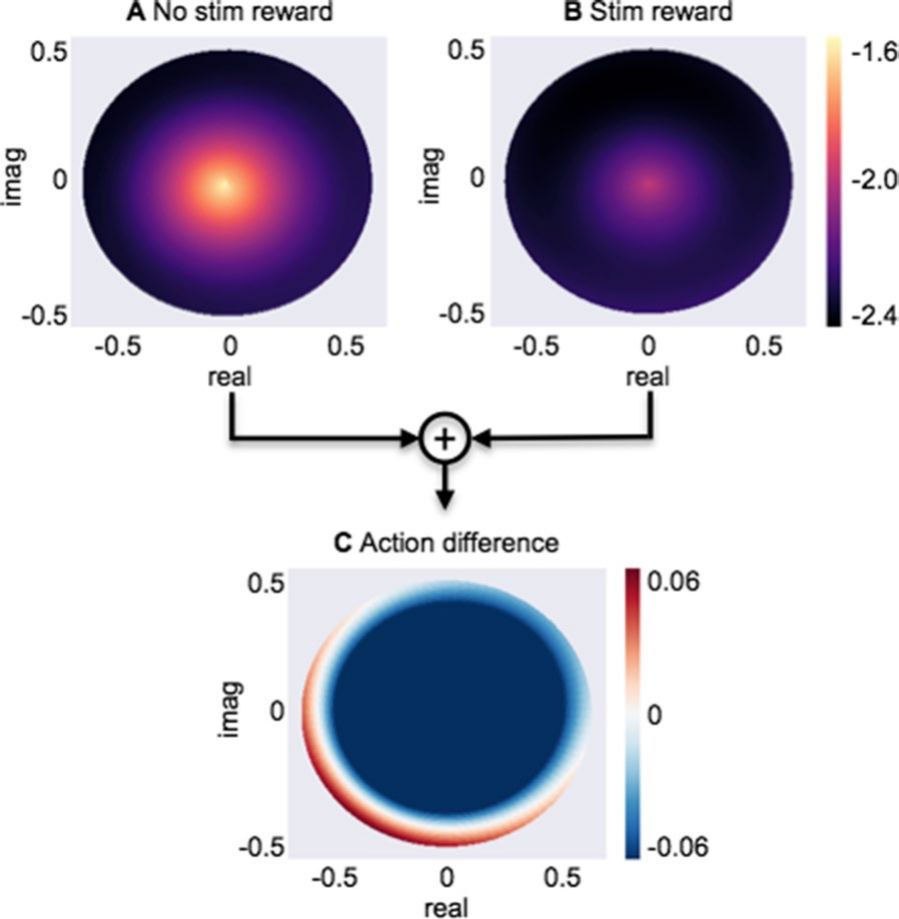


**Figure. 1.** Learned reward maps **A, B** and action difference **C** as a function of the phase and amplitude of the oscillation. **A and B** show the learned reward for no stimulation and stimulation respectively, while **C** shows the action difference. The algorithm selects the action that with the highest expected reward. The action difference reveals that the algorithm learns both phase- and amplitude-selective stimulation

**Acknowledgements**

Research supported by the *Systems Neuroengineering* NSF IGERT Program (DGE-1069104), NIH R01-NS094206, NIH P50-NS098573, and NSF CBET-1264432.

**References**

1. G. Deuschl, S. Paschen, and K. Witt: Clinical outcome of deep brain stimulation for Parkinson’s disease. *Handb. Clin. Neurol.*, vol. 116, pp. 107–128, 2013.

2. J. a Obeso, M. C. Rodríguez-Oroz, M. Rodríguez, J. L. Lanciego, J. Artieda, N. Gonzalo, and C. W. Olanow: Pathophysiology of the basal ganglia in Parkinson’s disease. *Trends Neurosci.*, vol. 23, no. 10 Suppl, pp. S8–S19, 2000.

3. S. J. van Albada and P. a Robinson. Mean-field modeling of the basal ganglia-thalamocortical system. I Firing rates in healthy and parkinsonian states. *J. Theor. Biol.*, vol. 257, no. 4, pp. 642–63, Apr. 2009.

## P186 Metrics for detection of delayed and directed coupling

### David P. Darrow^1^, Theoden I. Netoff^2^

#### ^1^Department of Neurosurgery, University of Minnesota, Minneapolis, MN 55455, USA; ^2^Department of Biomedical Engineering, University of Minnesota, Minneapolis, MN 55455, USA

##### Correspondence: David P. Darrow (Darro015@umn.edu)

*BMC Neuroscience* 2017, **18** (**Suppl 1**):P186

Detecting delayed coupling in dynamical systems remains a challenging frontier in Neuroscience. Frequently used tools such as cross-correlation have been shown to be robust against measurement noise but fail to identify coupling direction. [1] More recently developed tools such as multivariate granger causality and various forms of transfer entropy provide methods of detecting direction of coupling but may be less resilient to measurement noise and require more substantial quantities of data depending on the signal to noise ratio. With widespread use of these tools, it is important to have a complete understanding of the limitations of each metric and the circumstances of optimal use in experimental design.

To test these metrics over a salient parameter space, a linear, delayed vector autoregressive model was created with probabilistic and complex coupling over probabilistic time delays. The model was run with various measurement noise strengths, numbers of nodes, and number of available data points. Correlation, cross-correlation, mutual information, multivariate granger causality (MVGC), and transfer entropy (TE) were computed and compared to true coupling adjacency matrices using an L-2 metric.

Significant differences were found between reconstruction results between metrics. MVGC was found to outperform all other metrics when the signal to noise ratio exceeded 0.23. Transfer entropy and correlation fared worse than maximum cross-correlation and mutual information, as summarized in Figure 1. Reconstruction error was found to be minimally affected by number of nodes for metrics other than MVGC and TE, where MVGC outperformed all others. Similarly, MVGC and TE required a minimum number of samples to converge, and the required number of points was found to be a function of the number of nodes.
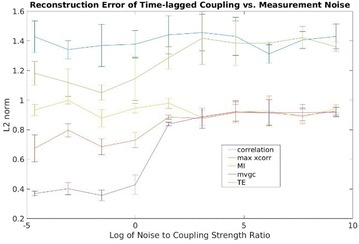


**Figure 1.** Reconstruction error of time-lagged coupling as a function of measurement noise with standard deviations

**Conclusions:** Based on this work, significant disparity exists between the performance of existing methods to detect delayed coupling. Many common tools fail to detect delayed coupling. However, even with a minimal density of time points to number of nodes, MVGC efficiently recovers complex and delayed coupling. Careful consideration should be given to metrics used in experiments where coupling may be delayed or spread out over time. Measurement noise and data sample density requirements may affect experimental design.

**References**

1. Netoff TI, Carroll TL, Pecora LM, Schiff SJ. 11 detecting coupling in the presence of noise and nonlinearity. *Handbook of Time Series Analysis: Recent Theoretical Developments and Applications*. John Wiley & Sons; 2006;

2. Barnett L, Seth AK. The MVGC multivariate Granger causality toolbox: a new approach to Granger-causal inference. J. Neurosci. Methods. Elsevier; 2014;**223**:50–68.

3. Lindner M, Vicente R, Priesemann V, Wibral M. TRENTOOL: a Matlab open source toolbox to analyse information flow in time series data with transfer entropy. BMC Neurosci. BioMed Central Ltd; 2011;12:119.

4. Barnett L, Barrett AB, Seth AK. Granger causality and transfer entropy Are equivalent for Gaussian variables. Phys. Rev. Lett. 2009;**103**:2–5.

## P187 Insurgence of network bursting events in formed neuronal culture networks: a computational approach

### Davide Lonardoni^1^, Hayder Amin^1^, Stefano Di Marco^2^, Alessandro Maccione^1^, Luca Berdondini^1†^, Thierry Nieus^1,3†^

#### ^1^Neuroscience and Brain Technology Department, Fondazione Istituto Italiano di Tecnologia, Genova, Italy, 16163; ^2^Scienze cliniche applicate e biotecnologiche, Università dell’Aquila, L’Aquila, Italy, 67100; ^3^Dept. of Biomedical and Clinical Sciences “Luigi Sacco”, University of Milan, Milan, Italy

##### Correspondence: Davide Lonardoni (davide.lonardoni@iit.it)

^†^co-senior authors

*BMC Neuroscience* 2017, **18** (**Suppl 1**):P187

A common property of developing neuronal systems is their intrinsic ability to generate spatiotemporally propagating spiking activity involving a large number of highly synchronously firing neurons. Primary neuronal cultures are among the experimental preparations that allow the investigation of the principles underlying the generation of such spontaneous coordinated spiking activity: cell cultures self-organize during development up to the stage where they elicit stereotyped network-wide spiking activity, called network bursts. The high spatial resolution of the high-density CMOS multi-electrode arrays revealed that network bursts correspond to a coordinated propagation of action potentials throughout the network [1]. Specifically, these propagations could be well clustered into few groups differing for their ignition sites (i.e. the starting point) and propagation paths (i.e. the mean trajectory followed by the spiking activity) [2]. This finding suggests the presence of regions in charge of triggering such spontaneous events. Following this direction, we investigated what were the main determinants underlying the generation of network bursts in cell cultures at the mature stage. To this end, we implemented a network model made of principal cells (excitatory) and fast spiking (inhibitory) neurons endowed with the proper synaptic currents (AMPA, NMDA, GABA). With minimal topological constraints on the coupling between neuronal pairs (i.e. a network structure based on the reciprocal distance among neurons), the model expressed realistic spontaneous activities that mimicked the experimental findings.

The results obtained in this study, by combining experimental datasets with our neural network computational model, shows that while the synaptic contribution is mainly involved in shaping the network burst, the key player in the generation of network bursts could be found in the local properties of the neuronal network.

Specifically, with functional connectivity analysis, we found and detected, both in simulation and in experiments, a few and specific ‘hot spots’ of the networks that matched with the ignition sites of the propagations. In particular, in the model, the neurons of to the hot spots were much more responsive than any other region to mild stimulations delivered to these regions. Although the connectivity was truly uniform by design we found that the ‘hot spots’ were characterized by local graph properties (i.e. higher clustering, lower path length respect to the remaining network) that favor the amplification of asynchronous firing and determine the onset of a network event. Our modeling study suggests that the ‘hot spots’ might naturally result from the simple constraints on the network topology and the sparseness of the network.

**Acknowledgements**

We acknowledge the financial support of the Future and Emerging Technologies (FET) programme within the Seventh Framework Programme for Research of The European Commission for the SICODE project, under FET-Open grant number: FP7-284553 and for the NAMASEN Marie-Curie Initial Training Network, under FET-Open grant number: FP7-264872.

**References**

1. Berdondini L, Imfeld K, Maccione A, Tedesco M, Neukom S, Koudelka-Hep M, Martinoia S: Active pixel sensor array for high spatio-temporal resolution electrophysiological recordings from single cell to large scale neuronal networks. *Lab Chip* 2009, **9(18):**2644–2651.

2. Gandolfo M, Maccione A, Tedesco M, Martinoia S, Berdondini L: Tracking burst patterns in hippocampal cultures with high-density CMOS-MEAs. *J Neural Eng* 2010, **7(5):**056001.

## P188 Brian2GeNN: Free GPU Acceleration for Brian 2 Users

### Marcel Stimberg^1^, Dan F. M. Goodman^2^, Thomas Nowotny^3^

#### ^1^Sorbonne Universités, UPMC Univ Paris 06, INSERM, CNRS, Institut de la Vision, Paris, France; ^2^Department of Electrical and Electronic Engineering, Imperial College, London, UK; ^3^School of Engineering and Informatics, University of Sussex, Brighton, UK

##### Correspondence: Thomas Nowotny (t.nowotny@sussex.ac.uk)

*BMC Neuroscience* 2017, **18** (**Suppl 1**):P188

Over the last decade graphics processing units (GPUs) have evolved into powerful, massively parallel co-processors that are increasingly used for scientific computing and machine learning. But it has also become quite clear that writing efficient code for GPU accelerators is difficult even with APIs designed for general purpose computing, such as CUDA and OpenCL. As a consequence, frameworks are being developed for making GPU acceleration available for specific applications without complex parallel code design. Examples include Matlab GPU extensions [1], TensorFlow GPU support [2], Theano GPU extensions [3] and so on. Here we present the first public release of Brian2GeNN [4], a software package that connects the popular Brian 2 simulator [5] to the GPU enhanced neuronal networks (GeNN) framework [6] to provide effortless GPU support for computational Neuroscience investigations to Brian 2 users.

Brian2GeNN was first announced at CNS*2014 and has undergone a long phase of maturation and development until its first public release this year. It is a Python based package that allows users to deploy their Brian 2 models to a device named “genn”, using the simple command “set_device(‘genn’)”. This triggers the use of Brian2GeNN, which generates code that can be executed on GPUs using GeNN. Brian2GeNN supports all common features of Brian 2 with few exceptions such as multi-compartment models, multiple networks or heterogeneous delays.

On this poster, we present the basic principles of how Brian2GeNN works and benchmark examples of its performance with a number of different benchmark models and using a number of diverse GPU accelerators. We can demonstrate that depending on the model and the accelerator, achieved speedups can vary considerably. Brian2genn is Open Source and freely available on GitHub under GPL v2.

**Acknowledgements**

The development of Brian2GeNN was partially supported by EPSRC, grant EP/J019690/1.

**References**

1. Mathworks web pages [https://uk.mathworks.com/company/newsletters/articles/gpu-programming-in-matlab.html], accessed 03-03-2017.

2. TensorFlow web pages [https://www.tensorflow.org/tutorials/using_gpu], accessed 03-03-2017.

3. Theano documentation [http://theano.readthedocs.io/en/latest/tutorial/using_gpu.html], accessed 03-03-2017.

4. Brian2genn repository [https://github.com/brian-team/brian2genn], accessed 03-03-2017

5. Stimberg M, Goodman DFM, Benichoux V, Brette R: Equation-oriented specification of neural models for simulations. *Front. Neuroinf*. 2014, doi: 10.3389/fninf.2014.00006.

6. E. Yavuz, J. Turner and T. Nowotny (2016). GeNN: a code generation framework for accelerated brain simulations. *Scientific Reports* 2016, **6**:18854. doi: 10.1038/srep18854.

## P189 Spike counts in the visual cortex consistently encode both stimuli and behavioral choices in a change-detection task

### Veronika Koren^1,2^, Valentin Dragoi^3^, Klaus Obermayer^1,2^

#### ^1^Neural Information Processing Group, Institute of Software Engineering and Theoretical Computer Science, Technische Universität Berlin, Berlin, 10587, Germany; ^2^Bernstein Center for Computational Neuroscience Berlin, Berlin, Germany; ^3^Department of Neurobiology and Anatomy, University of Texas Medical School, Houston, Texas, 77030, US

##### Correspondence: Veronika Koren (veronika.koren@ni.tu-berlin.de)

*BMC Neuroscience* 2017, **18** (**Suppl 1**):P189

In visual discrimination tasks, the subject collects information about sensory stimuli and makes behavioral decisions accordingly. In this study, we are searching for coding strategies in visual cortices of the macaque (*macaca mulatta*) that relate to both stimuli and behavior. Multi-units within a single cortical column are recorded in V1 and V4 areas simultaneously while the subject is performing a change detection task with matching and non-matching stimuli. We assess systematic differences in distribution of spike counts for matching vs. non-matching stimuli (detection probability) and for correct vs. incorrect behavioral performance (choice probability, [1]) on the single cell and on the population level. In addition, we estimate pair-wise correlations of spike counts. The spiking signal is weakly but significantly predictive on the type of stimulus (matching vs. non-matching stimuli with correct behavioral responses) as well as on different behavioral choices with correct and incorrect behavioral performance (correct vs. incorrect behavioral responses on non-matching stimuli). In both areas, the effect is limited to the superficial layers of the cortical column. Detection and choice probability are consistent, the behavioral choice “match” being characterized by higher spike counts in both cases. In V1, but not in V4, the signal corresponding to the choice”match” is even statistically invariant with changes in both the type of the stimulus and the behavioral performance. In incorrect trials, neural activity in V1 is in addition characterized by a systematic bias in spike counts already at the beginning of the trial. The bias is consistent with the future behavioral choice and is only present in the deep cortical layers. Comparing the distribution of correlation coefficients across pairs of neurons with matching and non-matching stimuli, distribution of coefficients in V4 is less variable with matching stimuli, in particular for short (0-0.5 mm) and middle-range (0.5-1 mm) inter-neuron distances. This effect could be interpreted as a fast adaptation of neural responses to two consecutive presentations of the same stimuli [2]. A change in long-range (>1 mm) correlations in V4 is observed when comparing trials with correct and incorrect behavioral performance, correlations in incorrect trials showing higher variability. In V1, we did not observe any systematic changes in spike-count correlations with different stimuli. However, correlations are significantly more variable in trials with incorrect compared to correct behavioral performance. This effect is once again limited to deep cortical layers. Higher variability of correlations in V1 might be a signature of spontaneously generated network state that is more likely leading to incorrect behavioral performance. Finally, we test the interactions between choice probabilities and spike-count correlations. Choice probabilities and correlations do not interact in V1, but weakly interact in the V4 area, where cells with similar choice probabilities tend to be more strongly correlated. In summary, we observe various differences in the first and second order statistics of spike counts in both V1 and V4 areas. The first order statistics is related to coding of both stimuli and behavioral choices while correlations would rather modulate the efficacy of encoded signals.

**Acknowledgements**

This work was supported by the Deutsche Forschungsgemeinschaft (GRK1589/2).

**References**

1. Britten KH, Newsome WT, Shadlen MN, Celebrini S, Movshon JA: A relationship between behavioral choice and the visual responses of neurons in macaque MT. *Visual Neurosci* 1996, **13(1):**87–100.

2. Gutnisky DA, Dragoi V: Adaptive coding of visual information in neural populations. *Nature* 2008, **452(7184):** 220–224.

3. Hansen BJ, Chelaru MI, Dragoi V: Correlated variability in laminar cortical circuits. *Neuron* 2012, **76(3)**: 590–602.

4. Nienborg H, Cumming BG: Decision-related activity in sensory neurons may depend on the columnar architecture of cerebral cortex. *J.Neurosci.* 2014, **34(10)**: 3579–85.

## P190 Local topology of connectome stabilizes critical points in mean field model

### Samy Castro^1,2^, Mariano Fernandez^3^, Wael El-Deredy^4^, Patricio Orio^1,5^

#### ^1^Centro Interdisciplinario de Neurociencia de Valparaíso, Universidad de Valparaíso, Valparaíso, 2360102, Chile; ^2^Programa de Doctorado en Ciencias, mención en Neurociencia, Facultad de Ciencias, Universidad de Valparaíso, Valparaíso, 2360102, Chile; ^3^Laboratorio de Electrónica Industrial, Control e Instrumentación, Universidad Nacional de La Plata, La Plata, Argentina; ^4^Escuela de Ingeniería Biomédica, Universidad de Valparaíso, 2362905, Valparaíso, Chile; ^5^Instituto de Neurociencia, Universidad de Valparaíso, Facultad de Ciencias, Universidad de Valparaíso, Valparaíso, 2360102, Chile

##### Correspondence: Samy Castro (samy.castro@cinv.cl)

*BMC Neuroscience* 2017, **18** (**Suppl 1**):P190

The interplay between structural connectivity (SC) and neural dynamics is still not yet fully understood. Applying topological analysis, the connectome approach links this anatomical network to brain function. Here we adopt a computational approach to find topology features related to the stability on global neural dynamics. A previous study of a mean field model based on the human cortex network, shows at least 2 global neural states, with either a low or high firing rate pattern [1, 3]. These 2 possible states, or bistability, emerge in the model within a range of the global coupling parameter *G*, limited by critical values *G*_-_ and *G*_+_[1, 3]. Also, at this bistable range, this model achieves the highest correlations with empirical resting state fMRI data. How the network connectivity pattern shapes the critical *G* values has not been yet investigated. Our aim is to identify local or global topology features related to the critical *G* values. We studied 4 different SC networks: a cortical parcellation of human brain [2], a human binary equivalent, a Random Network (RN) having the same degree distribution as human SC, and an equivalent Watts & Strogatz Small World (SW) network. For each of the analyzed networks, values in their critical *G* points have small or null variability. Then, we selectively prune the edges of the networks and calculate their critical *G* values to show the effect of structure pattern in maintaining the bistable dynamics. The edges were pruned selectively based on either the degree or the *k* core decomposition measure; interpreted as a local or global topology feature, respectively. Also, the pruning procedure is applied to the edges on one of 3 specific ways: i) high degree/*k* core nodes, ii) random cuts, and iii) low degree/no *k* core nodes. The highest shifts in critical *G* values are achieved when the edges of high degree or *k* core nodes are pruned. In contrast, when we prune those edges belong to low degree or no *k* core nodes, the shifts in the critical *G* points are irrelevant. We interpret this as that the model can use either local or global connectivity pattern in order to stabilize the critical *G* points. Furthermore, our study show that shifts in the critical *G* points are statistically equivalent when the degree distribution (but not *k* core structure) is shared, such as in the binary human SC compared to the RN. Therefore, in our simulation the degree distribution, interpreted as a local connectivity feature, determines the critical G points for bistability, capturing the essential structural pattern of the network. We also show that it is possible to obtain bistability in other types of networks, suggesting that structure dynamic relationships may obey a topological principle.

**Acknowledgements**

SC is recipient of a Ph.D. fellowship from CONICYT. PO is partially funded by the Advanced Center for Electronic Engineering (FB0008 CONICYT, Chile). The Centro Interdisciplinario de Neurociencia de Valparaíso (CINV) is a Millennium Institute supported by the Millennium Scientific Initiative of the Ministerio de Economía (Chile).

**References**

1. Deco G, McIntosh AR, Shen K, Hutchison RM, Menon RS, Everling S, Hagmann P, Jirsa VK: Identification of optimal structural connectivity using functional connectivity and neural modeling. *J Neurosci.* 2014, **34(23):**7910–7916.

2. Hagmann P, Cammoun L, Gigandet X, Meuli R, Honey CJ, Van Wedeen J, Sporns O: Mapping the structural core of human cerebral cortex. *PLoS Biol.* 2008, **6(7):**1479–1493.

3. Deco G, Ponce-Alvarez A, Mantini D, Romani GL, Hagmann P, Corbetta M: Resting-state functional connectivity emerges from structurally and dynamically shaped slow linear fluctuations. *J Neurosci. 2013*, **33(27):** 11239–11252.

## P191 How chaos in neural oscillators determine network behavior

### Kesheng Xu^1^, Jean Paul Maidana^1^, Patricio Orio^1,2^

#### ^1^Centro Interdisciplinario de Neurociencia de Valparaíso, Universidad de Valparaíso, Valparaíso, Chile; ^2^Facultad de Ciencias, Instituto de Neurociencia, Universidad de Valparaíso, Valparaíso, Chile

##### Correspondence: Patricio Orio (patricio.orio@uv.cl)

*BMC Neuroscience* 2017, **18** (**Suppl 1**):P191

Chaotic dynamics of neural oscillations has been shown at the single neuron and network levels, both in experimental data and numerical simulations. Theoretical works suggest that chaotic dynamics enrich the behavior of neural systems, by providing multiple attractors in a system. However, the contribution of chaotic neural oscillators to relevant network behavior has not been systematically studied yet. We investigated the synchronization of neural networks composed of conductance-based neural models that display subthreshold oscillations with regular and burst firing [1]. In this model, oscillations are driven by a combination of persistent Sodium current, a hyperpolarization-activated current (Ih) and a calcium-activated potassium current, very common currents in the CNS. By small changes in conductance densities, the model can be turned into either chaotic or non-chaotic modes [2]. We study synchronization of heterogeneous networks where conductance densities are drawn from either chaotic or non-chaotic regions of the parameter space. Measuring mean phase synchronization in a small-world network with electrical synapses, we characterize the transition from unsynchronized to synchronized state as the connectivity strength is increased. First, we draw densities from fixed-size regions of the parameter space and find the transition to synchronized oscillations is always smooth for chaotic oscillators but not always smooth for the nonchaotic ones. However, non-smooth transitions were found to be associated to a change in firing pattern from tonic to bursting. Nevertheless, we noticed that chaotic oscillators display a wider distribution of firing frequencies than non-chaotic oscillators, thus making more heterogeneous networks. Next, we draw the conductance densities from the parameter space in a way that maintained the same distribution of firing frequencies (hence the heterogeneity of the network) for both chaotic and non-chaotic. In this case, synchronization curves are very similar, being second order phase transition for both cases. However, we cannot discard that non-chaotic oscillators become chaotic (or vice versa) when in a network, because of the extra parameter associated to the electrical synapse. Finally, when the chaos-inducing Ih current is removed, the transition to synchrony occurs at a lower value of connectivity strength but with a similar slope.

Our results suggest that the chaotic nature of the individual oscillators may be of minor importance to the synchronization behavior of the network. Ongoing work is being conducted to measure the chaotic nature of the whole network, and how it is related to the synchrony behavior.

**Acknowledgements**

KX is funded by Proyecto Fondecyt 3170342. PO is partially funded by the Advanced Center for Electrical and Electronic Engineering (FB0008 Conicyt, Chile). The Centro Interdisciplinario de Neurociencia de Valparaíso (CINV) is a Millennium Institute supported by the Millennium Scientific Initiative of the Ministerio de Economía (Chile).

**References**

1. Orio P., Parra A., Madrid R., González O., Belmonte C., Viana F. Role of Ih in the Firing Pattern of Mammalian Cold Thermoreceptors. *J Neurophysiol* 2012, **108**:3009–3023

2. Xu K., Maidana JP, Caviedes M, Quero D, Aguirre P and Orio P. Hyperpolarization-activated current induces period-doubling cascades and chaos in a cold thermoreceptor model. *Front Comput Neurosci* 2017, **11**:12. doi: 10.3389/fncom.2017.00012.

## P192 STEPS 3: integrating stochastic molecular and electrophysiological neuron models in parallel simulation

### Weiliang Chen^1^, Iain Hepburn^1^, Francesco Casalegno^2^, Adrien Devresse^2^, Aleksandr Ovcharenko^2^, Fernando Pereira^2^, Fabien Delalondre^2^, Erik De Schutter^1^

#### ^1^Computational Neuroscience Unit, Okinawa Institute of Science and Technology Graduate University, Okinawa, Japan; ^2^Blue Brain Project, École Polytechnique Fédérale de Lausanne, Lausanne, Switzerland

##### Correspondence: Weiliang Chen (w.chen@oist.jp)

*BMC Neuroscience* 2017, **18** (**Suppl 1**):P192

Stochastic spatial molecular reaction-diffusion simulators, such as STEPS (STochastic Engine for Pathway Simulation) [1], often face great challenges when simulating large scale complex neuronal pathways, due to the massive computation required by the models. This issue becomes even more critical when combining with cellular electrophysiological simulation, one of the main focuses in computational neuroscience research. One example is our previous research on stochastic calcium dynamics in Purkinje cells [2], where a biophysical calcium burst model was simulated on approximate ¼ of a Purkinje cell dendritic tree morphology using the serial implementation of spatial Gillespie SSA and electric field (EField) solver in STEPS 2.0. Even with a state-of-the-art desktop computer, it still took months to finish the simulation, significantly slowing down research progress.

One possible, yet not trivial approach to speedup such simulation is parallelization. In CNS2016 we reported our early parallel implementation of an Operator-Splitting solution for reaction-diffusion systems, which achieved super-linear speedup in simulation of the buffer components of the above published model on full Purkinje cell morphology. While the performance of our parallel implementation was promising, the test model had no calcium presented in the system and only buffers were simulated. Since buffers were uniformly distributed in the geometry, the loading of each computing process was relatively balanced, resulting in a close to ideal scenario for parallel computation. The membrane potential computation, as well as voltage-dependent reactions in the published model, were omitted due to the lack of a parallel EField solver at the time. In a recent publication [3], we further extended the model by applying a dynamically updated calcium influx profile extracted from the published calcium burst simulation. Our result shown that in a realistic scenario with dynamic calcium influx, data recording, and without special load balancing, our parallel reaction-diffusion solution can still achieve more than 500 times of speedup with 1000 computing processes comparing to the conventional serial SSA solution.

STEPS 3 is the first public release out of the collaboration between the CNS Unit of OIST and the Blue Brain Project of EPFL. The ongoing collaboration aims to deliver a scalable parallel solution for future integrated stochastic molecular and electrophysiological neuron modelling. Combining the parallel TetOpSplit molecular solver developed by OIST and EPFL’s parallel EField solver based upon the PETSc library, our new release addresses the limitations of above test cases, and allows full scale parallel simulation of the complete Purkinje cell calcium burst model. It also contains new changes that are essential to parallel STEPS modelling and simulation pipeline, such as the improved python binding using Cython technology. In this poster, we will use this model as an example to showcase the general procedure of converting a serial STEPS simulation to its parallel counterpart using these new changes. We will also analyze the performance and scalability of our integrated solution, and discuss the direction of future STEPS development.

**References**

1. Hepburn, I., Chen, W., Wils, S., and De Schutter, E. (2012). STEPS: efficient simulation of stochastic reaction–diffusion models in realistic morphologies. *BMC Systems Biology* 6, 36. doi:10.1186/1752-0509-6-36.

2. Anwar, H., Hepburn, I., Nedelescu, H., Chen, W., and De Schutter, E. (2013). Stochastic calcium mechanisms cause dendritic calcium spike variability. *J. Neurosci.* 33, 15848–15867. doi:10.1523/JNEUROSCI.1722-13.2013.

3. Chen, W., and De Schutter, E. (2017). Parallel STEPS: Large Scale Stochastic Spatial Reaction-Diffusion Simulation with High Performance Computers. *Front. Neuroinform.* 11, 137–15. doi:10.3389/fninf.2017.00013.

## P193 A conductance-based model of cerebellar molecular layer interneurons

### Peter Bratby^1^, Erik de Schutter^1^

#### ^1^Okinawa Institute of Science and Technology Graduate University, 1919-1 Tancha, Onna-son, Kunigami-gun, Okinawa 904-0495, Japan

##### Correspondence: Peter Bratby (peter.bratby@oist.jp)

*BMC Neuroscience* 2017, **18** (**Suppl 1**):P193

The cortex of the cerebellum is one of the most well-characterized regions of the brain, comprising three distinct layers whose connectivity is well understood. Numerical simulations of parts of the cerebellar cortex, including the granular layer and Purkinje cell layer, have been instrumental in revealing the computational properties of the cerebellum. However, one important part of the cortex - the molecular layer - has yet to be modeled in detail.

The molecular layer is comprised of many thousands of parallel fibers (the long unmyelinated axons of granule cells), Purkinje cell dendrites and a network of inhibitory interneurons termed stellate cells and basket cells. The inhibitory interneurons were originally classified according to their morphology, although modern molecular techniques have indicated that they are likely to belong to a single class of neuron, the molecular layer interneuron (MLI). As well as forming excitatory connections onto Purkinje cells, parallel fibers make disynaptic connections via MLIs. Furthermore, MLIs form chemical and electrical connections with each other via GABAergic synapses and gap junctions. Thus, the MLIs form a sophisticated inhibitory network whose properties are important in shaping the output of the cerebellum itself.

We develop a detailed conductance-based model of an MLI, and present the results of a simulation of a small MLI network. The neuron model, developed using NEURON simulation software, comprises somatic and dendritic compartments containing distinct voltage- and calcium-dependent ion channels. Two types of synapse are simulated, representing chemical synapses and gap junctions. The connectivity and cellular geometry of the network model conforms with morphological reconstructions, and the model parameters were tuned in order to reproduce known electrophysiological properties of MLIs, including spontaneous spiking activity, modest spike frequency adaptation and the presence of a slow depolarization wave.

## P194 An Ultrasensitive ON/OFF Switch Mechanism Controls the Early Phase of Cerebellar Plasticity

### Andrew R. Gallimore, Erik De Schutter

#### Computational Neuroscience Unit, Okinawa Institute of Science and Technology Graduate University, Onna-son, Okinawa, Japan

##### Correspondence: Andrew R. Gallimore (andrew.gallimore@oist.jp)

*BMC Neuroscience* 2017, **18** (**Suppl 1**):P194

The expression of postsynaptic long-term depression (LTD) and long-term potentiation (LTP) in cerebellar Purkinje cells results from the internalisation or insertion, respectively, of postsynaptic AMPA receptors (AMPAR) [1]. LTD is induced by concurrent parallel fiber and climbing fiber stimulation of Purkinje cells, and is regulated by a complex intracellular signaling network that suppresses phosphatase activity leading to activation of a positive feedback loop that maintains PKC activity for at least 30 min [2]. LTP is dependent on nitric oxide [3], produced during parallel fiber stimulation [4], which nitrosylates N-ethylmaleimide-sensitive factor (NSF) and promotes exocytosis of AMPARs by actively disrupting the interaction between AMPAR-GluR2 and protein interacting with C-kinase 1 (PICK-1) [5, 6].

We report the largest and most sophisticated model of bidirectional synaptic plasticity to date at the PF-PC synapse. Our unified molecular model replicates both PF-PC LTD and NO/NSF-dependent LTP, as well as the sharp calcium threshold separating them. The importance of the positive feedback loop in LTD expression is now well-established. However, the control of feedback loop activation and deactivation has, until now, remained obscure. Model simulations reveal that the feedback loop is activated by an ultrasensitive ‘on-switch’ controlled by CaMKII activation. Furthermore, as predicted by experiments showing that the feedback loop is not required once the early phase of LTD induction is complete [2, 7], our model reveals a rapid and automatic ‘switch-off’ mechanism controlled by phosphatase activity. We are also able to replicate several experimental observations that have so far remained unexplained. These include reconciling conflicting data regarding the importance of nitric oxide in LTD induction: nitric oxide supports loop activation by augmenting phosphatase inhibition, but is not required when the calcium signal is high or sustained [4]. In addition, experiment has shown that selective inhibition of the cytosolic phosphatase, PP2A, elicits robust LTD, whereas inhibition of other phosphatases does not [8]. We show that only PP2A inhibition causes CaMKII-independent activation of the feedback loop and thus LTD induction, revealing the importance of PP2A in suppressing spontaneous loop activation under basal conditions.

**References**

1. Wang YT, Linden DJ: Expression of cerebellar long-term depression requires postsynaptic clathrin-mediated endocytosis. *Neuron* 2000, **25**(3):635–647.

2. Tanaka K, Augustine GJ: A positive feedback signal transduction loop determines timing of cerebellar long-term depression. *Neuron* 2008, **59**(4):608–620.

3. Lev-Ram V, Wong ST, Storm DR, Tsien RY: A new form of cerebellar long-term potentiation is postsynaptic and depends on nitric oxide but not cAMP. *Proceedings of the National Academy of Sciences of the United States of America* 2002, **99**(12):8389–8393.

4. Bouvier G, Higgins D, Spolidoro M, Carrel D, Mathieu B, Lena C, Dieudonne S, Barbour B, Brunel N, Casado M: Burst-Dependent Bidirectional Plasticity in the Cerebellum Is Driven by Presynaptic NMDA Receptors. *Cell Reports* 2016, **15**(1):104–116.

5. Huang Y, Man HY, Sekine-Aizawa Y, Han YF, Juluri K, Luo HB, Cheah J, Lowenstein C, Huganir RL, Snyder SH: S-nitrosylation of N-ethylmaleimide sensitive factor mediates surface expression of AMPA receptors. *Neuron* 2005, **46**(4):533–540.

6. Hanley JG, Khatri L, Hanson PI, Ziff EB: NSF ATPase and alpha-/beta-SNAPs disassemble the AMPA receptor-PICK1 complex. *Neuron* 2002, **34**(1):53–67.

7. Tsuruno S, Hirano T: Persistent activation of protein kinase C alpha is not necessary for expression of cerebellar long-term depression. *Molecular and Cellular Neuroscience* 2007, **35**(1):38–48.

8. Launey T, Endo S, Sakai R, Harano J, Ito M: Protein phosphatase 2A inhibition induces cerebellar long-term depression and declustering of synaptic AMPA receptor. *Proceedings of the National Academy of Sciences of the United States of America* 2004, **101**(2):676–681.

## P195 The use of hardware accelerators in the STochastic Engine for Pathway Simulation (STEPS)

### Guido Klingbeil, Erik de Schutter

#### Computational Neuroscience Unit, Okinawa Institute of Science and Technology, 1919-1 Tancha, Onna-son, Kunigami-gun, Okinawa 904-0495, Japan

##### Correspondence: Guido Klingbeil (guido-klingbeil@oist.jp)

*BMC Neuroscience* 2017, **18** (**Suppl 1**):P195

STEPS is a stochastic reaction-diffusion simulator. Its emphasis is on accurately simulating signaling pathways [1].

The Human Brain Project (HBP) is a European Project set out to gain long-sought insights into our brain and the processes that fundamentally make us human. A parallelised version of STEPS will be part of the Brain Simulation Platform of the Human Brain Project by efficiently simulating reaction-diffusion models in realistic morphologies [2]. The HPB will model the brain at unprecedented detail. It is becoming apparent that such large scale and computationally expensive models are required to either capture more realistic morphologies or to simulate more complex systems [3].

Hardware accelerators such as NVidia’s graphics processing units (GPU) or Intel’s Xeon Phi are one approach to mitigate the high computational cost of such models. They are, in general, massively parallel multicore co-processors and have become a cornerstone of modern high performance computing [4].

The hardware architecture of these two accelerator families differ significantly and thus require different software approaches. While both are programmable via the common programming interface OpenCL, important features such as unified memory or remote direct memory access (RDMA) are often only supported in the native hardware architecture specific programming frameworks [5, 6]. These not only need to be integrated into an overall parallel software system performing a coherent spatial simulation but also need to scale well over several accelerators and compute nodes.

Previous research has shown that we can exploit the computational power of accelerators to improve spatially homogenous stochastic simulations by two orders of magnitude while avoiding the limitation imposed to the size of the reaction system to be simulated by the small fast memory space [7].

STEPS implements a spatial version of Gillespie’s stochastic simulation algorithm computing reaction-diffusion systems on a mesh of tetrahedral sub-volumes [1, 8]. Operator splitting techniques allow to separate the reaction of molecules within a sub-volume from the diffusion of molecules between them.

We develop a layered hybrid software architecture using classic central processing units as well as multiple accelerators, integrated into STEPS. Multiple sub-volumes are assigned to an accelerator. To accommodate the different hardware characteristics, NVidia GPUs are applied within a sub-volume and the Intel Xeon Phi at the level of the operator splittings. Furthermore, due to differences in the performance characteristics of the accelerators the use of load balancing at the tetrahedral mesh level will be important.

Our architecture will be a plug-in solution to STEPS not requiring any changes to the interfaces towards the user or other software systems of STEPS itself.

**References**

1. Hepburn et al.: STEPS: efficient simulation of stochastic reaction-diffusion models in realistic morphologies. *BMC Syst Bio* 2012, **6:36**.

2. The Human Brain Project Brain Simulation Platform [https://www.humanbrainproject.eu/brain-simulation-platform1].

3. Anwar et al.: Stochastic Calcium Mechanisms Cause Dendritic Calcium Spike Variability. *J Neurosci 2013*, **33(40)**:15848–15867.

4. TOP500 Supercomputer Site [http://www.top500.orgError! Hyperlink reference not valid.].

5. Khronos OpenCL Working Group: The OpenCL Specification, V 2.1, 2015.

6. NVidia: CUDA C programming guide, V 8.0, 2017, [https://developer.nvidia.com/cuda-toolkit].

7. Klingbeil et al.: Stochastic simulation of chemical reactions with cooperating threads on GPUs. (in preparation).

8. Gillespie: Exact stochastic simulation of coupled chemical reactions. *J Phys Chem* 1977, **81(25)**:2340–2361.

## P196 A model of CaMKII sensitivity to the frequency of Ca^2+^ oscillations in Cerebellar Long Term Depression

### Criseida Zamora and Erik De Schutter

#### Computational Neuroscience Unit, Okinawa Institute of Science and Technology Graduate University, Okinawa 904-0895, Japan

##### Correspondence: Criseida Zamora (criseida.chimal@oist.jp)

*BMC Neuroscience* 2017, **18** (**Suppl 1**):P196

Cerebellar Long Term Depression (LTD) is a form of synaptic plasticity involved in motor learning. The LTD signaling network includes a PKC-ERK-cPLA_2_ positive feedback loop and mechanisms of AMPAR receptor trafficking. Experimental studies suggest that Ca^2+^/calmodulin-dependent protein kinase II (CaMKII) is required for the LTD induction [1]. Additionally, theoretical and experimental work has shown that CaMKII is sensitive to the frequency of Ca^2+^ oscillations [2, 3]. The activation and autophosphorylation of CaMKII by Ca^2+^ and calmodulin (CaM) are thought to influence its ability to decode Ca^2+^ oscillations. However, the molecular mechanism by which this sensitivity contributes to LTD is not fully understood.

The CaMKII enzyme is a multimeric complex conformed by 12 subunits, each of which contains a catalytic domain, a regulatory domain, and a carboxyl-terminal association domain. Due to the combinatorial complexity of activation of this enzyme, we chose to model four-subunits. We propose a model for the activation of CaMKII by Ca^2+^ in LTD signaling network. These reactions include: activation of the enzyme by Ca^2+^/CaM binding, intersubunit autophosphorylation at threonine residue Thr286, Ca^2+^-independent activation state through autophosphorylation and secondary intersubunit autophosphorylation at threonine residue Thr305/306. Noise in the signaling networks plays an important role in cellular processes. CaMKII models including its activation have been developed [3], but they have not included the intrinsic stochasticity of molecular interactions.

Our lab recently developed a stochastic model of the LTD signaling network including a PKC-ERK-cPLA_2_ feedback loop, Raf-RKIP-MEK interactions and AMPAR trafficking [4]. We have extended this model by adding the molecular network regulating CaMKII activity and its activation. This new model was solved stochastically by STEPS (STochastic Engine for Pathway Simulation) [5] to simulate the influence of noise on the LTD signaling network.

Through stochastic modeling we observed that CaMKII can decode the frequency of Ca^2+^ spikes into different amounts of kinase activity during LTD induction. This result is congruent with previous studies of CaMKII sensitivity to Ca^2+^ oscillations [2]. Furthermore, we observed that PKC activity is highly sensitive to the frequency, amplitude, duration and the number of Ca^2+^ oscillations and consequently has an important effect on LTD activation. The LTD signaling network involves phosphatases and phosphodiesterases related with CaMKII activity, such as PP2A and PDE1. Our stochastic model may be useful in understanding the role of these enzymes in the CaMKII sensitivity to the frequency of Ca^2+^ oscillations.

**References**

1. Hansel C, de Jeu M, Belmeguenai A, Houtman SH, Buitendijk GH, Andreev D, De Zeeuw CI, Elgersma Y: αCaMKII is essential for cerebellar LTD and motor learning. *Neuron* 2006, **51:**835–843.

2. Paul De Koninck and Howard Schulman: Sensitivity of CaM Kinase II to the Frequency of Ca2 + Oscillations. *Science 1998*, **279:** 227–230.

3. Geneviève Dupont, Gerald Houart, Paul De Koninck: Sensitivity of CaM Kinase II to the Frequency of Ca2 + Oscillations: a simple model. *Cell Calcium 2003*, **34:** 485–497

4. Iain Hepburn, Anant Jain, Himanshu Gangal, Yukio Yamamoto, Keiko Tanaka-Yamamoto and Erik de Schutter. A Model of Induction of Cerebellar Long-Term Depression Including RKIP Inactivation of Raf and MEK. *Front Mol Neurosci 2017*, **10:** 19.

5. Hepburn I, Chen W, Wils S, De Schutter E: STEPS: efficient simulation of stochastic reaction-diffusion models in realistic morphologies. *BMC Syst Biol* 2012, **6:**36.

## P197 Exploring the response to climbing fiber input in Purkinje neurons by a new experimental data based model

### Yunliang Zang, Erik De Schutter

#### Computational Neuroscience Unit, Okinawa Institute of Science and Technology Graduate University, Onna-son, Okinawa, Japan

##### Correspondence: Yunliang Zang (yunliang.zang@oist.jp)

*BMC Neuroscience* 2017, **18** (**Suppl 1**):P197

Purkinje neurons receive powerful climbing fiber (CF) input from Inferior Olive (IO) neurons to provide an instructive signal for cerebellar learning. The initial observation that CF input causes all or none responses has been questioned in recent years. However, the mechanisms of initiation and propagation of dendritic calcium spikes evoked by CF input are still poorly understood. Here, we build a new Purkinje cell model based on available experimental data to explore dendritic and somatic responses to CF input in the Purkinje cell under different conditions. All the ionic current models are well constrained according to the experimental data.

Model ionic currents regulate the electrophysiological properties of the Purkinje cell consistent with experimental observations. Our model reproduces a plethora of experimental observations, properties that are critical for the model to be able to predict responses to excitatory and inhibitory inputs. Both simple spike and complex spikes initiate first in the axonal initial segment (AIS). The first derivative and second derivative of the somatic simple spike are in agreement with experimental data.

Using this model, we can explain the discrepancies between experimental observations from different groups about the spatial propagation range of dendritic calcium spikes. Dendritic spikelets can initiate and propagate in a branch-specific manner and depolarization of dendrites can cause secondary spikelets. We find that the timing of occurrence of a spikelet is critical to determine whether it can affect somatic firing or not. The branch-specific dendritic spikelets can combine with contaminant excitatory input and inhibitory inputs to affect somatic firing output more efficiently. Our results indicate that voltage-dependent and branch specific spikelets may enrich CF instructive signals for cerebellar learning.

## P198 Effects of network topology perturbations on memory capacity in a hippocampal place cell model

### Patrick Crotty, Eric Palmerduca

#### Department of Physics and Astronomy, Colgate University, Hamilton, NY 13346, USA

##### Correspondence: Patrick Crotty (pcrotty@colgate.edu)

*BMC Neuroscience* 2017, **18** (**Suppl 1**):P198

The relationship between the structure, or topology, of a neural network and its dynamics remains largely unexplored. This relationship may be particularly significant for the place cell network in region CA3 of the hippocampus. Place cells are believed to encode position by firing when the animal is in a specific spatial location [1]. Multiple “charts” mapping place cells to locations for several different environments may be stored simultaneously in the network [2]. Given hippocampal neurogenesis and synaptic plasticity, the place cell network should be robust to small perturbations in its topology: it shouldn’t “forget” charts if the pattern of synaptic connections changes slightly. Conversely, if Alzheimer’s or another neurodegenerative disease attacks the place cell network, declines in the chart capacity could provide clues about the presence and progression of the disease. Using a computational model based on a place cell network model published by Azizi et al. [3], we investigated the effects that random removal of synapses in the network had on chart capacity. When small numbers of synapses were removed, the chart capacity was not measurably affected, but larger numbers removed caused the chart capacity to decline (see the Figure 1). Moreover, the decline in the chart capacity depended on how the synapses were selected. If they were selected with uniform probability, the chart capacity remained unaffected out to about 10% removed and then fell sharply. But if neurons, rather than synapses, were first selected with uniform probability, and then synapses randomly removed from the selected neurons, the chart capacity began to fall linearly at about 5% removed. These results suggest that the place cell network chart capacity is indeed stable to small perturbations in its topology, and that the effects of larger disruptions depend on the underlying mechanisms, i.e., whether it is the synapses or the cells themselves that are targeted by a disease.
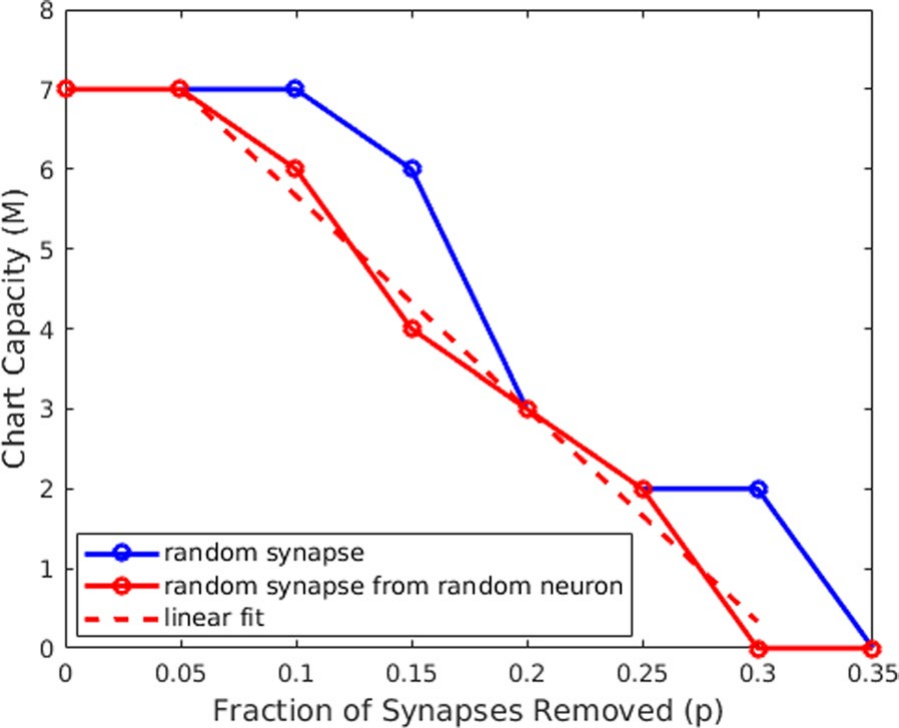


**Figure 1.** The chart capacity (*M*) as a function of the fraction of the synapses removed from the network (*p*), using two different synapse-removal algorithms. For the blue curve, *synapses* are selected and removed with equal probability. For the red curve, *neurons* are selected with equal probability, and then a random synapse is removed from the selected neuron. The dashed line is a linear fit to the random-neuron (red) curve, with slope -0.27

**Acknowledgements**

We thank A. Azizi and S. Cheng for helpful discussions.

**References**

1. O’Keefe J, Dostrovsky J: The hippocampus as a spatial map: preliminary evidence from unit activity in the freely-moving rat. *Brain Research* 1971, **34:**171–175.

2. Alme CB, Miao C, Jezek K, Treves A, Moser EI, and Moser M: Place cells in the hippocampus: eleven maps for eleven rooms. *Proceedings of the National Academy of Sciences* 2015, **111(52):**18428–18435.

3. Azizi A, Wiskott L, Cheng S: A computational model for preplay in the hippocampus. *Frontiers in Computational Neuroscience* 2013, **7(161):**1–15.

## P199 A NEST-simulated cerebellar spiking neural network driving motor learning

### Alberto Antonietti^1^, Claudia Casellato^1^, Csaba Erö^2^, Egidio D’Angelo^3^, Marc-Oliver Gewaltig^2^, Alessandra Pedrocchi^1^

#### ^1^Department of Electronics, Information and Bioengineering, Politecnico di Milano, Milano, Italy; ^2^Blue Brain Project, Ecole Polytechnique Fédérale de Lausanne (EPFL), Biotech Campus, Geneva, Switzerland; ^3^Department of Brain and Behavioral Sciences, University of Pavia, Pavia, Italy

##### Correspondence: Alberto Antonietti (alberto.antonietti@polimi.it)

*BMC Neuroscience* 2017, **18** (**Suppl 1**):P199

The brain organization is optimized to drive adaptive behavior. A key role in the control loop is played by the cerebellum, which implements prediction, timing and learning of motor commands, through complex plasticity mechanisms [1]. However, how plasticity is engaged during the behavior is still unclear. Cerebellar properties emerge in sensorimotor paradigms, such as the Eye Blink Classical Conditioning (EBCC). *In silico* simulations based on computational models are fundamental to investigate the physiological mechanisms. We developed a cerebellar network running on NEST. NEST is a simulator for spiking neural network models [2], focused on the dynamics, size and structure of neural systems by the generation of networks of single-point neurons. We built a network tailored on the mouse cerebellum. The network is made of 71,440 neurons: 250 Mossy Fibers (MF), 5’000 Glomeruli (Glom), 65’600 Granular Cells (GR), 100 Golgi Cells (GO), 400 Purkinje Cells (PC), 40 Inferior Olive cells (IO), 50 Deep Cerebellar Nuclei (DCN). The connectivity ratios used for the 11 types of synaptic connections are reported in Table 1. Three of these synaptic types could undergo specific plastic modifications, in particular Long Term Potentiation and Depression on different time scales. The numbers of the cells and the connectivity were taken from the neurophysiological literature. The model was tested with a simple closed-loop simulation of the EBCC, to check the functionalities of the network in a learning task [3]. In the EBCC, a Conditioned Stimulus (CS) precedes an Unconditioned Stimulus (US) by a fixed time interval. The cerebellum is able, after repeated presentations of CS and US paired during the acquisition phase, to anticipate the US onset, this action is called Conditioned Response (CR). During the extinction phase, only the CS is provided. The network, thanks to the distributed plasticity, was able to learn the CS-US temporal association during the acquisition trials, with a fast acquisition towards 80% values, and to rapidly unlearn the association during the extinction trials (Figure 1). We will extend this model to a large-scale reproduction of the mouse cerebellum, testing more complex paradigms.

**Table:** **Table 1**. Connectivity between the neural groups (Convergence and Divergence). In italics the plastic sites

Presyn	Postsyn	Type	Conv	Div	# Synapses
MF	Glom	Excitatory	1	20	5000
Glom	GR	Excitatory	4	53	262,400
Glom	GO	Excitatory	40	0.77	4000
GO	GR	Inhibitory	3.23	2120	212,000
GR	GO	Excitatory	2000	3	200,000
*GR*	*PC*	*Excitatory*	*65,600*	*400*	*26,240,000*
IO	PC	Teaching	1	10	400
*PC*	*DCN*	*Inhibitory*	*40*	*5*	*2,000*
DCN (30%)	IO	Inhibitory	0.34	1	14
IO	DCN	Excitatory	1	1,41	50
*MF*	*DCN*	*Excitatory*	*12*	*2.4*	*600*
Total number of synapses					26,926,464


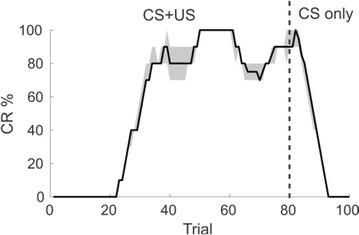


**Figure 1.** Behavioral outcome during the EBCC protocol, with 80 trials of Acquisition and 20 trials of Extinction. 10 simulations were performed. Solid line: the median outcome; grey area: the interquartile intervals

**Acknowledgements**

This work was supported by EU grants: Human Brain Project (HBP 604102) and HBP-Regione Lombardia.

**References**

1. D’Angelo E et al.: Modeling the Cerebellar Microcircuit: New Strategies for a Long-Standing Issue. *Front. Cell. Neurosci.* 2016; **10**:176.

2. Gewaltig MO and Diesmann M: NEST (neural simulation tool). *Scholarpedia* 2007**, 2(4)**:14303.

3. Antonietti et al.: Spiking Neural Network With Distributed Plasticity Reproduces Cerebellar Learning in Eye Blink Conditioning Paradigms. *IEEE Trans. Biomed. Eng.* 2016. **63:**1.210–219.

## P200 Spike-based probabilistic inference with correlated noise

### Ilja Bytschok^1^, Dominik Dold^1^, Johannes Schemmel^1^, Karlheinz Meier^1^, Mihai A. Petrovici^1,2^

#### ^1^Kirchhoff-Institute for Physics, Heidelberg University, Im Neuenheimer Feld 227, 69120 Heidelberg, Germany; ^2^Department of Physiology, University of Bern, Bühlplatz 5, 3012 Bern, Switzerland

##### Correspondence: Ilja Bytschok (ilja.bytschok@kip.uni-heidelberg.de)

*BMC Neuroscience* 2017, **18** (**Suppl 1**):P200

It has long been hypothesized that the trial-to-trial variability in neural activity patterns plays an important role in neural information processing. A steadily increasing body of evidence suggests that the brain performs probabilistic inference to interpret and respond to sensory input [1, 2, 3]. The neural sampling hypothesis [4] interprets stochastic neural activity as sampling from an underlying probability distribution and has been shown to be compatible with biologically observed dynamical regimes of spiking neurons [5]. In these studies, high-frequency Poisson spike trains were used as a source of stochasticity, which is a common way of representing diffuse synaptic input. However, this discounts the fact that cortical neurons may share a significant portion of their presynaptic partners, which can have a profound impact on the computation these neurons are required to perform. This is not only relevant in biology, but also for artificial implementations of neural networks [6], where bandwidth constraints limit the amount of available independent noise channels.

In neural sampling, the firing activity of a network of *N* Leaky Integrate-and-Fire (LIF) neurons is represented by a vector of binary random variables (RVs) *z* ∊ {0, 1}^*N*^. In such a network, synaptic weights can be adjusted such that the network samples from a Boltzmann distribution *p*(*z*) [5]. In particular, the weights *W*_*ij*_ control the pairwise correlations *r*_*ij*_ between RVs. When receiving correlated noise, the correlations *r*_*ij*_ are changed in a way that cannot be directly countered by changes in *W*_*ij*_. We show, however, that this is contingent on the chosen coding: when changing the state space from {0, 1}^*N*^ to {−1, 1}^*N*^, correlated noise has the exact same effect as changes in *W*. Unfortunately, the {−1, 1}-coding is incompatible with neuronal dynamics, because it would require neurons to influence each other while they are silent.

However, the translation of the problem to the {−1, 1}^*N*^ space allows the formulation of a two-step compensation procedure. We show how, by chaining a bijective map from noise correlations to interaction strengths *W*_*ij*_^’^ in {−1, 1}^*N*^ with a second bijective map from (*W*_*ij*_^’^, *b*_*ij*_^’^) in {−1, 1}^*N*^ to (*W*_*ij*_, *b*_*ij*_) in {0, 1}^*N*^ it is possible to find a synaptic weight configuration that compensates for correlations induced by shared noise sources. For an artificial embedding of sampling networks, this allows a straightforward transfer between platforms with different architecture and bandwidth constraints.

Furthermore, the existence of the above mapping provides an important insight for learning. Since in the {−1, 1}-coding the correlated noise can be compensated by parameter changes and because the {−1, 1}-coding can be transformed into a {0, 1}-coding while keeping the state probabilities invariant, a learning rule for Boltzmann machines will also find that distribution in the {0, 1}-coding, which we demonstrate in software simulations. In other words, spiking networks performing neural sampling are impervious to noise correlations when appropriately trained. This means that, if such computation happens in cortex, network plasticity does not need to take particular account of shared noise inputs.

**Acknowledgements**

Authors Bytschok, Dold and Petrovici contributed equally to this work. This research was supported by EU grants #269921 (BrainScaleS), #604102 (Human Brain Project) and the Manfred Stärk Foundation.

**References**

1. Körding K, Wolpert D: Bayesian integration in sensorimotor learning. *Nature* 2004

2. Fizser J, Berkes P, Orbán G, Lengyel M: Statistically optimal perception and learning: from behavior to neural representations. *Trends in Cognitive Sciences* 2010

3. Rich EL, Wallis JD: Decoding subjective decisions from orbitofrontal cortex. *Nature Neuroscience* 2016

4. Buesing L, Bill J, Nessler B, Maass W: Neural dynamics as sampling: a model for stochastic computation in recurrent networks of spiking neurons. *PLoS Comput Biol* 2011

5. Petrovici MA, Bill J, Bytschok I, Schemmel J, Meier K: Stochastic inference with spiking neurons in the high-conductance state. *Physical Review E* 2016

6. Furber S: Large-scale neuromorphic computing systems. *Journal of Neural Engineering* 2016

## P201 Optimal refractoriness from a rate-distortion perspective

### Hui-An Shen, Simone Carlo Surace, Jean-Pascal Pfister

#### Institute of Neuroinformatics, UZH and ETHZ, Zurich, CH-8057, Switzerland

##### Correspondence: Jean-Pascal Pfister (jpfister@ini.uzh.ch)

*BMC Neuroscience* 2017, **18** (**Suppl 1**):P201

The information transfer from neuron to neuron through chemical synapses undergoes two stages. In the presynaptic neuron, the (analog) membrane potential is encoded into a (digital) spike while in the postsynaptic neuron, this digital information is turned back into an (analog) depolarisation. It has been argued that for a given inhomogeneous Poisson encoder, the optimal decoder has dynamics that is consistent with short-term plasticity [1]. However, the optimal encoder is not known. Here, by studying the rate-distortion performance, we explore how presynaptic refractoriness influences the performance of the optimal postsynaptic decoder. First, we generalize the results of [2] and [3] by expressing the mutual information as a function of the mean *natural estimation loss*, in the presence of refractoriness. This expression provides a numerically stable and fast method of computing mutual information between two high-dimensional random variables. Next, we show with numerical simulations that for a fixed firing rate ranging from 20-120 Hz, there is an optimal level of refractoriness that minimizes the distortion, i.e. the mean squared error of the optimal postsynaptic decoder. To test our theory, we compare this optimal level of refractoriness with an HVC neuron in Zebra Finch to which the model has been fitted [4].

**References**

1. Pfister JP, Dayan P, Lengyel M: Synapses with short-term plasticity are optimal estimators of presynaptic membrane potentials. *Nat Neurosci.* 2010, **13(10):**1271–1275.

2. Atar R, Weissman T: Mutual information, relative entropy, and estimation in the Poisson channel. *IEEE Transactions on Information theory* 2012, **58(3):**1302–1318.

3. Liptser RS, Shiryaev AN: *Statistics of Random Processes II, 2nd Edition.* New York: Springer-Verlag; 2001.

4. Surace SC, Pfister JP: A statistical model for in vivo neuronal dynamics. *PloS one* 2015, **10(11):**e0142435.

## P202 Towards online accurate spike sorting for hundreds of channels

### Baptiste Lefebvre,, Marcel Stimberg, Olivier Marre, Pierre Yger

#### Institut de la Vision, INSERM UMRS 968, CNRS UMR 7210, Paris, France

##### Correspondence: Pierre Yger (pierre.yger@inserm.com)

*BMC Neuroscience* 2017, **18** (**Suppl 1**):P202

Understanding how assemblies of neurons encode information requires recording of large populations of cells in the brain. In recent years, multi-electrode arrays and large silicon probes have been developed to record simultaneously from thousands of electrodes packed with a high density. To tackle the fact that these new devices challenge the classical way to perform spike sorting, we recently developed a fast and accurate spike sorting algorithm (available as an open source software, called SpyKING CIRCUS), validated both with in vivo and in vitro ground truth experiments [1]. The software, performing a smart clustering of the spike waveforms followed by a greedy template-matching reconstruction of the signal, is able to scale to up to 4225 channels in parallel, solving the problem of temporally overlapping spikes. It thus appears as a general solution to sort, offline, spikes from large-scale extracellular recordings.

In this work, we aim at implementing this algorithm in an “online” mode, sorting spikes in real time while the data are acquired, to allow closed-loop experiments for high density electrophysiology. To achieve such a goal, we built a robust architecture for distributed asynchronous computations and we propose a modified algorithm that is composed of two concurrent processes running continuously: 1) “a template-finding” process to extract the cell templates (i.e. the pattern of activity evoked over many electrodes when one neuron fires an action potential) over the recent time course; 2) a “template-matching” process where the templates are matched onto the raw data to identify the spikes. The main challenge is to have a continuous update of the set of templates, with hundreds of electrodes and possible drifts over the time course of the experiment. A key advantage of our implementation is to be parallelized over a computing cluster to use optimally the computing resources: all the different processing steps of the algorithms (whitening, filtering, spike detection, template identification and fit) can be distributed according to the computational needs. During the clustering, the most computationally demanding step, templates are detected and tracked over time using a modified version of the density based clustering algorithm [2] able to handle data streams. Our software is therefore a promising solution for future closed-loop experiments involving recordings with hundreds of electrodes.

**References**

1. P. Yger et al., Fast and accurate spike sorting in vitro and in vivo for up to thousands of electrodes, *BioRxiv* 2016.

2. A. Rodriguez et al., Clustering by fast search and find of density peaks, *Science* 2014.

## P203 Modeling orientation preference in the apical and basal trees of L2/3 V1 neurons

### Athanasia Papoutsi^1^, Jiyoung Park^2^, Ryan Ash^2^, Stelios Smirnakis^2^, Panayiota Poirazi^1^

#### ^1^IMBB, FORTH, Heraklion, Crete, 70013, Greece; ^2^Neurology, Baylor College of Medicine, Houston, Texas, 77030, USA

##### Correspondence: Athanasia Papoutsi (athpapoutsi@gmail.com)

*BMC Neuroscience* 2017, **18** (**Suppl 1**):P203

Pyramidal neurons receive inputs in two anatomically and functional distinct domains [1], the apical and the basal tree. Inputs to the basal tree, due to their proximity to the soma, greatly influence neuronal output, whereas the more remote apical tree has less potential to influence somatic activity. How these inputs co-operate to form the functional output of the neurons is currently unknown. In this work, we focused on how inputs to the apical and basal trees shape orientation tuning in L2/3 V1 neurons. In particular, we investigated how dendritic integration of orientation tuned inputs to the apical versus basal trees allows for the emergence of stable neuronal orientation preference. Towards this goal, a model L2/3 V1 pyramidal neuron was implemented in the NEURON simulation environment. The passive and active properties of the model neuron were extensively validated against experimental data. Synaptic properties, number and distribution were also constrained according to available data (Figure 1A). Using this model neuron, we investigated a) the differences in the mean orientation preferences of the two trees and b) the distribution of orientation preferences to individual synapses that allow for the emergence of orientation tuning (Figure 1B). Given the parameter combinations that allow for the emergence of orientation tuning (Figure 1C), we found that neuronal orientation tuning follows in large part the orientation tuning of the basal tree. In addition, we have further identified how apical versus basal dendritic tree ablation would affect neuronal tuning in the different conditions implemented. Model results provide insights regarding the ‘tolerance’ to different input properties at the apical and basal tree in order to achieve stable orientation preference.
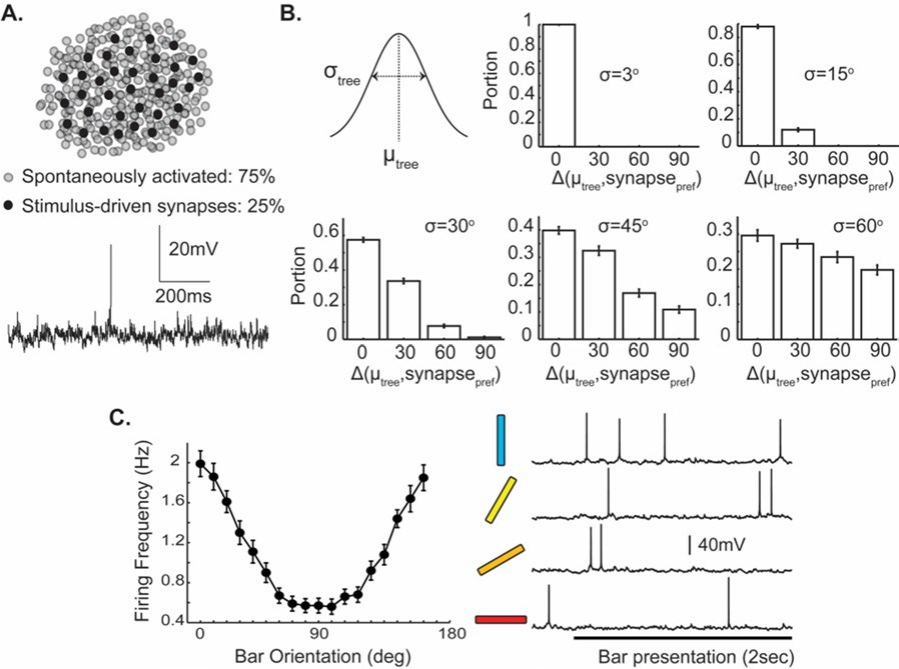


**Figure 1. A.** Top: From the pool of synapses, 25% were stimulus driven (black dots). Bottom: Indicative trace showing fluctuations of the membrane potential in the presence of background synaptic activity. Spikes are truncated for visualization purposes. **B.** Each tree was characterized by a μ ± σ orientation preference that was determined by the preferences of the individual synapses. Here it is shown the portion of synapses with same/different orientation preferences from the μ_tree_, for σ_tree_ = 3, 15, 30, 45 and 60°. Grouping to the reported value (x-axis) includes ± 10° differences. **C.** Orientation tuning curve of the model neuron (mean ff ± sem). Right: Indicative voltage traces of the neuronal responses for different bar orientations (0°, 30°, 60° and 90°)

**Reference**

1. Larkum ME: A cellular mechanism for cortical associations: an organizing principle for the cerebral cortex. *Trends Neurosci* 2012:1–11.

## P204 Dual recordings in the mouse auditory brainstem and midbrain reveal differences in the processing of vocalizations

### Richard A. Felix^1^, Alexander G. Dimitrov^1,2^, Christine Portfors^1^

#### ^1^Department of Integrative Biology and Neuroscience, Washington State University Vancouver, Vancouver WA 98686, USA; ^2^Department of Mathematics and Statistics, Washington State University Vancouver, Vancouver WA 98686, USA

##### Correspondence: Alexander G. Dimitrov (alex.dimitrov@wsu.edu)

*BMC Neuroscience* 2017, **18** (**Suppl 1**):P204

**Background:** A normal functioning auditory system must rely on fast and precise neuronal responses in order to accurately represent temporal information in complex sounds. Impairments in temporal processing contribute to a variety of listening disorders, yet our understanding of mechanisms that govern these processes remains limited. We examined how enhanced spike timing at the level of the inferior colliculus (IC) in the midbrain might underlie efficient encoding of vocalizations compared to the cochlear nucleus (CN), an earlier site in the ascending auditory pathway.

**Methods:** We recorded neuronal responses to conspecific vocalizations in the IC and CN of awake, normal-hearing mice that expressed Channelrhodopsin in VGlut2-positive neurons. We used an optrode that combined the recording of single unit activity with light delivery to the CN. Once a recording was established in the CN, a second electrode was placed in the IC and dual recordings were established at locations with matching frequency tuning. The CN was stimulated with light in the absence of sound to measure effects in the IC and then responses to sound stimuli were simultaneously recorded at each site. We assessed the extent of functional connectivity between CN and IC recording sites, the temporal precision of evoked spiking, and the neuronal selectivity to vocalization stimuli, using statistical and information-theoretic tools.

**Results:** We found that stimulating the CN with light caused evoked activity in the IC when the two recording sites had matched frequency tuning, suggesting that tonotopic organization reliably predicts functional connectivity between the sites. Despite matching frequency tuning, IC neurons exhibited greater selectivity to a common set of vocalization stimuli compared to the dorsal CN (DCN). Overall, CN responses had higher rates of evoked spiking, while IC responses were more transient and had enhanced spike timing, suggesting a shift toward the extraction of temporal information contained in vocalizations at the level of the midbrain (Figure 1).
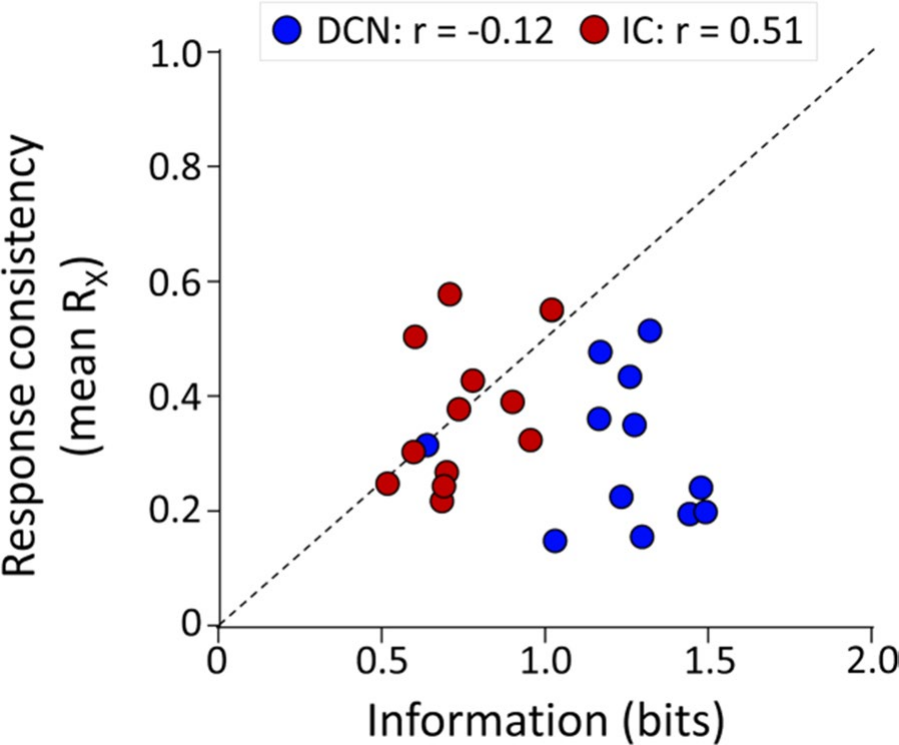


**Figure 1.** Relationship between information content and response consistency in mouse DCN and IC

**Conclusion:** Neurons in the CN often contributed to activity recorded in the IC. Dual recordings conducted under the same experimental conditions that have a degree of functional connectivity provide a strong paradigm for comparing processing at different stages of the auditory pathway. Enhanced selectivity to vocalizations and temporal precision of responses in the IC suggests that this region may be important for encoding biologically important sounds. When auditory processing is impaired, the IC may be a subcortical site for the generation of auditory disorders typically thought to arise in the cortex.

## P205 Modelling of leg decoupling in the stick insect and its possible significance for understanding the workings of the locomotor system

### Silvia Daun^1,2^, Tibor I. Toth^1^

#### ^1^Department of Animal Physiology, Institute of Zoology, University of Cologne, Cologne, 50674, Germany; ^2^Cognitive Neuroscience, Institute of Neuroscience and Medicine (INM-3), Research Center Juelich, Juelich, 52425, Germany

##### Correspondence: Silvia Daun (Silvia.Daun@uni-koeln.de)

*BMC Neuroscience* 2017, **18** (**Suppl 1**):P205

Amputation and temporary restraint of legs are widely used and accepted methods of the study of the locomotor systems of insects. The animal is studied during free walking, and its walking behaviour is compared before and after the amputation. Using the results, conclusions are drawn with regard to the organization of the locomotor system of the animal in question. In the stick insect, such investigations were carried out by [1] and more recently by [2]. In the latter study, it was even observed that the front legs could reversibly be decoupled by the animal itself and used to carry out search movements. Nevertheless, the hind and middle legs continued their coordinated walking. From these and other experimental observations detailed in [1] and [2], the question naturally arising is: what mechanisms underlie the changes found in the experiments. The underlying mechanisms obviously belong to the part of the nervous system that controls and coordinates locomotion. One promising way to study them is by using appropriate mathematical models. We used an existing model of coordinated stepping of the three ipsilateral legs of the stick insect [3] to mimic the various decoupling situations in the stick insect described in [1] and [2]. In the model, the levator-depressor neuro-muscular control networks (LD systems) of the individual legs play a pivotal role in producing coordinated stepping of the legs. We identified three main possibilities of decoupling a single leg: i) disrupting the inter-leg coordination between the legs’ LD systems; ii) blocking the normal function of the central pattern generator of the LD system of the leg to be decoupled; and iii) changing the activity of the levator and depressor motoneurones via their associated pre-motor inhibitory interneurones. Decoupling of the front leg in the model worked with any of the methods i)-iii). It was easily reversible, in accordance with the observations that such reversible decoupling happens in natural conditions when the animal uses its front legs for searching. The hind and middle leg continued their coordinated stepping, like in the experiments [1, 2]. Decoupling of the hind leg was most effective when method iii) was used. In this case, the middle and the front leg continued performing coordinated stepping irrespective of the decoupling method, in agreement with the experimental findings. In the model, the middle leg took over automatically the role of the hind leg as the origin of the coordinated stepping. Decoupling the middle leg yielded mixed results: in some cases, depending on the phase within a stepping period, the coordinated stepping of the front and hind leg was abolished, in others, it was not but its quantitative properties were changed. Both types of results were also found in the experiments [1, 2].

In conclusion, we suggest that, depending on the leg, various mechanisms are possible to decouple it from the system of inter-leg coordination. In all cases, method iii) worked most reliably and efficiently. However, the other mechanisms (methods) may represent redundance and can be activated, if necessary, to bring about decoupling of the leg.

**Acknowledgements**

This work was supported by the DFG grants to S. Daun (GR3690/2-1 and GR3690/4-1).

**References**

1. Graham D: The effect of amputation and leg restraint on the free walking coordination of the stick insect Carausius Morosus. J Comp Physiol 1977, **116:**91–116.

2. Grabowska M, Godlewska E, Schmidt J, Daun-Gruhn S: Quadrupedal gaits in hexapod animals - inter-leg coordination in free walking adult stick insects. J Exp Biol 2012, **215:**4255–4266.

3. Toth TI, Daun-Gruhn S: A three-leg model producing tetrapod and tripod coordination patterns of ipsilateral legs in the stick insect. J Neurophysiol 2016, **115:**887–906.

## P206 Spatio-temporal dynamics of key signaling molecules in growth cones

### Joanna Jędrzejewska-Szmek^1^, Nadine Kabbani^1,2^, Kim T. Blackwel^1,3^

#### ^1^Krasnow Institute, George Mason University, Fairfax, VA 22030, USA; ^2^School of Systems Biology, George Mason University, Fairfax, VA 22030, USA; ^3^Bioengineering Department, George Mason University, Fairfax, VA 22030, USA

##### Correspondence: Joanna Jędrzejewska-Szmek (jjedrzej@gmu.edu)

*BMC Neuroscience* 2017, **18** (**Suppl 1**):P206

Growth cones, guided by environmental cues, are necessary for proper neural functioning. The cues are detected by membrane-bound receptors, which in turn activate a plethora of signaling pathways. A majority of these pathways is governed by calcium, flowing into the growth cone through the plasmalemma or from the calcium stores. Both the magnitude of calcium increase and identity of calcium source seem to determine neural growth and retraction [1]. Calcium exerts its control through a variety of signaling molecules that interact non-linearly. This picture is further complicated by recent findings showing that the ionotropic alpha7 nicotinic receptor (a7nAChR) also has a metabotropic function and couples to heteromeric Gq proteins. A7nAChR action via the Gq pathway results in calcium release from the endoplasmic reticulum (ER) modulating cytoskeletal motility and structural growth [2–4].

Experimental evidence shows that both low and high cytosolic calcium results in growth cone repulsion, and medium cytosolic calcium results in attraction. It also shows that calcium influx through the plasmalemma results in repulsion and calcium influx from the internal stores results in growth. To investigate and unify these seemingly contradictory observations experimental observations, we developed a stochastic reaction-diffusion model of calcium, cAMP and Gq activated pathways. The model allows for evaluating the role of the transient calcium influx through the channel pore (the ionotropic contribution) compared to the role of calcium release caused by activation of the Gq subtype of GTP binding protein. Using the model, we investigated whether combined metabotropic and ionotropic action of a7nAChR, resulting in prolonged increase of cytosolic calcium, is responsible for experimentally observed growth attenuation.

To test whether we can predict neurite outgrowth and retraction in response to various environmental stimuli and to elucidate contribution of molecular gradients we looked at combined action of key signaling molecules. We show that combined activation of calcium and cAMP activated targets such as PP2B and PP1, CaMKII, PKA and calpain can explain the non-monotonic dependence of structural growth on calcium levels. Elucidating the mechanisms underlying synaptic growth will allow for better understanding of mechanisms of neural development and regeneration

**Acknowledgements**

The joint NIH-NSF CRCNS program through NSF grant 1515686

**References**

1. Henley J, Poo M-m: Guiding neuronal growth cones using Ca^2+^ signals. *Trends in Cell Biol* 2004, **14**:320–330. doi: 10.1016/j.tcb.2004.04.006

2. Nordman JC, Kabbani N: Microtubule dynamics at the growth cone are mediated by α7 nicotinic receptor activation of a Gαq and IP3 receptor pathway. *FASEB J* 2014, **28**:2995–3006. doi: 10.1096/fj.14-251439.

3. King JR, Nordman JC, Bridges SP, Lin MK, Kabbani N. Identification and characterization of a G protein-binding cluster in α7 nicotinic acetylcholine receptors. *J Biol Chem* 2015, 290:20060–70. doi:10.1074/jbc.M115.647040

4. King JR, Kabbani N: Alpha 7 nicotinic receptor coupling to heterotrimeric G proteins modulates RhoA activation, cytoskeletal motility, and structural growth. *J Neurochem* 2016, **138**:532–45. doi:10.1111/jnc.13660.

## P207 A simulation of EMG signal generation following TMS

### Bahar Moezzi^1,2^, Natalie Schaworonkow^3^, Lukas Plogmacher^3^, Mitchell R. Goldsworthy^2,4^, Brenton Hordacre^2^, Mark D. McDonnell^1^, Nicolangelo Iannella^1,5^, Michael C. Ridding^2^, Jochen Triesch^3^

#### ^1^Computational and Theoretical Neuroscience Laboratory, School of Information Technology and Mathematical Sciences, University of South Australia, Adelaide, Australia; ^2^Robinson Research Institute, School of Medicine, University of Adelaide, Adelaide, Australia; ^3^Frankfurt Institute for Advanced Studies, Frankfurt, Germany; ^4^Discipline of Psychiatry, School of Medicine, University of Adelaide, Adelaide, Australia; ^5^School of Mathematical Sciences, University of Nottingham, Nottingham, UK

##### Correspondence: Bahar Moezzi (bahar.moezzi@mymail.unisa.edu.au)

*BMC Neuroscience* 2017, **18** (**Suppl 1**):P207

Transcranial magnetic stimulation (TMS) is a technique that allows noninvasive manipulation of neural activity and is used extensively in both clinical and basic research settings [1]. The effect of TMS on motor cortex is often measured by electromyography (EMG) recordings from a small hand muscle, such as the first dorsal interosseous (FDI). However, the details of how TMS generates responses measured with EMG are not completely understood. Here, we aim to develop a biophysically detailed computational model to study the potential mechanisms underlying the generation of EMG signals in response to TMS.

Our model comprises a feed-forward network of cortical layer 2/3 cells, which drive morphologically detailed layer 5 corticomotoneuronal cells based on [2]. The cortical layer 5 cells in turn project to a pool of motoneurons and eventually the muscle. The EMG signal is the sum of motor unit action potentials. Model parameters are tuned to match results from EMG recordings from the FDI muscle performed in four human subjects.

The model successfully reproduces several properties of the experimental data. The simulated EMG signals match experimental EMG recordings in shape and size, and vary with stimulus and contraction intensities as in experimental data. They exhibit cortical silent periods that are close to the biological values, and reveal an interesting dependence on inhibitory synaptic transmission characteristics. Our model predicts neural firing patterns along the entire pathway from cortical layer 2/3 cells down to spinal motoneurons. In conclusion, our model successfully reproduces major features of EMG recordings and should be considered as a viable tool for analyzing and explaining EMG signals following TMS.

**References**

1. Hallett M: Transcranial magnetic stimulation and the human brain. Nature 2000, **406**:147–150.

2. Rusu CV, Murakami M, Ziemann U, Triesch J. A model of TMS-induced I-waves in motor cortex. *Brain Stimul* 2014, **7**:401–414.

## P208 The effect of LTP, LTD and non-specific LTD on the Recognition of Sparse Noisy Patterns in Simplified and Detailed Purkinje Cell Models

### Reinoud Maex^1^, Karen Safaryan^2^, Volker Steuber^3^

#### ^1^Department of Cognitive Sciences, Ecole Normale Supérieure, rue d’Ulm 25, 75005 Paris, France; ^2^Department of Physics and Astronomy, Knudsen Hall, University of California, Los Angeles, CA, 90095-0001, USA; ^3^Centre for Computer Science and Informatics Research, University of Hertfordshire, College Lane, Hatfield, AL10 9AB, United Kingdom

##### Correspondence: Reinoud Maex (reinoud.maex@ens.fr)

*BMC Neuroscience* 2017, **18** (**Suppl 1**):P208

Classic theories of cerebellar learning suggest that parallel fibre (PF) activity patterns in cerebellar cortex can be stored and recalled based on long-term depression (LTD) of PF - Purkinje cell synapses [1, 2]. As in other theories of learning in neural systems, it is commonly assumed that the weight changes are limited to activated synapses. However, it has been shown that a non-specific form of PF LTD can spread to neighbouring synapses that are inactive during learning [3]. Moreover, long-term potentiation (LTP) of PF synapses has also been found to contribute to cerebellar learning [4].

We have previously studied the effect of non-specific LTD (nsLTD) on pattern recognition and have shown that nsLTD can provide robustness against local spatial noise in the input patterns [5]. Here we extend our previous work by studying the functional role of LTP, and we investigate other determinants of the pattern recognition performance such as the sparsity and number of patterns and different types of pattern noise. We compare results from numerical simulations of a morphologically realistic conductance based Purkinje cell model (as in [2]) with those of a simple linear artificial neural network (ANN) unit. Further, to better understand the results of the numerical simulations, we perform a mathematical analysis of the pattern recognition performance of the ANN unit. As in previous work, we quantify the pattern recognition performance by calculating a signal-to-noise (s/n) ratio [2, 5].

The simulations and analysis of the ANN unit predict that adding LTP to the learning rule does not affect the pattern recognition performance, given that the mean and variance of responses, which appear in the enumerator and denominator of the s/n ratio, respectively, are equally affected by LTP. In contrast, however, the pattern recognition performance of the Purkinje cell model was sensitive to the average synaptic weight, which determined both the spontaneous spike rate and the response to pattern presentation. Adding LTP in the Purkinje cell model made nsLTD equivalent or superior to LTD at all noise levels. Moreover, the LTP based normalisation of weights prevented the Purkinje cell responses from becoming too weak and increased the number of patterns that could be stored for a given s/n ratio by a factor of 4. Finally, we show that our previous conclusions hold over a large range of pattern loadings and sparsities, and that local additive pattern noise can further increase the beneficial effect of nsLTD.

**References**

1. Marr D: A theory of cerebellar cortex. *J Physiol* 1969, **202:**437–470.

2. Steuber V, Mittmann W, Hoebeek FE, Silver RA, De Zeeuw CI, Hausser M, De Schutter E: Cerebellar LTD and pattern recognition by Purkinje cells. *Neuron* 2007, **54:**121–136.

3. Wang SS, Khiroug L, Augustine GJ: Quantification of spread of cerebellar long-term depression with chemical two-photon uncaging of glutamate. *Proc Natl Acad Sci USA* 2000, **97:**8635–8640.

4. Schonewille M, Belmeguenai A, Koekkoek SK, Houtman SH, Boele HJ, van Beugen BJ, Gao Z, Badura A, Ohtsuki G, Amerika WE, Hosy E, Hoebeek FE, Elgersma Y, Hansel C, De Zeeuw CI: Purkinje cell-specific knockout of the protein phosphatase PP2B impairs potentiation and cerebellar motor learning. *Neuron* 2010, **67:**618–628.

5. Safaryan K, Maex R, Adams RG, Davey N, Steuber V: Non-specific LTD at parallel fibre - Purkinje cell synapses in cerebellar cortex provides robustness against local spatial noise during pattern recognition. *BMC Neuroscience* 2011, **12:**P314.

## P209 Modeling causality of the smoking brain

### Rongxiang Tang^1^, Yi-Yuan Tang^2^

#### ^1^Department of Psychology, Washington University in St. Louis, St. Louis, MO 63130, USA; ^2^Department of Psychological Sciences, Texas Tech University, TX 79409, USA

##### Correspondence: Yi-Yuan Tang (yiyuan.tang@ttu.edu)

*BMC Neuroscience* 2017, **18** (**Suppl 1**):P209

Previous studies indicated that brain areas including prefrontal cortex (e.g., medial prefrontal cortex, mPFC), posterior cingulate cortex (PCC) and insula involved in smoking addiction [1]. However, functional connectivity among these regions only shows the correlative relationship but does not reveal the causal relationship such as the changes in information flow in these distributed brain areas involved in smoking. In prior studies [2-3], we applied a newly developed spectral dynamic causal modeling (spDCM) to resting state fMRI to demonstrate the causal relationships among the core regions in smoking addiction. Our results suggested that compared to nonsmokers, smokers had reduced effective connectivity from PCC to mPFC and from right inferior parietal lobule (R-IPL) to mPFC, a higher self-inhibition within PCC and a reduction in the amplitude of endogenous neuronal fluctuations driving the mPFC [2]. Given that Granger causality (GC) and DCM are two main causality methods and have distinct but complementary ambitions that are usefully considered in relation to the *detection* of functional connectivity and the *identification* of models of effective connectivity [4-5], therefore it’s important to use a same dataset to compare two models.

We used the dataset of college students previously reported in our study [2]. All fMRI data were collected using a 3-Telsa Siemens Skyra scanner and processed using the Data Processing Assistant for Resting-State fMRI, which is based on SPM and Resting-State fMRI Data Analysis Toolkit [2-3]. For fMRI analyses, we conducted the standard procedures included slice timing, motion correction, regression of WM/CSF signals and spatial normalization [3]. A standard GC analysis was also applied to test the causality among key regions involved in smoking [5-6]. Based on previous literature, in this study we specified four regions of interest within default mode network (DMN) - medial prefrontal cortex (mPFC), posterior cingulate cortex (PCC), and bilateral inferior parietal lobule (Left IPL and Right IPL), same coordinates as in previous spDCM studies [2]. Our results showed the similar causal relationship among these brain areas.

**Conclusions:** GC and DCM are complementary: both are concerned with directed causal interactions. GC models dependency among observed responses, while DCM models coupling among the hidden states generating observations. Despite this fundamental difference, the two approaches may be converging.

**Acknowledgements**

This work was supported by the Office of Naval Research.

**References**

1. Goldstein RZ, Volkow ND: Dysfunction of the prefrontal cortex in addiction: Neuroimaging findings and clinical implications. *Nat Rev Neurosci* 2011, **12**:652–669.

2. Tang R, Razi A, Friston KJ, Tang YY: Mapping smoking addiction using effective connectivity analysis. *Frontiers in Human Neuroscience.* 2016, **10**:195.

3. Razi A, Kahan J, Rees G, Friston KJ: Construct validation of a DCM for resting state fMRI. *Neuroimage* 2015, **106**:1–14.

4. Friston K, Moran R, Seth AK: Analysing connectivity with Granger causality and dynamic causal modelling. *Curr Opin Neurobiol.* 2013, **23**:172–8.

5. Seth AK: A MATLAB toolbox for Granger causal connectivity analysis. *J Neurosci Meth* 2010, **186**:262–273.

6. Zhao Z, Wang X, Fan M, Yin D, Sun L, Jia J, Tang C, Zheng X, Jiang Y, Wu J, Gong J: Altered effective connectivity of the primary motor cortex in stroke: a resting-state fmri study with Granger causality analysis. *PLoS One.* 2016, **11**:e0166210.

## P210 Modelling of calcium waves in astrocytic networks induced by neural activity

### Darya V. Verveyko^1^, Alexey R. Brazhe^2^, Andrey Yu Verisokin^1^, Dmitry E. Postnov^3^

#### ^1^Department of Theoretical Physics, Kursk State University, Kursk, 305000, Russian Federation; ^2^Department of Biophysics, Lomonosov Moscow State University, Moscow, 119991, Russian Federation; ^3^Department of Physics, Saratov State National Research University, Saratov, 410012, Russian Federation

##### Correspondence: Darya V. Verveyko (allegroform@mail.ru)

*BMC Neuroscience* 2017, **18** (**Suppl 1**):P210

We propose two-compartment model of calcium dynamics in astrocyte network, basing on Ullah model [1]. In order to count the specific features of different parts of astrocyte network we mark out three types of modelling space: astrocyte soma with thick branches, thin branches, and extracellular space. We have developed two variants of equation set which are different in relative contribution of specific ionic currents. We suppose that activation of astrocyte calcium dynamics is mediated by the extracellular space, specifically, via diffusion of synaptic glutamate released due to the neuronal activity, which we describe as some random signal incorporating noise effects.

We have performed a number of simulation runs at different parameter sets for individual astrocyte and multi-cell network. One of simulation examples within the computational multi-cell template is given in Figure 1. The global wave emerging in one of the points passes through the wide region of astrocyte network. The formation of the wave has a high degree of regularity and periodicity. There are also local regimes where excitation waves damp passing through a small number of cells.
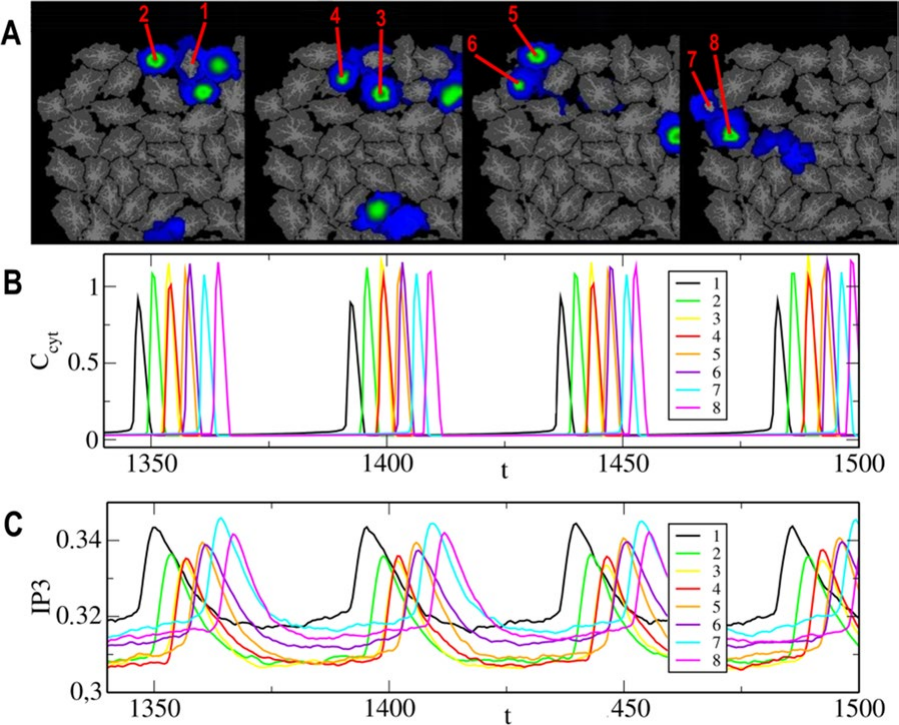


**Figure 1.** Calcium global wave in multi-cell ensemble. **A.** The representative snapshots of spatial patterns. Numbers from 1 to 8 indicate the cells according to its involvement in firing pattern. **B, C.** The time courses of cytosolic Ca^2+^ and IP_3_ concentrations, respectively

**Conclusions:** We have suggested the advanced model of astrocyte network dynamics, which fits well the recent experimental findings [2]. Specifically, we have suggested the development of model equations for intra-astrocyte calcium dynamics, which takes into account its specific topological features. We have tested the suggested approach for both individual cell image and multi-cellular structure. The obtained results confirm that our model is able to reproduce the evolution of spatio-temporal dynamics under neuronal activity represented by spatially uncorrelated and randomized in time process of glutamate injection. In multicellular system, a persistent self-organized rhythmicity of calcium activity in groups was found which can be explained by some interplay between the refractory time of calcium excitability and noise-triggered processes.

**Acknowledgements**

This work is partially supported by the Ministry of Education and Science of the Russian Federation within the research project №3.9499.2017 included into the basic part of research funding assigned to Kursk State University.

**References**

1. G. Ullah, P. Jung, A.H. Cornell-Bell: Anti-phase calcium oscillations in astrocytes via inositol (1, 4, 5)-trisphosphate regeneration. *Cell Calcium* 2006, **39**: 197–208.

2. M. Falcke: Reading the patterns in living cells - the Physics of Ca^2+^ signaling. *Adv. in Phys.* 2004, **53(3):** 255–44

## P211 Simulated voltage clamp: offline biophysical reconstruction of fast ionic currents in large cells with uncompensated series resistance

### Cengiz Günay^1,2^, Gabriella Panuccio^3^, Michele Giugliano^3^, Astrid A. Prinz^1^

#### ^1^Dept. Biology, Emory University, Atlanta, Georgia 30322, USA; ^2^School of Science and Technology, Georgia Gwinnett College, Lawrenceville, Georgia 30043, USA; ^3^Theoretical Neurobiology & Neuroengineering Lab, Dept. Biomedical Sciences, University of Antwerp, Antwerp, Belgium

##### Correspondence: Cengiz Günay (cgunay@ggc.edu)

*BMC Neuroscience* 2017, **18** (**Suppl 1**):P211

Characterization of ion channel kinetics from voltage-clamp experiments is inherently biased by the non-linear voltage error introduced by the resistance of the recording pipette in series with the membrane resistance (series resistance, Rs) [1]. Modern patch-clamp amplifiers provide built-in circuits for on-line Rs compensation. However, because of the nature of these circuits, it is theoretically impossible to achieve 100% Rs compensation without losing stability of the recording. Moreover, fast ionic voltage-dependent currents, like sodium (Na^+^) currents, require a high band-width operation of the Rs compensation circuit, which in turn might result in sudden oscillations of the cell membrane voltage (Vm). Consequently, Rs compensation is currently a trade-off between a commonly accepted error tolerance and the crucial need for preventing oscillations. Here, we build a novel “simulation method” as a new component to a previously developed computational framework [2] to overcome these limitations. In contrast to the amplifier’s strategy to force a flat voltage waveform, which is required for generating conventional current-voltage plots of peak ionic currents, we allow arbitrary voltage waveforms by simulating voltage-clamp in a computational neuron model and then curve fitting its output to match recordings to directly estimate Hodgkin-Huxley model parameters of the channel. The kinetics parameters so obtained are used to reconstruct the unbiased current trace. We demonstrate our method using voltage-clamp recordings of Na^+^ currents from ‘giant’ layer V pyramidal cells of the rat primary somatosensory cortex in the presence of uncompensated, significantly high (10-20 MΩ) Rs along with the low input resistance (~40 MΩ) typical of these cells, so as to maximize the compound voltage clamp errors. As shown in Figure 1, the model computes non-linear artifact currents and predicted actual Vm values. When Rs compensation is a major concern for the reliability voltage-clamp data, our approach is capable of overcoming the limitations posed by currently available hardware- and software-based Rs compensation methods, thus allowing to fully reconstructing the actual current kinetics.
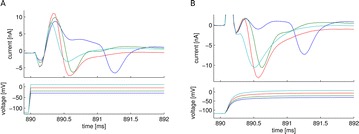


**Figure 1.** Offline subtraction of estimated amplifier-unaccounted passive currents. **A.** Raw recordings of Na^+^ currents contaminated by uncompensated artifacts (top) recorded during the corresponding voltage steps (bottom trace). **B.** Passive artifacts subtracted from the current traces (top) and actual Vm (bottom) estimated using the model simulation method. Note how the actual Vm differs significantly from the desired holding voltage-steps (see panel A)

**Acknowledgements**

Career Award at the Scientific Interface (CASI) from the Burroughs Wellcome Fund awarded to AAP.

**References**

1. Sakmann, B., and Neher, E. *Single*-*Channel Recording. 2nd Edition*, (Springer Science & Business Media, Plenum Press, New York, 1995).

2. Günay C, Edgerton JR, Li S, Sangrey T, Prinz AA, and Jaeger D. Database analysis of simulated and recorded electrophysiological datasets with PANDORA’s toolbox. *Neuroinformatics* 2009, **7:** 93–111.

## P212 Representing and implementing cognitive sequential interactions

### Pablo Varona^1^, Mikhail I. Rabinovich^2^

#### ^1^Grupo de Neurocomputación Biológica, Dpto. de Ingeniería Informática, Escuela Politécnica Superior, Universidad Autónoma de Madrid, Madrid, Spain; ^2^BioCircuits Institute, University of California, San Diego, CA, USA

##### Correspondence: Pablo Varona (pablo.varona@uam.es)

*BMC Neuroscience* 2017, **18** (**Suppl 1**):P212

Cognition as observed by imaging experiments involves sequential activations of different brain regions [1]. The sequential nature of most aspects of cognition is also reflected in the progression of successive components of decision-making and behavior. In this work, we present a family of models that describe hierarchical relationships among cognitive processes represented with robust sequential dynamics. These models build heteroclinic networks based on the winnerless competition principle where asymmetric inhibition shapes key properties for sequential information processing. The robustness of the sequential dynamics in these networks relies on stable heteroclinic channels, sequences of metastable states and their vicinity connected by separatrices that link them in a chain.

The models described in this work are implemented with generalized Lotka-Volterra equations whose variables can represent information perception items and also cognitive resources such as attention, working-memory and emotion [2–5]. Their hierarchical interactions give rise to binding and chunking processes. We discuss applications of these models in three different contexts: (i) the characterization of decision-making in terms of the sequential evolution of incoming information and the hierarchical organization of cognitive resources in time; (ii) the use of these models to build joint robot-human interactions which result in an increased joint creativity of such team; (iii) the use of these models to drive closed-loop stimulation in novel experiments to reveal healthy and pathological dynamics of cognitive processes in normal subjects and in subjects with cognitive impairments. The considered dissipative models are in general structurally stable and suitable for bifurcation analysis, which helps their interpretation in relationship with experimental data. Their robustness and computational efficiency also make them adequate for real-time implementations in the proposed applications.

Overall, we stress the need to interpret brain imaging experiments in the context of theoretical studies that describe information flows corresponding to sequential cognitive processes. The coarse-grained information of current imaging techniques can be matched to the variables represented in the proposed network models. The results of such analyses can lead to novel insights linking networks graphs to cognitive dynamics, and the development of novel technology for rehabilitation purposes and artificial cognition.

**Acknowledgements**

This work was funded by MINECO/FEDER DPI2015-65833-P (http://www.mineco.gob.es/) and ONRG grant N62909-14-1-N279 (PV) and by ONR MURI 14-13-1-0205 and MURI N00014-13-1-0678 (MIR)

**References**

1. Daselaar SM, Rice HJ, Greenberg DL, Cabeza R, LaBar KS, Rubin DC. The spatiotemporal dynamics of autobiographical memory: Neural correlates of recall, emotional intensity, and reliving. *Cereb. Cortex.* 2008; **18:**217–29.

2. Rabinovich MI, Afraimovich VS, Bick C, Varona P. Information flow dynamics in the brain. *Phys. Life Rev.* 2012; **9:**51–73.

3. Rabinovich MI, Tristan I, Varona P. Hierarchical nonlinear dynamics of human attention. *Neurosci. Biobehav. Rev.* 2015; **55:**18–35.

4. Rabinovich MI, Simmons AN, Varona P. Dynamical bridge between brain and main. *Trends Cogn. Sci.* 2015; **19:**453–461.

5. Varona P, Rabinovich MI. Hierarchical dynamics of informational patterns and decision making. Proc. R. Soc. B. 2016; **283:**20160475.

## P213 An integrated neuro-mechanical model of *C. elegans* locomotion

### Jack Denham, Thomas Ranner, Netta Cohen

#### School of Computing, University of Leeds, Leeds, LS2 9JT, UK

##### Correspondence: Jack Denham (scjde@leeds.ac.uk), Thomas Ranner (T. Ranner@leeds.ac.uk), Netta Cohen (N.Cohen@leeds.ac.uk)

*BMC Neuroscience* 2017, **18** (**Suppl 1**):P213

Across the animal kingdom, the generation and modulation of motor behaviour is attributed to Central Pattern Generators (CPGs) or neural circuits that endogenously produce oscillations. The ubiquity of CPGs prompts the use of coupled oscillator models to describe neural activity and the generation of behaviour. However, CPGs have not been identified in the forward locomotion system of the small roundworm *Caenorhabditis elegans*. In this case, a proprioceptive mechanism, in which motor-neurons respond to local body stretch, is thought to drive sustained body undulations. Since the wavelength and frequency of oscillations has been shown to depend on the visco-elasticity of the surrounding medium [1], it is important to include environmental effects in such locomotion models [1, 2]. This requires the integration of the nervous system and body mechanics in a continuous feedback loop which is able to adapt in response to environmental changes. Here, a biologically grounded model describing neural activity (adapted from [1]) is integrated into a novel continuum soft-body model [2]. We present a dynamical systems description of the local pattern generation mechanism with fictive proprioceptive feedback and compare this with the actual feedback in whole body simulations. The closed loop neuro-mechanical model is demonstrated to produce realistic travelling waves down the body in silico. The effect of the material properties of the body is investigated.

**References**

1. Boyle JH, Berri S, Cohen N: Gait modulation in c. elegans: an integrated neuro-mechanical model. *Frontiers in computational neuroscience 2012,* ***6***:10.

2. Cohen N, Ranner T: A new computational method for a model of C. elegans biomechanics: Insights into elasticity and locomotion performance, arXiv:1702.04988, 2017.

## P214 A computational approach to understanding functional synaptic diversity: the role of nanoscale topography of Ca^2+^ channels and synaptic vesicles

### Maria Reva^1^, Nelson Rebola^1^, Tekla Kirizs^2^, Zoltan Nusser^2^, David DiGregorio^1^

#### ^1^Laboratory of Dynamic Neuronal Imaging, Neuroscience Department, Institute Pasteur, Paris, France, 75015; ^2^Institute of Experimental Medicine, Hungarian Academy of Sciences, Budapest, Hungary, 1083

##### Correspondence: Maria Reva (maria.reva@pasteur.fr)

*BMC Neuroscience* 2017, **18** (**Suppl 1**):P214

Understanding the spatial relationship between the synaptic vesicles and the voltage-gated Ca^2+^ channels (VGCCs) is critical for deciphering the determinants of synaptic strength, time course, and plasticity. Furthermore, synaptic strength, within a homogeneous population of synapses, is highly heterogeneous, but the underlying mechanisms are poorly understood. We hypothesize that variations in the nanoscale organization of VGCCs and synaptic vesicles contribute to the diversity of synaptic function observed throughout the brain [1]. Because VGCCs and synaptic vesicles can be as close as 10-20 nm, direct experimental observation of the spatio-temporal dynamics driving synaptic vesicle fusion is still challenging. We have taken a computational approach to simulate the spatio-temporal dynamics of Ca^2+^ -triggered vesicle fusion to examine channel-vesicle topologies that is consistent with experimental findings.

To understand the influence of topography on synaptic diversity, we performed Monte Carlo (MC) simulations designed to predict the different functional behavior of inhibitory and excitatory terminals within the cerebellar cortex. Model parameters were constrained to experimental data (such as single channel open probability, Ca^2+^ buffers kinetics, etc.) leaving only topographical arrangements of VGCCs and location of the release sensor as variables. In addition, we have analyzed replicas in which the VGCC subunit Cav2.1 was labeled. Using Ripley’s analysis and mean nearest neighbor distances (NND) calculations we concluded that the distribution of the Cav2.1 subunit was significantly different from complete spatial randomness in both excitatory and inhibitory axon terminals. Then using cluster analysis, we determined that inhibitory terminals exhibited small clusters, while the labeling on excitatory boutons seemed more amorphous. We therefore considered an arrangement based on a few simple rules: VGCCs and vesicles were placed randomly within the AZ, but with a minimal separation, we called this the exclusion zone (EZ) model. The EZ model produced channel NND distributions that were consistent with the electron microscopy data. We then performed reaction diffusion MC simulations, considering perimeter coupled model for inhibitory terminals and the exclusion topology for excitatory terminals. Our simulations predicted well the experimental data of Ca^2+^ chelator inhibition of synaptic release (EGTA inhibition) and release probability.

Our results suggest that inhibitory terminals use small clusters of VGCC to drive the fusion of vesicles located in their periphery (perimeter release model) as described previously at the excitatory calyx of Held synapses [2]. In contrast, excitatory synapses made by cerebellar parallel fibers require a more random placement of up to 3 times more VGCCs within the AZ, as well as random placement of vesicles with an exclusion zone of >40 nm. We therefore suggest that nanoscale distribution of VGCCs and synaptic vesicles differs among synapses and is a key factor underlying functional synaptic diversity.

**References**

1. Chabrol FP, Arenz A, Wiechert MT, Margrie TW, DiGregorio DA: Synaptic diversity enables temporal coding of coincident multisensory inputs in single neurons. *Nat Neurosci* 2015, **18(5):** 718–727.

2. Nakamura Y, Harada H, Kamasawa N, Matsui K, Rothman JS, Shigemoto R, Silver RA, DiGregorio DA, Takahashi T: Nanoscale distribution of presynaptic Ca(2 +) channels and its impact on vesicular release during development. *Neuron* 2015, **85(1):** 145–158.

## P215 Is object saliency perceived different cross-culturally: a computational modelling study

### Eirini Mavritsaki^1,2^, Panos Rentzelas^1^

#### ^1^Department of Psychology, Birmingham City University, Birmingham, UK; ^2^School of Psychology, University of Birmingham, Birmingham, UK

##### Correspondence: Eirini Mavritsaki (eirini.mavritsaki@bcu.ac.uk)

*BMC Neuroscience* 2017, **18** (**Suppl 1**):P215

Research on cross-cultural differences of visual attention has identified that cultural membership influence performance in object perception [1, 2]. Participants with collectivist background focus more on the background (distractors) and omit the target relevant information while participants from the individualists’ background tend to attend the target and omit the background information. Previous modelling work from our lab [3] predicted that in Visual Search task cultural memberships influences the performance of the tasks. The results showed that simulated efficiency of participants from the individualist group is significantly higher than simulated efficiency from participants from the collectivists group when the task is to identify a target amongst distractors in a classical easy visual search. Work in our lab then confirmed these predictions. Preliminary behavioral data supports the idea that the effect remains even if the target is more salient than the distractors. This difference is simulated and explored further by investigating the changes in the effect for different levels of saliency using the binding Search over Time and Space (bsSoTS) computational model [4, 5] as predictor of behavior.

bsSoTS is based on integrate-and-fire neurons that are tighter connected when they encode a specific characteristic of an item presented in one position on the Visual Field and loosely connected when they present the same characteristics but items presented in different positions on the visual field. Moreover, the model incorporates a number of synaptic currents and processes that allowed us to successfully simulate the Visual Search experiment [4, 5]. In research, cultural membership is usually investigated between collectivists (Asian cultures) and individualists’ groups (Western Europeans cultures) [1, 2]. The experiments that bsSoTS simulated so far are based on individualists’ groups [4, 5]. To simulate therefore the difference in behavior between collectivists and individualists, we need to simulate the difference observed in collectivists cultures. To do that we tested the coupling between the neurons that encode a specific item presented in one position on the Visual Field as a saliency parameter. The same parameter was used in preliminary modelling work in our lab [3].

The results showed that the saliency parameter successfully simulates the behavioral results. Additionally, further behavioral work is proposed by investigating the relationship between the different saliency levels and the observed effect.

**References**

1. Nisbet RE, Masuda T: Culture and point of view. *Proceedings of the National Academy of Sciences of the United States of America 2003,* **100**: 11163–11170.

2. Nisbet RE, Peng K, Choi I, Norenzayan A: Culture and systems of thought: Holistic versus analytic cognition. *Psychological Review 2001,* **108**: 291–310.

3. Mavritsaki E, Rentzelas P: Cross-cultural differences in visual attention: A computational modelling study. *BMC Neuroscience,* **16**: 204.

4. Mavritsaki E, Humphreys GW: Temporal binding and segmentation in Visual Search: A computational neuroscience analysis. *Journal of Cognitive Neuroscience 2015,* **28:** 1553–1567

5. Mavritsaki E, Heinke D, Allen HA, Deco G, Humphreys GW: Bridging the gap between physiology and behavior: Evidence from the sSoTS model of human visual attention. *Psychological Review 2011,* **118**: 3–41.

## P216 NeuroNLP: a natural language portal for aggregated fruit fly brain data

### Nikul H. Ukani^1^, Adam Tomkins^2^, Chung-Heng Yeh^1^, Wesley Bruning^3^, Allison L. Fenichel^4^, Yiyin Zhou^1^, Yu-Chi Huang^5^, Dorian Florescu^2^, Carlos Luna Ortiz^2^, Paul Richmond^6^, Chung-Chuan Lo^5^, Daniel Coca^2^, Ann-Shyn Chiang^5^, Aurel A. Lazar^1^

#### ^1^Department of Electrical Engineering, Columbia University, New York, NY 10027, USA; ^2^Department of Automatic Control & Systems Engineering, The University of Sheffield, Sheffield, S1 3JD, UK; ^3^Department of Computer Science, Columbia University, New York, NY 10027, USA; ^4^Data Science Institute, Columbia University, New York, NY 10027, USA; ^5^Brain Research Center, National Tsing Hua University, Hsinchu 30013, Taiwan; ^6^Department of Computer Science, The University of Sheffield, Sheffield, S1 4DP, UK

##### Correspondence: Aurel A. Lazar (aurel@ee.columbia.edu)

*BMC Neuroscience* 2017, **18** (**Suppl 1**):P216

NeuroNLP, a key application on the Fruit Fly Brain Observatory [1] platform (FFBO, http://fruitflybrain.org), provides a modern web-based portal for navigating fruit fly brain circuit data. Increases in the availability and scale of fly connectome data demand new, scalable and accessible methods to facilitate investigation into the functions of the complex circuits being uncovered. Combining data from multiple sources into a single database, with a common data model, NeuroNLP facilitates access to data from various sources simultaneously. It is built on top of the NeuroArch database [2] which codifies fly connectome data from both the FlyCircuit database [3] and the Janelia Fly Medulla data [4]. The former hosts meso-scale connectome data on the whole-brain level and the latter contains detailed, micro-scale synaptic information about the Medulla neuropil. NeuroNLP allows users to probe biological circuits in the NeuroArch database with plain English queries, such as “show glutamatergic local neurons in the left antennal lobe” and “show neurons with dendrites in the left mushroom and axons in the fan-shaped body”, replacing the cumbersome menus prevalent in today’s neurobiological databases. This enables in-depth exploration and investigation of the structure of brain circuits, using intuitive natural language queries that are capable of revealing latent structure and information. Equipped with powerful 3D visualization, NeuroNLP standardizes tools and methods for graphical rendering, representation, and manipulation of brain circuits, while integrating with existing databases such as the FlyCircuit. It currently supports queries to show, add, filter and remove neurons based on 1) the parent neuropil, 2) neuron type (local or projection), 3) dendritic/axonal arborization, 4) neurotransmitter and 5) related postsynaptic or presynaptic neurons. The graphical user interface complements the natural language queries with additional controls for exploring neural circuits. Designed with an open-source, modular structure, it is highly scalable and extensible to additional databases and languages. Accessible through a laptop or smartphone (Figure 1) at https://neuronlp.fruitflybrain.org, NeuroNLP significantly increases the accessibility of fruit fly brain data, streamlining the way we explore and interrogate distal data sources to open new avenues of research, and enrich neuroscience education.
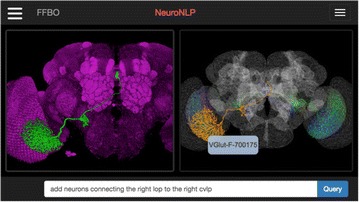


**Figure 1.** Smartphone screenshot of NeuroNLP showing 16 lobula plate tangential cells. Each neuron can be cross-linked to the FlyCircuit Database (left panel)

**References**

1. Ukani NH, Yeh C-H, Tomkins A, Zhou Y, Florescu D, Ortiz CL, Huang Y-C, Wang C-T, Richmond P, Lo C-C et al., The Fruit Fly Brain Observatory: from structure to function. *Neurokernel Request for Comments,* Neurokernel RFC #7, 2016. DOI: 10.1101/092288.

2. Givon LE, Ukani NH, Lazar AA, NeuroArch: A Graph dB for Querying and Executing Fruit Fly Brain Circuits, *Neurokernel Request for Comments*, Neurokernel RFC #4, 2015. DOI: 10.5281/zenodo.31947.

3. Chiang A-S, Lin C-Y, Chuang C-C, Chang H-M, Hsieh C-H, Yeh C-W, Shih C-T, Wu J-J, Wang G-T, Chen Y-C et al., Three-dimensional reconstruction of brain-wide wiring networks in Drosophila at single-cell resolution. *Cell* 2011, **21(1):**1–11.

4. Takemura S, Xu CS, Lu, Z, Rivlin PK, Parag T, Olbris DJ, Plaza S, Zhao T, Katz WT, Umayam L et al., Synaptic circuits and their variations within different columns in the visual system of Drosophila. *PNAS* 2015, **112(44):**13711–13716.

## P217 Towards prediction of plasticity response to paired cTBS from resting state network connectivity

### Bahar Moezzi^1^, Brenton Hordacre^1^, Mitchell R. Goldsworthy^1,2^, Michael C. Ridding^1^

#### ^1^Robinson Research Institute, School of Medicine, University of Adelaide, Adelaide, Australia; ^2^Discipline of Psychiatry, School of Medicine, University of Adelaide, Adelaide, Australia

##### Correspondence: Bahar Moezzi (bahar.moezzi@mymail.unisa.edu.au)

*BMC Neuroscience* 2017, **18** (**Suppl 1**):P217

Paired continuous theta burst stimulation (cTBS) is a non-invasive brain stimulation technique that can induce neuroplastic change in the primary motor cortex [1]. The response shows high intersubject variability and having a marker that might predict response would be useful in many situations. Our hypothesis is that a more strongly connected cortical network is associated with a greater plasticity response. To test this hypothesis, we quantify the correlation between graph theoretical measures of EEG connectivity data and the plasticity response to paired cTBS. We use state of the art methodologies in order to provide biological markers of response to paired cTBS to be used in their prediction.

We tested eighteen healthy adults (8 male, 1left handed) with a mean age of 24.2 (SD 6.0). Three minutes of continuous resting state EEG with open eyes was acquired. Baseline MEPs (n = ?) were recorded and then paired cTBS was applied to the left primary motor cortex, followed by three blocks of 20 TMS pulses. Surface EMG was used to record the motor evoked potential from the right first dorsal interosseous (FDI) muscle. We preprocessed EEG data and removed artefacts.

Graph theory provides a method to characterize the brain as a set of nodes interconnected by a set of edges [2]. It is suggested that an intracortical electrical source approach in graph theoretical analysis of EEG data is superior to the analysis at the surface level. Debiased weighted phase lag index is used as a measure of functional connectivity in the source space among the regions of interest. The connectivity matrix is thresholded and a graph is constructed. Several graph theoretical measures including degree, density, distance, clustering coefficient and characteristic path length are computed. Each participant’s plasticity response to paired cTBS is correlated with that participant’s graph theoretical measures (at each region of interest).

Preliminary analysis shows that the distance from the site of stimulation associates with the response to paired cTBS, while degree, density, clustering coefficient and characteristic path length do not. These findings suggest that graph theoretical measures of network connectivity may have some utility in predicting the neuroplasticity response to paired cTBS.

**References**

1. Goldsworthy MR, Pitcher JB, Ridding MC: Neuroplastic modulation of inhibitory motor cortical networks by spaced theta burst stimulation protocols. *Brain stimul* 2013, **6**:340–345.

2. Bullmore ET, Sporns O: Complex brain networks: graph theoretical analysis of structural and functional systems*. Nature Rev Neurosci* 2009, **10**:186–98.

## P218 Mathematical Analysis of Transient “domino effect” like Brain Dynamics

### Jennifer L. Creaser^1^, Congping Lin^1^, Peter Ashwin^1^, Jonathan T. Brown^2^, Thomas Ridler^2^

#### ^1^Department of Mathematics, University of Exeter, Exeter, EX4 4QD, UK; ^2^Institute of Biomedical and Clinical Sciences, University of Exeter Medical School, Exeter, EX4 4PS, UK

##### Correspondence: Jennifer L. Creaser (j.creaser@exeter.ac.uk)

*BMC Neuroscience* 2017, **18** (**Suppl 1**):P218

There has been much research into complex neurological diseases such as, for example, epilepsy and Alzheimer’s disease, however much remains unknown. It has become clear that such diseases are associated with abnormal brain network function including hyperexcitability. Brain network models used to study excitability, are often characterized by different dynamic regimes, such as alternating rest and excited states. The transient dynamics responsible for transitions between dynamic states are often discounted or overlooked in favour of the long term asymptotic behaviour. However, analysis of these transitions is instrumental in understanding, for example, the onset and evolution of epileptic seizures.

We consider a model of seizure initiation represented by a network of diffusively coupled bi-stable neurones driven by noise. Nodes in the network can switch between the quiescent attractor and active attractor due to noise fluctuations. We focus on the case of sequential escapes of nodes and the associated escape times. Understanding the factors controlling sequential transitions between stable/unstable attractors is important as they have been implicated in a diverse range of brain functions associated with neuronal timing, coding, integration as well as coordination and coherence [1, 2]. Network properties such as the coupling and excitability of nodes in such systems can promote (or suppress) escape of others on the network. We aim to quantify and characterise the escape times in terms of the coupling and excitability of nodes.

We apply our theoretical framework to investigate escape times to the propagation of epileptiform activity in parasagittal brain slices containing mouse medial entorhinal cortex (mEC). We observe sequential recruitment of electrodes to the ictal-like state and can determine the escape time, that is the equivalently average burst start time of each electrode. The sequential recruitment of electrodes to the ictal-like state could be seen as sequential escapes to an excited state in the underlying functional brain networks. We explore differences in intrinsic (node) excitability across the mEC by incorporating an excitability gradient into our prototypical bi-stable model. Figure 1 shows preliminary findings comparing the average burst start time observed in experiments (grey) and computed with the bi-stable model (black). In this presentation, I will address the question how a network’s structure and its properties influence sequential recruitment/escape of nodes in a network.
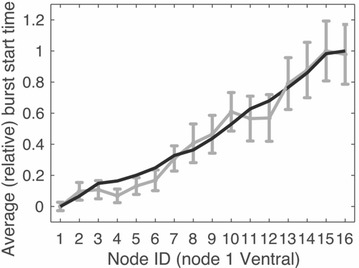


**Figure 1.** The average start time of ictal activity relative to ventral-most channel recorded from along the dorso-ventral axis of the mEC in vitro using a 16-shank silicon probe array (grey) with the average start time for each channel computed using 1000 simulations of a unidirectionally coupled 16 node bi-stable system with a linear excitability gradient (black)

**References**

1. Rabinovich, MI, Pablo V: Robust transient dynamics and brain functions. *Front Comput Neurosci* 2011, **5:** 24–33.

2. Rabinovich, MI, Ramon H, Gilles L: Transient dynamics for neural processing. *Science* 2008, **321(5885):** 48–50.

## P219 Synchronized neocortical dynamics during NREM sleep

### Daniel Levenstein^1,2^, Brendon O. Watson^2,3^, György Buzsáki^1,2^, John Rinzel^1,4^

#### ^1^Center for Neural Science, New York University, New York, NY, 10003, USA; ^2^NYU Neuroscience Institute, New York University, New York, NY, 10016, USA; ^3^Dept. of Psychiatry, Weill Cornell Medical Center, New York, NY, 10065, USA; ^4^Courant Institute for Mathematical Sciences, New York University, New York, NY, 10012, USA

##### Correspondence: Daniel Levenstein (dl2820@nyu.edu)

*BMC Neuroscience* 2017, **18** (**Suppl 1**):P219

During periods of behavioral quiescence such as NREM sleep, quiet wakefulness, and under anesthesia, neocortical populations can show ‘synchronized dynamics’ [1]: low-frequency alternations between low-rate spiking (UP states) and population-wide inactivity (DOWN states). Previous work has indicated that these dynamics are mediated by the interaction of recurrent excitation and neuronal adaptation [1–3]. Using a Wilson-Cowan model (Figure 1A), we show that synchronized regimes are seen during low levels of drive to a recurrent adapting neural population. Due to the possibility for both noise-induced and adaptation-induced transitions, this type of oscillation can show a range of spectral properties and UP/DOWN state dwell time statistics, which fit into 4 broad classes of synchronized regimes (Figure 1B). Using a nonparametric distribution-matching method, we find that this idealized model is able to reproduce the dwell time statistics of UP/DOWN states from multiple behavioral contexts in vivo.

During NREM sleep [4], DOWN states are coincident with large deflections in the LFP/EEG in a stereotyped pattern termed the ‘slow oscillation’. Unlike synchronized dynamics in other behavioral states (e.g. [5]), we find that the NREM slow oscillation is best represented by an ‘Excitable_UP_’ regime, in which noise or perturbation of a stable UP state can induce brief DOWN states (Figure 1C). Our model reveals a mechanistic basis for multiple features of NREM sleep that are thought to be related to mnemonic and homeostatic functions [6]: impulse-initiated slow waves and sequential activity at the DOWN->UP transition accompanied by gamma-band activity.
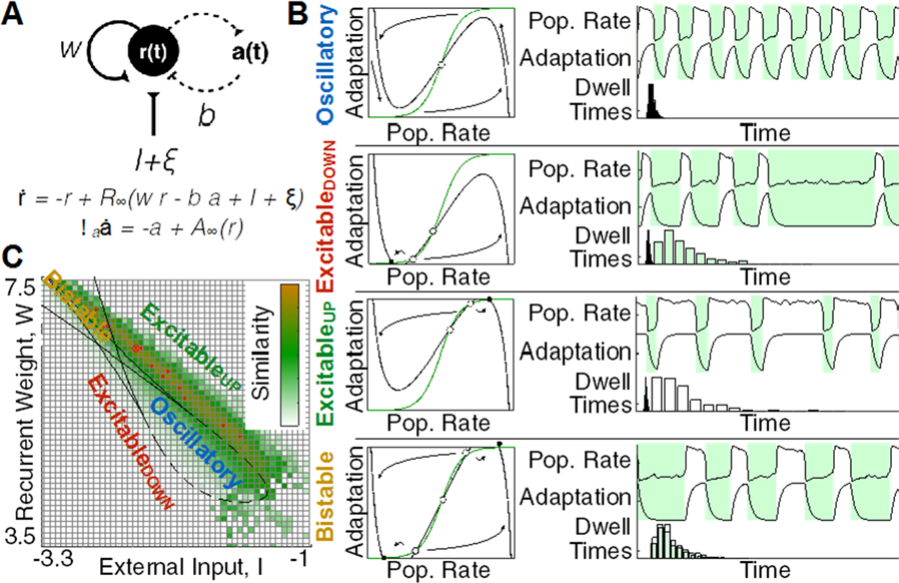


**Figure 1**. Synchronized dynamics in an adapting Wilson-Cowan model. **A**. Model schematic and equations. **B**. Synchronized regimes available to the model. (Left) Phase plane. (Right) Simulated time courses and dwell time distributions. **C.** State diagram in I-W reveals parameter domain for each synchronized regime. Color indicates similarity to NREM sleep. Solid/dashed line: saddle-node/Hopf bifurcations

**References**

1. Harris KD, Thiele A: Cortical state and attention. *Nature Reviews Neuroscience* 2011. **12(9):**509–523.

2. Parga N, Abbott LF: Network model of spontaneous activity exhibiting synchronous transitions between up and down States. *Frontiers in Neuroscience* 2007; **1(1):**57–66.

3. Compte A, Sanchez-Vives MV, McCormick DA, Wang XJ: Cellular and network mechanisms of slow oscillatory activity and wave propagations in a cortical network model. *J. Neurophys* 2003; **89(5):**2707–2725.

4. Watson BO, Levenstein D, Greene JP, Gelinas JN, Buzsáki G: Network Homeostasis and State Dynamics of Neocortical Sleep. *Neuron* 2016; **90(4):**839–852.

5. Mochol G, Hermoso-Mendizabal A, Sakata S, Harris KD, de la Rocha, J: Stochastic transitions into silence cause noise correlations in cortical circuits. *PNAS* 2015; **112(11):**3529–3534.

6. Levenstein D, Watson BO, Rinzel J, Buzsáki G. Sleep regulation of the distribution of cortical firing rates. *Current Opinion in Neurobiology* 2017. *In press.*

## P220 Accumulation process and multi-layer mechanisms of perceptual alternation in auditory streaming

### Rodica Curtu^1^, Anh Nguyen^1^, John Rinzel^2^

#### ^1^Department of Mathematics, The University of Iowa, Iowa City, IA 52242, USA; ^2^Courant Institute of Mathematical Sciences, New York University, New York, NY 10003, USA

##### Correspondence: Rodica Curtu (rodica-curtu@uiowa.edu)

*BMC Neuroscience* 2017, **18** (**Suppl 1**):P220

In daily life, the auditory system sorts the mixture of sounds from different sources into specific acoustic information by grouping acoustic events over time and forming internal representations of sound streams. A particular set of stimuli that have been used intensively to study that phenomenon consists of sequences of alternating high (A) and low (B) pure tones presented as repeated triplets, ABA_ABA_….Depending on the frequency separation (df) between the two tones, subjects report either of two percepts: “integration” (a single, coherent stream of high and low tones, like a galloping rhythm) or “segregation” (two parallel distinct streams). In our lab, the psychophysical experiment was conducted on 15 human subjects of normal hearing. They were prompted to listen to repeating sequences of ABA_ triplets at df = 3, 5, 7 semitones difference, with a total of 675 trials per df condition. Each sequence was comprised of sixty 500 ms-long triplets, resulting in a 30 s-long presentation. Subjects were asked to press and hold different buttons on a keypad when they perceived integration and segregation, respectively. Data analysis revealed time course and statistical distribution of perceptual switching. After the stimulus onset, it takes several seconds for the trial-averaged probability of stream segregation to build up, and the first percept is typically integration. Also, subjects report spontaneous alternations between the two percepts, and the percept durations are gamma-distributed. Furthermore, a previous study reveals that there are similarities between build-up functions of stream segregation from psychophysical experiments (*psychometric* functions) and those from multi-unit recordings from monkeys’ primary auditory cortex (area A1) (*neurometric* functions) [1]. In this presentation, we first demonstrate that a signal-detection model introduced in [1] to compute neurometric functions, is not sufficient to produce realistic percept durations as reported experimentally. In particular, mean spike counts extracted from cortical recordings [1] were used to generate neuronal responses, which were used as inputs to a signal-detection model. We showed that this model produces percept durations whose distribution is exponential (not gamma) and whose means are significantly smaller than those reported experimentally. We propose an extension to this model in the form of a multi-stage feedforward auditory network with components: i) area “A1” whose local outputs (mean spike counts) are subject to threshold-based binary classifiers (binary neurons); ii) An ensemble of binary neurons (BN) receiving local input from “A1”; and iii) Two competing units (“the accumulators”) whose activities depend on accumulated evidence from neuronal ensemble BN for each of the two percepts, integration and segregation. The suppressed neuronal unit accumulates evidence against the current percept while the dominant unit gradually reduces its activity. Both are drifting towards their given thresholds.

**Conclusion:** The proposed evidence accumulation model is able to reproduce qualitatively and quantitatively switching behavior between integration and segregation in auditory streaming. At each df the model produced percept durations whose distribution is gamma-like and whose means are comparable to those obtained in our psychophysical experiment.

**Acknowledgements**

This material is based upon work supported by the National Science Foundation under Grant Number CRCNS 1515678

**References**

1. C. Micheyl, B. Tian, R. Carlyon, R. Rauschecker: Perceptual organization of tone sequences in the auditory cortex of awake macaques. *Neuron* 2005, **48:**139–148.

2. D. Barniv, I. Nelken: Auditory streaming as an online classification process with evidence accumulation. *PLOS ONE 2015.*

3. R. Cao, A. Pastukhov, M. Mattia, J. Braun: Collective Activity of Many Bistable Assemblies Reproduces Characteristic Dynamics of Multistable Perception. *J Neurosci* 2016, **36(26):**6957–6972.

## P221 The Necessity of Sleep and Wake: Synaptic Homeostasis via System-Level Plasticity and the Ascending Arousal System

### Sahand Assadzadeh^1,2^, Peter A. Robinson^1,2^

#### ^1^School of Physics, The University of Sydney, NSW 2006, Sydney, Australia; ^2^Center for Integrative Brain Function, The University of Sydney, NSW 2006, Sydney, Australia

##### Correspondence: Sahand Assadzadeh (sahanda@physics.usyd.edu.au)

*BMC Neuroscience* 2017, **18** (**Suppl 1**):P221

One of the important functions of sleep is believed to be the regulation of synaptic weights in the brain. Mounting experimental evidence has found that on average, synapses that are upscaled during wakefulness are downscaled during sleep, providing a possible mechanism through which synaptic stability is maintained in the brain. This is often referred to as the synaptic homeostasis hypothesis (SHH) [1]. However, the questions of how and why sleep is necessary to fulfill this function remain unanswered. Neural field theory (NFT) has shown that synaptic plasticity dynamics depend strongly on network level effects, such as the overall system frequency response, with especially enhanced plasticity at resonances [2]. NFT is used to study the system-level effects of plasticity in the corticothalamic system, where arousal states are represented parametrically by the connection strengths of the system, among other physiologically based parameters (Fig. 1). Here it is found that the plasticity dynamics have no fixed points or closed cycles in the parameter space of the connection strengths; but parameter subregions exist where flows have opposite signs. Remarkably, these subregions coincide with previously identified regions corresponding to wake and slow-wave sleep, thus demonstrating the role of state-dependent activity on the sign of synaptic modification. We then show that a closed cycle in the parameter space is possible by coupling the plasticity dynamics to that of the ascending arousal system (AAS), which moves the brain back and forth between sleep and wake, and thus between the opposite-flow subregions to form a closed loop. In this picture, both wake and sleep are necessary to stabilize connection weights in the brain, because each modifies synaptic strengths in an opposite direction relative to the other.
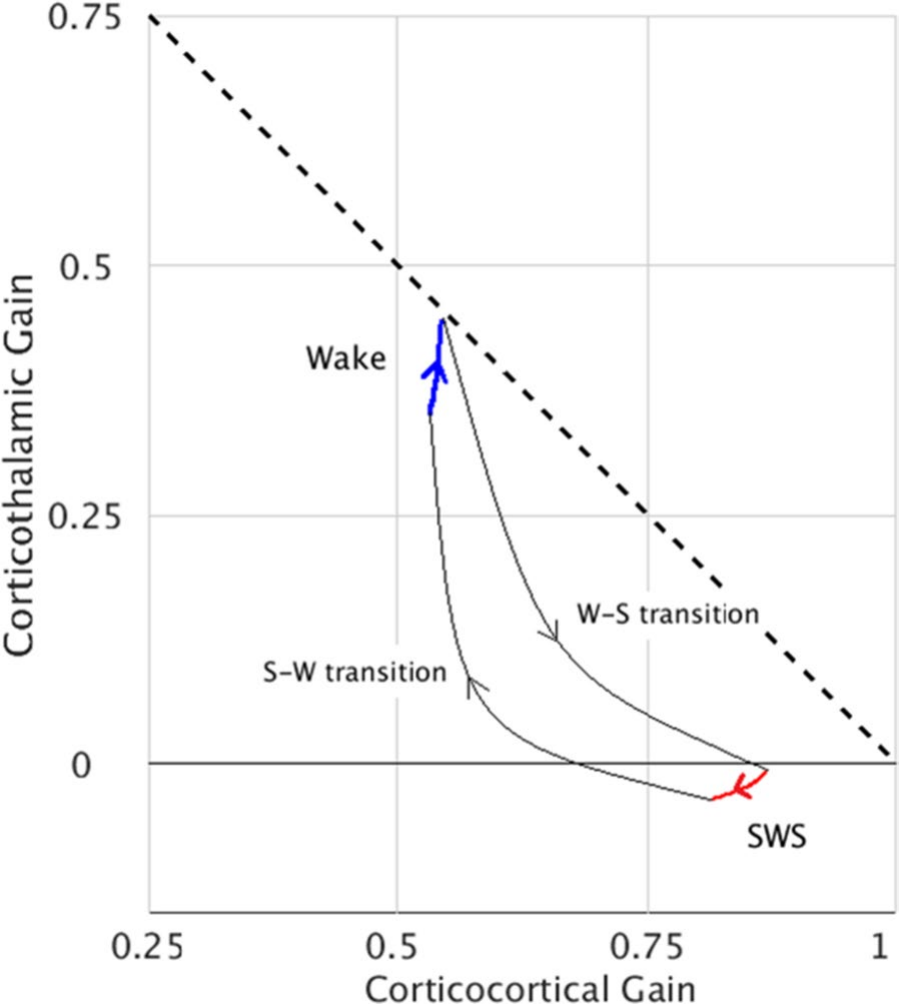


**Figure 1.** Evolution of connection strengths around a wake-sleep cycle forming a closed loop in arousal state space. The blue line represents plastic effects during wakefulness that result an increase of the corticothalamic and corticocortical loop gains in the corticothalamic system, with red lines corresponding to the opposite effect observed during slow-wave sleep. Thin lines indicate the action of the AAS in switching between wake and sleep states

**Acknowledgements**

This work was supported by the Australian Research Council under Center of Excellence for Integrative Brain Function Grant CE140100007 and Laureate Fellowship Grant FL140100025.

**References**

1. Tononi G, Cirelli C. Sleep and the Price of Plasticity: From Synaptic and Cellular Homeostasis to Memory Consolidation and Integration. Neuron. 2014; **81(1**): 12–34.

2. Robinson PA. Neural field theory of synaptic plasticity. J Theor Biol. 2011; **285(1):** 156–163.

## P222 Low- and high-mode waking states in the corticothalamic system

### Paula Sanz-Leon^1,2^, Peter A. Robinson^1,2^

#### ^1^School of Physics, University of Sydney, Sydney, New South Wales, Australia; ^2^Center for Integrative Brain Function, University of Sydney, Sydney, New South Wales, Australia

##### Correspondence: Paula Sanz-Leon (paula.sanz-leon@sydney.edu.au)

*BMC Neuroscience* 2017, **18** (**Suppl 1**):P222

A neural field model of the corticothalamic system has multistable regions of five steady-state solutions, up to three of which are linearly stable [1]; and, up to two of which lie within firing rate levels that are considered moderate, yet normal, in adult human physiology [2]. This confirms the existence of additional arousal states beyond the traditional steady states which have been identified with either normal or seizure-like activity [2]. The signature of these additional states, which we call H-mode states, is an overall increased level of activity up to 35 s^−1^ [blue dots in Figs 1(a) and 1(b)] with respect to the canonical waking states, or L-mode states (black dots). More specifically, compared to the L-states (illustrated as black dots), the H-states exhibit enhanced thalamic activity. In Fig. 1(c) mean firing rates are arranged in parallel coordinates where the coordinates correspond to cortical (ϕ_e_), reticular (ϕ_r_), and relay nuclei (ϕ_s_) firing rates. This type of plot allows for the identification of trends within a group, and for the comparison with another group. Here, we observe that the qualitative behavior of the H-states (blue lines) is similar to the one of the L-states (black lines): ϕ_e_ < ϕ_r_ and ϕ_s_ < ϕ_r_. However, in the H-states, despite the large dispersion of relay activity, cortical activity remains relatively constant. In Fig. 1(d), we show the power spectra for both L- and H-states (illustrated in black and blue lines, respectively). The H-states (i) have higher power density than the L-states over all the frequency range (0 < f < 45 Hz); and (ii) have a 5-order of magnitude increase in the power in the high-beta and gamma bands (20-35 Hz) with respect to the baseline spectra of waking states. This last result is consistent with focused and hyperarousal states found in the literature [3]. In hyperarousal increased thalamic activity is linked to high levels of attention and gamma enhancements expected due to increased activity in the relay nuclei of the thalamus.
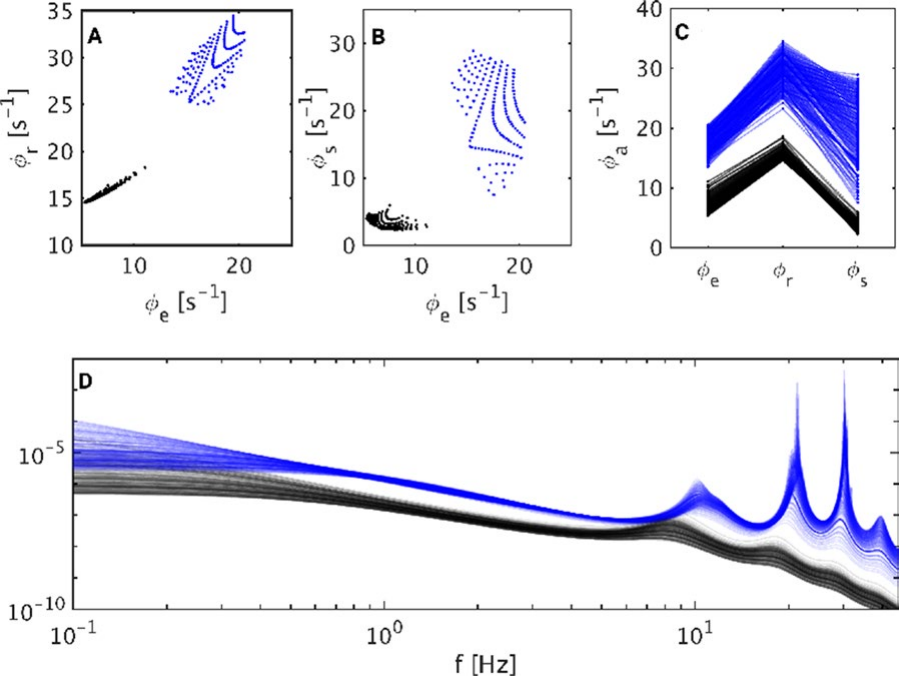


**Figure 1.** Comparison of L-mode states and H-mode states from multistable regions of the corticothalamic system. Black dots and lines correspond to properties of L-states (f_a_ < 20 s^−1^), while blue dots and lines are those of the H-states (f_a_ around 30 s^−1^). Panels **A** and **B** are the steady states in ϕ_e_-ϕ_r_ and ϕ_e_-ϕ_s_ space, respectively. Panel **C** shows a parallel coordinate plot of the corticothalamic firing rates. Panel **D** shows the spectral signature the L-states and H-states

**References**

1. Sanz-Leon P and Robinson PA: Multistability in the corticothalamic system. *J. Theor. Biol. 2017* (under review)

2. Robinson PA, Rennie CJ, Wright JJ, Bahramali H, Gordon E, Rowe DL: Prediction of electroencephalographic spectra from neurophysiology. *Phys. Rev. E 2001*; **63:**021903.

3. GrØnli J, Rempe MJ, Clegern WC, Schmidt M and Wisor JP. Beta EEG reflects sensory processing in active wakefulness and homeostatic sleep in quiet wakefulness. *J. Sleep Res. 2001;* **25:**257–268.

## P223 Closed-loop temporally structured light stimulation in weakly electric fish

### Caroline G. Forlim^1,2^, Lírio O. B. de Almeida ^3^, Ángel Lareo^4^, Reynaldo D. Pinto^3^, Pablo Varona^4^, Francisco B. Rodríguez^4^

#### ^1^Clinic and Policlinic for Psychiatry and Psychotherapy, University Medical Center Hamburg-Eppendorf, Hamburg, 20246, Germany; ^2^Departamento de Física Geral, Universidade de Sao Paulo, Sao Paulo, 05508-090, Brazil; ^3^Instituto de Física de Sao Carlos, Universidade de Sao Paulo, Sao Carlos, 13560-970, Brazil; ^4^Escuela Politécnica Superior, Universidad Autonoma de Madrid, Madrid, 28049, Spain

##### Correspondence: Caroline G. Forlim (c.garcia-forlim@uke.de), Francisco B. Rodríguez (f.rodriguez@uam.es)

*BMC Neuroscience* 2017, **18** (**Suppl 1**):P223

Closed-loop stimulation is a promising technique for neuroscience studies, especially in behavioral experiments [1, 2]. Weakly electric fish discharge short electric pulses or waves through an electric organ and detect small changes in the electric field using electroreceptors [1, 3]. These fish live in turbid waters and use electrical sensing as an additional sense to increase visual details. In addition, their electric pulses are also used to communicate by changing their inter pulse intervals depending on the behavioral context [3]. Recently, attention has been paid to the visual system [4]. However, most experiments assessing vision were conducted with periodic flashlights lasting just a few seconds and moreover, in restrained animals.

We developed the first closed-loop setup that uses temporally structured light as a stimulus for long periods in freely swimming fish. In these closed-loop protocols, the light pulses are triggered based on the real time monitored electrical activity, resulting in stimulus with similar complex temporal structure as the electrical signaling of the fish. The setup can be easily adapted to different stimulus modalities such as mechanical, acoustic and electrical stimulation allowing studies of multisensory integration.

Our validation protocol consisted of 15 min control session followed by 15 min light pulse stimulation in *Gnathonemus petersii*. The light stimuli were either triggered by the fish’s own electrical activity and therefore with complex temporal structure or periodic. It is important to emphasize that the main differences between these two stimuli is the temporal structure, the closed-loop share similar complex temporal structure as the electrical signaling and the periodic does not, no temporal structure is encoded in the light stimulus. We show that, for long light stimulation periods, fish decreased the discharge rate. The decrease in discharge was more accentuated when light stimuli were triggered by the fish’s electrical activity as opposed to periodic stimuli, meaning that probably the information encoded in the temporal structure was somehow meaningful for the fish and that the brain processed it distinctly from a simple periodic structure.

To the best of our knowledge, this is the first study on how light can influence the fish electrical system for long periods of time. The results give rise to important questions on the influence of light in electrocommunication and the processing of multisensory information, which can be addressed using the proposed methodology.

**Acknowledgements**

This work was funded by Spanish projects of Ministerio de Economía y Competitividad/FEDER TIN2014-54580-R, DPI2015-65833-P, ONRG grant N62909-14-1-N279, Spanish-Brazilian Cooperation PHB2007-0008 and 7ª Convocatoria De PROYECTOS de COOPERACION INTERUNIVERSITARIAUAM-SANTANDER con America Latina and Brazilian Agency of Conselho Nacional de Desenvolvimento Científico e Tecnológico and Fundação de Amparo à Pesquisa do Estado de São Paulo.

**References**

1. Forlim CG, Pinto RD, Varona P, Rodríguez FB. Delay-Dependent Response in Weakly Electric Fish under Closed-Loop Pulse Stimulation. *PLoS ONE* 2015;10:e0141007. doi:10.1371/journal.pone.0141007.

2. Lareo A, Forlim CG, Pinto RD, Varona P, Rodriguez F. de B. Temporal Code-Driven Stimulation: Definition and Application to Electric Fish Signaling. *Front Neuroinform* 2016;10:41. doi:10.3389/fninf.2016.00041.

3. Bullock TH, Hopkins CD, Popper AN, Fay RR, editors. Electroreception. vol. 21. Springer New York; 2005.

4. Pusch R, Kassing V, Riemer U, Wagner HJ, von der Emde G, Engelmann J. A grouped retina provides high temporal resolution in the weakly electric fish Gnathonemus petersii. *J Physiol Paris* 2013;107:84–94.

## P224 Information-theoretic analysis of temporal code-driven stimulation applied to electroreception

### Ángel Lareo^1^, Caroline Garcia Forlim^2^, Reynaldo D. Pinto^3^, Pablo Varona^1^, Francisco B. Rodríguez^1^

#### ^1^Grupo de Neurocomputación Biológica, Departamento de Ingeniería Informática, Escuela Politécnica Superior, Universidad Autónoma de Madrid, Madrid, Spain; ^2^Clinic and Policlinic for Psychiatry and Psychotherapy, University Medical Center, Hamburg-Eppendorf, Hamburg, Germany; ^3^Lab. Neurodynamics/Neurobiophysics - Dept. Physics and Interdisciplinary Sciences - Institute of Physics of São Carlos, Universidade de São Paulo, São Paulo, Brazil

##### Correspondence: Ángel Lareo (angel.lareo@uam.es), Francisco B. Rodríguez (f.rodriguez@uam.es)

*BMC Neuroscience* 2017, **18** (**Suppl 1**):P224

Biological systems can encode information in a sequential manner, and temporal encoding gives rise to complex temporal patterns of activity. Thus, information processing in those systems can be analyzed studying the temporal structure of event trains. This is the approach followed by a recently defined real time stimulation methodology, temporal code-driven stimulation (TCDS) [1]. TCDS is a closed-loop stimulation protocol that first digitizes and binarizes a biological signal and then delivers the stimulus when a predefined code is detected. This code represents the sequential activity in the signal whose meaning is the goal of the system study. The methodology can use the study of changes in the information processing of a given biological system among different sessions: code-driven stimulation sessions, control sessions without stimulation and open-loop stimulation sessions.

In order to test this methodology, an implementation of TCDS using hard real time has been applied to electroreception using the weakly electric fish Gnathonemus Petersii. The electromotor neurons of this animal generate electrical signal pulses which can be measured in a water tank using appropriate hardware [2, 3]. These signals follow a temporal coding scheme [4] where information is encoded in the inter-pulse interval (IPI) [5]. Thus, it constitutes a convenient animal model to test closed-loop stimulation methods in an alive and freely-behaving biological system. The TCDS protocol binary digitizes the signal of the fish detecting the presence or absence of a pulse event during the binarization period and uses this codification to stimulate after detecting a preselected code from the fish’ activity. Analysis of information processing in weakly electric fish is done in previous studies in terms of IPIs distribution [1].

We complement the analysis of the TCDS protocol with a measure based on information theory: Transitions between codes. As a proof of concept, we used 4-bit codes and selected as the trigger a code with mean probability of occurrence during control sessions. Codes were grouped by the number of pulses in them, defining three sets: *low*, *medium* and *high* number of pulses. Preliminary results applying TCDS to electroreception in weakly electric fish indicates that it distinctly conditions the response of the system when stimulating after a predetermined code. This conclusion is also drawn by analyzing the probability of transitions between codes, as an increase in *low*-*low* transition probability is detected when the system is stimulated with the code 0101.

**Acknowledgements**

We acknowledge support from MINECO/FEDER TIN2014-54580-R, DPI2015-65833-P (http://www.mineco.gob.es/) and ONRG grant N62909-14-1-N279.

**References**

1. Lareo A, Forlim CG, Pinto RD, Varona P, Rodriguez F: Temporal Code-Driven Stimulation: Definition and Application to Electric Fish Signaling. *Frontiers in Neuroinformatics* 2016, **10**:41.

2. Forlim CG, Pinto RD: Automatic realistic real time stimulation/recording in weakly electric fish: Long time behavior characterization in freely swimming fish and stimuli discrimination. *PLoS ONE* 2014, **9**:e84885 + .

3. Forlim CG, Pinto RD, Varona P, Rodriguez FB: Delay-dependent response in weakly electric fish under closed-loop pulse stimulation. 2015, **10**.

4. Baker CA, Kohashi T, Lyons-Warren AM, Ma X, Carlson BA: Multiplexed temporal coding of electric communication signals in mormyrid fishes. *The Journal of experimental biology* 2013, **216**:2365–2379.

5. Carlson BA: Electric signaling behavior and the mechanisms of electric organ discharge production in mormyrid fish. *Journal of Physiology*-*Paris* 2002, **96**:405–419.

## P225 Gain control mechanism based on lateral inhibition of antennal lobe improves pattern recognition performance under wide concentration variability

### Aaron Montero^1^, Thiago Mosqueiro^2^, Ramon Huerta^1,2^, Francisco B. Rodriguez^1^

#### ^1^Grupo de Neurocomputación Biológica, Dpto. de Ingeniería Informática, Escuela Politécnica Superior, Universidad Autónoma de Madrid, Madrid, 28049, Spain; ^2^BioCircuits Institute, University of California, San Diego, La Jolla, CA 92093-0402, USA

##### Correspondence: Aaron Montero (aaron.montero.m@gmail.com), Francisco B. Rodriguez (f.rodriguez@uam.es)

*BMC Neuroscience* 2017, **18** (**Suppl 1**):P225

Many animals depend on odor information for living. Although different levels of concentration produce variation in the activation patterns observed in olfactory receptor neurons, most animals can correctly recognize the identity of odors regardless of their concentration. It is not clear yet what mechanisms olfactory systems employ to recognize the same stimulus regardless of their concentrations. Experiments suggest that in insects this concentration invariance appears in the Antennal Lobe, where the activity of Projection Neurons remains nearly constant, even though the concentration changes [1]. One hypothesis is that the Local Neurons are responsible to down regulate the levels of activity (also known as gain control) by laterally inhibiting the Projection Neurons [2]. We examine the impact of this gain control mechanism on pattern recognition by designing a biologically plausible model based on the interactions between Local and Projection Neurons. For this purpose, we used a computational model that represents the olfactory system of insects by a single hidden layer network [3, 4, 5]. We consider three layers: Antennal Lobe, Kenyon cells and Mushroom Body Output Neurons. In order to simulate the activation patterns of Antennal Lobe for different concentration levels, we used Gaussian functions with a variable height and width, where their centers encode the identity of the odor. We used datasets of 3000 patterns divided into 10 pattern classes and 3 concentration levels. To model the intrinsic variations observed in real olfactory systems, we added a multiplicative white noise to these Gaussians with 3 different levels (small, medium, large). The performance of a network with this gain control mechanism presented significantly lower classification error rate than a network without gain control, with an improvement of ~45%. A network with this gain control achieved a classification error of ~0% for sets of patterns with small and medium noise and <5% for large noise. These results suggest that gain control mechanism does not only suppress outbursts of activity from input layers but also greatly improves learning in Mushroom Bodies. Finally, because this mechanism does not depend on any synaptic plasticity, in agreement with the biological literature, it can also be applied to chemical sensors in electronic devices for controlling changes in environmental conditions [6, 7].

**Acknowledgements**

This research was supported by TIN2014-54580-R, BES-2011-049274, NIH grant R01GM113967 and CNPq grant 234817/2014-3.

**References**

1. Stopfer M, Jayaraman V, and Laurent G: Intensity versus identity coding in an olfactory system. *Neuron* 2003, **39**:991–1004.

2. Olsen SR, Wilson RI: Lateral presynaptic inhibition mediates gain control in an olfactory circuit. *Nature* 2008 **452(7190)**:956–960.

3. Huerta R and Nowotny T: Fast and robust learning by reinforcement signals: Explorations in the insect brain. *Neural Comput.* 2009, **21**:2123–2151.

4. Montero A, Huerta R, and Rodriguez FB: Regulation of specialists and generalists by neural variability improves pattern recognition performance. *Neurocomputing*, 2015, **151**:69–77.

5. Montero A, Huerta R, Rodriguez FB: Specialist neurons in feature extraction are responsible for pattern recognition process in insect olfaction. *Artificial Computation in Biology and Medicine* - *International Work*-*Conference on the Interplay Between Natural and Artificial Computation* (IWINAC), Elche, Spain; 2015. part I p. 58–67.

6. Trincavelli M, Vergara A, Rulkov N, Murguia JS, Lilienthal A, Huerta R: Optimizing the operating temperature for an array of mox sensors on an open sampling system. *AIP Conference Proceedings*, 2011, **1362**:225.

7. Huerta R, Mosqueiro T, Fonollosa J, Rulkov NF, Rodriguez-Lujan I: Online decorrelation of humidity and temperature in chemical sensors for continuous monitoring. *Chemometr Intell Lab Syst*, 2016, **157**:169–176.

## P226 Maximum Relative Area as a Feature for Adaptability in ERP-based BCI Systems

### Vinicio Changoluisa^1,2^, Pablo Varona^1^, Francisco B. Rodriguez^1^

#### ^1^Grupo de Neurocomputación Biológica, Dpto. de Ingeniería Informática. Escuela Politécnica Superior, Universidad Autónoma de Madrid, Madrid, Spain; ^2^Universidad Politécnica Salesiana, Quito, Ecuador

##### Correspondence: Vinicio Changoluisa (fchangoluisa@ups.edu.ec), Francisco B. Rodriguez (f.rodriguez@uam.es)

*BMC Neuroscience* 2017, **18** (**Suppl 1**):P226

Adaptive Brain Computer Interfaces (BCI) are an important research topic in the last years. However, a critical and pending problem is their variable performance even within subjects. In event-related potentials (ERP)-based BCIs the variability of amplitude and latency impair the detection of the ERP components. In order to overcome those problems, target and non-target stimuli are repeated several times (trials). Repetitions can cause fatigue and a decrease in task performance. Therefore, achieving high accuracy with a few stimuli is a challenge. We propose a methodology that contributes to the management of variability in ERP-based BCIs through the characterization of the maximum relative voltage area (max^R^AUC) in the region of the EEG signal where a ERP component can be located. We call max^R^AUC relative since it is a maximum value within each trial, not the maximum value of all trials. This method calculates max^R^AUC incrementally in time for each stimulus. The one with the highest value is considered a target stimulus. In this way, the differences between a target and a non-target stimulus are maximized. Electrodes having the highest max^R^AUC in the ERP region of the signal are potentially likely to have better characteristics for detecting ERP effectively. Our method was tested with a linear classifier (LDA) based on the Krusienski method (KM) [1] and the dataset_IIb of the BCI competition (http://www.bbci.de/competition/ii/). This dataset contains the data of one user, divided into three sessions: two training sessions (called 10 and 11) and one session to test the classifier. Users were stimulated through P300 Speller Paradigm described in the competition. The electrodes with the largest max^R^AUC were found in the central and frontal lobes. We checked the influence of these electrodes on the system’s adaptability and evaluated the classifier with two configurations: the first, with 8 electrodes used in KM; and the second, by replacing Fz and Cz by the electrodes among those with the higher max^R^AUC of each session. With this electrode selection, the accuracy of the classifier improved and reached 100% success with a low number of trials, see Table 1. We also validated the robustness of our method by combining data from training sessions 10 and 11.

**Table:** Table 1. Trials needed to achieve 100% success in each session. Common Electrodes (CE): Pz, P3, P4, PO7, PO8, Oz. We emphasize the best results with italic font

	Session 10		Session 11		Session 10 + 11	
Electrode configuration	Elect.	Trials	Elect.	Trials	Elect.	Trials
KM electrodes	Cz + Fz + CE	4	Cz + Fz + CE	12	Cz + Fz + CE	9
CE + 2 max^R^AUC electrode	C1 + FPz + CE	3	*C1* *+* *FC1* *+* *CE*	*6*	*C3* *+* *F1* *+* *CE*	*4*
CE + 1 max^R^AUC electrode	*C3* *+* *CE*	*3*	F1 + CE	9	F1 + CE	5

In summary, here we propose a new methodology to extract additional information from EEG electrodes that contributes to manage the adaptability of ERP-based BCIs. This method adapts to the variability of each session and helps to decrease the number of electrodes and trials necessary to achieve a 100% success. The max^R^AUC contributes to early detection of ERP and further adaptation. This method can also be applied to other ERP components (N200, N100, etc.) which are considered for future work.

**Acknowledgements**

This work was funded by Spanish projects of Ministerio de Economía y Competitividad/FEDER TIN2014-54580-R, DPI2015-65833-P and Predoctoral Research Grants 2015-AR2Q9086 of the Government of Ecuador (SENESCYT).

**Reference**

1. Dean J Krusienski, Eric W Sellers, François Cabestaing, Sabri Bayoudh, Dennis J McFarland, Theresa M Vaughan, and Jonathan R Wolpaw: A comparison of classification techniques for the P300 Speller. *Journal of neural engineering* 2006, **3(4):**299–305.

## P227 Intrinsically stochastic neuron models for use in network simulations

### Vinícius L. Cordeiro, César C. Ceballos, Nilton L. Kamiji, Antonio C. Roque

#### Departamento de Física-FFCLRP, Universidade de São Paulo, Ribeirão Preto, SP 14040-901, São Paulo, Brazil

##### Correspondence: Vinícius L. Cordeiro (vinicius.lima.cordeiro@usp.br)

*BMC Neuroscience* 2017, **18** (**Suppl 1**):P227

Experimental evidence suggest that neurons are inherently stochastic systems displaying trial-to-trial response variability [1]. This stochasticity may have functional consequences on network behavior, so it is important to construct stochastic single-neuron models to be used in network simulations. There are basically two ways of constructing a stochastic neuron model [2, 3]. One is to consider a deterministic model, e.g. the leaky integrate-and-fire (LIF), Izhikevich or AdEx model [2], and add stochastic terms to the inputs received by the neuron. The other is to model a spike as an intrinsically stochastic event. The second way can be implemented in two different but equivalent manners: by a randomly varying spike threshold as in the escape noise model [4], or by a spike probability function Φ(*V*), which depends on the membrane potential *V* as in the simplified version of the Galves-Löcherbach (GL) discrete-time model [5] recently proposed by Brochini et al. [3].

Here we have considered the Brochini et al. [3] version of the GL model (from here onwards simply called GL model) and empirically determined the probability function Φ(*V*) so that the model can describe stochastic firing behaviors of the two most import cortical cell types, namely regular (RS) and fast (FS) spiking neurons [6]. To determine Φ(*V*) for these two cell types, biophysically detailed models of RS and FS neurons were chosen from the neuron database ModelDB (http://senselab.med.yale.edu/modeldb/) and submitted to realistic patterns of synaptic input. The detailed neuron model simulations were done in NEURON [7]. These simulations generated time series of membrane potential values *V*_t_ for the detailed RS and FS neuron models. From these time series, we determined action potential onset values *V*_th_ from the d*V*/d*t* versus *V* phase space using so-called Method II of [8]. For each action potential, the voltage values above threshold were discarded and with the remaining ones we constructed two distribution histograms, one for all voltage values (including *V*_th_) and the other for threshold values only. The histograms were superposed as in Figure 12 of [9] to allow an estimate of the probability of firing for each discretization bin.

The resulting probability functions display nonlinear exponential behavior. Based on them we constructed stochastic GL models for RS and FS neurons and submitted them to simulated input currents to obtain frequency-current (FI) curves. These stochastic neuron models can be used in large-scale simulations of cortical network models.

**Acknowledgements**

This work was produced as part of the activities of FAPESP Research, Disseminations and Innovation Center for Neuromathematics (grant 2013/07699-0, S. Paulo Research Foundation). NLK is supported by a FAPESP postdoctoral fellowship (grant 2016/03855-5). ACR is partially supported by a CNPq fellowship (grant 306251/2014-0).

**References**

1. Longtin A: Neuronal noise. *Scholarpedia* 2013, **8(9):**1618.

2. Gerstner W, Kistler WM, Naud R, Paninski L: Neural Dynamics: From Single Neurons to Networks and Models of Cognition. *Cambridge University Press* 2014.

3. Brochini L, Costa AA, Abadi M, Roque AC, Stolfi J, Kinouchi O: Phase transitions and self-organized criticality in networks of stochastic spiking neurons. *Sci Rep* 2016, **6:**35831.

4. Gerstner W, van Hemmen L: Associative memory in a network of ‘spiking’ neurons. *Network* 1992, **3:**139–164.

5. Galves A, Löcherbach E: Infinite systems of interacting chains with memory of variable length: a stochastic model for biological neural nets. *J Stat Phys* 2013, **151:**896–921.

6. McCormick DA, Connors BW, Lighthall JW, Prince DA: Comparative electrophysiology of pyramidal and sparsely spiny stellate neurons of the neocortex. *J Neurophysiol* 1985, **54:**782–806.

7. Carnevale NT, Hines ML: *The NEURON Book*. Cambridge University Press; 2006.

8. Sekerli M, Del Negro CA, Lee RH, Butera RJ: Estimating action potential thresholds from neuronal time-series: new metrics and evaluation of methodologies. *IEEE Trans Biomed Eng* 2004, **51:**1665–1672.

9. Azouz R, Gray CM: Cellular mechanisms contributing to response variability of cortical neurons in vivo. *J Neurosci* 1999, **19:**2209–2223.

## P228 Modeling action potential and network effects after site-directed RNA editing of sodium channels

### William W. Lytton^1,2^, Andrew Knox^3^, Joshua J. C. Rosenthal^4^

#### ^1^Depts. of Physiology & Pharmacology and Neurology, SUNY Downstate, Brooklyn, NY 11203 USA; ^2^Dept. of Neurology, Kings County Hospital, Brooklyn, NY 11203 USA; ^3^Dept. of Neurology, University of Wisconsin, Madison, WI 53705 USA; ^4^Dept. of Neurobiology, Marine Biological Laboratory, Woods Hole, MA 02543 USA

##### Correspondence: William W. Lytton (bill.lytton@downstate.edu)

*BMC Neuroscience* 2017, **18** (**Suppl 1**):P228

New techniques now make it possible to modify messenger RNA and thereby modify specific proteins in vivo. Experimentally, we have edited RNA using adenosine deamination to modify the mammalian fast sodium (Naf) channel (NaV1.4) by converting a key lysine residue to arginine in the selectivity region that is part of the aspartate-glutamate-lysine-alanine motif (DEKA to DERA). This change allows the channel to be permeable to both Na and K, effectively changing the reversal potential associated with this conductance to a value intermediate between the Nernst potentials of those two ions. The degree of alteration in the Naf channel can be manipulated, producing a mixed population of native and mutated channels. We modeled the effects of this manipulation on the classical Hodgkin-Huxley model of action potential propagation in the squid axon, as well as in other axonal models closer to mammalian morphology and temperature. As expected, action potential amplitude was reduced at higher percentages of the modified Naf channel, reaching a point where an action potential could no longer be maintained at the maximal conductance provided. Action potential conduction velocity was fast (approximately 10 mm/ms) when using a high-impedance axon termination, and showed little fall off with increased percent of modified channel. Conduction velocity was much slower (approximately 2 mm/ms) when using a low impedance termination, and showed a 20% falloff with increase in percent of the modified channel. These results were seen both at squid axon temperature and Ra (6.3^o^ C and 34.5 O-cm) and at mammalian values (37^o^ C and 250 O-cm). Action potentials were formed at lower sodium channel density and conducted at greater velocity at the low temperature, where the more prolonged activation due to the slower kinetics provided increased effect at neighboring locations.

RNA editing is being used experimentally to erase the mutations that introduce the premature termination codons that lead to cystic fibrosis. This manipulation has potential for clinical use in patients with this deadly genetic disease. Similarly, clinical manipulation of the RNA for the sodium channel has potential for use in intractable epilepsies such as Lennox-Gastaux syndrome, where neither surgical nor pharmacological intervention is generally effective.

**Acknowledgements**

The authors would like to acknowledge NIH support from EB02290301 (WL), EB017695 (WL), MH086638 (WL), NS087726 (JR).

## P229 Movement-related delta-theta synchronization in young and elderly healthy subjects

### Silvia Daun^1,2^, Svitlana Popovych^1,2^, Liqing Liu^1,2^, Bin A. Wang^1^, Tibor I. Tóth^2^, Christian Grefkes^1,3^, Gereon R. Fink^1,3^, Nils Rosjat^1,2^

#### ^1^Cognitive Neuroscience, Institute of Neuroscience and Medicine (INM-3), Research Center Juelich, Juelich, 52428 Germany; ^2^Heisenberg Research Group of Computational Neuroscience - Modeling Neural Network Function, Department of Animal Physiology, Institute of Zoology, University of Cologne, Cologne, 50674, Germany; ^3^Department of Neurology, University Hospital Cologne, Cologne, 50937, Germany

##### Correspondence: Silvia Daun (silvia.daun@uni-koeln.de)

*BMC Neuroscience* 2017, **18** (**Suppl 1**):P229

The wealth of data showing that human motor performance is affected by normal ageing is contrasted by the dearth of data on ageing effects on the neural processes underlying action. For example, it remains to be elucidated how the different phases of an action (i.e., preparation, initiation and execution) are expressed in neural oscillations and how these are affected by normal ageing. The interest in ageing-related changes of motor performance and the neural basis thereof are governed by the quest for more detailed insights into the possible reorganization of the key phases of an action. For this reason, it is apt and timely to study ageing-dependent effects on the neural organization of motor performance in more detail. The crucial point of such investigations is the study of synchronization, a key mechanism underlying the coordination of distinct neural populations in shaping complex motor tasks.

In an earlier EEG-study [1] on young adults, we found that when generating unilateral index-finger movements, *local* oscillations in the δ-θ frequency band over the centroparietal, central and frontocentral regions (corresponding to the primary motor area (M1), the supplementary motor area (SMA) and the pre-motor area (PM), respectively) exhibited robust phase locking both prior to and during the movement. The local oscillations were most pronounced in the hemisphere contralateral to the moving hand in both externally and internally triggered actions. A subsequent study [2] using an identical experimental paradigm with a population of older adults found that the *local* phase locking in the δ-θ frequency band was also present during the motor acts of the older participants.

To investigate the neural processes underlying ageing-related dependence of the motor performance in more detail, we employed *inter*-*regional* phase-locking analysis by calculating the phase-locking values (PLVs) from the EEG records of the two data sets mentioned above. PLV measures the extent of instantaneous synchronization between two distinct brain regions.

Our analysis revealed significant PLV in both age groups in the δ-θ frequencies around movement onset. Invariant sub-networks were established by strong PLV between brain areas involved in the motor act, which were different in older and younger subjects. More intra- and inter-hemispheric PLVs occurred in older than in younger subjects. Furthermore, data suggest that older subjects compensate for the diminished connectivity observed between contralateral M1 and SMA, and ipsilateral PM and SMA during movement preparation and execution by establishing additional intra- and inter- hemispheric connections.

Based on the above findings on local and inter-regional phase locking, we built a mathematical model consisting of phase oscillators representing two main regions of the motor network, i.e. SMA and M1. This simple model is capable of reproducing the effects of increased PLI and, independently of this, the effect of increased PLV between both regions. After extending the network model to all core motor regions and fitting the model parameters to the experimental data it will serve as a tool to make predictions on disturbed networks dynamics, e.g. decoupling of nodes.

**References**

1. Popovych S, Rosjat N, Tóth TI, Wang BA, Liu L, Abdollahi RO, Viswanathan S, Grefkes C, Fink GR, Daun S: Movement-related phase locking in the delta-theta frequency band. *NeuroImage* 2016, **139:** 439–449.

2. Liu L, Rosjat N, Popovych S, Yeldesbay A, Wang BA, Tóth TI, Grefkes C, Fink GR, Daun S: Movement related intra-regional phase locking in the delta-theta frequency band in young and elderly subjects. Program No. 624.08. 2016. Neuroscience Meeting Planner. San Diego, CA: Society for Neuroscience, 2016. Online.

## P230 ePyNN: a low cost embedded system for simulating Spiking Neural Networks

### Abraham Perez-Trujillo^1^, Andres Espinal^2^, Marco A. Sotelo-Figueroa^2^, Ivan Cruz-Aceves^3^, Horacio Rostro-Gonzalez^1^

#### ^1^Department of Electronics, University of Guanajuato, 36885 Salamanca, Guanajuato, Mexico; ^2^Department of Organizational Studies, University of Guanajuato, 3625 Guanajuato, Mexico; ^3^CONACYT, Mathematics Research Center (CIMAT), 36000 Guanajuato, Mexico

##### E-mail: Horacio Rostro-Gonzalez (hrostrog@ugto.mx)

*BMC Neuroscience* 2017, **18** (**Suppl 1**):P230

In this work, we present a low cost embedded system to simulate Spiking Neural Networks through PyNN [1]. PyNN is a Python library widely used in the neuroscience community to simulate at software and hardware level several existent simulators (NEURON, NEST, PCSIM and BRIAN) by acting as an interface to unify the different instructions and neuron model definitions. At hardware level, serves as a high-level interface to directly map spiking neuron models on the SpiNNaker neuromorphic system [2]. Albeit, SpiNNaker and other systems such as TrueNorth have demonstrated tremendous capabilities to process information such as the brain does, these systems are still unreachable for the large community who wants to implement or validate simplest models on a hardware platform. In this regard, we developed ePyNN, which is the PyNN simulator embedded on a Raspberry Pi 3 board, which has a 1.2 GHz 64-bit quad-core ARMv8 CPU. Here, we have been able to implement a neural network with the ≪ if_curr_exp ≫ model, which is a leaky integrate-and-fire model with fixed threshold and exponentially-decaying post-synaptic conductance to generate real time locomotion patterns expressed as spike trains for a hexapod robot [3, 4]. Specifically, we designed a network of 12 neurons, where each of them controls one of the degrees of freedom (servomotors) of the robot with a specific topology, which was offline performed by an evolutionary approach. Finally, the ePyNN has been successfully validated on a real hexapod robot (Figure 1C) for three different locomotion gaits (walk, jog and run) running in real time (Figure 1 A, B).
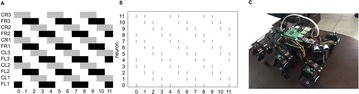


**Figure 1 A**. Biological Patterns **B**. Generated patterns **C**. Robot + ePyNN platform

**Acknowledgements**

This research has been supported by the CONACYT project “Aplicación de la Neurociencia Computacional en el Desarrollo de Sistemas Robóticos Biológicamente Inspirados” (No 269798).

**References**

1. Davison AP, Bruderle D, Eppler J, Kremkow J, Muller E, Pecevski D, Perrinet L, Yger P: PyNN: A Common Interface for Neuronal Network Simulators. *Front Neuroinform* 2008, **2**:11.

2. Furber SB, Galluppi F, Temple S, Plana LA: The SpiNNaker Project. *Proceedings of the IEEE* 2014, **102**(5):652–665.

3. Rostro-Gonzalez H, Cerna-Garcia PA, Trejo-Caballero G, Garcia-Capulin CH, Ibarra-Manzano MA, Avina-Cervantes JG, Torres-Huitzil C: A CPG system based on spiking neurons for hexapod robot locomotion. *Neurocomputing* 2015, **170**:47–54.

4. Espinal A, Rostro-Gonzalez H, Carpio M, Guerra-Hernandez EI, Ornelas-Rodriguez M, Sotelo-Figueroa M: Design of Spiking Central Pattern Generators for Multiple Locomotion Gaits in Hexapod Robots by Christiansen Grammar Evolution. *Frontiers in Neurorobotics* 2016, **10**:6.

## P231 Temporal structure of bilateral coherence in essential and physiological hand tremor

### Martin Zapotocky^1,2^, Soma Chakraborty^1,2^, Martina Hoskovcová^2^, Jana Kopecká^2^, Olga Ulmanová^2^, Evžen Růžička^2^

#### ^1^Institute of Physiology, Czech Academy of Sciences, Prague, 14220, Czech Republic; ^2^Department of Neurology, First Faculty of Medicine, Charles University in Prague, 120 00, Czech Republic

##### Correspondence: Martin Zapotocky (zapotocky@biomed.cas.cz)

*BMC Neuroscience* 2017, **18** (**Suppl 1**):P231

Pathological hand tremor is associated with a number of neurological diseases and may significantly impede motor functions in the patient. The most common pathological type is essential tremor (ET), found in 4.6% of the population aged over 65 years [1]. The neurophysiological basis of ET is still under debate, and recent literature suggests that patients with the ET diagnosis may in fact fall into several categories with distinct disease origins [2]. Detailed quantitative analysis of the features of the tremor may help in further classification and in clarifying the underlying neurophysiological mechanisms.

Depending on the underlying mechanism, the tremors in the left hand and right hand may be coupled or independent. In the previous literature on tremors, this bilateral coupling was assessed using stationary spectral coherence analysis, both on the level of hand kinematics and of muscle activity. Highly prevalent bilateral coherence was found for orthostatic [3] and psychogenic [4] tremors, while for other tremor types including ET, such coupling was only rarely reported. In our recent study [5], we used nonstationary, wavelet-based coherence analysis of kinematic recordings to show that the oscillations of the two hands are intermittently coupled in ET. We found that intervals of strong bilateral coherence, lasting for up to a dozen seconds, alternate with time intervals of insignificant coherence. We also observed intermittent bilateral coherence for physiological tremor (a normal hand oscillation of low amplitude) recorded in healthy subjects.

Here we further extend the analysis of Ref. [5], based on the same dataset of accelerometric recordings obtained from 34 ET patients and 42 healthy subjects. We analyze the distribution of durations of the bilaterally coherent time intervals extracted from wavelet analysis, and examine its dependence on the tremor type (physiological vs. essential) and on the hand position. The statistical significance of the coherence intervals is evaluated with surrogate analysis, using “natural” surrogates (the hand acceleration recorded from other subjects), as well as artificially constructed surrogates that have randomized Fourier phases but match the power spectrum and value distribution of the recorded time series [6]. We analyze separately the bilateral coupling of tremor amplitude, and evaluate its contribution to the bilateral coherence of tremor as assessed by spectral/wavelet coherence.

**Acknowledgements**

Supported by Czech Science Foundation (P304/12/G069), Charles University in Prague (Progres Q27, SVV NeST III), and Czech Health Research Council (AZV 16-28119A).

**References**

1. Louis ED, Ferreira JJ: How common is the most common adult movement disorder? Update on the worldwide prevalence of essential tremor. *Mov Disord* 2010, **25(5):**534–41.

2. Louis ED: Essential tremors: a family of neurodegenerative disorders? *Arch Neurol* 2009, **66(10):**1202–1208.

3. Lauk M, Köster B, Timmer J, Guschlbauer B, Deuschl G, Lücking CH. Side-to-side correlation of muscle activity in physiological and pathological human tremors. *Clin Neurophysiol* 1999, **110:**1774–1783.

4. Raethjen J, Kopper F, Govindan RB, Volkmann J, Deuschl G: Two different pathogenetic mechanisms in psychogenic tremor. *Neurology* 2004, **63:**812–815.

5. Chakraborty S, Kopecká J, Šprdlík O, Hoskovcová M, Ulmanová O, Růžička E, Zapotocky M: Intermittent bilateral coherence in physiological and essential hand tremor. *Clin Neurophysiol* 2017, **128(4):**622–634.

6. Schreiber T, Schmitz A: Improved surrogate data for nonlinearity tests. *Phys Rev Lett* 1996 **77(4):**635–638.

## P232 Detecting joint pausiness in parallel spike trains

### Matthias Gärtner^1^, Sevil Duvarci^2^, Jochen Roeper^2^, Gaby Schneider^1^

#### ^1^Institute of Mathematics, Goethe-University, Frankfurt, Germany; ^2^Neuroscience Center, Institute of Neurophysiology, Goethe-University, Frankfurt, Germany

##### Correspondence: Matthias Gärtner (gaertner@math.uni-frankfurt.de)

*BMC Neuroscience* 2017, **18** (**Suppl 1**):P232

Transient periods with reduced neuronal discharge - called ‘pauses’ - have recently gained increasing attention. In dopamine neurons, pauses are considered important teaching signals, encoding negative reward prediction errors. Particularly simultaneous pauses are likely to have increased impact on information processing. Available methods for detecting joint pausing analyze temporal overlap of pauses across spike trains. Such techniques are threshold dependent and can fail to identify joint pauses that are easily detectable by eye, particularly in spike trains with different firing rates.

We introduce a new statistic called ‘pausiness’ that measures the degree of synchronous pausing in spike train pairs and avoids threshold-dependent identification of specific pauses. A new graphic termed the ‘cross-pauseogram’ compares the joint pausiness of two spike trains with its time shifted analogue, such that a (pausiness) peak indicates joint pausing. When assessing significance of pausiness peaks, we use a stochastic model with synchronous spikes to disentangle joint pausiness arising from synchronous spikes from additional ‘Joint Excess Pausiness’ (JEP). Parameter estimates are obtained from auto- and cross-correlograms, and statistical significance is assessed by comparison to simulated cross-pauseograms.

Our new method was applied to dopamine neuron pairs recorded in the ventral tegmental area of awake behaving mice. Significant JEP was detected in about 20% of the pairs. Given the neurophysiological importance of pauses and the fact that neurons integrate multiple inputs, our findings suggest that the analysis of JEP can reveal interesting aspects in the activity of simultaneously recorded neurons.

**Acknowledgements**

This work was supported by the Priority Program 1665 of the DFG (DU 1433/1-1 to SD and JR, and SCHN 1370/2-1 to MG and GS), by an EMBO long-term fellowship (ALTF_210-2012 to SD), and by the German Federal

Ministry of Education and Research (BMBF, 01ZX1404B to GS).

## P233 A stochastic model relates responses to bistable stimuli to underlying neuronal processes

### Stefan Albert^1^, Katharina Schmack^2^, Gaby Schneider^1^

#### ^1^Institute of Mathematics, Goethe-University, Frankfurt a.M., Germany; ^2^Department of Psychiatry and Psychotherapy, Charité Universitätsmedizin, Berlin, Germany

##### Correspondence: Stefan Albert (albert@math.uni-frankfurt.de)

*BMC Neuroscience* 2017, **18** (**Suppl 1**):P233

Viewing of ambiguous stimuli can lead to bistable perception alternating between the possible percepts. The respective response patterns show differences between schizophrenic patients and healthy controls [1, 2]. At the same time, these patterns show similarities with spiking patterns of dopaminergic cells [3] that may be related to schizophrenia spectrum disorders. Specifically, oscillatory behavior [4] with single percept changes occurs during continuous viewing of ambiguous stimuli, and stable more or less regular periods followed by bursts of percept changes are observed during intermittent viewing of ambiguous stimuli.

Therefore, we propose a stochastic model that provides a link between the observed response patterns and potential underlying neuronal processes. To that end, we first develop a Hidden Markov Model that captures the observed group differences by describing switches between stable and unstable states in the intermittent presentation and using only one state in continuous presentation. Second, the model is embedded into a hierarchical model that describes potential underlying neuronal activity as difference between two competing neuronal populations similar to [5]. This differential activity is assumed here to generate switching between (i) the two conflicting percepts and between (ii) stable and unstable states with comparable mechanisms on different neuronal levels. Using only a small number of parameters, the model can be fitted to a large data set of perceptual responses of schizophrenic patients and healthy controls under continuous and intermittent stimulation. The model can closely reproduce a wide variety of response patterns and is able to capture and to provide potential neuronal mechanisms for group differences between healthy controls and schizophrenic patients such as the weaker tendency to stabilized perception in the patient group under intermittent stimulation [2].

**Acknowledgements**

This work was supported by the German Federal Ministry of Education and Research (BMBF, Funding number: 01ZX1404B; SA, KS, GS).

**References**

1. Schmack K, Gòmez-Carrillo de Castro A, Rothkirch M, Sekutowicz M, Rössler H, Haynes J, Heinz A, Petrovic P, Sterzer S: Delusions and the Role of Beliefs in Perceptual Inferences. *J Neurosci E* 2013, **33(34):**13701–13712.

2. Schmack K, Schnack A, Priller J, Sterzer P: Perceptual instability in schizophrenia: Probing predicitive coding accounts of delusions with ambiguous stimuli. *Schizophr Res Cog* 2015, **2(2):**72–77.

3. Bingmer M, Schiemann J, Roeper J, Schneider G: Measuring burstiness and regularity in oscillatory spike trains. *J Neurosci Methods* 2011, **201:** 426–437.

4. Brascamp JW, Pearson J, Blake R, van den Berg AV: Intermittent ambiguous stimuli: Implicit memory causes periodic perceptual alternations. *J Vis* 2009, **9(3):** 1-23.

5. Gigante G, Mattia M, Braun J, Del Guidice P: Bistable perception Modeled as Competing Stochastic Integration at Two Levels. *PLoS Comput Bio* 2009, **5(7):** e1000430.

## P234 Function and energy consumption constrain biophysical properties of neurons - an example from the auditory brainstem

### Michiel Remme^1,2^, John Rinzel^3,4^, Susanne Schreiber^1,2^

#### ^1^Institute for Theoretical Biology, Humboldt University, 10115 Berlin, Germany; ^2^Bernstein Center for Computational Neuroscience Berlin, Germany; ^3^Center for Neural Science, New York University, New York, NY 10003, United States; ^4^Courant Institute of Mathematical Sciences, New York University, New York, NY 10012, United States

##### Correspondence: Michiel Remme (michiel.remme@hu-berlin.de)

*BMC Neuroscience* 2017, **18** (**Suppl 1**):P234

Neural morphology and membrane properties vary greatly between cell types in the nervous system. While the function of neurons is thought to be the key constraint for their biophysical properties, additional constraints may further shape neuronal design and explain observed properties. Here, we focus on principal neurons in the MSO nucleus of the auditory brainstem and show that a tradeoff between a functionally relevant computation and energy consumption predicts optimal ranges of biophysical parameters.

Biophysical properties of MSO cells as well as their function are well characterized: MSO cells encode the direction of sound in the horizontal plane. Inputs to MSO cells are phase-locked to sound wave stimuli to each ear and the interaural time difference (ITD) of sound waves is used to compute source location. To achieve sensitivity to ITDs in the range of tens of μs, MSO cells have specialized membrane properties, including a very fast membrane time constant (~1 ms) and a low-threshold potassium current (I_KLT_), both contributing to a very short input integration window [1]. Furthermore, MSO cell function is supported by their bipolar morphology, with inputs from the two ears segregated to the two main dendrites [2].

Next to function, energy use can be assumed to significantly constrain MSO cell properties. Overall, the brain accounts for a disproportionately large part (~20%) of the energy budget, with metabolic energy being mostly spent on synaptic input, action potentials, and resting potentials [3]. MSO cells, in particular, receive inputs at very high rates (hundreds of Hz), generate action potentials at similarly high rates, and display a very leaky membrane.

Here, we quantify and contrast sensitivity of MSO cells to ITDs as well as the associated metabolic cost. We developed a simplified dendritic model of an MSO cell that includes the KLT-current. We first fit the model to experimental data from [1] and then explored how varying the morphological and membrane parameters affects performance and energy consumption. We found that most experimentally constrained parameters were close to a functional optimum; if a wider range of functionally good values was available, the fitted parameters tended towards lower energy usage. Interestingly, we found that the KLT-current increases energy costs, but strongly improves coincidence detection, beyond passive capabilities. We next explored the full parameter space by considering 100,000 models with random combinations of parameters. The experimentally constrained model was among the top 13% regarding performance and top 12% regarding energy efficiency (i.e., sensitivity per energy). Exploration of the full parameter space highlighted that two model features explain most of their performance and energy consumption: 1) the level of saturation of the driving force of the synaptic conductance inputs and 2) the width of the somatic compound EPSPs. We conclude that the neural design of MSO cells is indeed compatible with both functional and energetic constraints, with a preference of function over cost.

**Acknowledgements**

This work was supported by the Einstein Foundation Berlin and the German Federal Ministry of Education and Research (01GQ0901, 01GQ1403).

**References**

1. Mathews PJ, Jercog PE, Rinzel J, Scott LL, Golding NL. Control of submillisecond synaptic timing in binaural coincidence detectors by Kv1 channels. *Nat Neurosci* 2010. **13**:601–609.

2. Agmon-Snir H, Carr CE, Rinzel J: The role of dendrites in auditory coincidence detection. *Nature* 1998, **393:**268–272.

3. Attwell D, Laughlin SB: An energy budget for signaling in the grey matter of the brain. *J Cereb Blood* *Flow Metab* 2001, **21:**1133–1145.

## P235 The Brain Simulation Platform of the Human Brain Project: collaborative web applications and tools for data-driven brain models

### Michele Migliore^1^, Carmen A. Lupascu^1^, Luca L. Bologna^1^, Rosanna Migliore^1^, Stefano M. Antonel^2^, Jean-Denis Courcol^2^, Felix Schürmann^2^

#### ^1^Institute of Biophysics, National Research Council (CNR), Palermo, Italy; ^2^Blue Brain Project, École Polytechnique Fédérale de Lausanne (EPFL), Geneva, Switzerland

##### Correspondence: Michele Migliore (michele.migliore@cnr.it)

*BMC Neuroscience* 2017, **18** (**Suppl 1**):P235

The Brain Simulation Platform (BSP) of the Human Brain Project (HBP) provides a large set of tools to build, reconstruct, simulate and analyze data-driven brain models in a collaborative manner (Figure 1). The available tools are organized by use cases, consisting of selected procedures illustrating specific practical examples on how to exploit the Platform capabilities to pursue scientific goals.

The platform is designed to target users with different background and expertize such as: *a)* “end-users”, interested in using the platform in a user-friendly manner, *b)* “power-users”, able to take advantage of the platform services while integrating their own High Performance Computing resources, *c)* “expert-users”, who can contribute to the development of the tools, and *d)* “co-design developers” who are early adopters of initial versions of the platform facilities.

In this poster, we will give an overview of the current BSP release, the services it provides and the collaborative approach underlying its design. To illustrate the potential of the platform, and how users with different background can take full advantage of its tools, we will demo a few use cases in which “end-users” and-or “expert-users” are guided through step-by-step python-based jupyter notebook and web applications graphical interfaces (Figure 1).
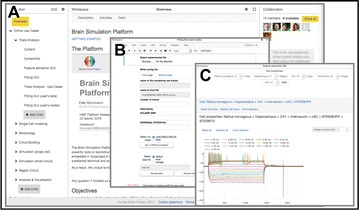


**Figure 1.** The HBP Brain Simulation Platform web interface. **A.** BSP Overview web page. **B and C.** synaptic events Fitting and Electrophysiological Feature Extraction GUIs, developed as a jupyter notebook and a web app respectively

**Acknowledgements**

This project has received funding from the *European Union’s Horizon 2020 research and innovation programme* under grant agreement No 720270.

## P236 A Single Pyramidal-Cell and Network Computational Model of the Hippocampal CA3 Region

### Sami Utku Çelikok^1^, Eva M. Navarro-López^2^, Neslihan Serap Şengör^3^

#### ^1^Biomedical Engineering Department, Boğaziçi University, Istanbul, 34342, Turkey; ^2^School of Computer Science, The University of Manchester, Manchester, M13 9PL, UK; ^3^Electronics and Communication Department, Istanbul Technical University, Istanbul, 34469, Turkey

##### Correspondence: Sami Utku Çelikok (utku.celikok@boun.edu.tr)

*BMC Neuroscience* 2017, **18** (**Suppl 1**):P236

Hippocampal subarea CA3 has long drawn attention for its major role in encoding spatial representations and episodic memories [1]. Due to the presence of rich recurrent feedback connections, CA3 has been considered to play a key role in long-term memory formation. Moreover, CA3 has long been proposed as an auto-associative network capable of pattern completion and path integration for the retrieval and storage of episodic/declarative memory traces [2]. A broad range of experimental studies have supported the idea that hippocampal oscillations must be taken into consideration while investigating the region as a memory network. Empirically-validated studies on freely moving rats have identified two major oscillatory patterns of hippocampal activity in a behaviour-dependent context: theta- (4–8 Hz) and gamma-band (30–100 Hz) frequency rhythms [3, 4]. In rodents and humans, gamma rhythms embedded into theta oscillations become prominent during memory functions, object exploration, and spatial navigation [1]. The consideration of the spiking patterns of the neurons during oscillatory regimes is key to uncover the significance of hippocampal network oscillations in different processes. When the broad electrophysiological repertoire of CA3 pyramidal cells is considered, the computational description of the network requires a neural model. This model has to be simple enough to support a large hippocampal network, but still rich enough to capture complex pyramidal-cell dynamics. This is precisely what we propose here: a single-cell computational model for a CA3 pyramidal neuron that is used as the basic element to form a CA3 network model which will be able to reproduce key hippocampal oscillatory patterns. The spiking patterns of the offered single-cell model capture some essential features of well-known hippocampal spiking behaviour, such as: spike broadening at the end of a burst, rebound bursting, low-frequency bursts, and high-frequency tonic spiking (Figure 1). Moreover, the model for the CA3 population is also able to generate theta and gamma-band oscillations, known to be present in the CA3 region.
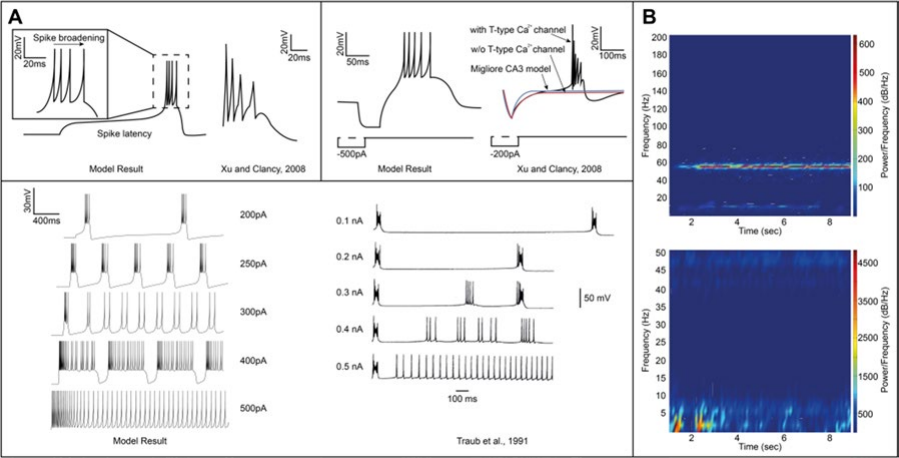


**Figure 1. A.** Single-cell model results. Upper-left: Initial spike generation, upper-right: rebound bursting in response to hyperpolarisation, bottom: burst-to-tonic spike transition with increased input current. **B.** Population model spectrograms. Upper: gamma-band oscillations in the network, bottom: theta-band oscillations in the network

**References**

1. O’Keefe J, Nadel, L: *The Hippocampus as a Cognitive Map.* Oxford, UK: Oxford University Press; 1978.

2. Samsonovich A, McNaughton BL: Path integration and cognitive mapping in a continuous attractor neural network model. *J Neurosci* 1997, **17(15):**5900–5920.

3. Gloveli T, Kopell N, Dugladze T: Neuronal activity patterns during hippocampal network oscillations in vitro. *In: Hippocampal Microcircuit* 2010, *Springer* 247–276.

4. Leung LS, Lopes da Silva F, Wadman WJ: Spectral characteristics of the hippocampal EEG in the freely moving rat. *Clin Neurophysiol* 1982, **54**:203–219.

## P237 Functional connectivity between prefrontal cortex and striatum showed by computational model

### Rahmi Elibol, Neslihan Serap Sengor

#### Electronics and Communication Engineering, Istanbul Technical University, Istanbul, Turkey

##### Correspondence: Rahmi Elibol (rahmielibol@itu.edu.tr)

*BMC Neuroscience* 2017, **18** (**Suppl 1**):P237

It is well-known that there is a strong correlation between cortex and striatal activity especially during progression of action selection and goal directed behavior. This interaction between cortex and striatum project back to the cortex through direct and indirect pathways and over thalamus forming a closed loop [1]. Such structural associations of the brain are called structural connectivity or connectome. Due to the development of measurement technologies as fMRI, more work has been carried to build up the association between the different areas of the brain and the cognitive processes, and such associations are called functional connectivity or functional connectome. Besides these, the processes carried out at neuronal level and/or the changes at synaptic connections which give rise to relations that are observed at frequency and/or phase levels is called dynome [2]. The structural connection between cortex and striatum is already known and their functional connectivity has been shown with experimental studies. In this work, based on the experimental results given in [3], a computational model is proposed based on the dynamical connection of neurons and synapses showing the dynome relation between cortex and striatum.

During the experimental studies that have been explained in [3], LFP in prefrontal cortex and striatum are measured. Beta and gamma frequency bands have been observed and with PLV, the correlation between cortical and striatal activity has been shown [3, 4]. These experimental results have been recreated with the computational model proposed and it is shown that the results given in Figure 1 are similar to the experimental results. The simulations are carried out by considering the similar conditions considered in experiments. The stimuli are applied as in the experimental work and the role of different reward quantities is investigated by changing the dopamine levels.
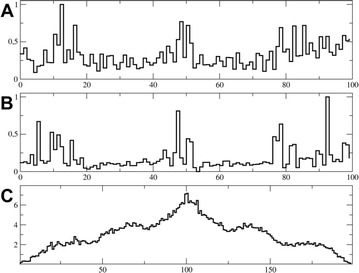


**Figure 1.** The correlation between the PFC and striatal activity: A. The activity in PFC B. The activity in striatum, C. The correlation between PFC and striatum. The activities in PFC and striatum are given with normalized firing rate values. The results show the there is a correlation between cortex and striatum

**References**

1. GE Alexander, MD Crutcher, MR DeLong, Basal ganglia-thalamocortical circuits: Parallel substrates for motor, oculomotor, “prefrontal” and “limbic” functions. *Progress in Brain Research*, 85, 119–146, http://dx.doi.org/10.1016/S0079-6123(08)62678-3.

2. NJ Kopell, HJ Gritton, MA Whittington, MA Kramer: Beyond the connectome: the dynome. *Neuron* 2014, 83(6):1319–1328. doi: 10.1016/j.neuron.2014.08.016.

3. Y Zhang, X Pan, R Wang, M Sakagami: Functional connectivity between prefrontal cortex and striatum estimated by phase locking value. *Cogn Neurodyn*. 2016, 10(3):245–254. doi: 10.1007/s11571-016-9376-2.

4. EG Antzoulatos, EK Miller: Increases in functional connectivity between prefrontal cortex and striatum during category learning. *Neuron* 2014, 83(1):216–225. doi: 10.1016/j.neuron.2014.05.005.

## P238 A spiking neural network model of basal ganglia-thalamocortical circuit with Brian2

### Mustafa Yasir Özdemir, Neslihan Serap Şengör

#### Electronic-Communication Department, İstanbul Technical University, İstanbul, Turkey

##### Correspondence: Mustafa Yasir Özdemir (musyasoz@gmail.com)

*BMC Neuroscience* 2017, **18** (**Suppl 1**):P238

Basal ganglia circuit which is located in the midbrain has an essential role in action selection, decision making and reward based learning processes. In this work, especially basal ganglia-thalamocortical circuit responsible for motor control giving rise to voluntary movement is considered.

The characteristics of neuronal activity and their functional abilities, properties of synaptic connections, effect of neurotransmitters as dopamine and the relation between different nuclei defined by pathways, all these are effective in realizing voluntary movement. It is long known that abnormalities in dopamine level influence basal ganglia operations negatively giving rise to neurological disorders like Parkinson’s Disease, Hungtinton’s chorea, hemiballismus, dystonia [1].

The equations written for neuronal activity are complicated and simulations of computational models are especially versatile to predict the neuronal activity. Computational models reflect the consequences of various assumptions made in forming the models [2]. Most computational models of basal ganglia circuits consider a specific process and only partly reflect their nature and function. In this work, an attempt is made to obtain a holistic model of basal ganglia-thalamocortical circuit in Brian 2 environment to ease the further improvement and testing of the model by the neuroscientist.

Here a spiking neural network model is realized to configure the entire properties of basal ganglia circuit. The characteristic neuronal activities of each substructure are obtained by modification of Izhikevich neuron model [3]. The proposed model of basal ganglia-thalamocortical circuit is also capable of showing the dopamine effect on the processes due to the modified striatum neurons. Medium spiny neurons which have different dopamine receptors are considered in the model separately. Also, direct, indirect and hyper-direct pathways exist in the model and effect of dopamine on these pathways can be observed in the simulations. Synaptic connections configured to realize learning and probability of connections are set according to the research presented in the literature. The model is formed with inspiration from another study [4] and realized on Brian2 simulator.

The simulation results of the model are given by raster plots, firing rates and time-frequency analysis. The stimulus activity in the cortex is projected to the thalamus in the simulations and the model reveals the role of direct, indirect and hyper-direct pathways on the formation of this projection separately.

**References**

1. Wichmann T, DeLong MR: Deep Brain Stimulation for Neurologic and Neuropsychiatric Disorders. *Neuron* 2006, **52(1):** 197–204.

2. Schroll H, Hamker FH: Computational models of basal-ganglia pathway functions: focus on functional neuroanatomy. *Frontiers in Sys Neu* 2013, doi:10.3389/fnsys.2013.00122.

3. Izhikevich EM: Which Model to Use for Cortical Spiking Neurons? IEEE Trans Neural Networks 15:1063–1070.

4. Çelikok U, Navarro-Lopez EM, Şengör NS: A computational model describing the interplay of basal ganglia and subcortical background oscillations during working memory processes. arXiv:1601.07740

## P239 Coordinate-transformation spiking neural network for spatial navigation

### Tianyi Li, Angelo Arleo, Denis Sheynikhovich

#### Sorbonne Universités, UPMC Univ Paris 06, INSERM, CNRS, Institut de la Vision, 17 rue Moreau, 75012 Paris, France

##### Correspondence: Denis Sheynikhovich (denis.sheynikhovich@upmc.fr)

*BMC Neuroscience* 2017, **18** (**Suppl 1**):P239

Spatial navigation in primates is thought to be mediated by neural networks linking the dorsal visual pathway (including parietal and retrosplenial cortices) and the medial temporal lobe [1]. Neurons along this pathway are sensitive to visual cues of varying complexity (from simple visual features to views of spatial scenes [2, 3]) and have been characterized to code environmental features in different reference frames (from egocentric eye- or head-centered representations early in the pathway to allocentric world-centered ones later in the pathway [3, 4]). However, neural mechanisms underlying the transformation between egocentric-visual and allocentric-spatial representations remain poorly understood.

In this work, we present a spiking-neural-network model of visuo-spatial coordinate transformation that receives input in the form of realistic head-centered visual input with limited view field. After processing this input with V1-like orientation-sensitive neuronal filters, it is transformed to an allocentric directional frame using two mechanisms, experimentally observed along the dorsal pathway. First, head direction signal, thought to be provided by the retrosplenial cortex, is used by the network to align egocentric input views with a world-centered directional frame [4]; Second, short-term visual working memory in the parietal network serves to link subsequent views during head rotation into scene-like representation of visual features. The output of the coordinate-transformation network serves as input to the hippocampus, where location-sensitive neuronal responses are learned using spike-timing-dependent plasticity.

Neuronal activities in the model are shown to reproduce basic features of dorsal-pathway neurons. In particular, in an experimental setup mimicking an animal sitting in front of a screen, visual receptive fields of model parietal/retrosplenial neurons code features in head- or world-centered reference frames, and firing activities in the transformation network exhibit gain fields with respect to head direction, as observed in classical experiments with monkeys. In a setup where the simulated animal explores an experimental environment, modeled hippocampal cells exhibit location-sensitive firing fields after learning. These purely visual place fields are influenced by changes in the visuo-spatial environmental layout (e.g. its spatial geometry [5]), and are modulated by currently observed view [2]. Moreover, spike synchrony patterns in this model reflect environment topology [6]. This model links the processing of low-level visual features in the brain with high-level cognitive processes implicated in spatial navigation.

**Acknowledgements**

This research was supported by ANR - Essilor SilverSight Chair ANR-14-CHIN-0001

**References**

1. Kravitz DJ, Saleem KS, Baker CI, Mishkin M: A new neural framework for visuospatial processing. *Nat Rev Neurosci.* 2011, **12:**217–230.

2. Ekstrom AD: Why Vision is Important to How We Navigate. *Hippocampus* 2015, **25:**731–735.

3. Snyder LH, Grieve KL, Brotchie P, Andersen R: Separate body- and world-referenced representations of visual space in parietal cortex. *Nature* 1998, **394:**887–891.

4. Byrne P, Becker S, Burgess N: Remembering the past and imagining the future: A neural model of spatial memory and imagery. *Psychol Rev.* 2007, **114:**340–375.

5. Sheynikhovich D, Chavarriaga R, Strösslin T, Arleo A, Gerstner W, Strosslin T, Arleo A, Gerstner W: Is there a geometric module for spatial orientation? Insights from a rodent navigation model. *Psychol Rev.* 2009, **116:**540–566.

6. Curto C, Itskov V: Cell Groups Reveal Structure of Stimulus Space. *PLoS Comput Biol.* 2008, **4:**e1000205.

## P240 Micro-connectomics with cognitive task selectivity

### Akihiro Nakamura^1,^ Masanori Shimono^1,2^

#### ^1^Osaka University, Toyonaka, Osaka, Japan; ^2^Riken Brain Science Institute, Saitama, Japan

##### Correspondence: Masanori Shimono (smn@bpe.es.osaka-u.ac.jp)

*BMC Neuroscience* 2017, **18** (**Suppl 1**):P240

Various cognitive functions of our brain are realized by interactions among a large number of neurons. Traditionally, the selectivity of neuronal activity to individual cognitive tasks has been studied [1]. In order to understand the function of the brain more deeply, we need to investigate the micro-connectome, which is a comprehensive map of connectivity or interactions of neurons or synapses, beyond the basic statistical observations of its individual elements [2]. This study reports the interactions among neurons measured from the anterior lateral motor cortex (ALM) of mice using calcium fluorescence imaging and focuses on selectivity for cognitive planning of directed licking behaviors [3]. We reconstructed the functional networks from the spiking activities of the neuron ensembles at resting periods and compared them with the motion-selectivity of individual neurons (Figure 1). The network structure was characterized using graph theory [4]. Past studies [3] have declared that significant activities can be observed in layer 5 of the ALM. However, the contributions of different layers were not reported. Our connectome analyses also consistently showed that, in layer 5 of the ALM, a simple *connection strength* measure in motion-selective neurons was significantly stronger than in motion-nonselective cells. Surprisingly, in layer 2, a *Centrality measure* was significantly higher in selective cells, especially contralateral selective cells, than in non-selective cells. *Centrality* represents that the cell is in an important position within the network. It has been repeatedly reported that the effective connectivity, the estimated neuronal activities recorded using Ca Imaging technique in the resting period, reflects the underlining structural synaptic connectivity fairly well [5]. Therefore, our results suggest that the neurons involved in motor-planning were located at highly central positions in the micro-connectome from the structural design. Because of the position, they will be able to influence a large number of neuropiles within, and probably beyond, the ALM. If we observe the brain more widely, layer 5 exists on the bottom-up information flow that originally came from the thalamus, and layer 2 exists on the top-down information flow relatively close to the output to the thalamus. Therefore, layer 2 in the micro-connectome may represent a different functional role of the motor-planning than the neuron group existing in layer 5. Our findings and methodological schemes will contribute to a more accurate understanding of cognitive functions, the effects of aging, and various neurodegenerative diseases.
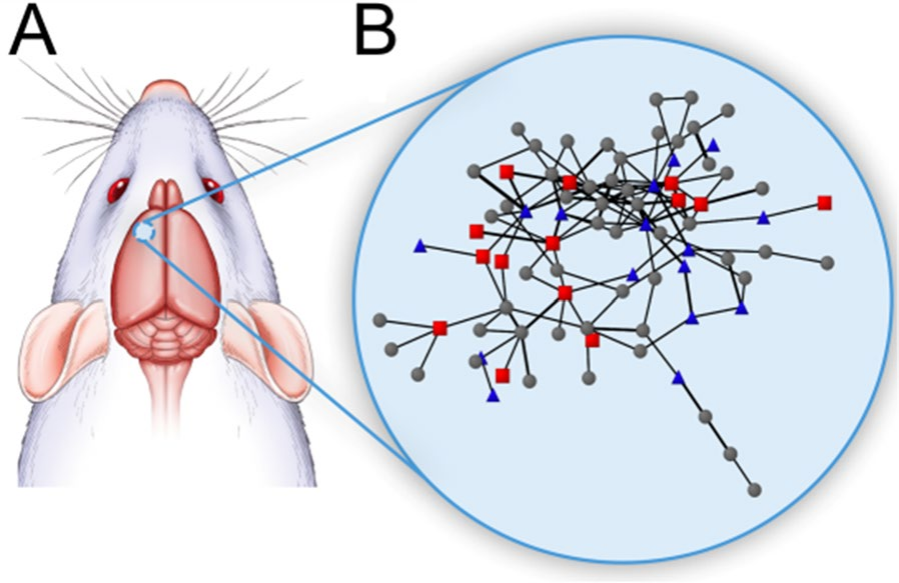


**Figure 1**. The general concept of this study. **A.** Neuronal activities when rodents are taking rest (or just waiting a task) or when performing licking tasks were recorded using Ca Imaging technique. **B.** is an example of effective/functional networks of neurons reconstructed from the neuronal dynamics. The differences of markers show differences of responses of neurons. (Neurons responding selectively to contralateral lickings (△), to ipsilateral lickings (□), and neurons showing no responses to these licking behaviors (○))

**References**

1. Hubel DH, Wiesel TN: Receptive fields and functional architecture of monkey striate cortex. *The Journal of physiology* 1968, **195(1):** 215–243.

2. Shimono M, Beggs, JM: Functional clusters, hubs, and communities in the cortical microconnectome. *Cerebral Cortex* 2015, ***25*** **(10):** 3743–3757.

3. Li, N, Chen, TW, Guo ZV, Gerfen CR, Svoboda, K.: A motor cortex circuit for motor planning and movement. *Nature* 2015, **519(7541):** 51–56.

4. Bullmore, E., Sporns, O.: Complex brain networks: graph theoretical analysis of structural and functional systems. *Nature Reviews Neuroscience* 2009, **10(3)**: 186–198.

5. Stetter O, Battaglia D, Soriano J, Geisel T: Model-free reconstruction of excitatory neuronal connectivity from calcium imaging signals. *PLoS Comput Biol* 2012, **8(8):** e1002

## P241 Does reinforcement learning explain zone-allocation behavior between two competing mice?

### Youngjo Song^1^, Sol Park^1,2^, Ilhwan Choi^2^, Jaeseung Jeong^1,3^, Hee-sup Shin^2^

#### ^1^Department of Bio and Brain Engineering, KAIST, Daejeon, 34141, Republic of Korea; ^2^Center for Cognition and Sociality, IBS, Daejeon, 34047, Republic of Korea; ^3^Program of Brain and Cognitive Engineering, KAIST, Daejeon, 34141, Republic of Korea

##### Correspondence: Youngjo Song (jsjeong@kaist.ac.kr)

*BMC Neuroscience* 2017, **18** (**Suppl 1**):P241

In the previous study (Choi et al., in revision), we observed two mice showing cooperative-like behavior in the competitive situation over rewards. We have also shown that this cooperative-like behavior enhanced mutual rewords and produced payoff equity between two competing mice. However, the origin of this behavior is not clear. Thus, the aim of this study is to address whether the cooperative-like behavior could be explained by reinforcement learning or not. In the behavior chamber for mice, two light cues which indicate two reward zones, respectively. If a mouse goes the left reward zone when the left light cue turns on, the mouse gets reward, and a mouse can get rewards if the mouse get in the right reward zone when the right light cue turns on. The reward is given by wireless brain stimulation from the electrode implanted in the Medial forebrain bundle (MFB), the pleasure center in the mouse brain. When the mice learned the meaning of light cues, we performed the pair test in which the two mice released in one training chamber. In this experiment, 15 out of 19 pairs showed the tendency to separate and allocate their own reward zone by themselves. In other words, those mice had their own preferred sides and did not interfere opponent’s preferred side (we called this behavior as ‘zone-allocation behavior’). We followed the ethical guidelines of the Institutional Animal Care and Use Committee in the KAIST. This behavior could be considered as a heuristic rule of reciprocity and cooperation. To investigate if the reinforcement learning can explain this behavior in two competing mice, we developed computational model based on the Temporal difference (TD) learning model. In this computational simulation, the environment is set up identically with the real training room. The model mouse makes decisions only based on a state-action value function which is updated by the TD rule. We found that the computational model successfully mimicked the zone allocating behavior between two model mice. Two types of pairs in our model were observed. The first type is a pair dividing their own reward zone each other, which indicates each mouse obtained its own preferred side (Figure 1A). This can be thought as a case of zone allocating pair in actual experiment. The second type is that one mouse dominates both side of reward zone (Figure 1B). From repetitive iterations, we obtained 75% of model mouse pairs showing the zone allocating behavior, which is quite consistent with the experimental results of the real zone-allocating pair ratio (69%). Moreover, we examined whether a mouse achieve this behavior when it uses model-based learning. We used Dyna-Q algorithm to implement this model mouse. Zone allocating behavior, however, could not be achieved. If it uses model-based learning, it updates its state-action value too often. Therefore, the mouse’s behavior did not converge.
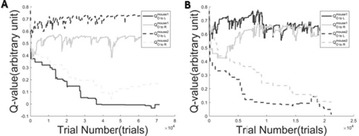


**Figure 1. A.** State-action values (Q-value) of a pair of mice showing zone-allocation behavior. In mouse_1_, Q-value for R(right) reward zone is larger than Q-value for L(left) reward zone. It means that mouse_1_ prefer R reward zone. In the same way, mouse_2_ prefer L reward zone. Moreover, Q-value of mouse_1_ for L reward zone and Q-value of mouse_2_ for R reward zone becomes less than 0.2. It means that each mouse didn’t interfere opponent’s preferred side. **B.** State-action values (Q-value) of a pair of mice not showing zone-allocation behavior. Q-value of mouse2 for reward zone is converging to zero. It means that mouse_2_ prefer not to move, so mouse_1_ got all the reward

**Conclusion:** This computational result supports the hypothesis that the zone allocating rodent behavior can be explained from positive reinforcement learning (particularly model-free learning). Zone-allocation might be a strategy to maximize reward and to minimize cost in aspect of reinforcement learning in competitive situation. We suggest that, to investigate the social heuristic behavior, it might be crucial to remove convergent egoistic characteristic of animal behavior.

**References**

1. Richard S. Sutton, Andrew G. Barto:Reinforcement Learning: An Introduction. The MIT press; 1998.

2. Paul W. Glimcher, Ernst Fehr: *Neuroeconomics, 2* ^*nd*^ *Edition*. Academic press; 2014

## P242 Optimal synaptic scaling emerges from Hebbian learning rules in balanced networks

### Sadra Sadeh^1^, Padraig Gleeson^1^, R. Angus Silver^1^

#### ^1^Department of Neuroscience, Physiology and Pharmacology, University College London, London WC1E 6BT, UK

##### Correspondence: Sadra Sadeh (s.sadeh@ucl.ac.uk)

*BMC Neuroscience* 2017, **18** (**Suppl 1**):P242

Synaptic connectivity varies widely across cell types and brain regions and connections are formed and lost during development and learning. However, normal function cannot be maintained by simply adding or subtracting excitatory synaptic inputs onto a neuron, since this will cause neurons to become hyper- or hypo-excitable, resulting in network instability and loss of function. How then do neurons scale their synaptic input to maintain function? Theoretical work suggests that the optimal way of scaling of synaptic weights (J) as the number of synaptic connections per neuron (degree, K) is J ~ 1/√K [1], a result that has recently been confirmed experimentally [2]. However, the mechanisms by which such optimal scaling arises are unknown. To address this question, we implemented Hebbian-like plasticity rules at excitatory (E) and inhibitory (I) synapses in large-scale balanced spiking networks of primary visual cortex [3]. As K was increased in the networks we found that synaptic weight decreased with a dependence of J = 1/K^0.6^, close to the theoretically optimal scaling [1] and closely matching that found experimentally [2]. Interestingly, optimal synaptic scaling emerged when Hebbian plasticity was present at both E and I synapses. In contrast, spiking networks relying solely on plasticity of I → E synapses to balance excitation and inhibition [4] did not exhibit optimal scaling. A simplified mean-field analysis of network dynamics explained the dependence of J on K in networks with Hebbian-like plasticity of E and I synapses, while revealing why the optimal scaling does not always hold in networks with plasticity of only I → E synapses.

Irrespective of the initial weights and number of synaptic connections, spiking networks with Hebbian-like plasticity of E and I synapses robustly self-regulated themselves through recurrent inhibition and learning into a low activity regime where the activity of the E neuronal population exhibited a long tail of activity. Notably, this was accompanied by higher activity and lower selectivity of I neurons, consistent with experimental observations. Examination of the input-output relationship of individual current-based or conductance-based neurons revealed that optimal synaptic scaling robustly preserved neuronal gain as the number of synaptic inputs was altered. Moreover, contrast-invariant input tuning curves translated to contrast-invariant output tuning curves only when the optimal (1/√K) scaling of weights was preserved. Our results thus suggest that Hebbian learning in both E and I connections is necessary for preserving cortical computation and function during changes in synaptic connectivity. These findings have important implications for cortical function during development, and cortical dysfunction during brain diseases.

**Acknowledgements**

yFunded by the Wellcome Trust and the ERC.

**References**

1. van Vreeswijk C, Sompolinsky H: Chaos in neuronal networks with balanced excitatory and inhibitory activity. *Science* 1996, **274(5293):**1724–1726.

2. Barral J, Reyes AD: Synaptic scaling rule preserves excitatory-inhibitory balance and salient neuronal network dynamics. *Nat Neurosci* 2016, **19(12)**:1690–1696.

3. Sadeh S, Clopath C, Rotter S: Emergence of Functional Specificity in Balanced Networks with Synaptic Plasticity. *PLoS Comput Biol* 2015, **11(6)**: e1004307.

4. Vogels TP, Sprekeler H, Zenke F, Clopath C, Gerstner W: Inhibitory Plasticity Balances Excitation and Inhibition in Sensory Pathways and Memory Networks. *Science* 2011, **334(6062)**:1569–1573.

## P243 Deciphering the contributions of oriens-lacunosum/moleculare (OLM) cells during local field potential (LFP) theta rhythms in CA1 hippocampus

### Alexandra Pierri Chatzikalymniou^1,2^ Frances K. Skinner^1,3,2^

#### ^1^Krembil Research Institute, University Health Network, Toronto, ON, Canada; ^2^Department of Physiology, University of Toronto, Toronto, ON, Canada; ^3^Department of Medicine (Neurology), University of Toronto, Toronto, ON, Canada

##### Correspondence: Alexandra Pierri Chatzikalymniou (alexandra.chatzikalymniou@mail.utoronto.ca)

*BMC Neuroscience* 2017, **18** (**Suppl 1**):P243

In the hippocampus, one of the most prevalent LFP rhythms is the 3–12 Hz “theta” oscillation [1]. This LFP theta rhythm is tightly correlated with spatial navigation, episodic memory and rapid eye movement (REM) sleep [1]. Recent work by Goutagny and colleagues [4] showed that theta rhythms emerge in the CA1 region of an intact in vitro hippocampus preparation due to local interactions between hippocampal interneurons and pyramidal (PYR) cells. Oriens-lacunosum/moleculare (OLM) cells are a major class of GABAergic interneurons in the hippocampus [5]. In addition to inhibiting distal dendrites of PYR cells in stratum LM, OLM cells disinhibit PYR cells in stratum radiatum, an inner to middle layer, by inhibiting interneurons that target PYR cells in that region [5].

Our goal is to examine the contributions of OLM cells to ongoing LFP theta rhythms in the context of the intact in vitro preparation using computational modeling. We use network models of OLM cells, bistratified cells (BiCs), and basket/axo-axonic cells (BC/AACs) that target PYR cells in specific layers [3], and assess the role of OLM cells as their interactions with BiCs and the PYR cell vary. We find that the LFP power is mostly affected by changes in the synaptic conductance from OLM cells to BiCs rather than by synaptic conductance changes from BiCs to OLM cells, indicating a more important role for the former. This observation suggests that progressive inhibition of OLM cells and thus progressive decrease of their synaptic inputs onto the PYR cell does not strongly alter LFP characteristics whereas progressive inhibition of BiCs does. Decomposition of the LFP signal reveals that fluctuations in power occur due to BiC and BC/AAC synaptic inputs onto the PYR cell rather than to OLM cell synaptic inputs onto the PYR cell. Selective removal of either OLM cells or BiCs/BCs/AACs reveal minimal contribution of the OLM cells to the total LFP power across the dendritic tree. Conversely, the BiCs/BCs/AACs generated LFP component comprises approximately 90% of the total signal. Furthermore, changes in synaptic weights from OLM cells to the PYR cell do not produce substantial changes in the LFP.

Brain rhythms can be considered as representations of brain function [1, 2]. Given that particular inhibitory cell populations and abnormalities in theta rhythms are associated with disease states [2], it is important to understand the cellular contributions to LFP theta rhythm modulations. Our results show that OLM cells prominently contribute to local LFP theta through their interactions with other local inhibitory cell types. Decomposition of the LFP reveals little contribution of synaptic inputs from OLM cells onto the PYR cell. In CA1 PYR cells, distal and middle apical dendrites comprise two distinct dendritic domains with separate branching [6]. Since we find that maximum LFP power is recorded around the soma and the proximal dendrites, OLM cell contributions to LFP theta can be understood in the context of the cytoarchitectonic separation of the of distal and proximal dendrites in PYR cells which prohibits distal inhibitory inputs from effectively propagating to the soma.

**Acknowledgements**

Supported by NSERC Canada, Margaret J. Santalo Fellowship (Physiology, Univ Toronto) and SciNet HPC.

**References**

1. Buzsáki G: Theta oscillations in the hippocampus. *Neuron* 2002, **33:**325–340.

2. Colgin L: Rhythms of the hippocampal network. *Nat Neurosci Rev* 2016, **17**:239–249.

3. Ferguson KA, Huh CYL, Amilhon B, Williams S, Skinner FK: Network models provide insight into how oriens-lacunosum-moleculare (OLM) and bistratified cell (BSC) interactions influence local CA1 theta rhythms. *Front Syst Neurosci* 2015, 9:110.

4. Goutagny R, Jackson J, Williams S: Self-generated theta oscillations in the hippocampus. *Nat Neurosci* 2009, **12:**1491–1493.

5. Maccaferri G: Stratum oriens horizontal interneurone diversity and hippocampal network dynamics. *J Physiol* 2005, **562.1**:73–80.

6. Spruston N: Pyramidal neurons: dendritic structure and synaptic integration. *Nature Neurosci Rev* 2008, **9:**206.

## P244 Nonlinear optimal control of brain networks

### Lazaro M. Sanchez-Rodriguez, Roberto C. Sotero

#### Hotchkiss Brain Institute and Department of Radiology, University of Calgary, Calgary, Alberta, Canada, T2 N 1N4

##### Correspondence: Lazaro M. Sanchez-Rodriguez (lazaro.sanchezrodrgu@ucalgary.ca)

*BMC Neuroscience* 2017, **18** (**Suppl 1**):P244

The problem of controlling brain networks has been the focus of several recent studies given its relationship to brain stimulation. In this work, we introduce the State-Dependent Ricatti Equation formalism (SDRE) [1] for the computation of optimal control signals in nonlinear brain networks. Firstly, the optimal input for the abatement of epileptic-like activity in the model proposed in [2] was calculated (see Figure 1B). Additionally, we looked at higher dimensional systems consisting of coupled autonomous Duffing oscillators (see Figure 1, panels C-E). In the linear case our results are in agreement with those obtained in [3]. However, as the strength of the non-linearity increases, the fraction of the networks that can be controlled is generally lower whereas the cost of controlling the systems grows. Thus, we find evidence for supporting the use of realistic nonlinear modeling of electrical neural activity in the design of optimal controllers for brain networks.
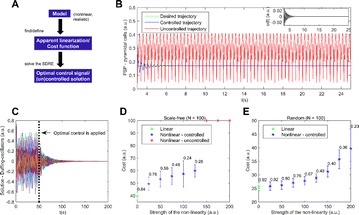


**Figure 1.** SDRE-optimal control of the networks. **A.** General scheme. **B.** Controlling the model in [2]. As soon as the control signal (top right corner) is sent, the diseased solution –in red– is derived to normal background activity. **C.** Typical trajectory for a controlled network of autonomous Duffing oscillators coupled through a scale-free connectivity matrix. Stimuli are inputted over the nodes with lower degree –third part of the total number of nodes in the network. **D.** Expected cost for the control over 25 scale-free networks (N = 100, mean degree ≈ 6). The numbers over each of the error bars indicate the fraction of the realizations of the network in which control is achieved as the non-linearity (coefficient of the cubic term) is changed. For strengths past 125, none of the networks can be controlled. In this case, the costs are infinitely high in theory. They are represented as red asterisks at the top of the panel. **E.** Analogue to **D** for randomizations of the previously computed scale-free networks

**References**

1. Jayaram A, Tadi M: Synchronization of chaotic systems based on SDRE method. *Chaos Solitons Fractals* 2006, **28**:707–715.

2. Taylor PN, Thomas J, Sinha N, Dauwels J, Kaiser M, Thesen T, Ruths J: Optimal control based seizure abatement using patient derived connectivity. *Front. Neurosci* 2015, **9**:1–10.

3. Liu YY, Slotine JJ, Barabási AL: Controllability of complex networks. *Nature* 2011, **473**:167–173.

## P245 An inhibitory microcircuit that amplifies the redistribution of somatic and dendritic inhibition

### Loreen Hertäg^1^, Owen Mackwood^1^, Henning Sprekeler^1^

#### ^1^Modelling of Cognitive Processes, Berlin Institute of Technology and Bernstein Center for Computational Neuroscience, Berlin, 10587, Germany

##### Correspondence: Loreen Hertäg (loreen.hertaeg@tu-berlin.de)

*BMC Neuroscience* 2017, **18** (**Suppl 1**):P245

GABAergic interneurons constitute only a small fraction of neurons in the brain, but their importance for brain function is undeniable [1]. Moreover, they display a large diversity in their biophysical, physiological and anatomical properties [2], suggesting a functional ‘division of labor’. However, the computational roles of the various interneuron types and how they are supported by their individual properties is largely unknown.

A striking difference between inhibitory cell types is that they form synapses onto different compartments of their postsynaptic targets. Parvalbumin- (PV) and somatostatin (SOM)-expressing interneurons, in particular, seem to predominantly target the perisomatic regions and the dendrites, respectively. As SOM and PV cells are also connected, it has been suggested that inhibition can be dynamically redistributed between the dendrites and somata of pyramidal cells (PCs) [3, 4]. Here, we argue that a different cortical sub-circuit consisting of SOM- and vasoactive intestinal peptide (VIP)-expressing interneurons is optimized to control this redistribution by amplifying small top-down control signals.

To support this hypothesis, we performed a mathematical analysis and simulations of a network model comprising excitatory PCs and inhibitory PV, SOM and VIP neurons. The connectivity in the circuit was chosen according to experimental findings [4]. We show that the SOM-VIP circuit can serve as an amplifier that translates small top-down signals onto VIP cells [5, 6] into large changes in the somato-dendritic distribution of inhibition onto PCs. Taken to the extreme, the circuit can generate winner-take-all (WTA) dynamics that implement a binary switch for somato-dendritic inhibition.

Furthermore, we interpret key properties of the SOM-VIP sub-circuit in the light of this hypothesis. We show that the striking lack of recurrent inhibition as well as the presence of short-term synaptic facilitation (STF) observed among VIP and SOM cells strengthens the amplification properties of the network. Artificially including recurrent inhibitory connections within the VIP or SOM populations not only weakens the amplification, but can also lead to pathological conditions in which almost all cells within each population are silenced. These pathological states are not observed when firing rate adaptation is included that is, indeed, a common feature of SOM and VIP neurons.

In summary, our analysis shows that the SOM-VIP sub-circuit is well suited to redistribute inhibition onto soma and dendrites of excitatory PC neurons by amplifying small changes in the input signal to VIP cells. The synaptic and neural properties, including lack of recurrence, presence of STF and firing rate adaptation, underpin this computation by strengthening the amplification properties and/or avoiding pathological states.

**Acknowledgements**

yThe project is funded by the German Federal Ministry for Education and Research, FKZ 01GQ1201.

**References**

1. Isaacson, JF, Scanziani M: How inhibition shapes cortical activity. *Neuron* 2011, **72(2):** 231–243.

2. Tremblay, R, Lee, S, and Rudy, B: GABAergic interneurons in the neocortex: from cellular properties to circuits. *Neuron* 2016, **91(2):** 260–292.

3. Pouille, F, Scanziani, M: Routing of spike series by dynamic circuits in the hippocampus. *Nature* 2004, **429(6993):** 717–723.

4. Pfeffer, CK, Xue, M, He, M, Huang, ZJ, Scanziani, M: Inhibition of inhibition in visual cortex: the logic of connections between molecularly distinct interneurons. *Nature neuroscience* 2013, **16(8):** 1068–1076.

5. Lee, S, Kruglikov, I, Huang, ZJ, Fishell, G, Rudy, B: A disinhibitory circuit mediates motor integration in the somatosensory cortex. *Nature neuroscience* 2013, **16(11):** 1662–1670.

6. Pi, HJ, Hangya, B, Kvitsiani, D, Sanders, JI, Huang, ZJ, Kepecs, A: Cortical interneurons that specialize in disinhibitory control. *Nature* 2013, *503*(7477), 521–524.

## P246 Learning grid cells in recurrent neural networks

### Steffen Puhlmann^1^, Simon N. Weber^1,2^, Henning Sprekeler^1,2^

#### ^1^MKP, Modelling of cognitive processes, Berlin Institute of Technology, 10587 Berlin, Germany; ^2^Bernstein Center for Computational Neuroscience, 10115, Berlin, Germany

##### Correspondence: Steffen Puhlmann (s.puhlmann@campus.tu-berlin.de)

*BMC Neuroscience* 2017, **18** (**Suppl 1**):P246

Grid cells are spatially tuned neurons in the entorhinal cortex, whose spatial firing fields tessellate the environment with a hexagonal lattice. The mechanisms that underlie this highly symmetric firing pattern are currently subject to intense debate [1]. As an alternative to attractor and oscillatory interference models that perform path integration and assume a specific connectivity [1], we recently suggested that grid cells could be learned in a feedforward network by interacting excitatory and inhibitory plasticity on spatially modulated inputs [2]. A central prerequisite for the suggested mechanism is that inhibitory inputs have a broader spatial tuning than their excitatory counterparts. Given that recurrent inhibition is abundant in entorhinal cortex [3] and spatially tuned [4], we reasoned that this broadened inhibition could be the result of recurrent processing.

To corroborate this hypothesis, we analyzed a recurrent network model consisting of excitatory and inhibitory rate neurons. For the sake of the argument, only the excitatory neurons in the network receive external, spatially modulated excitatory input. All synapses in the network are plastic, with Hebbian plasticity on the excitatory synapses and homeostatic plasticity on the inhibitory synapses [5]. When exposing the network to inputs that mimic the movement of an animal on a linear track, a large fraction of cells in the recurrent network rapidly develops a grid-like firing pattern. We find that the underlying mechanism is robust to details of the spatial input tuning and that the spatial scale of the resulting grids is primarily determined by the spatial autocorrelation length of inputs. Based on insights from earlier work on the interaction of excitatory and inhibitory synaptic plasticity [6, 2], we identify key mechanisms in the circuit that are required for the formation of grid cells: 1) a smooth, saturating nonlinearity in the interneurons, which ensures that their spatial tuning is broader than the tuning of their excitatory drive, and 2) sufficiently many and diverse excitatory inputs to the inhibitory neurons.

Based on these findings, we suggest that grid cells could be bootstrapped from a large variety of spatially modulated excitatory inputs to a recurrent network of excitatory and inhibitory neurons with synaptic plasticity on all synapses.

**Acknowledgements**

The project is funded by the German Federal Ministry for Education and Research, FKZ 01GQ1201.

**References**

1. Giocomo LM, Moser MB, Moser EI: Computational models of grid cells. Neuron 2011, **71(4):**589–603.

2. Weber SN, Sprekeler H: Learning place cells, grid cells and invariances: A unifying model. bioRxiv 2017, 102525.

3. Couey JJ, Witoelar A, Zhang SJ, Zheng K, Ye J, Dunn B, Czajkowski R, Moser MB, Moser EI, Roudi Y, et al.: Recurrent inhibitory circuitry as a mechanism for grid formation. Nat Neurosci 2013, **16(3):**318–324.

4. Buetfering C, Allen K, Monyer H: Parvalbumin interneurons provide grid cell-driven recurrent inhibition in the medial entorhinal cortex. Nat Neurosci 2014, **15(5):**710–718.

5. Vogels TP, Sprekeler H, Zenke F, Clopath C, Gerstner W: Inhibitory plasticity balances excitation and inhibition in sensory pathways and memory networks. Science 2011, ***334*** **(6062):**1569–1573.

6. Clopath C, Vogels TP, Froemke RC, Sprekeler H: Receptive field formation by interacting excitatory and inhibitory synaptic plasticity. bioRxiv 2016, 066589.

## P247 A model of perceptual learning, biases, and roving

### David Higgins^1,2^, Henning Sprekeler^1,2^

#### ^1^Modelling of Cognitive Processes, TU Berlin, 10587, Germany; ^2^Bernstein Center for Computational Neuroscience, Berlin, 10115, Germany

##### Correspondence: David Higgins (dave@uiginn.com)

*BMC Neuroscience* 2017, **18** (**Suppl 1**):P247

Roving is a random task-sequencing paradigm, in perceptual learning, whereby multiple tasks are learned in a randomly interleaved sequence. For certain experiments, such as bisection tasks, human subjects appear to be unable to learn the individual tasks under roving conditions [1]. In general, theoretical descriptions of perceptual learning experiments have resorted to approaches involving tuning of inputs, using either recurrence or suppression [2, 3]. However, these approaches have exhibited only partial success in tackling roving. In 2012, Herzog et al. [4] proposed a theoretically inspired explanation involving a constant drift in synaptic efficacies in the system (unsupervised bias), due to an inability to maintain accurate task specific estimates of performance. This leads to a failure to learn using feedback. We update this approach, adding additional features, which though adding realism tend to counteract the action of the unsupervised bias. We then use this model to examine whether the unsupervised bias is sufficient to explain roving or not.

The proof-of-concept model proposed in Herzog et al. [4] does indeed lead to a failure to correctly learn during roving but, while it fails due to the mooted unsupervised bias in the learning rule, the implementation relies on unbounded weight growth, an unrealistic phenomenon. We introduce a simple weight normalisation term, to counteract the unbounded weight growth, and implement a cognitive bias, often observed in human subjects, towards 50:50 presentation ratios. We thus discover a more appropriate model of human perceptual learning performance. Our model (i) learns correctly on a single bisection or vernier task, (ii) fails to learn during roving of multiple tasks, (iii) exhibits the human tendency towards 50:50 ratios of choice, thus failing when a 75:25 ratio is used, and (iv) correctly learns when informed of the altered presentation ratio, similarly to human subjects (unpublished data). A further extension to the original model, operating on a much slower timescale, allows the task critic system to learn over time to separately identify the tasks. This ultimately leads to learning of the initially unlearnable tasks, as seen in [5].

Our model can be seen as the distillation of the mechanism of failure to learn due to the unsupervised bias. Consistent with intuitions within the perceptual learning community, our model indicates that the degree of overlap in task representations, combined with the unsupervised bias, leads to the difference in outcomes between successful transfer learning versus failure. Interestingly, a cognitive bias in the task presentation ratio appears to be quite helpful in a range of presentation paradigms, often counteracting the unsupervised bias and rescuing potential failures to learn correctly. Our work would combine quite well with the more detailed work of Liu et al. [6] to provide a full model of perceptual learning in the visual system.

**References**

1. Otto, TU, Herzog MH, Fahle M, Zhaoping L: Perceptual Learning with Spatial Uncertainties. *Vision Research* 2006, **46(19):** 3223–3233.

2. Zhaoping, L, Herzog MH, Dayan P: Nonlinear Ideal Observation and Recurrent Preprocessing in Perceptual Learning. *Network* 2003, **14(2):** 233–247.

3. Schäfer, R, Vasilaki E, Senn W: Adaptive Gain Modulation in V1 Explains Contextual Modifications during Bisection Learning. *PLOS Comput Biol* 2009, **5(12):** e1000617.

4. Herzog, MH, Aberg KC, Frémaux N, Gerstner W, Sprekeler H: Perceptual Learning, Roving and the Unsupervised Bias. *Vision Research* 2012, **61:** 95–99.

5. Parkosadze K, Otto TU, Malania M, Kezeli A, Herzog M: Perceptual Learning of Bisection Stimuli under Roving: Slow and Largely Specific. *Journal of Vision* 2008, **8(1):** 5.

6. Liu J, Dosher BA, Lu ZL: Augmented Hebbian Reweighting Accounts for Accuracy and Induced Bias in Perceptual Learning with Reverse Feedback. J*ournal of Vision* 2015, **15(10):** 10–10.

## P248 Presynaptic inhibition provides a rapid stabilization of recurrent excitation in the face of plasticity

### Laura B. Naumann^1,2^, Henning Sprekeler^1,2^

#### ^1^Modelling of Cognitive Processes, Berlin Institute of Technology, Berlin, Germany; ^2^Bernstein Center for Computational Neuroscience, Berlin, Germany

##### Correspondence: Laura B. Naumann (laura-bella.naumann@bccn-berlin.de)

*BMC Neuroscience* 2017, **18** (**Suppl 1**):P248

Synaptic plasticity in recurrent neural networks is believed to underlie learning and memory in the brain. One practical problem of this hypothesis is that recurrent excitation forms a positive feedback loop that can easily be destabilized by synaptic plasticity. Numerous homeostatic mechanisms have been suggested to stabilize plastic recurrent networks [1], but recent computational work indicates that all these mechanisms share a major caveat: An effective rate stabilization requires a homeostatic process that operates on the order of seconds, while experimentally observed mechanisms such as synaptic scaling occur over much longer timescales [2].

Here, we suggest presynaptic inhibition as an alternative homeostatic process, which does not suffer from this discrepancy in timescales. Experimental studies have revealed that excess network activity can trigger an inhibition of transmitter release at excitatory synapses through the activation of presynaptic GABA_B_ receptors, which effectively weakens synaptic strength [3]. This attenuation of recurrent interactions has been observed to be fully reversible and acts on timescales of 100 s of milliseconds, thus constituting a candidate mechanism for the rapid compensation of synaptic changes.

To highlight the beneficial properties of presynaptic inhibition in excitatory recurrent circuits, we analyzed a simple rate-based recurrent network model. Presynaptic inhibition is mimicked by multiplicatively scaling down recurrent excitatory weights in response to excess population activity. Using analytical and numerical methods, we show that presynaptic inhibition ensures a gradual increase of firing rates with growing recurrent excitation, even for very strong recurrence (Fig. 1A). An in-depth mathematical analysis of the underlying dynamical system further reveals that the stability of non-zero fixed points (Fig 1A, filled markers) is largely independent of model parameters. In contrast, classical subtractive postsynaptic inhibition is unable to control recurrent excitation once it has surpassed a critical value (Fig. 1B). Moreover, we investigate the conditions under which presynaptic inhibition can stabilize recurrent networks if Hebbian assemblies are imprinted.

In summary, the multiplicative character of presynaptic inhibition provides a powerful homeostatic mechanism to rapidly reduce effective recurrent interactions while retaining synaptic weights and hence conserving the underlying connectivity. It might therefore set the stage for stable learning without interfering with plasticity at the level of single synapses.
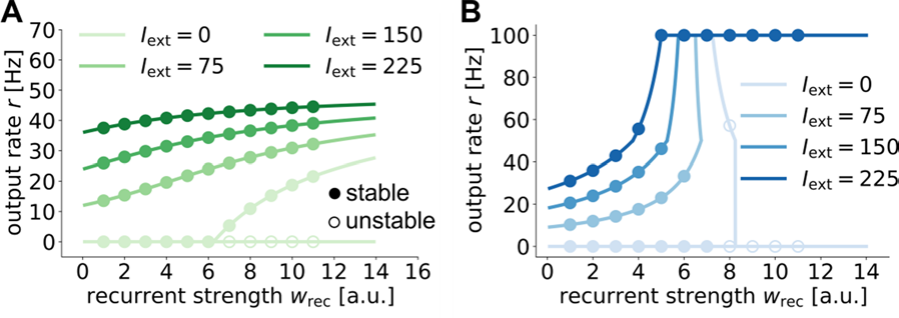


**Figure 1.** Steady state firing rates as a function of recurrent strength for different input intensities *I*_ext_. **A.** presynaptic inhibition **B.** postsynaptic inhibition

**References**

1. Abbott LF, Nelson SB: Synaptic plasticity: taming the beast. *Nat Neurosci* 2000, **3**:1178–1490.

2. Zenke F, Gerstner W: Hebbian plasticity requires compensatory processes on multiple timescales. *Phil Trans R Soc B* 2017, **372(1715):**20160259.

3. Urban-Ciecko J, Fanselow EE, Barth AL: Neocortical Somatostatin Neurons Reversibly Silence Excitatory Transmission via GABAb Receptors. *Curr Biol* 2016, **25(6):**722–731.

## P249 A grid score for individual spikes of grid cells

### Simon N. Weber^1,2^, Henning Sprekeler^1,2^

#### ^1^Berlin Institute of Technology, 10587 Berlin, Germany; ^2^Bernstein Center for Computational Neuroscience, 10115 Berlin, Germany

##### Correspondence: Simon N. Weber (weber@tu-berlin.de)

*BMC Neuroscience* 2017, **18** (**Suppl 1**):P249

The location-specific firing of cells in the entorhinal cortex is subject to extensive experimental and theoretical research. When classifying the tuning properties of entorhinal cells, researchers distinguish between grid cells, i.e., cells whose firing locations form a hexagonal grid, and cells that fire periodically but without hexagonal symmetry [1–3]. This classification requires a measure for the symmetry of spatially modulated firing patterns — a grid score. The most established grid score is computed in multiple stages [e.g., 4]. Spike locations are transformed into a rate map. Subsequently, an autocorrelogram of the rate map is cropped, rotated and correlated with its unrotated copy. The final grid score is obtained from the resulting correlation-vs-angle function at selected angles. This procedure results in a global grid score for the firing pattern, whose exact value depends on the parameter choices required at each stage.

Here we suggest a new approach that computes a local grid score — and the local grid orientation — for each individual spike, directly from spike locations. We compare it to established grid scores and show that it is at least as reliable in quantifying the global grid score of the spike pattern and robust to noise on the spike locations. The score enables the plotting of spike locations, color coded with the local grid score or the local orientation of the grid and could thus simplify the visualization of experimental data. More specifically, it could be used to quantify and highlight recent experimental findings, like boundary effects on the structure of grids in asymmetric enclosures [5], drifts in grid orientation along the arena [6] or the preferred alignment of grids to one of the boundaries [6]. The grid score is applicable to any n-fold symmetry.

We provide a public Python package (using SciPy and NumPy) that efficiently determines the grid score directly from spike locations.

**Acknowledgements**

Funded by the German Federal Ministry for Education and Research, FKZ 01GQ1201.

**References**

1. Krupic J, Burgess N, O’Keefe J: Neural representations of location composed of spatially periodic bands. *Science 2012*, **337(6096):**853–857.

2. Buetfering C, Allen K, Monyer H: Parvalbumin interneurons provide grid cell-driven recurrent inhibition in the medial entorhinal cortex. *Nature Neurosci 2014*, **17(5):**710–718.

3. Kropff E, Carmichael JE, Moser MB, Moser EI. Speed cells in the medial entorhinal cortex. *Nature 2015*, **523(7561)**, 419–424.

4. Sargolini F, Fyhn M, Hafting T, McNaughton BL, Witter MP, Moser MB, Moser EI: Conjunctive representation of position, direction, and velocity in entorhinal cortex. *Science 2016*, **312(5774)**, 758–762.

5. Krupic J, Bauza M, Burton S, Barry C, O’Keefe J: Grid cell symmetry is shaped by environmental geometry. *Nature 2015*, **518(7538)**, 232–235.

6. Stensola T, Stensola H, Moser MB, Moser EI: Shearing-induced asymmetry in entorhinal grid cells. *Nature 2015*, **518(7538)**, 207–212.

## P250 Cortical circuits implement optimal integration of context

### Ramakrisnan Iyer, Stefan Mihalas

#### Allen Institute for Brain Science, Seattle, WA, 98109, USA

##### Correspondence: Stefan Mihalas (stefanm@alleninstitute.org)

*BMC Neuroscience* 2017, **18** (**Suppl 1**):P250

Neurons in the primary visual cortex (V1) predominantly respond to a patch of the visual input, their classical receptive field. These responses are modulated by the visual input in the surround [1]. This reflects the fact that features in natural scenes do not occur in isolation: lines, surfaces are generally continuous, and the surround provides context for the information in the classical receptive field. It is generally assumed that the information in the near surround is transmitted via lateral connections, between neurons in the same area [1]. A series of large scale efforts have recently described the relation between the lateral connectivity and visual evoked responses and found like-to-like connectivity between excitatory neurons [2, 3]. Additionally, specific cell type connectivity for inhibitory neuron types has been described [4]. However current normative models of cortical function rely on sparsity [5], saliency [6] predict functional inhibition between similarly tuned neurons. What computations are consistent with the observed structure of the lateral connections between the excitatory and diverse types of inhibitory neurons? We combined natural scene statistics [7] and mouse V1 neuron responses [8] to compute the lateral connections and computations of individual neurons which would optimally integrate information from the classical receptive field with that from the surround. The direct implementation requires single neurons to make complex computations on their inputs. While it is possible for such computations to be implemented by the dendritic trees, we show that an approximation can be achieved with relatively simple neurons. We show that this network has “like-to-like” lateral connections between excitatory neurons similar to the observed one [2, 3], distance dependence of connections similar to the observed ones [9], and requires three classes of inhibitory neurons: one performing local normalization, one surround inhibition, and one gating the inhibition from the surround, similar to anatomical [4] and physiological studies. This method generates an entire connectivity matrix for lateral connections in a layer in a purely unsupervised fashion, such that it generates testable hypotheses for connectome studies. Additionally, when these lateral connections are implemented in a neuronal network the reconstruction of natural scenes is significantly improved. For images with different statistics, such as independent and identically distributed random patches, using a natural scene prior hurts reconstruction. However, an additional gating mechanism allows optimal reconstruction for this type of features as well. We hypothesize that this computation: optimal integration of contextual cues is a general property of cortical circuits, and the rules constructed for mouse V1 generalize to other areas and species.

**References**

1. Angelucci A. and Bressloff P. C. Contribution of feedforward, lateral and feedback connections to the classical receptive field center and extra-classical receptive field surround of primate V1 neurons. *In Progress in brain research* 2006, **154**:93–120.

2. Ko H., B Hofer S. B., Pichler B., Buchanan K. A., Sjöström P. J., and Mrsic-Flogel T. D. Functional specificity of local synaptic connections in neocortical networks. Nature, **473(7345)**:87–91, 5 2011.

3. Lee WC., Bonin V., Reed M., Graham B. J., Hood G., Glattfelder K., and Reid R.C. Anatomy and function of an excitatory network in the visual cortex. Nature, **532(7599):**370–4, 4 2016.

4. Jiang X., Shen S., Cadwell C. R., Berens P., Sinz F., Ecker A. S., Patel S., and Tolias A. S. Principles of connectivity among morphologically defined cell types in adult neocortex. Science, 350, 11 2015.

5. Olshausen B. A. and Field D. J. Emergence of simple-cell receptive field properties by learning a sparse code for natural images. Nature, **381(6583):**607–9, 6 1996.

6. Coen-Cagli R., Dayan P., and Schwartz O. Cortical Surround Interactions and Perceptual Salience via Natural Scene Statistics. PLoS computational biology, **8(3)**:e1002405, 3 2012.

7. Martin D, Fowlkes C, Tal D, and Malik J. A Database of Human Segmented Natural Images and its Application to Evaluating Segmentation Algorithms and Measuring Ecological Statistics. Proc. 8th Int’l Conf. Computer Vision **2** 416–423 2001

8. Durand S., Iyer R., Mizuseki K., De Vries S., Mihalas S., and Reid R.C. A comparison of visual response properties in the lateral geniculate nucleus and primary visual cortex of awake and anesthetized mice. Journal of Neuroscience, **36(48),** 2016.

9. Levy R. B. and Reyes A. D. Spatial Profile of Excitatory and Inhibitory Synaptic Connectivity in Mouse Primary Auditory Cortex. Journal of Neuroscience, **32(16)**, 2012.

## P251 Neural cross-frequency coupling functions in the resting state with eyes open and eyes closed

### Valentina Ticcinelli^1^, Tomislav Stankovski^1,2^, Peter V. E. McClintock^1^ and Aneta Stefanovska^1^

#### ^1^Department of Physics, Lancaster University, Lancaster LA1 4YB, United Kingdom; ^2^Faculty of Medicine, Ss Cyril and Methodius University, Skopje 1000, Macedonia

##### Correspondence: Valentina Ticcinelli (v.ticcinelli@lancaster.ac.uk)

*BMC Neuroscience* 2017, **18** (**Suppl 1**):P251

The electrophysiological activity of the brain emerges from interactions between large-scale neuronal ensembles [1], and is regulated by different types of cross-frequency coupling [2–5]. The latter are usually characterised by their coupling strength and directionality [2–4]. However, it is also possible to investigate the functional mechanisms of the interaction [5]. We introduce dynamical Bayesian inference for estimation of the coupling functions of neural oscillations in the presence of noise [6–8]. All of the possible phase-to-phase interactions between the oscillators of the network are inferred. Thus, the coupling can be decomposed into its partial functional contributions [8]. This allows one e.g. to isolate the estimated direct coupling between two nodes from the possible common coupling and self-coupling also involved in the interaction. As an illustrative example, the method is applied to characterization of the phase-to-phase neural coupling functions in electroencephalographic (EEG) data from the Neurophysiological Biomarker Toolbox (NBT) dataset [9]. Comparisons are made between the resting states with the eyes open (EO) and eyes closed (EC). We constructed the network by investigating the couplings between delta and alpha waves extracted from any pair of probes within the 10–20 measuring system; and we used phase-shuffled surrogates to test the significance of the inferred direct coupling strength. In doing so, we confirmed the earlier observation that the direct coupling is stronger in the EC state [10]. By investigating the form of the coupling functions, we were able to evaluate both inter-subject and intra-subject variability. We also evaluated the time variability of the form of the coupling. We showed that the coupling function is significantly less variable for the EC state. In a wider context, the method could in principle be applied to any pair of coupled oscillations in the same way as in the example shown here.

**Acknowledgements**

This work was supported by the Engineering and Physical Sciences Research Council (UK) [Grant No.EP/100999X1], by the EU projects BRACCIA [517133] and COSMOS [642563], and by the Action Medical Research (UK) project MASDA [GN1963]. VT is supported by a PhD grant from the Department of Physics, Lancaster University. Our grateful thanks are due to Klaus Lehnertz and Andreas Daffertshofer for valuable discussions. We also thank Lall Hussain for pointing out the toolbox and for his initial contribution to the work, and Lars Michels and the NBT research team for sharing their data and for most useful comments.

**References**

1. Breakspear, M., Heitmann, S., and Daffertshofer, A.: Generative models of cortical oscillations: neurobiological implications of the Kuramoto model, *Front. Human Neurosci*. 2010, **4**:190.

2. Friston, K. J., Harrison, L., and Penny, W.: Dynamic causal modelling, *Neuroimage* 2003, **19**: 1273–1302

3. Jensen, O. and Colgin, L. L.: Cross-frequency coupling between neuronal oscillations, *Trends Cognit. Sci*. 2007, **11**: 267–269.

4. Varela, F., Lachaux, J.-P., Rodriguez, E., and Martinerie, J. (). The brainweb: phase synchronization and large-scale integration, *Nat. Rev. Neurosci*. 2001, **2**: 229–239.

5. Kralemann, B., Cimponeriu, L., Rosenblum, M., Pikovsky, A., and Mrowka, R.: Phase dynamics of coupled oscillators reconstructed from data, *Phys. Rev. E* 2008, **77:** 066205.

6. Stankovski, T., Duggento, A., McClintock, P. V. E., and Stefanovska, A.: Inference of time-evolving coupled dynamical systems in the presence of noise*, Phys. Rev. Lett.* 2012, **109**: 024101.

7. Stankovski, T., Duggento, A., McClintock, P. V., and Stefanovska, A.: A tutorial on time-evolving dynamical Bayesian inference, *The European Physical Journal Special Topics* 2014, **223.13**: 2685–2703.

8. Stankovski, T., Ticcinelli, V., McClintock, P. V. E., and Stefanovska, A.: Coupling functions in networks of oscillators*, New J. Phys*. 2015, **17**: 035002.

9. Neurophysiological Biomarker Toolbox [https://www.nbtwiki.net/]

10. Deco, G., Jirsa, V. K., and McIntosh, A. R.: Emerging concepts for the dynamical organization of resting-state activity in the brain*, Nat. Rev. Neurosci.* 2010, **12**: 43–56.

## P252 Dissecting the total astrocytic potassium current in a computational model

### Predrag Janjić^1^, Dimitar Solev^3^, Gerald Seifert^2^, Ljupčo Kocarev^1^, Christian Steinhäuser^2^

#### ^1^Laboratory for Complex Systems and Networks, Macedonian Academy of Sciences and Arts, Skopje, Macedonia; ^2^Institute of Cellular Neurosciences, University of Bonn Medical School, Bonn, Germany; ^3^Unaffiliated, dimitar.solev@gmail.com

##### Correspondence: Predrag Janjić (predrag.a.janjic@gmail.com)

*BMC Neuroscience* 2017, **18** (**Suppl 1**):P252

Despite the growing experimental evidence about composition of the K^+^ conductance in mammalian astroglia [1], the origin of its typical linear whole cell I/V relation has not been addressed in detail with a computational model. We have used data of pharmacologically isolated Kir4.1, K2P and Kv4 K^+^ currents in freshly isolated astrocytes from mouse hippocampus [2] to describe mathematically equilibrium I/V characteristics and activation kinetics where applicable, for each current component separately. To account in more detail for the notable outward current in weakly rectifying Kir4.1 channels, we propose an extension of the Hagiwara model of dominant inwardly-rectifying Kir current, by adding a residual outward component. Allowing for a separate outward Kir current component, additive to the standard Boltzman equation we achieved a much better fit of the Ba^2+^sensitive current (Fig. 1). Assuming a short-pore structure of Kir4.1 channels we describe the outward voltage- and concentration-dependence of Kir4.1 permeability using the transition-state theory of the Eyring reaction-rate formalism, considering that the Mg^2+^block shapes the permeability of weak rectifier channels. Our extended model exposes several parameters whose ranges could be more precisely estimated by molecular dynamics simulation studies, or with single channel measurements in targeted point-mutation studies. In addition to describing the steady-state voltage dependence of K2P currents (represented in our case mostly by currents through TREK-1 and TREK-2 channels) its activation kinetics has been estimated, which smoothed out the contribution of this current component in differential models of K^+^ homeostasis or other dynamic phenomena. Inactivating 4-AP sensitive currents through Kv4 channels which are expressed at low density by astrocytes, have been modeled using standard Hodgkin-Huxley model. Added up, all three modeled current components successfully described the voltage-dependence of total experimental whole-cell K^+^ currents. We exemplified the usefulness of our model by simulating astrocytic currents in elevated K^+^ concentration in a single ECS pocket apposing the glial membrane. We believe that such a detailed model, which separately describes the individual current components, could be useful in describing the impact of channelopathies underlying altered astrocytic electrophysiology.
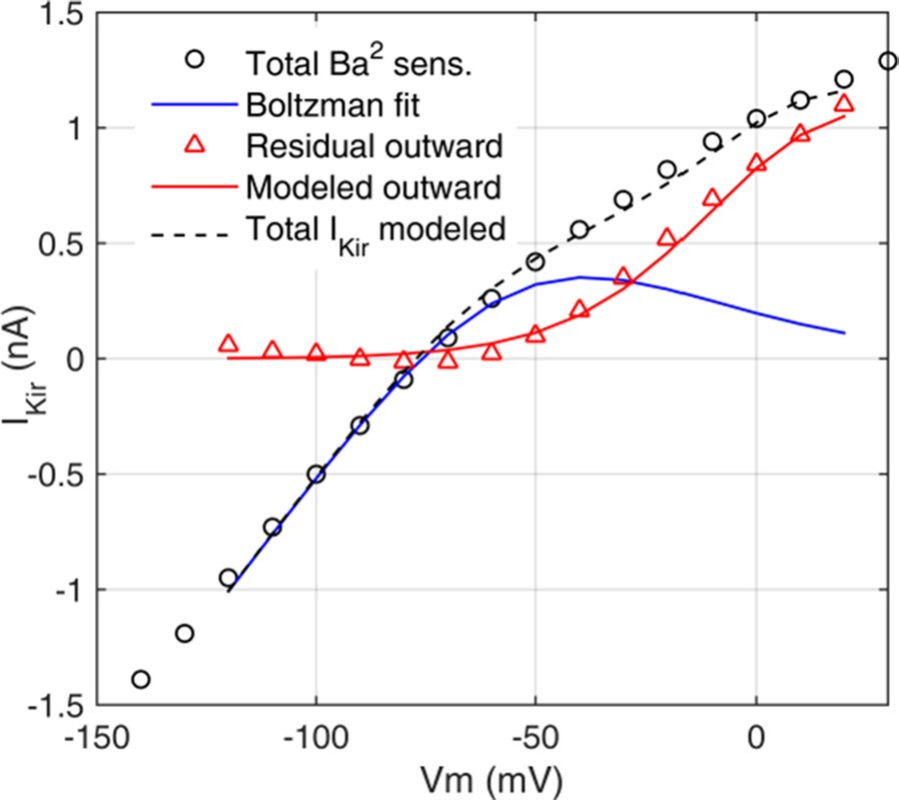


**Figure 1.** Total Ba^2+^sensitive current through Kir4.1 channels in an isolated astrocyte (circles) has been initially fitted by the Hagiwara model to describe the inwardly-rectifying part (blue line, extending to -120 mV). Residual outward Kir current (red triangles) obtained after subtracting inward current from the control, has been fitted with a 1-site, 2-barrier reaction rate model (red line). Added together (black line) they represent the total fit of Kir4.1 current. Both model components describe voltage- and concentration-dependence. External K^+^ concentration has been elevated to [K]_o_ = 5 mM in all measurements, with [K]_i_ = 130 mM. Numerical fit has been limited to -120 mV due to distortions from external Na^+^ block for very negative voltages, beyond the physiological voltage range.

**References**

1. Seifert G, Henneberger C, Steinhäuser C: Diversity of astrocyte potassium channels - An update, *Brain Res Rev* 2017, in-press, available online at: http://dx.doi.org/10.1016/j.brainresbull.2016.12.002

2. Seifert G, Hüttmann K, Binder DK, Hartmann C, Wyczynski A, Neusch C, Steinhäuser C: Analysis of Astroglial K^+^ Channel Expression in the Developing Hippocampus Reveals a Predominant Role of the Kir4.1 Subunit, *J. Neurosci* 2009; **29 (23):** 7474–7488.

## P253 Information rate of multiple synaptic release sites with separately released vesicles during short-term depression

### Mehrdad Salmasi^1,2,3^, Stefan Glasauer^1,2,3,4^, Martin Stemmler^2,5^

#### ^1^Graduate School of Systemic Neurosciences, Ludwig-Maximilian University, Munich, Germany; ^2^Bernstein Center for Computational Neuroscience, Munich, Germany; ^3^German Center for Vertigo and Balance Disorders, Ludwig-Maximilian University, Munich, Germany; ^4^Department of Neurology, Ludwig-Maximilian University, Munich, Germany; ^5^Department of Biology II, Ludwig-Maximilian University, Munich, Germany

##### Correspondence: Mehrdad Salmasi (mehrdad.salmasi@lrz.uni-muenchen.de)

*BMC Neuroscience* 2017, **18** (**Suppl 1**):P253

Chemical synapses are conduits for much of the brain’s information, but they are inherently unreliable, with unprovoked, spontaneous release of neurotransmitter alternating with intermittent unresponsiveness of the synapse to action potentials. A release of vesicle at a synapse depresses the probability of further releases in the short term. Given the stochastic nature of synapses, synaptic information efficacy has been used to quantify information transmission through synapses [1, 2]. Many theoretical approaches treat the synapse as a static, monolithic communication device. Yet many synapses have multiple release sites, each subject to separate short-term dynamics. These multiple sites compensate for the unreliability of individual release sites. Here we seek to quantify how the number of release sites affects the information efficacy of a synapse with short-term depression. In addition, we study the trade-off between the reliability of information transmission and energy consumption at the synapse.

To analyze the amount of information that a neuron can transfer through its release sites during short-term depression (Fig. 1A), we model each release site as a binary asymmetric channel whose state (release probability) is determined by its release history. A Markov chain of state transitions implements short-term depression followed by exponential recovery of the release site. It is assumed that the release sites are independent and the released vesicles are separable. We prove that the mutual information rate between the input spike process, *X*, and the release outcomes of the release sites, (*Y*_1_, *Y*_2_,…, *Y*_*K*_), is equal to the statistical average over the information rates of an equivalent communication channel for every possible state combination of the release sites. Using the derived expression, we show the compensatory effect of having multiple release sites in Fig. 1B. The dashed black line connects the capacity values of the neuron for different number of release sites. For a neuron with larger number of release sites, capacity is achieved at higher input spike rates. We then normalize the information rate by the energy consumed for the release and assess the compromise between the energy and the information rate of the neuron.
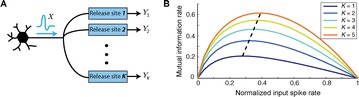


**Figure 1. A.** Information transmission through multiple release sites of a neuron. **B.** Mutual information rate of the neuron’s release site(s) as a function of normalized input spike rate, for different numbers of release sites. The normalized spike rate is the probability of having a spike in a time bin, and is equal to one when there is one spike per time bin

**Acknowledgements**

This work was supported by the BMBF grant 01EO1401 (German Center for Vertigo and Balance Disorders).

**References**

1. London M, Schreibman A, Häusser M, Larkum ME, Segev I: The information efficacy of a synapse. *Nature Neuroscience* 2002, **5(4)**, 332-340.

2. Fuhrmann G, Segev I, Markram H, Tsodyks M: Coding of temporal information by activity-dependent synapses. *Journal of Neurophysiology* 2002, **87(1)**, 140–148.

## P254 Properties of recurrent networks at maximum capacity for storing sequences of network states

### Danke Zhang, Chi Zhang, Armen Stepanyants

#### Department of Physics and Center for Interdisciplinary Research on Complex Systems, Northeastern University, Boston, MA 02115, USA

##### Correspondence: Armen Stepanyants (a.stepanyants@neu.edu)

*BMC Neuroscience* 2017, **18** (**Suppl 1**):P254

The ability of neural networks to associate successive states of network activity lies at the basis of various cognitive functions. In this study, we hypothesized that ubiquitous features of cortical network structure and dynamics develop as a result of continual memory storage. To test this hypothesis, we consider recurrent McCulloch and Pitts networks in which neurons may belong to different classes defined by their excitatory or inhibitory nature, firing probability, and robustness to noise. Learning in the network is mediated by changes in connection weights in the presence of constraints on the *l*_1_-norms of presynaptic weights of individual neurons.

To determine the memory storage capacity of the network we train the network on a set of random sequences of different lengths and subsequently test the retrieval of learned memories. The maximum (critical) capacity of the network is defined as the sequence length for which the success rate in memory retrieval equals 0.5. To retrieve a learned sequence, we initialize the network state at the beginning of the sequence and monitor memory playout. The sequence is considered to be retrieved successfully if the network states during the retrieval do not deviate substantially from the learned sequence. In practice, there is no need to precisely define the threshold amount of deviation. This is because for large networks, e.g. *N* > 100 neurons, the Hamming distance between the final states of the learned and retrieved sequences either remains within ~*N* ^0.5^, or diverges to ~*N*. Memory retrieval in the former case is said to be successful, while in the latter case the memory could not be retrieved.

We performed numerical simulations for networks of *N* = 1000 neurons and also solved the problem theoretically in the thermodynamic limit by using the replica theory [1–3]. The results show that critical networks have unique structural and dynamic properties which resemble those observed in many cortical systems from cerebellum to neocortex to hippocampus. First, we find that, consistent with the experimental data, probability of inhibitory connections in critical networks is greater than 0.5, whereas excitatory connectivity is sparse with connection probabilities less than 0.5. Second, we compare the distributions of connection weights in critical networks with the distributions of amplitudes of excitatory and inhibitory postsynaptic potentials. Due to the presence of very strong connections, the latter distributions typically have long, super-exponential tails. We show that in critical networks this feature can result from the heterogeneity of properties of individual neurons. Third, we find that with increasing robustness, critical networks exhibit a phase transition from networks with ordered dynamics quickly terminating in a frozen state, to networks with chaotic dynamics during which neurons exhibit irregular and correlated firing activity with average correlation coefficients in the 0.1–0.2 range. Finally, we show that the observed transition is accompanied with the emergence of neuron clusters, existence of which is suggested by recent experimental studies [4]. These results are consistent with the idea that cortical networks are operating in a critical state configured at the edge of order-to-chaos phase transition.

**Acknowledgements**

This work is supported by Air Force grant FA9550-15-1-0398 and NSF grant IIS-1526642

**References**

1. Brunel N, Hakim V, Isope P, Nadal JP, Barbour B: Optimal information storage and the distribution of synaptic weights: perceptron versus Purkinje cell. *Neuron* 2004, **43**(5):745-757.

2. Chapeton J, Gala R, Stepanyants A: Effects of homeostatic constraints on associative memory storage and synaptic connectivity of cortical circuits. *Frontiers in computational neuroscience* 2015, **9**:74.

3. Chapeton J, Fares T, LaSota D, Stepanyants A: Efficient associative memory storage in cortical circuits of inhibitory and excitatory neurons. *Proc Natl Acad Sci U S A* 2012, **109**(51):E3614–3622.

4. Perin R, Berger TK, Markram H: A synaptic organizing principle for cortical neuronal groups. *Proc Natl Acad Sci U S A* 2011, **108**(13):5419–5424.

## P255 Modulation of epileptic activity in thalamo-cortical networks by input from the cerebellar nuclei

### Julia Goncharenko^1^, Lieke Kros^2^, Neil Davey^1^, Christoph Metzner^1^, Chris de Zeeuw^2^, Freek Hoebeek^2^, Volker Steuber^1^

#### ^1^Centre for Computer Science and Informatics Research, University of Hertfordshire, Hatfield, AL10 9AB, UK; ^2^Department of Neuroscience, Erasmus MC, Wytemaweg 80, 3015 CN, Rotterdam, the Netherlands

##### Correspondence: Julia Goncharenko (i.goncharenko@herts.ac.uk)

*BMC Neuroscience* 2017, **18** (**Suppl 1**):P255

Epilepsy is one of the most prevalent neurological diseases in humans, affecting people of all ages. One of the most common forms of epilepsy in children is absence epilepsy [1]. Characteristic symptoms of absence epilepsy are sudden seizures that are accompanied by periods of behavioral arrest and impaired consciousness [1]. As in other forms of epilepsy, these seizures are electrophysiologically described by neuronal oscillations in thalamo-cortical networks and appear as generalized spike-and wave discharges (GSWDs) in the electroencephalogram (EEG) [2]. Oscillatory activity in cerebral cortex and thalamus can be caused by excessive inhibition in thalamus or by excessive cortical activity [2]. It has been suggested that the initiation of absence seizures can be triggered by events that switch neuronal activity in thalamo-cortical networks from normal asynchronous activity to synchronised oscillations [2].

Previous experimental studies have shown that oscillatory activity in thalamo-cortical networks and the accompanying GSWDs can be disrupted by stimulation of the thalamus [3]. Recently, it has been found that optogenetic activation of neurons in the cerebellar nuclei (CN) is a powerful tool to stop epileptic absence seizures using a closed-loop system in two unrelated mouse models [4]. Due to their anatomical bottleneck location, CN neurons can control the balance of excitation and inhibition in thalamus, resetting the oscillatory activity in thalamo-cortical loops. However, the mechanism underlying the disruption of thalamo-cortical oscillations and absence seizures by stimulation of the CN remains unknown.

Here we use computer simulations to investigate the mechanisms underlying the termination of absence seizures by optogenetic stimulation of CN neurons. We simulate a thalamo-cortical network model of adaptive exponential integrate-and-fire neurons, displaying complex intrinsic properties such as low-threshold spiking, regular spiking, fast spiking and adaptation [5]. The network activity can exhibit oscillatory or asynchronous irregular (AI) dynamics, depending on the level of adaptation in cortical cells [5]. We use electrophysiologically recorded spike trains that result from optogenetic activation of CN neurons in mouse models of absence epilepsy as input to the network model to analyse the mechanism of reverting abnormal oscillatory activity to the normal AI state. Our results illustrate how input from the CN can control oscillatory activity in thalamo-cortical networks and therefore provide a mechanism to terminate epileptic absence seizures.

**References**

1. Berg AT, Berkovic SF, Brodie MJ, et al.: Revised terminology and concepts for organization of seizures and epilepsies: report of the ILAE Commission on Classification and Terminology, 2005–2009. *Epilepsia* 2010, **51**:676–685.

2. Snead OC III: Basic mechanisms of generalized absence seizures. *Ann Neurol* 1995, **37(2)**:146–157.

3. Paz JT, Davidson TJ, Frechette ES, et al.: Closed-loop optogenetic control of thalamus as a tool for interrupting seizures after cortical injury. *Nat Neurosci* 2013, **16**:64–70.

4. Kros L, Eelkman Roda OHJ, Spanke JK, et al.: Cerebellar Output Controls Generalised Spike-and-Wave Discharge Occurrence. *Ann Neurol* 2015, **77(6)**:1027–1049.

5. Destexhe A.: Self-sustained asynchronous irregular states and up-down states in thalamic, cortical and thalamocortical networks of nonlinear integrate-and-fire neurons. *J Comput Neurosci* 2009, **27**:493–506.

## P256 The effect of homeostatic structural plasticity on associative memory in a network with spike-time dependent inhibitory synaptic plasticity

### Ankur Sinha, Christoph Metzner, Roderick Adams, Michael Schmuker, Neil Davey, Volker Steuber

#### UH Biocomputation Group, University of Hertfordshire, Hatfield, AL10 9AB, UK

##### Correspondence: Ankur Sinha (a.sinha2@herts.ac.uk)

*BMC Neuroscience* 2017, **18** (**Suppl 1**):P256

The stability of neuronal networks that are continuously modified by various activity dependent processes and their robustness to lesions or deafferentation necessitate the co-existence of complementary homeostatic mechanisms [1]. Recent research has studied these homeostatic plasticity mechanisms using both experiments and computational modelling.

In a computational study of homeostatic synaptic plasticity, Vogels and collaborators showed that inhibitory synaptic plasticity governed by a symmetric spike timing dependent plasticity rule successfully stabilises a spiking neuronal network to an asynchronous irregular (AI) state, as is observed in the cortex [2]. The proposed model also permitted the storage and recall of non-attractor Hebbian associative memories in the network.

Butz and van Ooyen recently presented a spiking neural network model of homeostatic structural plasticity [3]. In their study, neurons in the network attempt to maintain a fixed level of electrical activity by forming or breaking synaptic connections as required. The structural reorganisation of the network is also shown to replicate experimentally observed aspects of the restructuring of the visual cortex following deafferentation by focal retinal lesions [4, 5].

In the present study, we investigate the capacity of a cortical network model balanced by homeostatic inhibitory plasticity to store and recall non-attractor Hebbian associative memories. Extending our previous work [6], we investigate the functional effect of homeostatic structural plasticity on associative memory performance during network deafferentation and repair. We explore the interaction between the two homeostatic mechanisms, inhibitory spike-time dependent synaptic plasticity and structural plasticity, and investigate how the experimentally observed AI state is affected by the coexistence of these two homeostatic mechanisms that operate on different time scales. Furthermore, we discuss enhancements to the model of structural plasticity aimed at increasing biological plausibility and study their effect on memory capacity. Finally, we report on the variation in associative memory performance during network deafferentation and repair, and discuss the parameters that affect it.

**References**

1. Turrigiano GG: Homeostatic plasticity in neuronal networks: the more things change, the more they stay the same. *Trends in neurosciences* 1999, **22(5):** 221–227.

2. Vogels T, Sprekeler H, Zenke F, Clopath C, Gerstner W: Inhibitory plasticity balances excitation and inhibition in sensory pathways and memory networks. *Science* 2011, **334:**1569–1573.

3. Butz M, van Ooyen A: A simple rule for dendritic Spine and axonal bouton formation can account for cortical reorganization after focal retinal lesions. *PLoS Comput Biol*. 2013, **9(10):** e1003259

4. Keck T, Mrsic-Flogel TD, Afonso MV, Eysel UT, Bonhoeffer T, Hübener M: Massive restructuring of neuronal circuits during functional reorganization of adult visual cortex. *Nature Neuroscience* 2008, **11(10):** 1162–1167.

5. Yamahachi H, Marik SA, McManus JN, Denk W, Gilbert CD: Rapid axonal sprouting and pruning accompany functional reorganization in primary visual cortex. *Neuron* 2009, **64(5):** 719–729.

6. Sinha A, Davey N, Adams R, Steuber V: Structural plasticity and associative memory in balanced neural networks with spike-time dependent inhibitory plasticity. *BMC Neuroscience* 2015, **16(1):** P235.

## P257 The dependence of arithmetic operations on input location in cerebellar nucleus and cortical pyramidal neurons

### Maria Psarrou, Maria Schilstra, Neil Davey, Benjamin Torben-Nielsen, Michael Schmuker, Volker Steuber

#### Centre for Computer Science and Informatics Research, University of Hertfordshire, Hatfield, AL10 9AB, UK

##### Correspondence: Maria Psarrou (m.psarrou@herts.ac.uk)

*BMC Neuroscience* 2017, **18** (**Suppl 1**):P257

Neurons are constantly bombarded with numerous synaptic signals, which are integrated in order to generate output spikes. A simple way to depict neuronal computations is to plot the relationship between the neuronal input rate and the corresponding output spike rate, that is, the Input - Output relationship (I-O, or transfer function) [1]. A change in the slope or *gain* of the I-O curve in the presence of different cellular and synaptic mechanisms, such as synaptic noise, shunting inhibition or synaptic plasticity is an indicator of ongoing multiplicative operations [1–4]. Gain modulation is a brain-wide principle of neuronal computation, enabling nonlinear combinations of sensory and cognitive information. An essential component of gain modulation is that a modulatory input alters the sensitivity of the neuron to the original (driving) input, without changing its selectivity [5]. Different nonlinearities in the relationships between input firing rate, excitatory synaptic conductance and output firing rate have been shown to underlie gain modulation [2, 4]. In the present study, we investigate in two different types of neurons whether the dendritic location of excitatory input affects the arithmetic operation performed by different modulatory inhibitory inputs. We used two well described morphologically realistic conductance based models, a cerebellar nucleus (CN) neuron model [6] and a layer V pyramidal neuron model [7], and we explore various driving and modulatory input conditions. Modulatory input was provided either by distributed synaptic inhibitory input or a tonic somatic inhibitory conductance. When the driving and modulatory input were both of synaptic nature, we observed a correlation between the distance of the excitatory driving input from the soma and the extent of the multiplicative gain change in both the CN and the layer V pyramidal neurons. In the CN neuron, we found that excitatory inputs underwent additive operations when delivered in somatic and perisomatic areas, and multiplications when delivered to distal dendritic areas. In contrast, in the layer V pyramidal neuron excitatory driving input was always multiplied, independent of the synapse location. In all cases where inputs underwent multiplicative operations, the mapping between synaptic excitatory conductance and output firing rate revealed a nonlinearity, with more pronounced nonlinearities due to dendritic saturation in distal synaptic locations corresponding to larger multiplicative gain changes. To show that these non-linear mappings between input conductance and output rate were the basis of the multiplicative gain changes, we drove the two neuronal types with excitatory current injections, at the soma or different dendritic locations, in the presence of modulatory tonic somatic inhibition. In this case, the arithmetic operations performed in all distinct neuronal locations were additive shifts. Moreover, synaptic inhibition had a greater effect on neuronal output than somatic tonic inhibition. Our results indicate that the location and the nature of excitatory inputs affect in a systematic way whether the input undergoes a multiplicative or additive operation. The extent of these operations is also related to the nature of the inhibitory input. Furthermore, different neuronal types might perform different operations when the inputs are received in their perisomatic areas.

**References**

1. Silver RA. Neuronal arithmetic. *Nat Rev Neurosci.* 2010; **11:**474–89.

2. Prescott S a, De Koninck Y. Gain control of firing rate by shunting inhibition: roles of synaptic noise and dendritic saturation. *Proc. Natl. Acad. Sci. U. S. A*. 2003; **100:**2076–81.

3. Chance FS, Abbott LF, Reyes AD. Gain modulation from background synaptic input. *Neuron* 2002; **35:**773–82.

4. Rothman JS, Cathala L, Steuber V, Silver RA. Synaptic depression enables neuronal gain control. *Nature* 2009; **457**:1015–8.

5. Salinas E, Sejnowski TJ. Gain modulation in the central nervous system: where behavior, neurophysiology, and computation meet. *Neuroscientist* 2001; **7:**430–40.

6. Steuber V, Schultheiss NW, Silver RA, De Schutter E, Jaeger D. Determinants of synaptic integration and heterogeneity in rebound firing explored with data-driven models of deep cerebellar nucleus cells. *J. Comput. Neurosci.* 2011; **30:**633–58.

7. Hay E, Hill S, Schürmann F, Markram H, Segev I. Models of neocortical layer 5b pyramidal cells capturing a wide range of dendritic and perisomatic active properties. *PLoS Comput. Biol.* 2011; **7**

## P258 A Framework for Automated Validation and Comparison of Models of Neurophysiological and Neurocognitive Biomarkers of Psychiatric Disorders

### Christoph Metzner^1^, Achim Schweikard^2^, Tuomo Mäki-Marttunen^3^, Bartosz Zurowski^4^ and Volker Steuber^1^

#### ^1^Centre for Computer Science and Informatics Research, University of Hertfordshire, College Lane, Hatfield, AL10 9AB, United Kingdom; ^2^Institute for Robotics and Cognitive Systems, University of Luebeck, Luebeck, 23562, Germany; ^3^NORMENT, Institute of Clinical Medicine, University of Oslo, Oslo, Norway; ^4^Department of Psychiatry, University of Luebeck, Schleswig-Holstein, Luebeck, 23562, Germany

##### Correspondence: Christoph Metzner (c.metzner@herts.ac.uk)

*BMC Neuroscience* 2017, **18** (**Suppl 1**):P258

Research on psychiatric disorders has gradually shifted its focus from complex clinical phenotypes towards the identification of biomarkers and endophenotypic measures. Computational approaches have gained significantly more attention over the last years, and this has led to the emergence of ‘Computational Psychiatry’ as an independent discipline. Computational modelling of biomarkers promises to more readily shed light on the mechanisms underlying disorders and to facilitate the discovery of novel medications [1]. However, in order to develop a computational model, scientists need to have an in-depth understanding of the current, relevant experimental data, the current state of computational modeling and the state-of-the-art of statistical testing. Based on this knowledge, they have to choose the appropriate criteria with which the model predictions and experimental observations will be compared [2]. In a field where both the number of experimental and computational studies grows rapidly, as is the case for psychiatry, this becomes more and more impracticable. Omar et al. therefore proposed a framework for automated validation of scientific models, SciUnit [3]. Here, we propose to adopt this framework for the computational psychiatry community and to collaboratively build common repositories of experimental observations, computational models, test suites and tools. As a case in point, we have implemented test suites for auditory steady-state response deficits in schizophrenic patients, which are based on observations from several experimental studies [4–6], and we demonstrate how existing computational models [6, 7] can be validated against these observations and compared against each other. We have included sets of observations from three experimental studies, which concur on most findings but also disagree on some. This allows us to demonstrate the usefulness of our approach in highlighting and clarifying existing, potentially conflicting, experimental data. We have included computational models that not only comprise biophysically detailed as well as abstract models, but that also differ in implementation (native Python vs. Genesis vs NeuroML2), in order to demonstrate the flexibility of the approach. Furthermore, this additionally allows us to showcase the ability of the framework to compare models against each other based on a set of experimental observations. Furthermore, our approach enables us to assess the variability of the produced model output, and therefore the robustness of the findings, by generating a distribution of model instances where certain parameters, such as the precise timing of noise (however, not strength and type of noise) or the precise connectivity (however, not the distribution of connections) vary, which then are used to produce a distribution of model outputs. This can inform on the robustness of the findings and be compared against the variability of experimental observations.

**References**

1. Siekmeier, P.: Computational modeling of psychiatric illnesses via well-defined neurophysiological and neurocognitive biomarkers. *Neurosci Biobehav Rev* 2015, **57**: 365–380

2. Gerkin, R.C. and Omar, C.: NeuroUnit: Validation Tests for Neuroscience Models. *Front. Neuroinform. Conference Abstract: Neuroinformatics* 2013

3. Omar, C., Aldrich, J., and Gerkin, R.C.: Collaborative infrastructure for test-driven scientific model validation. *In CompanionProceedings of the 36th International Conference on Software Engineering, ACM,* 2014.

4. Kwon J.S., O’Donnell B.F., Wallenstein G.V., Greene R.W., Hirayasu Y., Nestor P.G., Hasselmo M.E., Potts G.F., Shenton M.E., and McCarley R.W..: Gamma frequency–range abnormalities to auditory stimulation in schizophrenia. *JAMA Psychiatry* 1999, **56(11)**:1001–1005

5. Krishnan, G.P., Hetrick, W.P., Brenner, C.A., Shekhar, A., Steffen, A.N., and O’Donnell, B.F.: Steady state and induced auditory gamma deficits in schizophrenia. *Neuroimage* 2009 **47(4)**:1711–1719

6. Vierling-Claassen, D., Siekmeier, P., Stufflebeam, S., and Kopell, N.: Modeling GABA alterations in schizophrenia: a link between impaired inhibition and altered gamma and beta range auditory entrainment.

*J Neurophysiol* 2008, **99(5)**:2656–2671

7. Metzner, C., Schweikard, A. and Zurowski, B.: Multi-factorial modeling of impairment of evoked gamma range oscillations in schizophrenia. *Front Comp Neurosci* 2016, **10**

## P259 Synergetic and redundant information flow in dynamical systems: an operative definition based on prediction

### Daniele Marinazzo^1^, Luca Faes^2^, Sebastiano Stramaglia^3^

#### ^1^Department of Data Analysis, Ghent University, Ghent, B9000, Belgium; ^2^BIOtech, Dept. of Industrial Engineering, University of Trento, and IRCS-PAT FBK, 38010 Trento, Italy; ^3^Dipartimento di Fisica, Università degli Studi Aldo Moro, Bari, and INFN, Sezione di Bari, 70123 Bari, Italy

##### Correspondence: Daniele Marinazzo (daniele.marinazzo@ugent.be)

*BMC Neuroscience* 2017, **18** (**Suppl 1**):P259

Information theoretic treatment of groups of correlated degrees of freedom can reveal their functional roles as memory structures or information processing units. Furthermore, by looking at the common amount of information shared in a group of variables we can tell whether they are mutually redundant or synergetic. The application of these insights to identify functional connectivity structure is a promising line of research. Another topic of general interest is the understanding of couplings between dynamical systems and their parts. Transfer entropy and Granger causality are popular approaches used to distinguish effectively driving and responding elements and to detect asymmetry in the interaction of subsystems. These two methods can be unified under some conditions, opening new computational and methodological perspectives. Several techniques can evidence sets of variables which provide information for the future state of the target. This information can be synergetic or redundant, with important implication on our understanding of the functioning of the dynamical system under analysis.

Importantly, not taking into account the joint dynamical influence of two or more variables can lead to bias and wrong estimations of links (false positive and false negatives).

In the field of information theory these concepts are often defined and studied by means of axioms. Here we will instead use an operative definition based on reduction in variance, using the unnormalized version of Granger causality. We will present an application to simulated datasets and neuroimaging data, such as the one depicted in Figure 1, where average redundant and synergetic contributions, computed on 116 brain regions from 90 subjects from the Human Connectome Project dataset are depicted.
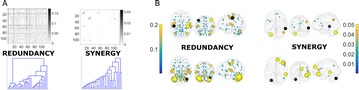


**Figure 1.** Synergetic and redundant influences between 116 brain regions from the AAL template, averaged over 90 subjects from the HCP dataset. A: matrix of synergetic/redundant contributions (top) and dendrograms (bottom). B: Redundant and synergetic contributions for two representative regions, a cortical one (top) and a cerebellar one (bottom)

**Reference**

1. Stramaglia S, Angelini L, Wu G, Cortes J, Faes L, Marinazzo D: Synergetic and redundant information flow detected by unnormalized Granger causality: application to resting state fMRI. *IEEE Trans. Biomed. Eng.* 2016, **63 (12):**2518–2524.

## P260 Forming and Using Hierarchical Cognitive Maps: a Neural Network Model

### Henry O. C. Jordan, Simon M. Stringer

#### OFTNAI, Dept. Experimental Psychology, University of Oxford, South Parks Road, OX1 3UD, Oxford, UK

##### Correspondence: Henry O. C. Jordan (hocjordan@live.co.uk)

*BMC Neuroscience* 2017, **18** (**Suppl 1**):P260

Clear evidence exists that model-based planning using cognitive maps occurs in mice and humans [1] and such planning has been modelled by researchers such as Gaussier et al.[2] and Ponulak & Hopfield [3] using a gradient or propagating-wave approach. It is also well-known that planning can be hierarchical [4]; such hierarchical planning has been explored by computer scientists [5, 6] but almost exclusively in model-free reinforcement learning tasks. We combine these strands of research to create an *unsupervised neural network model of hierarchical planning* using cognitive maps [1, 3] and policy-based option discovery [6]. Our model:

- Receives sensory information from a simulated agent as it explores a grid environment.
- Stores the structure of that environment within recurrent neural connections as a cognitive map.
- Uses this internal map to reach goal states via a propagating wave front mechanism.
- Solves a set of planning tasks and identifies common elements (‘options’) within those solutions.
- Uses these common elements (‘options’) to speed up the planning process for further tasks.

Our model plans by causing a wave of neural activation to propagate from the goal state to the agent’s current state. Learned options act as shortcuts for this wave, allowing the agent to speed up navigation through well-travelled areas (Fig. 1). We also demonstrate that options provide more benefit in larger, more structured environments.

**Conclusion:** Previous work [5, 6] has shown that including options increases an agent’s ability to *accumulate reward.* In contrast, we show that learning and using options can instead increase the *processing*-*per*-*step efficiency of action selection* when making decisions in a learned environment. Our model also demonstrates that such hierarchical learning and planning can be performed by an unsupervised neural network and therefore hints at a biological implementation.
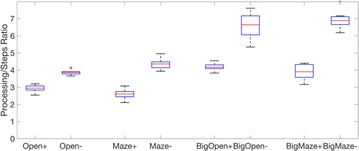


**Figure 1.** Planning per Action, with (+) and without (-) learned options. This box plot shows how many time steps an agent requires to select its next action, averaged over 100 trials in each of four different environments: an open environment without obstacles, a structured maze with four rooms, a big open environment with 4x as many states, and a big maze

**Acknowledgements**

We thank the Oxford Foundation for Theoretical Neuroscience and AI (OFTNAI) for supporting this work.

**References**

1. Dolan RJ, Dayan P. Goals and habits in the brain. Neuron. 2013. p. 312–25.

2. Gaussier P, Revel A, Banquet JP, Babeau V. From view cells and place cells to cognitive map learning: Processing stages of the hippocampal system. Biol. Cybern. 2002;86:15–28.

3. Ponulak F, Hopfield JJ. Rapid, parallel path planning by propagating wavefronts of spiking neural activity. Front. Comput. Neurosci. 2013;7:98.

4. Botvinick MM, Niv Y, Barto AC. Hierarchically organized behavior and its neural foundations: a reinforcement learning perspective. Cognition. 2009;113:262–80.

5. Sutton RS, Precup D, Singh S. Between MDPs and semi-MDPs: A framework for temporal abstraction in reinforcement learning. Artif. Intell. 1999;112:181–211.

6. Girgin S, Polat F, Alhajj R. Improving reinforcement learning by using sequence trees. Mach. Learn. 2010;81:283–331.

## P261 Harmonic SSEP Spectra are Determined by Modulation of Population Firing Rate - a Modeling Study

### Elżbieta Gajewska-Dendek, Piotr Suffczyński

#### Department of Biomedical Physics, Institute of Experimental Physics, University of Warsaw, Warsaw, 02-093 Poland

##### Correspondence: Elżbieta Gajewska-Dendek (egd@fuw.edu.pl)

*BMC Neuroscience* 2017, **18** (**Suppl 1**):P261

Steady State Evoked Potentials (SSEP) are EEG signal responses to periodically changing stimulus. SSEPs consist of a strong fundamental response and sometimes also its harmonic and subharmonic frequencies. The SSEP can be observed in visual, auditory and somatosensory modalities. Despite multiple applications of SSEP in cognitive neuroscience, clinical neuroscience and brain computer interfaces (BCI), some basic questions concerning this phenomenon still remain open: what is the physiological mechanism of generation of harmonic spectra and what determines relative spectral power of SSEP at fundamental frequency and at the harmonics.

The aim of this study was to investigate the SSEP generation mechanisms and its characteristic with a realistic computational model. The presented results are an extension from previously published version [1]. The model consists of single compartment excitatory and inhibitory cells of the Hodgkin-Huxley type, arranged in multiple cortical columns. The network contains 8000 neurons and more than 10^6^ synapses, based on connectivity data from cat primary visual cortex. The sensory stimulus is modeled as 7 to 50 Hz square or sine modulated rate of Poisson process. The simulated EEG signal is as a sum of synaptic currents of all pyramidal neurons. Additionally, for the square stimulus, we varied duty cycle: 50% (default), 33% and 66% in order to investigate whether SSEP spectral power depends on magnitude of ON and OFF responses or on the overall energy of the stimulus. The magnitude of transient responses was determined by firing adaptation strength of excitatory cells by modulating the conductivity of Ca-dependent potassium current. We compare the simulation data with experimental EEG recordings obtained in somatosensory cortex during vibrotactile stimulation as well as from visual cortex in response to flickering stimuli. The spectra of modeled SSEP exhibit fundamental and higher harmonic frequencies, similarly to experimental observations. The neurons firing rates are approximately constant and much lower than stimulus frequencies. The network oscillation emerges from irregular and sparse firing of individual neurons but in phase with the population fundamental rhythm. The harmonic frequencies cannot be directly related to firing of individual neurons but rather to EEG waveform resulting from overall network activity. Additionally, our modeling study shows that the SSEP power is dependent on both the stimulus duty cycle and degree of adaptation: in general, the largest spectral power of dominant frequency was observed for duty cycle 50%, and medium adaptation strength. The signal energy increased for lower duty cycle (<66%) and low adaptation, or for higher duty cycle (>33%) and stronger neuronal adaptation.

**Reference**

1. Gajewska-Dendek E, Suffczyński P: Investigation of SSEP by means of a realistic computational model of the sensory cortex. In: Villa AEP, Masulli P, Pons RAJ. Artificial Neural Networks and Machine Learning - ICANN 2016: 25th International Conference on Artificial Neural Networks, Barcelona, Spain, September 6-9, 2016, Proceedings, Part I. Springer International Publishing; 2016. p. 532.

## P262 Computational measure to account for erroneous neural deactivation when oxygen supply cannot meet metabolic demand in neuroimaging studies

### Nicoladie Tam^1^, George Zouridakis^2^, Luca Pollonini^2^

#### ^1^Department of Biological Sciences, University of North Texas, Denton, TX 76203, USA; ^2^Departments of Engineering Technology, Computer Science, and Electrical and Computer Engineering, University of Houston, Houston, TX, 77204, USA

##### Correspondence: Nicoladie Tam (nicoladie.tam@unt.edu)

*BMC Neuroscience* 2017, **18** (**Suppl 1**):P262

**Introduction:** Functional near-infrared spectroscopy (fNIRS) is an emerging optical imaging technique that can detect the neural activation/deactivation based on the optical absorption characteristics of both oxy-hemoglobin (oxy-Hb) and deoxy-hemoglobin (deoxy-Hb). The supply of oxygen to neural tissues can be affected not only by vasoconstriction or vasodilation, but also by the limited capacity of the system to supply more oxygen when demand exceeds the maximum oxygen availability. When this rate-limiting condition occurs, a decrease in oxygen availability is detected that can be erroneously interpreted as deactivation. Thus, absolute changes in oxy-/deoxy-Hb concentrations do not always represent neural activation/deactivation. It is therefore necessary to develop alternative measures to account for such a paradox, so that a decrease in Hb concentration is not misinterpreted as deactivation.

**Methods:** Rather than using absolute oxy-Hb and deoxy-Hb concentration as the metric, we propose to normalize these measures by the total blood volume (oxy-Hb + deoxy-Hb), so that they become oxy-Hb/(oxy-Hb + deoxy-Hb) and deoxy-Hb/(oxy-Hb + deoxy-Hb), respectively. We observed in previous studies [1–4] that the oxygen demand could exceed the oxygen supply in the cortex for such a motor task. To test the proposed measures of hemodynamic responses, 75 human subjects were recruited to perform arm movements in two orthogonal directions (front-back and left-right) while we recorded the hemodynamic responses from the motor and prefrontal cortices.

**Results:** Using the proposed measures, we were able to detect the relative changes in oxy- and deoxy-Hb concentrations in relation to the blood supply (i.e., the total blood volume). In most circumstances, when deoxy-Hb (oxygen extraction) concentration increased, oxy-Hb (oxygen delivery) concentration decreased simultaneously. In certain phases of movement execution, oxygen extraction (deoxy-Hb) appeared to remain constant after it had increased, while oxygen delivery (oxy-Hb) continued to decrease. When oxygen availability was restricted, the ability to extract more oxygen was also limited, resulting in an apparent maxed-out response. The analysis showed that the paradoxical hemodynamic changes in deoxy-Hb could be compensated by the normalized measures. This metric could indicate an *increase* in normalized deoxy-Hb response (oxygen demand), in spite of a detected *decrease* in both deoxy-Hb (oxygen extraction) and oxy-Hb (oxygen delivery) concentrations.

**Conclusions:** The proposed normalized oxy- and deoxy-Hb measures can correctly detect relative changes in oxygen demand with respect to the available oxygen (oxy-Hb + deoxy-Hb), even when oxygen supply cannot meet demand. We have established an alternative measure to account for the erroneous interpretation of neural deactivation.

**References**

1. Tam ND, Pollonini L, Zouridakis G: Decoding movement direction using phase-space analysis of hemodynamic responses to arm movements based on functional near-infrared spectroscopy. In: *38th Annual International Conference of the IEEE Engineering in Medicine & Biology Society: August 16*-*20, 2016 2016; Orlando, FL*: IEEE; 2016: 1580–1583.

2. Tam ND, Pollonini L, Zouridakis G: Phase space analysis of hemodynamic responses to intentional movement directions using functional near-infrared spectroscopy (fNIRS) optical imaging technique. In: *25th Annual Computational Neuroscience Meeting: CNS*-*2016: July 2*–*7, 2016 2016; Jeju, South Korea*; 2016: 54.

3. Tam ND, Zouridakis G: Temporal decoupling of oxy- and deoxy-hemoglobin hemodynamic responses detected by functional near-infrared spectroscopy (fNIRS). *Journal of Biomedical Engineering and Medical Imaging* 2014, **1**(2):18–28.

4. Tam ND, Zouridakis G: Decoding movement direction from motor cortex recordings using near-infrared spectroscopy. In: *Infrared Spectroscopy: Theory, Developments and Applications.* edn. Hauppauge, NY: Nova Science Publishers, Inc.; 2014.

## P263 Detecting brain hubs following brief mindfulness training

### Yi-Yuan Tang^1^, Rongxiang Tang^2^

#### ^1^Department of Psychological Sciences, Texas Tech University, Lubbock, TX 79409, USA; ^2^Department of Psychological & Brain Sciences, Washington University in St. Louis, St. Louis, MO 63130, USA

##### Correspondence: Yi-Yuan Tang (yiyuan.tang@ttu.edu)

*BMC Neuroscience* 2017, **18** (**Suppl 1**):P263

Many studies have shown that mindfulness training improves attention control, emotion regulation and cognitive performance through changing brain activity and the efficiency of brain networks [1–4]. Graph theory analysis could reveal the role of specific functional areas that are particularly important for integrating information across the whole-brain networks, called hubs [5, 6]. However, what hubs involve in mindfulness training and support positive changes are not well understood. Here, we applied a novel graph theory analysis to resting-state fMRI data to identify brain hubs induced by a brief mindfulness training - integrative body–mind training (IBMT), which was previously reported in our series of randomized studies [1–4].

Forty-two (21 ± 1.6 years old) healthy college students were recruited and randomly assigned to an IBMT group or a relaxation group (RT). The participants had no previous training experience and received 4 weeks of IBMT or RT training with 30 min per session for 20 sessions (~10 h training in total). All subjects gave written informed consent in accordance with the Declaration of Helsinki. The protocol was approved by the local Institutional Review committee. Neuroimaging data was collected using a 3-Telsa Siemens Allegra scanner and pre-processed following the standard procedures included slice timing, motion correction, regression of WM/CSF signals and spatial normalization [4]. For network parcellation and construction, we used a well-validated parcellation scheme consisting of 333 cortical parcels that are distributed across the brain and assigned to 13 different functional networks [5]. We applied network-based approach towards neuroimaging data and two network measures - global efficiency and participation coefficient were computed based on literature [6]. Compared to RT, after 10 h training, IBMT induced significant reduction of global efficiency in the midline default mode network (DMN) and increased participation coefficient at ventral anterior cingulate cortex (vACC).

**Conclusions:** This study utilized a novel graph theory analysis of functional networks to assess the brain efficiency and participation of hubs following brief mental training. Consistent with our and other research, our results suggest that brief mindfulness training IBMT significantly reorganizes DMN activity and network efficiency that may reallocate more resources for better self-control through the key hub in the vACC.

**Acknowledgements**

This work was supported by the Office of Naval Research.

**References**

1. Tang YY, Holzel BK, Posner MI: The neuroscience of mindfulness meditation. *Nat Rev Neurosci.* 2015, **16**: 213–225.

2. Tang YY, et al.: Short-term meditation training improves attention and self-regulation. *Proceedings of the National Academy of Sciences, USA.* 2007, **104:**17152–17156.

3. Tang YY, et al.: Central and autonomic nervous system interaction is altered by short term meditation. *Proceedings of the National Academy of Sciences, USA.* 2009, **106:** 8865–70

4. Tang YY, Tang R, Posner MI: Brief meditation training induces smoking reduction. *Proceedings of the National Academy of Sciences, USA.* 2013, **110:** 13971–13975.

5. Gordon EM, et al.: Generation and evaluation of a cortical area parcellation from resting-state correlations. *Cerebral Cortex* 2016, **26:** 288–303.

6. Rubinov M, Sporns O: Complex network measures of brain connectivity: uses and interpretations. *Neuroimage* 2010, **52:** 1059–1069.

## P264 Interplay between propagation delay and frequency of oscillation determines emergent structures of neuronal networks driven by triplet-based STDP

### Mojtaba Madadi Asl^1^, Alireza Valizadeh^1,2^, Peter A. Tass^3^

#### ^1^Department of Physics, Institute for Advanced Studies in Basic Sciences (IASBS), Zanjan, 45195-1159, Iran; ^2^School of Cognitive Sciences, Institute for Research in Fundamental Sciences (IPM), Tehran, 19395-5746, Iran; ^3^Department of Neurosurgery, School of Medicine, Stanford University, Stanford, CA, 94305, USA

##### Correspondence: Mojtaba Madadi Asl (m.madadi@iasbs.ac.ir)

*BMC Neuroscience* 2017, **18** (**Suppl 1**):P264

Spike-timing-dependent plasticity (STDP) adjusts synaptic strengths according to the relative timing of pre- and postsynaptic spikes [1]. The classic STDP rule eliminates bidirectional connections between two coupled neurons and turns them into unidirectional connections [2, 3]. As shown recently, by taking into account dendritic and axonal propagation delays, the conventional pair-based additive STDP may lead to both unidirectional and bidirectional connections, or decouple neurons by weakening reciprocal connections in both directions [4]. The triplet-based STDP, however, employs triplets of spikes that modify synaptic strengths [5]. Hence, the latter captures the effect of frequency of oscillations on the pre-post pairing. Here, we provide a general theoretical framework by assuming that the neurons are phase-locked with a phase lag which is determined by the temporary values of the synaptic strengths, propagation delays, frequency of oscillation, and the phase sensitivity of the neurons, and explore how the final configuration of the system can be predicted. In the absence of propagation delays, low-frequency oscillation leads to unidirectional connection for both pair- and triplet-based STDP. However, for higher frequencies, the triplet-based model has a tendency to achieve bidirectional connections, but results for the pair-based are the same as in the low-frequency regime. We show that employing triplet-based STDP leads to diverse connectivity patterns of oscillatory neurons in the presence of propagation delays, which qualitatively differ from the results obtained by pair-based STDP. In particular, large axonal propagation delay in the high-frequency regime is associated with a stable decoupling of both reciprocal synapses when the neurons are in a phase-locked state (see Figure 1F).
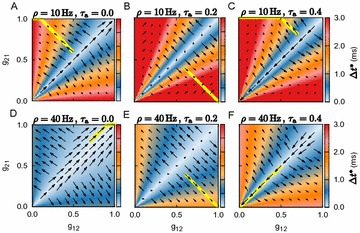


**Figure 1.** Theoretical prediction of triplet-based synaptic plasticity modification. **A-C.** Low-frequency regime. The colors show the phase lag of spiking of the neurons derived from the joint phase model and the vector field shows the direction of change in the joint synaptic strengths. The yellow curves denote the simulated synaptic strengths for a random initial value. **D-F.** High-frequency regime. Total time of simulations is 10 s. The dendritic delay is fixed at 0.2

**References**

1. Bi GQ, Poo MM: Synaptic modifications in cultured hippocampal neurons: dependence on spike timing, synaptic strength, and postsynaptic cell type. *J Neurosci* 1998, **18(24):** 10464–10472.

2. Song S, Miller KD, Abbott LF: Competitive Hebbian learning through spike-timing-dependent synaptic plasticity. *Nat Neurosci* 2000, **3(9):** 919–926.

3. Bayati M, Valizadeh A: Effect of synaptic plasticity on the structure and dynamics of disordered networks of coupled neurons. *Phys Rev E 2012,* **86(1):** 011925.

4. Madadi Asl M, Valizadeh A, Tass PA: Dendritic and Axonal Propagation Delays Determine Emergent Structures of Neuronal Networks with Plastic Synapses. *Sci Rep* 2017, **7(39682)**. doi: 10.1038/srep39682.

5. Pfister JP, Gerstner W: Triplets of spikes in a model of spike timing-dependent plasticity. *J Neurosci* 2006, **26(38):** 9673–9682.

## P265 Plasticity and network implications of a synaptic LPA-signalling pathway

### Andreas Nold^1†^, Wei Fan^2^, Sara Konrad^1^, Heiko Endle^2^, Johannes Vogt^2†^, Tatjana Tchumatchenko^1†^

#### ^1^Theory of Neural Dynamics, Max Planck Institute for Brain Research, 60438 Frankfurt, Germany; ^2^Institute for Microscopic Anatomy and Neurobiology, University Medical Center, Johannes Gutenberg University, 55131 Mainz, Germany

##### Correspondence: Andreas Nold (andreas.nold@brain.mpg.de)

^†^equal contribution

*BMC Neuroscience* 2017, **18** (**Suppl 1**):P265

We explore the implications of a synaptic signalling pathway involving the plasticity-related gene 1 (PRG-1), a molecule which is located at the postsynaptic membrane and modulates glutamatergic transmission, for the stability of the steady states of cortical circuit models. An accurate synaptic function is of crucial importance for learning and memory formation. Recently, the importance of this postsynaptic control element has been shown for synaptic signalling: Deletion of PRG-1 in mice leads to neuronal hyper excitability [1], and a single nucleotide polymorphism (SNP) in the PRG-1 gene affecting approx. 5 million European and US citizens is linked to psychiatric disorders such as schizophrenia [2].

PRG-1 modulates glutamatergic transmission via its ability to take up lysophosphatidic acid (LPA), which is synthetized by autotaxin, from the synaptic cleft [1, 2]. Inhibition of LPA-uptake, as present in PRG-1 deficient mice, leads to elevated synaptic LPA levels which via LPA_2_-receptors lead to higher levels of presynaptic intracellular Ca^2+^, higher vesicle release probabilities, and ultimately to neuronal hyperexcitability. This pathway was recently suggested to modulate synaptic short-term plasticity properties in the hippocampus [3]. Presynaptically, the pathway is modulated by glutamate which stimulates autotaxin (ATX) activity and thereby LPA-synthesis.

Here, we present a synaptic model implementing this LPA-based signalling pathway as an extension of the classical Tsodyks-Markram model for short-term synaptic plasticity [4], and explore the implications at a single synapse level, depending on increasing pre- and postsynaptic firing rates. The former leads to higher levels of LPA production via ATX, therefore increasing the LPA-concentration in the synaptic cleft, and elevating the presynaptic Calcium concentration, which leads to higher vesicle release probabilities. The latter modulates the activity of LPA-uptake via PRG-1. Implications are explored for short-term facilitation and depression of synapses, both for wild-type, as well as PRG-1 deficient cases.

We propose an efficient network implementation and analyze how loss of PRG-1 function affects the steady states of cortical circuits models, characterized by large, sparsely connected spiking networks in an asynchronous balanced excitation-inhibition regime. Results for bistable states of the network [5] are presented.

**Acknowledgements**

We acknowledge financial support from DFG through the CRC1080 and from Max Planck Society.

**References**

1. Trimbuch T, Beed P, Vogt J, Schuchmann S, Maier N, Kintscher M, Breustedt J, Schuelke M, Streu N, Kieselmann O, Brunk I. Synaptic PRG-1 modulates excitatory transmission via lipid phosphate-mediated signaling. *Cell* 2009, **138(6)**:1222–35.

2. Vogt J, Yang JW, Mobascher A, Cheng J, Li Y, Liu X, Baumgart J, Thalman C, Kirischuk S, Unichenko P, Horta G. Molecular cause and functional impact of altered synaptic lipid signaling due to a PRG‐1 gene SNP. *EMBO Mol Med* 2016, **8(1)**:25–38.

3. Vogt J, Kirischuk S, Unichenko P, Schlüter L, Pelosi A, Endle H, Yang JW, Schmarowski N, Cheng J, Thalman C, Strauss U. Synaptic Phospholipid Signaling Modulates Axon Outgrowth via Glutamate-dependent Ca2 + -mediated Molecular Pathways. *Cerebral Cortex* 2017, **27**:131–145.

4. Tsodyks M, Pawelzik K, Markram H. Neural networks with dynamic synapses *Neural Comput* 1998, **10(4)**:821–35.

5. Mongillo G, Hansel D, van Vreeswijk C. Bistability and spatiotemporal irregularity in neuronal networks with nonlinear synaptic transmission. *Phys Rev Lett* 2012, **108(15)**:158101.

## P266 Postsynaptic Activity-Dependent Synaptic Scaling Enables the Functional Organization of Memories

### Juliane Herpich^1,2^, Christian Tetzlaff^1,2^

#### ^1^Third Institute of Physics - Biophysics, Department of Computational Neuroscience, University of Goettingen, Goettingen, Germany; ^2^Bernstein Center for Computational Neuroscience, University of Goettingen, Goettingen, Germany

##### Correspondence: Juliane Herpich (juliane.herpich@phys.uni-goettingen.de)

*BMC Neuroscience* 2017, **18** (**Suppl 1**):P266

As known from everyday life, humans are permanently exposed to a variety of sensory inputs from their environment. Thereby, the ongoing challenge, humans have to deal with, is to continuously and adaptively respond to these sensory stimulations. On the neuronal level, modification of synapses (interface between two neurons) is a weighty mechanism for adapting the response properties of neurons according to their external stimulation. Hereby, synaptic plasticity is the main mechanism underlying learning [1–4] and, in combination with a homeostatic mechanism, yields the formation of strongly interconnected subgroups of neurons [5–7], so-called Hebbian cell assemblies (CAs) [1]. Such a CA represents the learned memory trace of the corresponding environmental stimulus [1]. Moreover, dependent on the details of the stimuli, humans exhibit the remarkable ability to organize memories (i.e. CAs), thus, to connect, generalize, and discriminate them, which supports the integration of novel stimuli and enables complex behavior [8, 9]. How these memory organizations are realized on a neuronal level based on the idea of cell assemblies is still unknown.

Here, we analyze in a theoretical neuronal network model whether the interaction of synaptic plasticity with different formulations of synaptic scaling [10] fulfills basic requirements of single synapse dynamics, such as sensitivity to stimulations and stability in their weight dynamics. Our analyses show that synaptic plasticity in combination with synaptic scaling dependent on the local (synapse specific) synaptic weight and the global (dendritic-branch specific) postsynaptic activity suffices the aforementioned characteristics of synapse dynamics. For simplicity, we abstract the neuronal dynamics of the network to its respective dynamics in a mean-field model of two interconnected, homogeneous populations of neurons. These populations serve as memory representations on the neuronal level (i.e. CAs; strong synaptic weights). Given different stimulation protocols, these two CAs can be dynamically connected with each other. Our analyses show that, dependent on the stimulation protocol, the CAs can be associated, discriminated, or can form a sequence. Remarkably, this rich repertoire of memory interactions is only present if the CAs themselves are in a matured state, in other words, if the timescale of the synaptic dynamics within the cell assemblies are slower than between them. This indicates that the interaction between memory items strongly depends on the internal state of the cell assemblies involved.

In summary, this work reveals a neuronal network model that is capable to exhibit with a local (Hebbian synaptic plasticity and synaptic weight-dependent scaling) and global (postsynaptic activity-dependent scaling) acting learning rule different functional organizations of memories observed in human behavior.

**References**

1. Hebb D.O.: The Organization of Behaviour. Wiley, New York, 1949.

2. Anderson R.C. and Kulhavy R.W.: Learning Concepts from Definitions 1. *American Educational Research Journal,* **9.3:**385–390, 1972.

3. Martin S.J., Grimwood P.D., and Morris R.G.M.: Synaptic plasticity and memory: An evaluation of the hypothesis. *Annual Review Neuroscience*, **23:**649–711, 2000

4. Eichenbaum H.: The cognitive neuroscience of memory: An introduction. *Oxford University Press*, 2011.

5. Abbott L.F. and Nelson S.B.: Synaptic plasticity: taming the beast. *Nature neuroscience,* **3:**1178–1183, 2000.

6. Tetzlaff C., Kolodziejski C., Timme M., and Wörgötter F.: Synaptic scaling in combination with many generic plasticity mechanisms stabilizes circuit connectivity. *Frontiers in Computational Neuroscience*, **5:**47, 2011.

7. Tetzlaff C., Kolodziejski C., Timme M., Tsodyks M., and Wörgötter F.: Synaptic scaling enables dynamically distinct short- and long-term memory formation. *PLoS Computational Biology*, **9(10):**e1003307, 2013.

8. Gagné R.M: The conditions of learning. 1970.

9. Tulving E., and Donaldson W.: Organization of memory. 1972.

10. Turrigiano G.G., Leslie K.R., Desai N.S., Rutherford, L.C., and Nelson, S.B.: Activity-dependent scaling of quantal amplitude in neocortical neurons. *Nature*, ***391***(6670):892–896, 1998.

## P267 Input-dependent Synaptic Consolidation of Memory Representations

### Jannik Luboeinski^1^, Christian Tetzlaff^2^

#### ^1^Third Institute of Physics - Biophysics, Department of Computational Neuroscience, University of Göttingen, Göttingen, Germany; ^2^Bernstein Center for Computational Neuroscience, University of Göttingen, Göttingen, Germany

##### Correspondence: Jannik Luboeinski (jannik.luboeinski@uni-goettingen.de)

*BMC Neuroscience* 2017, **18** (**Suppl 1**):P267

Long-term synaptic plasticity serves as the main process underlying learning and forming sustained memory representations in neuronal networks. In conjunction with homeostatic plasticity, long-term synaptic plasticity leads to the formation of strongly interconnected subgroups of neurons [1, 2]. These subgroups are referred to as memory representations or Hebbian cell assemblies [3]. Experiments have identified two different types of long-term synaptic plasticity [4, 5, 6, 7]: On the one hand, early-phase plasticity acts on a time scale of a few hours, while, on the other hand, late-phase plasticity operates on a time scale of more than eight hours up to several days. The transition of a synapse from the early-phase to the late-phase state is called synaptic consolidation and requires protein synthesis in the soma of the postsynaptic neuron. These proteins are transported along the dendrite and lead to a state-switch of those synapses that have formed a tag beforehand (synaptic tagging and capture hypothesis [2]). Protein synthesis as well as the formation of tags depend on the ongoing neuronal activity and external stimuli. However, the impact of synaptic consolidation on the formation and maintenance of cell assemblies, thus on their stability, is still widely unknown.

Here, we investigate in a theoretical network model under which input conditions synaptic consolidation yields the stabilization of cell assemblies, transferring the corresponding synapses from the early-phase to the late-phase state. For this examination, we developed and analyzed a spiking network model based on a well-known single-synapse model of the processes of synaptic consolidation [8, 9, 10]. Interestingly, our results show that the dynamics of synaptic consolidation are not homogeneous within a cell assembly: namely, the system shows a discrimination between the induction of late-phase plasticity and early-phase plasticity. In the ‘core’ of the cell assembly, synapses are in the long-living late-phase state, whereas in the surroundings of the core (‘halo’), synaptic changes do not overcome the early phase. Further analysis indicates that this discrimination could imply functionally important principles. In summary, our work provides a further step in understanding the step-by-step consolidation of memory representations in biologically realistic neuronal networks.

**References**

1. Tetzlaff C, Kolodziejski C, Timme M, Wörgötter F: Synaptic scaling in combination with many generic plasticity mechanisms stabilizes circuit connectivity. *Frontiers in Computational Neuroscience* 2011, **5:**47.

2. Tetzlaff C, Kolodziejski C, Timme M, Tsodyks M, Wörgötter F: Synaptic scaling enables dynamically distinct short- and long-term memory formation. *PLoS Computational Biology* 2013, **9:**e1003307.

3. Hebb DO: *The Organization of Behaviour, 1st Edition.* New York: Wiley; 1949.

4. Abraham WC: How long will long term potentiation last? *Phil. Trans. R. Soc. B* 2003, **258:**735–744.

5. Frey U, Morris R: Synaptic tagging and long-term potentiation. *Nature* 1997, **385:**533–536.

6. Redondo R, Morris RGM: Making memories last: the synaptic tagging and capture hypothesis. *Nat. Rev. Neurosci.* 2011, **12:**17–30.

7. Sajikumar S, Navakkode S, Frey JU: Identification of compartment- and process-specific molecules required for “synaptic tagging” during long-term potentiation and long-term depression in hippocampal CA1. *J. Neurosci.* 2007, **27:**5068–5080.

8. Barrett AB, Billings G, Morris RGM, van Rossum MC: State based model of long-term potentiation and synaptic tagging and capture. *PLoS Comput. Biol.* 2009, **5:**e1000259.

9. Clopath C, Ziegler L, Vasilaki E, Büsing L, Gerstner W: Tag-trigger-consolidation: a model of early and late long-term potentiation and depression. *PLoS Comput. Biol.* 2008, **4:**e10000248.

10. Li Y, Kulvicius T, Tetzlaff C: Induction and consolidation of calcium-based homo- and heterosynaptic potentiation and depression. *PLoS One* 2016, **11:**e0161679.

## P268 Why working memory is not a reservoir: the role of transient dynamics and attractors when processing unreliably timed inputs

### Timo Nachstedt^1,2^, Christian Tetzlaff^1,2^

#### ^1^Third Institute of Physics, Universität Göttingen, Göttingen, 37077, Germany; ^2^Bernstein Center for Computational Neuroscience, Göttingen, 37077, Germany

##### Correspondence: Timo Nachstedt (timo.nachstedt@phys.uni-goettingen.de)

*BMC Neuroscience* 2017, **18** (**Suppl 1**):P268

Working memory (WM) refers to the ability of humans and animals to store as well as to process the continuously incoming stream of stimuli and information on short time scales [1]. The neuronal dynamics implementing these two core functions of WM, to store and to process information, are still a matter of debate. In particular, it is unclear whether working memory relies on attractor dynamics [2] or whether it is realized by transient dynamics [3]. Several pieces of experimental evidence as well theoretical considerations provide support for both of these seemingly contradictory hypotheses.

Here, we approach this debate by considering the unreliability of the timing of the stimuli received by the WM. Quite obviously, when interacting with the environment, subjects cannot rely on precisely timed input stimuli. The consequence of unreliability of input stimuli on the operation of WM has been psychologically studied on subjects performing the N-back task. It has been found that, in this task, introducing unpredictability of the occurrence timing of the stimuli does not significantly influence the subject’s performance [4]. Based on this finding, we investigate which kind of neuronal dynamics enables a network to perform the N-back task with a comparable level of robustness with respect to variances in the stimuli timing.

The most widely used network model of transient neuronal dynamics is the framework of reservoir networks [5, 6]. We test the performance of reservoir networks trained with different learning algorithms and with different feedback topologies on the N-back task. Interestingly, we find that introducing already small variations in the timing of the input stimuli reduces the performance of reservoir networks in the N-back task significantly. We show that the performance can be restored by explicitly training the network to represent past input stimuli via the activity states of feedback loops. As this, in turn, effectively introduces attractor states into the network, we conclude that only by exploiting the properties of both, attractor states as well as of transient dynamics, a neuronal network is able to achieve a performance comparable to the one found in working memory experiments. Task-relevant information is stored in attractor states and processing of information is accomplished by transient dynamics. As a consequence, we predict that in the N-back task, an explicit recall stimulus should avoid a drop in performance resulting from introducing delays between the current stimulus and the execution of the respective action. Thus, we provide an experimentally verifiable hypothesis about the underlying dynamics of WM ruling out pure transient reservoir networks as a plausible model.

**References**

1. Baddeley AD: Working memory: theories, models, and controversies. *Annu Rev Psychol* 2012, **63:**1–29.

2. Riley MR, Constantinidis C: Role of prefrontal persistent activity in working memory. *Front Syst Neurosci* 2016, **9**:181.

3. Barak O, Sussillo D, Romo R, Tsodyks M, Abbot LF: From fixed points to chaos: three models of delayed discrimination. *Prog Neurobiol* 2013, **103:**241–222.

4. Koppe G, Gruppe H, Sammer G, Gallhofer B, Kirsch P, Lis S: Temporal unpredictability of a stimulus sequence affects brain activation differently depending on cognitive task demands. *Neuroimage* 2014, **101:**236–244.

5. Jaeger H: The “echo state” approach to analyzing and training recurrent neural networks. Tech. Rep., GMD - German National Research Institute for Computer Science; 2001.

6. Maass W, Natschläger T, Markram H: Real-time computing without stable states: a new framework for neural computation based on perturbations. *Neural Comput* 2002, **14:**2531–2560.

## P269 Application of spike train synchrony measure *Spike*-*contrast* to quantify the effect of bicuculline on cortical networks grown on microelectrode arrays

### Manuel Ciba^1^, Andreas Bahmer^2^ and Christiane Thielemann^1^

#### ^1^Biomems lab, Faculty of Engineering, UAS Aschaffenburg, 63743 Aschaffenburg, Germany; ^2^Comprehensive Hearing Center, University ENT-Clinic Würzburg, 97080 Würzburg, Germany

##### Correspondence: Manuel Ciba (manuel.ciba@h-ab.de)

*BMC Neuroscience* 2017, **18** (**Suppl 1**):P269

In neurosciences, it is assumed that neuronal information is mainly coded in the timing of spikes which is why spike trains are typically the data base for further analyzing procedures [1]. Synchrony is an important parameter since it is related to basic brain functions [2, 3] as well as to pathological states [4]. In order to quantify synchrony between two or more spike trains, several methods have been established, being either time scale dependent or time scale independent.

The use of a new time scale independent and multivariate synchrony measure is proposed, called *Spike*-*contrast,* with the aim to apply it on spike train data sets recorded from cortical networks with a biochemically induced synchrony increase. The histogram based approach, calculates a visual contrast of a raster plot across different time scales to quantify synchrony and is computational efficient when dealing with large number of parallel spike trains, which makes it suitable for the analysis of big data volumes recorded from high-density microelectrode arrays (MEA) Although its basic principal is different to existing time scale independent measures like *SPIKE*-*distance* [5], synchrony values of *Spike*-*contrast* and *SPIKE*-*distance* show a high correlation for test data from Poisson spike models and Izhikevich networks. However, their results diverge, when applied to spike train data containing synchronized bursts made of non-synchronized spikes: Whereas *SPIKE*-*distance* considers all time scales equally important [6] (and therefore is also sensitive to non-synchronized spikes in bursts), *Spike*-*Contrast* considers such spike trains perfectly synchronized.

The reflection of larger time scales in the range of burst duration has been suggested before by [7, 8] to analyze bicuculline induced synchrony modification. Here, the new algorithm *Spike*-*contrast,* prioritizing large time scales, shall be applied on experimental data recorded from cortical rat neurons grown on microelectrode arrays (MEA). Signals were recorded in a control situation and with bicuculline (10 µM), a well-known drug that blocks the inhibitory action of GABA_A_ receptors.

We find that *Spike*-*contrast* is able to significantly quantify the increase in synchrony induced by bicuculline. Statistical significance is higher than from other synchrony measures like *SPIKE*-*distance*, suggesting that the bicuculline mediated synchrony increase is more distinct in larger time scales and that *Spike*-*contrast* is an appropriate synchrony measure for spike trains taken from experimental data, e.g. in biosensor applications [9].

**References**

1. Rieke F: Spikes: exploring the neural code. MIT press, 1999.

2. Engel AK, Fries P, Singer W: Dynamic predictions: oscillations and synchrony in top–down processing. Nature Reviews Neuroscience, **2(10):**704–716, 2001.

3. Ward LM: Synchronous neural oscillations and cognitive processes. Trends in cognitive sciences, **7(12):**553–559, 2003.

4. Uhlhaas PJ, Singer W: Neural synchrony in brain disorders: relevance for cognitive dysfunctions and pathophysiology. Neuron, **52(1):**155–168, 2006.

5. Kreuz T, Chicharro D, Houghton C, Andrzejak RG, Mormann F: Monitoring spike train synchrony. Journal of neurophysiology, **109(5):**1457–1472, 2013.

6. Satuvuori E, Mulansky M, Bozanic N, Lenk K, Kreuz T: Measures of spike train synchrony for data with multiple time-scales. arXiv preprint arXiv:1702.05394, 2017.

7. Selinger JV, Pancrazio JJ, Gross GW: Measuring synchronization in neuronal networks for biosensor applications. Biosensors and Bioelectronics, **19(7):**675–683, 2004.

8. Chiappalone M, Vato A, Berdondini L, Koudelka-Hep M, Martinoia S: Network dynamics and synchronous activity in cultured cortical neurons. International journal of neural systems, **17(02):**87–103, 2007.

9. Flachs D, Ciba M: Cell-based sensor chip for neurotoxicity measurements in drinking water. Lékař a Technika - Clinician and Technology, **46(2):**46–50, 2016.

## P270 Cortical states affect the optimal linear readout of network dynamics

### Eric S. Kuebler^1^, Joseph S. Tauskela^2^, & Jean-Philippe Thivierge^1^

#### ^1^School of Psychology, Faculty of Social Science, University of Ottawa, Ottawa, Ontario, Canada; ^2^Human Health Therapeutics, National Research Council of Canada, Ottawa, Ontario, Canada

##### Correspondence: Eric S. Kuebler (ekueb021@uottawa.ca)

*BMC Neuroscience* 2017, **18** (**Suppl 1**):P270

Spontaneous neuronal activity in vitro is often characterized by network bursts, whereby a large proportion of neurons are active in close temporal contiguity. How this network state affects the optimal linear readout of neuronal dynamics remains an unresolved question in neuroscience. Here, we recorded from dissociated cortical neurons using multi-electrode arrays (MEAs) and computed a ‘spike-triggered average’ of population activity for each electrode (*N* = 59), by measuring the probability of co-occurrences between the spiking activity on the electrode of interest and that recorded on each of the other electrodes, termed the ‘preferred network state’. Results show that despite fluctuations in spontaneous activity over time, population activity over all electrodes can be described by a low-dimensional attractor with *N*-1 parameters, substantially fewer than the number of parameters required for pairwise correlations (*N* ^2^). To test whether activity across different networks could be accurately discriminated, preferred network states from the first half of recordings (10 min) were fed into a linear model trained with a Fisher criterion. Then, the model was tested by presenting it spiking activity from six networks sequentially. We found that this model was useful in successfully discriminating amongst different networks with less than 3% error rate (Figure 1A). Further, the linear model was robust to adjustments in the number of electrodes included for input to the LDA (Figure 1B), suggesting that fewer than *N*-1 parameters can be useful to accurately discriminate between networks. Using simulations of neural activity in a branching model, we show that network activity near a critical regime (but not necessarily at the exact critical point) is optimally discriminated by a linear readout.

**Conclusion:** Overall, our results point to a dynamical signature for representations of cortical activity whereby states near the critical point are most amenable to decoding by linear downstream structures. Computation in the brain may occur by distributed processing whereby several subnetworks are each responsible for contributing to dynamical neuronal representations.
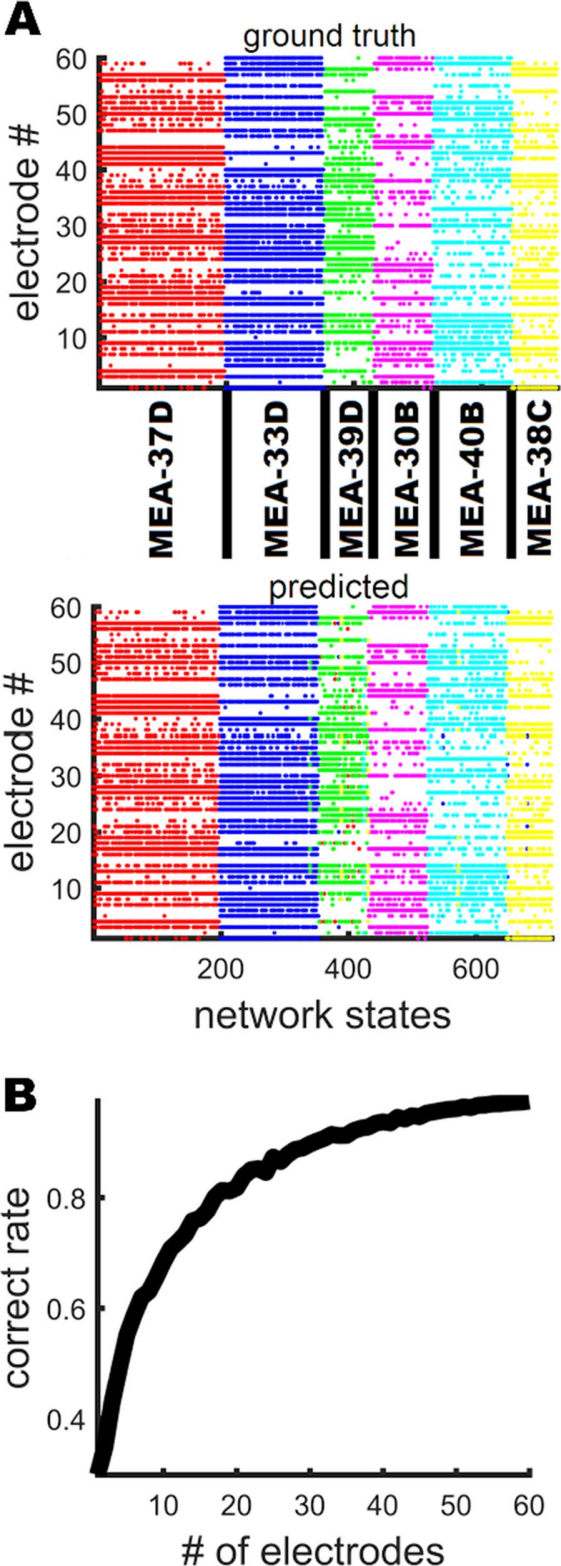


**Figure 1. A.** Preferred network states of several networks, denoted by colour. Top panel: ground truth data. Bottom panel: predictions based on the linear model. **B.** Correct rate of classification as a function of the number of electrodes used for input

## P271 Workflow, data format and tools to register neuron morphologies to a reference brain atlas

### Rembrandt Bakker^1,2^, María García-Amado^3^, Marian Evangelio^3^, Francisco Clascá^3^, Paul Tiesinga^1^

#### ^1^Neuroinformatics department, Donders Institute for Brain, Cognition and Behaviour, Radboud University Nijmegen, Nijmegen, The Netherlands; ^2^Institute of Neuroscience and Medicine (INM-6) and Institute for Advanced Simulation (IAS-6) and JARA BRAIN Institute I, Jülich Research Centre, Jülich, Germany; ^3^Departamento de Anatomía, Histología y Neurociencia, Facultad de Medicina, Universidad Autónoma de Madrid, Madrid, Spain

##### Correspondence: Rembrandt Bakker (r.bakker@donders.ru.nl)

*BMC Neuroscience* 2017, **18** (**Suppl 1**):P271

Neuronal reconstructions are essential building blocks for neuronal tissue simulation at subcellular resolution. Several databases with thousands of reconstructed morphologies exist, the largest being NeuroMorpho.Org [1]. In this database, the location of each neuron is described in terms of species and brain region. For a neuronal tissue builder like the one created in the Human Brain Project (HBP), this information is too coarse. Only neurons that are registered into a spatial reference system can be used. Here we present a workflow and accompanying tools to do this, based on the manual annotation of a few points on the neuron.

Our use-case is the atlas registration of long-range projection neurons in mouse, which is particularly challenging since the neurons run across many brain regions. The steps of the workflow are:

1. The experimental lab uses the widely used Neurolucida (MBF BioScience) software to reconstruct the neuron. For a small set of points, the location is eye-balled in the Franklin-Paxinos mouse brain atlas [2], and added to the reconstruction as a marker point.
2. The Neurolucida file (choice of binary, asci, xml) is read by a newly written open-source parser (https://www.npmjs.com/package/morphology_io), and converted to a newly developed SWC + format (https://github.com/HumanBrainProject/swcPlus), an extension of the widely used SWC format [3].
3. The markers with atlas coordinates are extracted from the SWC + file by a python script and used to estimate an affine transformation that maps the local coordinate system to the reference space. The transformation parameters are saved to the SWC + file.
4. The Morphology Viewer [4], a new web-based morphology suite, recognizes the transformation parameters in the file, and displays the neuron along with sections from the atlas, see screenshot in Fig. 1.

In the use-case, 50 points were manually mapped to the atlas. To accelerate the procedure, this can be reduced to about 10 points. An alternative approach to this workflow is investigated in sub-project 5 of the HBP. It uses a set of tissue-sections from which the neuron was reconstructed to semi-automatically find a similar location in the reference atlas using the AligNII tool (http://www.nesys.uio.no/AligNII/).
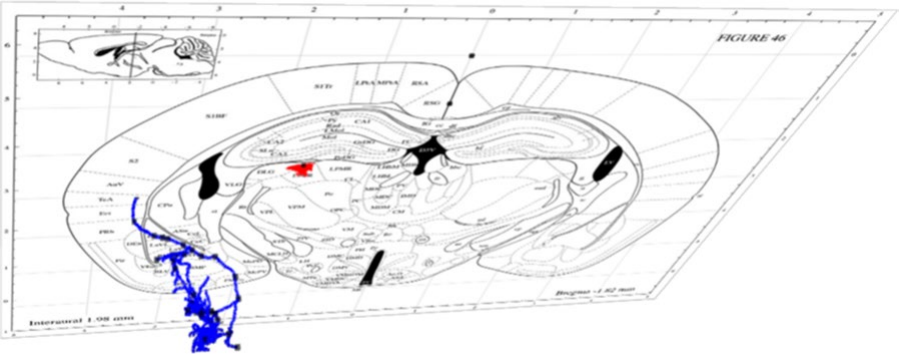


**Figure 1.** Registered neuron with soma and dendrites (red) in region LPLR and the main axonal arbor (blue) in area V1

**Acknowledgements**

Supported by the European Union Seventh Framework Programme (FP7/2007-2013) under grant agreement numbers 604102 (HBP RUP) and 720270 (HBP SGA1).

**References**

1. Halavi M, Polavaram S, Donohue DE, et al.: NeuroMorpho.org implementation of digital neuroscience: dense coverage and integration with the NIF. *Neuroinformatics* 2008, **6**(3):241–252. doi:10.1007/s12021-008-9030-1.2.

2. Paxinos G, Franklin K: *the Mouse Brain in Stereotaxic Coordinates, 4* ^*th*^ *Edition.* Academic Press; 2013.

3. Cannon RC, Turner DA, Pyapali GK, Wheal HV: An on-line archive of reconstructed hippocampal neurons.

*J Neurosci Methods* 1998, **84**(1–2):49–54. doi: 10.1016/S0165-0270(98)00091-0.

4. HBP Neuron Morphology Viewer [http://neuroinformatics.nl/HBP/morphology-viewer/]

## P272 Simple models of closed-loop cortical-environment interactions

### Christopher L. Buckley^1^, Taro Toyoizumi^2^

#### ^1^Informatics, University of Sussex, Brighton, BN1 9RH, UK; ^2^Brain Science Institute, RIKEN, Wako, Saitama. 351-0106, Japan

##### Correspondence: Christopher L. Buckley (c.l.buckley@sussex.ac.uk)

*BMC Neuroscience* 2017, **18** (**Suppl 1**):P272

Many investigations of the neural circuits underlying behaviour have commonly started from the assumption that the brain is an input/output device. On this view, the brain operates in an *open*-*loop,* mapping sensory input caused by the environment (*exafferent input*) to appropriate motor output. However, during active behaviour (e.g., running, whisking, swimming) sensory input is directly shaped by motor actions and sensory perceptions inform future motor commands forming a *closed*-*loop* between the brain and environment, see Fig. 1.
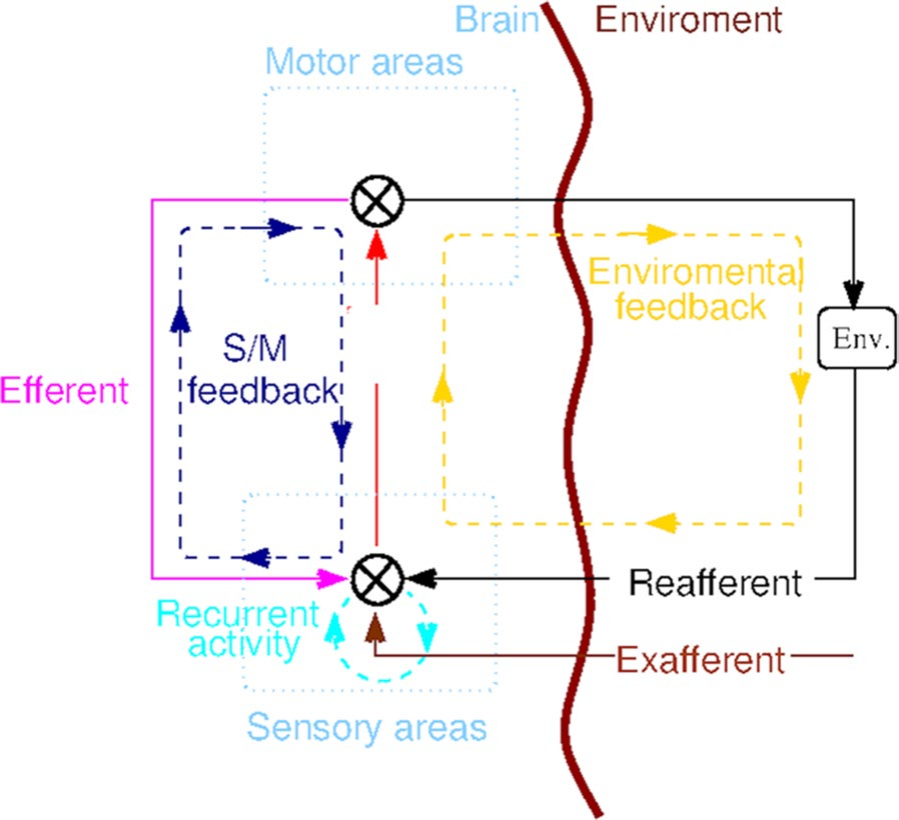


**Figure 1.** A schematic of brain/body/environment interaction during active behaviour. Motor actions on the body/environment impact on sensory areas as reafferent input (black arrow) which is combined with exafferent input (brown arrows) to form the sensory stream. Motor areas also send efferent signals, or corollary discharges, to sensory systems (magenta arrows). Further during active behavior sensory input also informs motor output (red arrow). Thus, neuronal dynamics in sensory areas are affected by three forms of feedback: environmental feedback (yellow dashed), internal sensor/motor feedback (blue dashed) and endogenous recurrent feedback (cyan dashed)

The onset of active behaviours coincides with marked changes in cortical dynamics. Typically, synchronous fluctuations of neural activity are strongly modulated by [2–4]. Further active behaviours shape neuronal responses. For example, the onset of running sharpens response is visual cortex [4] but suppresses responses in auditory cortex [3]. In the barrel cortex responses to brief whisker perturbations are suppressed by whisking but responses to active touch events (when the whisker is actively driven into an object) are enhanced [5]. By analysing simple dynamical models, we examine to what extent these phenomena can be accounted for by the closed-loop feedback circuits necessary for active behaviour, see Fig. 1. In particular, we argue that the onset of these feedback loops suggests a parsimonious account of the changes to synchronous neuronal fluctuations and sensory responses caused by the presence of active behaviour and can account for mismatch responses caused by the interruption of environmental feedback. Lastly we discuss the development of an experimental setup to test these ideas that utilizes closed-loop interactions in larval zebrafish behaving in a closed-loop virtual reality.

**References**

1. von Holst E. Relations between the central Nervous System and the peripheral organs. The British *Journal of Animal Behaviour* 1954, **2**:89–94.

2. Crochet S, Petersen CCH. Correlating whisker behavior with membrane potential in barrel cortex of awake mice*. Nat Neurosci*. 2006, **9**: 608–610.

3. Schneider DM, Nelson A, Mooney R. A synaptic and circuit basis for corollary discharge in the auditory cortex. *Nature* 2014, **513**: 189–194.

4. Niell CM, Stryker MP. Modulation of visual responses by behavioral state in mouse visual cortex. *Neuron*. 2010, **65**: 472–479

5. Crochet S, Poulet JFA, Kremer Y, Petersen CCH. Synaptic mechanisms underlying sparse coding of active touch. *Neuron* 2011, **69**:1160–1175.

## P273 Encoding multiple spaces in grid-cells networks

### Alexis M. Dubreuil^1^, Rémi Monasson^1^, Alessandro Treves^2^

#### ^1^Laboratoire de Physique Théorique, Ecole Normale Supérieure, Paris, France; ^2^Cognitive Neuroscience, SISSA, Trieste, Italy

##### Correspondence: Alexis M. Dubreuil (alexis.dubreuil@gmail.com)

*BMC Neuroscience* 2017, **18** (**Suppl 1**):P273

Grid-cells found in entorhinal cortex form a representation of space through their receptive fields that pave space with triangular lattices. It has been proposed that the firing of grid-cells with similar grid spacing is supported by recurrent connectivity, such that network dynamic lies on a 2-D manifold of stable states [1]. Recent empirical evidence suggests that the grid-cell code is not limited to physical space encoding in entorhinal cortex, but could also subserve encoding of more abstract spaces in other cortical areas [2]. In order to assess the efficiency of neural networks in using a grid-cell code, the present study is focused on the problem of counting the number of grid-like manifolds that can be reliably embedded in a neural network, this number being referred to as the storage capacity of the network. We consider a network of binary neurons in which each manifold is imprinted in the connectivity matrix via a Hebbian component (neurons with similar receptive fields in a manifold are connected). We compute the number of stable manifolds by extending replica calculations developed in [3] for a model of place cells. Such analytical calculations allow us to explore the performance of various grid-cell codes. We focus on two characteristics of such codes. First, we explore how the typical paving of space in a manifold impact network’s storage capacity (see Figure 1 A and B). Second we explore how the storage capacity depends on the parameters of the Hebbian rule shaping the connectivity matrix. Overall, our study suggests that a grid-cell code can make an efficient use of neural resources since up to 200 stable manifolds can be imprinted in the connectivity matrix of a network of 10,000 neurons (see Figure B), roughly the size of a cortical column.
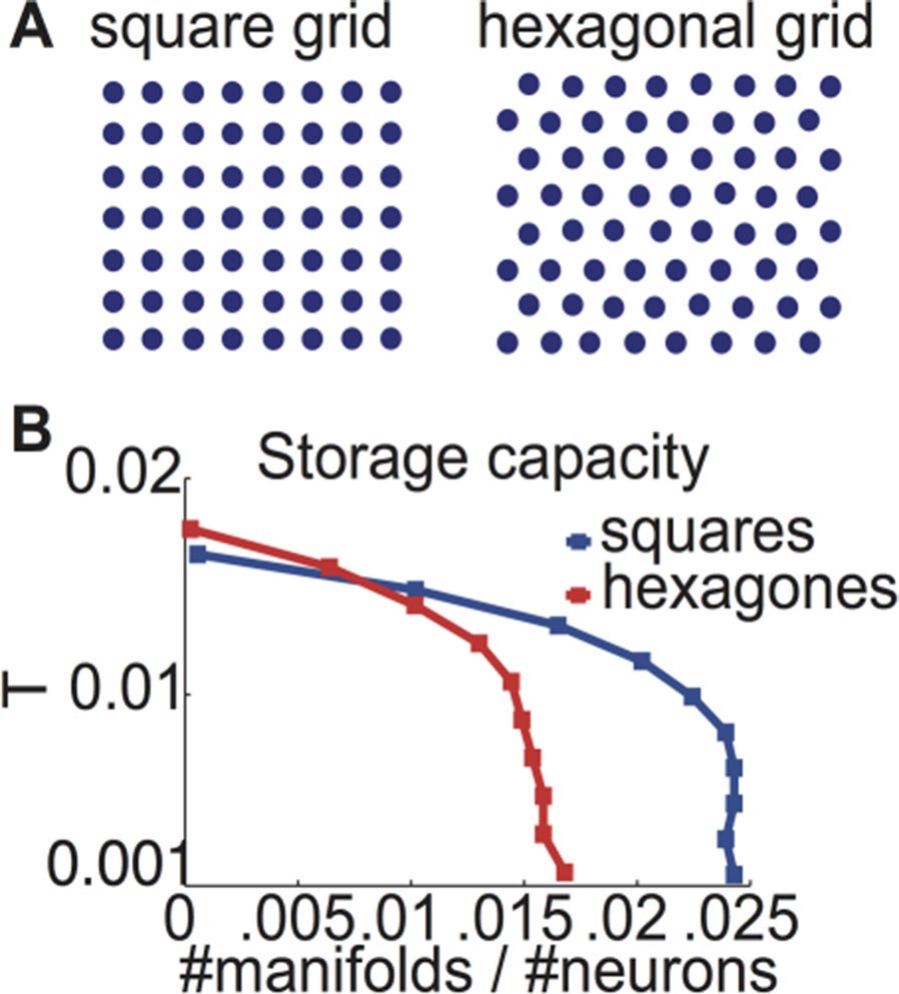


**Figure 1. A**. Example of the receptive field of a neuron that paves the 2-D flat space. We implement different kinds of paving, for instance a square lattice (left), or a hexagonal lattice (right) as those found in entorhinal cortex. **B**. Storage capacity of the network as a function of the amount of thermal noise T in the network’s dynamics. For a given T, the lines give the maximal number of stable manifolds the network can support, given the number of neurons it is composed of

**References**

1. Burak Y, Fiete IR: Accurate path integration in continuous attractor network models of grid cells. *PLoS Computational Biology* 2009, **5(2):** e1000291. doi:10.1371/journal.pcbi.1000291

2. Constantinescu AO, O’Reilly JX, Behrens TEJ: Organizing conceptual knowledge in humans with a gridlike code. *Science* 2016, **352(6292):**1464–1468.

3. Monasson R, Rosay S: Crosstalk and transitions between multiple spatial maps in an attractor neural network model of the hippocampus: Phase diagram. *Phys Rev E* 2013, **87:**062813.

## P274 Storage capacity of threshold-linear networks for gridlike continuous attractors

### Davide Spalla, Sophie Rosay, Alessandro Treves

#### Cognitive Neuroscience Sector, SISSA, Trieste, 34136, Italy

##### Correspondence: Davide Spalla (dspalla@sissa.it)

*BMC Neuroscience* 2017, **18** (**Suppl 1**):P274

Attractor neural networks play an important role in modeling the mechanisms of spatial memory, allowing for analytical and computational analyses of the recurrent circuitry of hippocampal and cortical networks known to be involved in the cognitive representation of space.

Here we study a recurrent network of threshold-linear neurons, and its capacity for storing multiple spatial maps as continuous attractors.

In this model, storage capacity can be analytically evaluated in the mean field approximation, as shown in [1].

The existence of a retrieval phase reduces to the existence of the solutions of a single equation, which disappear at a critical value of the storage load. This result holds both for a sparse activity model suitable for a plausible description of CA3 place cells, and for a toy model version with periodic boundary condition on a two-dimensional torus.

Imposing now periodic hexagonal boundary condition on the connectivity allows us to model grid cell like behavior, and to calculate the regime in which the system can retrieve and/or maintain a representation of position in one of the stored environments. Surprisingly, the storage capacity appears to be very much higher than in the “square” toy model, by several orders of magnitude.

In graded-response networks, however, mixed states of multiple attractors (corresponding to recall of multiple environments) are known to also exhibit stability, even though in a limited region of parameter space [2].

Also in our “hexagonal continuous attractor” network we find, in addition to the “classical” retrieval behavior, a regime in which external cues trigger retrieval in more than one map simultaneously, in a similar way as described in [3] for two 1D periodic manifolds.

Our study generalizes these results to 2-dimensional uncorrelated maps, and extends them to the case with a large number of stored maps.

**References**

1. Battaglia F, Treves A: Attractor neural networks storing multiple space representations: A model for hippocampal place fields. *Physical Review E.* 1998, **58(6):**7738.

2. Roudi Y, Treves A: Disappearance of spurious states in analog associative memories. *Phys Rev E Stat Nonlin Soft Matter Phys*. 2003, **67**(4 Pt 1):041906.

3. Romani S, Tsodyks M: Continuous attractors with morphed/correlated maps. *PLoS Comput Biol.* 2010; **6(8)**

## P275 A network model of the turnover dynamics of excitatory and inhibitory synapses in cortex

### Florence I. Kleberg, Jochen Triesch

#### Neuroscience Lab, Frankfurt Institute for Advanced Studies, Frankfurt am Main, Hessen, 60438, Germany

##### Correspondence: Florence I. Kleberg (kleberg@fias.uni-frankfurt.de)

*BMC Neuroscience* 2017, **18** (**Suppl 1**):P275

It has long been known that there is substantial turnover of excitatory synapses in cortex during both development and adult life [1]. Recent experiments using markers for GABAergic synapses have shown that inhibitory synapses are also highly dynamic [2–3]. Specifically, inhibitory synapses exhibit approximately exponentially decreasing survival fractions and show reduced lifetimes when sensory input is decreased [3]. Here we show that such dynamics of excitatory and inhibitory synapses result from a combination of structural plasticity, Spike-Timing Dependent Plasticity (STDP), and multiplicative normalisation in a Self-Organizing Recurrent Neural Network (SORN; [4]) of Leaky Integrate-and-Fire neurons with membrane noise and external Poisson inputs. Both synapse types are grown from an initially unconnected network state by random synapse creation. Synapses whose efficacies fall below a threshold are pruned. We find that excitatory and inhibitory synaptic weights develop lognormal-like distributions as observed experimentally [2] and that the lifetimes of synapses follow a power law-like distribution. Furthermore, we find that the fraction of surviving inhibitory synapses decays approximately exponentially as observed experimentally ([3]; Figure 1A) and is modulated by the strength of potentiation (LTP) and depression (LTD) in the inhibitory STDP rule. Finally, depriving the network of external input decreases the survival fraction of inhibitory synapses as observed in vivo ([3]; Figure 1A). To gain deeper insight into the underlying mechanisms we formulate a statistical model of the time evolution of synaptic efficacies and find that it well describes the power law-like lifetimes and exponential-like decreasing survival fractions (Figure 1B). We conclude that the experimentally observed turnover dynamics of inhibitory synapses can be explained by local, biologically plausible plasticity mechanisms and are well described by a simple stochastic model.
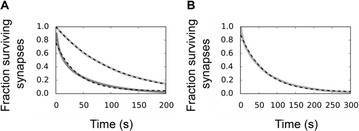


**Figure 1. A.** Survival fractions of inhibitory synapses in the SORN are shown by the solid grey lines. Light grey, with external input to the network. Dark grey, without external input. The exponential fit is shown by the dotted line. **B.** Survival fractions of weights in a simple stochastic model are shown by the solid grey line. The exponential fit is shown by the dotted line. The time scale of plasticity in the simulations in A and B is sped up compared to experimental findings in order to save computation time

**References**

1. Berry KP, Nedivi E: Experience-dependent structural plasticity in the visual system. *Annu. Rev. Vis. Sci.* 2016, **2**:17–35.

2. Rubinski A, Ziv NE: Remodeling and Tenacity of Inhibitory Synapses: Relationships with Network Activity and Neighboring Excitatory Synapses. *PLoS Comput Biol* 2015, **11(11)**: e1004632.

3. Villa KL, Berry KP, Subramanian J, Cha JW, Oh WC, Kwon H, Kubota Y, So PTC, Nedivi E: Inhibitory Synapses are repeatedly assembled and removed at persistent sites in vivo. *Neuron* 2016, **89(2)**:756–789.

4. Lazar A, Pipa G, Triesch J: SORN: a self-organizing recurrent neural network. *Front Comp Neurosci* 2009, **3**:23

## P276 Retinal prostheses from stimulation to seeing

### Willy Wong^1,2^, Bruno de Oliveira Floriano^1^, Toshihiko Matsuo^3^, Tetsuya Uchida^4^

#### ^1^Dept. of Electrical and Computer Engineering, University of Toronto, Toronto, ON, M5S3G4, Canada; ^2^Institute of Biomaterials and Biomedical Engineering, University of Toronto, Toronto, ON, M5S3G9, Canada; ^3^Ophthalmology, Okayama University Medical School, Okayama, 700-8558, Japan; ^4^Polymer Materials Science, Faculty of Engineering, Okayama University, Okayama, 700-8530, Japan

##### Correspondence: Willy Wong (willy.wong@utoronto.ca)

*BMC Neuroscience* 2017, **18** (**Suppl 1**):P276

A major concern with the design of any prosthetic device is the need for objective evaluation. Is there a way to better evaluate what the person senses without resorting to subjective testing after implantation? This study addresses a new approach to retinal prosthetic design, together with detailed modelling of retinal function to tackle the question of what is a person actually “sees”. Retinitis pigmentosa and age-related macular degeneration can lead to blindness due to loss of the photoreceptor layer. The Okayama University-type Retinal Prosthesis (OUReP) is a new approach to retinal implant design which does not involve stimulation via micro-electrode arrays [1]. Instead a thin film consisting of photosensitive dye molecules attached to a polyethylene layer is placed at back of the eye. The dye is designed to respond only in the visible wavelengths and generates an electric potential in response to incident light likely due to the formation of dipoles. Currently OUReP has been successfully tested in rats with a next step in rabbit implantation. A current problem is understanding how the dye works to facilitate vision.

We have been studying the effect of the potential on the visual pathway by first modelling the physiological system as an electromagnetic boundary value problem. The potential was solved computationally in COMSOL to obtain the extracellular potential outside the retinal cells. This is what generates activity in the retinal cells and ultimately drives action potentials in the optic nerve. The neural activity was then solved using Hodgkin-Huxley equations and the cable model with the ‘activating function’ [2] derived from the extracellular potential solved from Laplace’s equation. From this, we found that ganglion cells are excited by stimulating the OUReP photosensitive dye layer at ambient light levels.

The next step involves exploring the neural code behind ‘seeing’. A theory of sensory information processing has been in development for nearly 50 years [3] and provides, perhaps for the first time, an accurate model of the firing rate response in primary afferent neurons [4]. This approach is based on the entropy of the sensory signal and calculates the response due to uncertainty associated with the input. The theory has been shown to work well across different modalities as well as for different animal species. When applied to the retina, a single equation of 5 parameters together with a coupling equation provides a good estimate of the potential of retinal bipolar cells, and also the average spike rate response of retinal ganglion cells for both on-centre and off-centre cells. The population of retinal cells is diverse, and it is not likely that a simple model can encompass the entirety of the physiological response. However, this is a first step in understanding the quantitative functioning of the retina. By combining the physiological response solved through the activating function, and comparing this with predictions from entropy theory, we can better estimate what a person will see, and thus provide a more objective assessment of implant function as well as guide future development.

**Acknowledgements**

This work was supported by a grant from Natural Science and Engineering Research Council of Canada to WW. BF acknowledges the support of Ciências sem Fronteiras.

**References**

1. Alamusi, Matsuo T, Hosoya O, et al. Vision maintenance and retinal apoptosis reduction in RCS rats with Okayama University-type retinal prosthesis (OUReP™) implantation. *J Artif Organs*. 2015, **18(3):**264–271.

2. Werginz P, Benav H, Zrenner E, Rattay F. Modeling the response of ON and OFF retinal bipolar cells during electric stimulation. *Vision Res* 2015, **111(Pt B):**170–181.

3. Norwich, K.: Information, Sensation and Perception. *New York: Academic Press;* 1993.

4. Wong W. On the Asymptotic, Near-Equilibrium Sensory Response. *arXiv*. 2013 **:1307.6445**

## P277 Metabolic cost of neuronal oscillations

### Domenica Dibenedetto^1^, Kâmil Uludağ^1,2^

#### ^1^Maastricht Centre for Systems Biology (MaCSBio), Maastricht University, Maastricht, The Netherlands; ^2^Maastricht Brain Imaging Centre (MBIC), Faculty of Psychology & Neuroscience, Maastricht University, Maastricht, The Netherlands

##### Correspondence: Domenica Dibenedetto (domenica.dibenedetto@maastrichuniversity.nl)

*BMC Neuroscience* 2017, **18** (**Suppl 1**):P277

The association between cognitive functions in humans, the increase in glucose and oxygen utilization and the expression of energy metabolism genes is an active area of research [1]. The human brain accounts for at least 20% of the body’s energy consumption [2]. Much of the brain’s energy use goes on to re-establish electrochemical gradients following action potentials and synaptic currents [3, 4]. The high energy demand at the synapse implies that local mechanisms must exist to sense synaptic activity and provide the energy substrates necessary to sustain pre- and postsynaptic processes. Here, we investigate the relationship between ionic currents associated with neuronal activity at the synaptic site and the brain energy consumption using both experimental data and mathematical models. Using a bottom-up approach, we model a recurrent network of excitatory-inhibitory neurons, stimulated with realistic dynamic inputs, in order to determine the metabolic costs of neuronal oscillations [5]. An important early finding from studies in rat was that energy use by neurons (oxidative glucose consumption) is linearly correlated to excitatory neuronal activity (glutamate release) [6]. As first step, we are investigating how the various ionic currents measured at the excitatory synaptic site respond at different frequency ranges of a square-pulsed input signal through a parameters space exploration study. A morphologically and functionally realistic pyramidal neuron is considered as postsynaptic compartment [7]. This new physiologically inspired conductance-based [4] neuron-model can be the basis of a more complex network in order to monitor metabolism at micro-circuit level. The result of the modelling will be linked to non-invasive neuroimaging modalities, such as fMRI and EEG [8, 9], which are related to either the local metabolic costs of neuronal activity or local synchronicity of the microcircuit. Studying the mechanisms of brain metabolism is of great interest in order to understand not only the fundamental physiological phenomena of brain functions, but also the significance of alterations in functional brain imaging signals detected in several neurodegenerative disorders affecting cognitive processes.

**References**

1. Magistretti, Pierre J. and I. Allaman, A Cellular Perspective on Brain Energy Metabolism and Functional Imaging. *Neuron*. **86(4):** p. 883–901.

2. Attwell, D. and S.B. Laughlin, An energy budget for signaling in the grey matter of the brain. *J Cereb Blood Flow Metab,* 2001. **21(10):** p. 1133–45.

3. Harris, Julia J., R. Jolivet, and D. Attwell, Synaptic Energy Use and Supply. *Neuron.* **75**(5): p. 762–777.

4. Hines, M.L. and N.T. Carnevale, The NEURON simulation environment. *Neural Comput*, 1997. **9(6):** p. 1179–209.

5. Mazzoni, A., et al., Understanding the relationships between spike rate and delta/gamma frequency bands of LFPs and EEGs using a local cortical network model. *Neuroimage*, 2010. **52(3):** p. 956–72.

6. Sibson, N.R., et al., Stoichiometric coupling of brain glucose metabolism and glutamatergic neuronal activity. *Proc. Natl. Acad. Sci. U.S.A*. **95**, 316–321.

7. Hay, E., et al., Models of Neocortical Layer 5b Pyramidal Cells Capturing a Wide Range of Dendritic and Perisomatic Active Properties. *PLoS Comput. Biol.,* 2011. **7**, e1002107.

8. Raichle, M.E. and M.A. Mintun, Brain work and brain imaging. *Annu Rev Neurosci*, 2006. **29**: p. 449–76.

9. Logothetis, N.K., et al., Neurophysiological investigation of the basis of the fMRI signal. *Nature*, 2001. **412(6843):** p. 150–7.

## P278 Multi-cluster structure and dynamics in networks of coupled phase oscillators through different classes of STDP profiles

### Abdorreza Goodarzinick^1^, Mojtaba Madadi Asl^1^, Alireza Valizadeh^1,2^

#### ^1^Department of Physics, Institute for Advanced Studies in Basic Sciences (IASBS), Zanjan 45137-66731, Iran; ^2^School of Cognitive Sciences, Institute for Research in Fundamental Sciences (IPM), Tehran 19395-5746, Iran

##### Correspondence: Abdorreza Goodarzinick (a.goodarzinick@iasbs.ac.ir)

*BMC Neuroscience* 2017, **18** (**Suppl 1**):P278

In the brain networks, strength of synaptic connections is adjusted according to relative spike timing of pre- and post- synaptic neurons, known as spike-timing-dependent plasticity (STDP) [1]. The theoretical and simulational studies have shown different emergent collective activity and patterns of connectivity when using different STDP profiles with different set of parameters. For example, depending on the parameter set, STDP can either promote or oppose synchrony [2]. It also might lead to potentiation of bidirectional connections or eliminate two-neuron loops by depressing one of the connections for every pair of reciprocally connected neurons [3].

Here, we have inspected how different biologically observed STDP profiles can result in different emergent dynamics. Based on Kuramoto model for description of the phase dynamics in neuronal ensembles [4, 5], we have designed classes of plasticity functions with symmetric and anti-symmetric profiles, mimicking typical forms of STDP for excitatory and inhibitory connections (see Figure 1). Related sets of differential equations for evolution of the phases and the synapses are analytically solved to justify results of numerical simulations. The main observation is that while anti-symmetric profile promotes one structural cluster with almost synchronized dynamics, symmetric profiles lead to multi-cluster structure with various phase relations within and between the clusters.
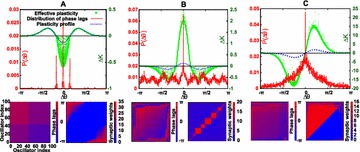


**Figure 1**. Steady-state distribution of phase differences and connection strengths which are emerged through the function of 3 different sets of plasticity profiles. In the upper panels the plasticity profile, distribution of phase lags and effective plasticity function which is obtained by convolution of the phase lags and the plasticity profile are shown. The final pattern of the phase lags and the synaptic weights between each pair of oscillators are shown in the bottom panels

**References**

1. Bi GQ, Poo MM: Synaptic modifications in cultured hippocampal neurons: dependence on spike timing, synaptic strength, and postsynaptic cell type. *J Neurosci* 1998, **18(24):** 10464–10472.

2. Lubenov E V, Siapas AG: Decoupling through synchrony in neuronal circuits with propagation delays. *Neuron* 2008, **58:**118–131.

3. Madadi Asl M, Valizadeh A, Tass PA: Dendritic and Axonal Propagation Delays Determine Emergent Structures of Neuronal Networks with Plastic Synapses. *Sci Rep* 2017, 7:39682.

4. Kuramoto, Y: *Chemical oscillations, waves, and turbulence.* Berlin: Springer; 1984.

5. Maistrenko YL, Lysyansky B, Hauptmann C, Burylko O, Tass PA: Multistability in the Kuramoto model with synaptic plasticity. *Phys Rev E* 2007, **75:**066207.

## P279 Multi-scale network structure of macaque visual cortex: connectivity map, cortical architecture, and layer-specific pathways

### Maximilian Schmidt^1,2^, Rembrandt Bakker^1,3^, Claus C. Hilgetag^4,5^, Markus Diesmann^1,6,7^, Sacha J van Albada^1^

#### ^1^Institute of Neuroscience and Medicine (INM-6) and Institute for Advanced Simulation (IAS-6), Jülich Research Centre and JARA, Jülich, Germany; ^2^Laboratory for Neural Circuit Theory, RIKEN Brain Science Institute, Wako, Japan; ^3^Donders Institute for Brain, Cognition and Behavior, Radboud University Nijmegen, Nijmegen, Netherlands; ^4^Department of Computational Neuroscience, University Medical Center Eppendorf, Hamburg, Germany ^5^Department of Health Sciences, Boston University, Boston, MA, USA; ^6^Department of Psychiatry, Psychotherapy and Psychosomatics, Medical Faculty, RWTH Aachen University, Aachen, Germany; ^7^Department of Physics, Faculty 1, RWTH Aachen University, Aachen, Germany

##### Correspondence: Sacha J van Albada (s.van.albada@fz-juelich.de)

*BMC Neuroscience* 2017, **18** (**Suppl 1**):P279

Extensive available axonal tracing data along with predictive connectomics allows a novel quantitative description of the network structure of macaque cortex. Since the effects of connectivity on network dynamics are influenced by the size of cortical populations, and since neuron density is predictive of connectivity [1, 2], it is relevant to also characterize numbers of neurons when deriving a connectivity map. In this study, we integrate data on cortical architecture and axonal tracing data into a multi-scale account of the network structure of macaque vision-related cortex. The resulting connectivity map predicts the connection probability between any two neurons based on their types, areas, and layers. Combining cell densities with published micrographs provides a quantification of the reduction of relative layer 4 thickness with cell density from structurally differentiated to less differentiated areas. Similarly, total cortical thickness decays with cell density. Under the assumption of a relatively constant density of synapses, this yields denser connectivity in structurally less differentiated areas. Combined anterograde and retrograde tracing data reveal that synaptic target patterns of corticocortical connections depend on the laminar origin of the projection in a manner that complements earlier accounts of the association between source and target patterns [3, 4]. Statistically assigning synapses to target neurons based on dendritic length in anatomical reconstructions [5] suggests that layer 4 neurons receive non-negligible feedback. Our layer-specific connectivity map enables a novel characterization of direct and polysynaptic pathways through the network. It can be tested in simulations and experiments whether these directionally specific paths open up channels for targeted corticocortical communication, akin to recently highlighted hierarchically differential oscillatory interactions [6, 7].

**Acknowledgements**

Helmholtz Portfolio Supercomputing and Modeling for the Human Brain (SMHB), European Union (BrainScaleS, grant 269921 and Human Brain Project, grant 604102), the Jülich Aachen Research Alliance (JARA), and the German Research Council (DFG grants SFB936/A1,Z1 and TRR169/A2).

**References**

1. Hilgetag CC, Medalla M, Beul SF, Barbas H: The primate connectome in context: principles of connections of the cortical visual system. *NeuroImage* 2016, **134:**685–702.

2. Beul SF, Barbas H, Hilgetag CC: A predictive structural model of the primate connectome. *arXiv preprint* 2015, arXiv:1511.07222.

3. Felleman DJ, Van Essen DC: Distributed hierarchical processing in the primate cerebral cortex. *Cereb Cortex* 1991, **1:**1–47.

4. Markov NT, Ercsey-Ravasz M, Van Essen DC, Knoblauch K, Toroczkai Z, Kennedy H: Cortical high-density counterstream architectures. *Science* 2013, **342:**1238406.

5. Binzegger T, Douglas RJ, Martin KA: A quantitative map of the circuit of cat primary visual cortex. *J Neurosci* 2004, **24:**8441–8453.

6. Van Kerkoerle T, Self MW, Dagnino B, Gariel-Mathis MA, Poort J, Van Der Togt C, Roelfsema PR: Alpha and gamma oscillations characterize feedback and feedforward processing in monkey visual cortex. *PNAS* 2014, **111(40),** 14332–14341.

7. Bastos AM, Vezoli J, Bosman CA, Schoffelen JM, Oostenveld R, Dowdall JR, De Weerd P, Kennedy H, Fries P: Visual areas exert feedforward and feedback influences through distinct frequency channels. *Neuron* 2015, 85: 390–401.

## P280 The interaction of synaptic and structural plasticity in recurrent networks

### Michael Fauth, Mark van Rossum

#### School of Informatics, University of Edinburgh, Edinburgh, United Kingdom

##### Correspondence: Michael Fauth (mfauth@gwdg.de)

*BMC Neuroscience* 2017, **18** (**Suppl 1**):P280

The connectivity of cortical networks determines the way they process information. Changes in the connectivity and, hence, in the information processing in response to certain stimuli are associated with learning and memory formation [1, 2]. There are two classes of activity-dependent processes that change the connectivity of cortical networks: synaptic plasticity, which changes the transmission efficacies or weights of the synapses, and structural plasticity, which creates and removes synapses. These processes are strongly interacting, for example, the lifetime of synapses depends on their synaptic weight and correlated quantities as the volume of the corresponding dendritic spine head [3, 4]. Hence, to understand how memories are stored in the connectivity, we analyze the interaction of synaptic and structural plasticity in recurrent networks. Moreover, we address the question how memories stored by the connectivity can be maintained on timescales of months and years, although the underlying synapses are removed or exchanged on the timescale of days [4, 5].

Using mean-field analysis and simulations, we show that the synapses in a population of recurrently connected neurons exhibit a collective dynamics which gives rise to two stable states: the population can be either weakly interconnected or strongly interconnected with synapses stabilizing each other. The population remains in its current state despite the creation or removal of individual synapses, such that information about the population state can be retained much longer than the lifetime of individual synapses. Moreover, the population can be brought to either state by changing the input stimulation. These results also extend to sub-populations of the network. For example, when providing a small subset of neurons in a network with a higher input current, this subset becomes highly interconnected, effectively forming a Hebbian cell assembly [6–8].

Interestingly, this collective dynamics can be implemented independent of the bistability of neuronal activities controlled by synaptic weights in recurrently connected populations, which has been proposed as a model of observed persistent activity [9]. As a consequence, even at low activities, a (sub-)population can remain connected with many synapses with relatively small weights. This, in turn, allows for a rapid increase of these weights upon retrieval or relearning, which might be related to Ebbinghaus’ savings phenomenon.

**References**

1. Yang G, Pan F, Gan WB: Stably maintained dendritic spines are associated with lifelong memories. *Nature* 2009, **462**: 920–924.

2. Xu T, Yu X, Perlik AJ, Tobin WF, Zweig JA, et al.: Rapid formation and selective stabilization of synapses for enduring motor memories. *Nature* 2009, **462**:915–919

3. Yasumatsu, N, Matsuzaki, M, Miyazaki, T, Noguchi, J & Kasai, H: Principles of Long-Term Dynamics of Dendritic Spines. *J Neurosci* 2008, **28**:13592–13608

4. Loewenstein, Y; Yanover, U, Rumpel, S: Predicting the dynamics of network connectivity in the neocortex. *J Neurosci* 2015, **35**:12535–12544

5. Fauth, M., Wörgötter, F., Tetzlaff, C.: Formation and Maintenance of Robust Long-Term Information Storage in the Presence of Synaptic Turnover. *PLoS Comput Biol* 2015, **11**:e1004684

6. Tetzlaff, C, Kolodziejski, C, Timme, M, Tsodyks, M, Wörgötter, F: Synaptic scaling enables dynamically distinct short- and long-term memory formation. *PLoS Comput Biol* 2013, 9, e1003307

7. Litwin-Kumar, A, Doiron, B: Formation and maintenance of neuronal assemblies through synaptic plasticity. *Nat Commun*, 2014, **5**:5319

8. Zenke, F, Agnes, EJ, Gerstner, W: Diverse synaptic plasticity mechanisms orchestrated to form and retrieve memories in spiking neural networks *Nat. Commun* 2015, **6**:6922

9. Brunel, N: Persistent activity and the single-cell frequency-current curve in a cortical network model. *Network* 2000, 11:261–280

## P281 Automatic calibration for hybrid circuits of living and artificial neurons

### Manuel Reyes-Sánchez, Irene Elices, Rodrigo Amaducci, Carlos Muñiz, Francisco B. Rodríguez, Pablo Varona

#### Grupo de Neurocomputación Biológica, Dpto. de Ingeniería Informática, Escuela Politécnica Superior, Universidad Autónoma de Madrid, Madrid, Spain

##### Correspondence: Pablo Varona (pablo.varona@uam.es)

*BMC Neuroscience* 2017, **18** (**Suppl 1**):P281

Hybrid circuits are networks built with living neurons and artificial model (electronic or software) neurons and connections [1]. These circuits allow characterizing neural network dynamics, probing circuit function and have been used to assess the role of individual cells and synapses in specific networks (e.g. see [2–6]).

The temporal scale and amplitude ranges of membrane voltage in living neurons are in general different from the characteristic time scales and amplitudes of the corresponding voltage variables in the models, and thus in most cases signals from/to the living neuron and the model have to be scaled, and/or offset. Model integration time and real time deadlines to implement the hybrid circuit closed-loop are also part of the problem. The process to calibrate both amplitude and time domains is a main impairment in the construction of hybrid circuits. Manual calibration is often difficult and entails a long time and damage risk, something critical due to the limited survival time of the biological preparations and their low resistance to current injection beyond unknown physiological limits. Recording drift is also an issue that has to be addressed in hybrid configurations.

In this work, we present a protocol to perform automatic calibration in hybrid circuits. The protocol is based on achieving a target synchronization level through an artificial electrical connection between the living and the artificial neuron in a regime that guarantees active generation of action potentials. Based on the synchronization criteria, parameters of both temporal scale (model integration time and acquisition/stimulation time constraints) and amplitude scale (voltage and current from/to the living neuron) are set automatically in just a few seconds.

We illustrate our protocol by building a hybrid circuit in the pyloric central pattern generator of *Carcinus Maenas*. The automatic calibration algorithm allows the construction of hybrid circuits in minutes. By reducing calibration time and the risk of damaging the preparation, it is possible to extend the experimental time for the goal given to the hybrid circuit, for instance the exploration of specific dynamical regimes. The automatic search of model parameters in hybrid circuits also allows tuning the best model configuration in the experiment. The proposed algorithm can be easily generalized for any electrophysiological preparation.

**Acknowledgements**

We acknowledge support from MINECO/FEDER DPI2015-65833-P, TIN2014-54580-R (http://www.mineco.gob.es/) and ONRG grant N62909-14-1-N279.

**References**

1. Szucs A, Varona P, Volkovskii AR, Abarbanel HDI, Rabinovich MI, Selverston AI. Interacting Biological and Electronic Neurons Generate Realistic Oscillatory Rhythms. *Neuroreport.* 2000; **11:**563–9.

2. Pinto RD, Varona P, Volkovskii AR, Szücs A, Abarbanel HDI, Rabinovich MI. Synchronous behavior of two coupled electronic neurons. *Phys. Rev. E.* 2000; **62:**2644–56.

3. LeMasson G, Masson SR-L, Debay D, Bal T. Feedback inhibition controls spike transfer in hybrid thalamic circuits. *Nature.* 2002; **417:**854.

4. Nowotny T, Varona P. Dynamic Clamp Technique. *In: Jaeger D, Jung R, editors. Encycl. Comput. Neurosci. Springer New York* 2015. p. 1048–51.

5. Hooper RM, Tikidji-Hamburyan RA, Canavier CC, Prinz AA. Feedback control of variability in the cycle period of a central pattern generator. *J. Neurophysiol.* 2015; **114:**2741–52.

6. Linaro D, Couto J, Giugliano M. Real-time Electrophysiology: Using Closed-loop Protocols to Probe Neuronal Dynamics and Beyond. *J. Vis. Exp.* 2015; **100:**e52320.

## P282 Role of asymmetry in shaping spiking-bursting activity of Central Pattern Generators

### Irene Elices^1^, David Arroyo^1^, Rafael Levi^1,2^, Francisco B. Rodriguez^1^, Pablo Varona^1^

#### ^1^Grupo de Neurocomputación Biológica, Dpto de Ingeniería Informática, Escuela Politécnica Superior, Universidad Autónoma de Madrid, Madrid, Spain; ^2^Department of Biological Sciences, University of Southern California, Los Angeles, CA, USA

##### Correspondence: Irene Elices (irene.elices@uam.es)

*BMC Neuroscience* 2017, **18** (**Suppl 1**):P282

Motor rhythmic patterns in many biological systems are produced by Central Pattern Generators. These networks are typically based on reciprocal inhibitory subcircuits responsible for the production of alternating spiking-bursting activity and the intrinsic dynamics of their constituent cells [1, 2]. In many modelling studies, neurons are considered identical and the reciprocal inhibition, a hallmark of CPG connectivity, is frequently modeled as a symmetric interaction. In this study, we emphasize the importance of asymmetry in the generation and coordination of CPG rhythms from a computational and experimental point of view.

The conductance-based models used in our computational study are inspired by the crustacean pyloric CPG [3, 4]. In particular, the network considered in this work is built up with four Hodgkin-Huxley type neurons and simplified versions of the known connection topologies of the pyloric CPG. The chosen neuron model displays a wide dynamical regime that includes irregular spiking-bursting modes similar to the observed behavior of CPG neurons in isolation. Using these models, we studied the role of asymmetric maximal synaptic conductances, time constants and gap-junction connectivity in the production of regular and irregular bursting activity. Our results show that large regions of both regular and irregular but coordinated rhythms exists as a function of the asymmetry in the circuit. Both asymmetric maximal conductances and inhibitory synaptic time scales contribute to the shaping of wide regimes of regular and irregular triphasic spiking-bursting activity.

Our experimental results of irregular spiking-bursting activity in *Carcinus maenas* indicate the relevant role of asymmetry in producing a triphasic rhythm while maintaining an observed dynamical invariant. Irregularity induced by ethanol [5] revealed the heterogeneity of neuron activity within the CPG circuit, and the resultant irregular pattern could be explained by asymmetry of the synaptic connections. Our recordings of CPG activity at irregular regimes illustrate that the dynamics of neurons and their connections actively bound flexibility to produce a coordinated robust rhythm.

The distinct sources of asymmetry in the model, in particular maximal conductances and two different synaptic time scales, play a key role in producing triphasic rhythms similar to the pyloric’s CPG. Overall, our experimental and modeling results show that the study of asymmetric circuit components and their dynamical interaction help to understand how flexibility and robustness are balanced in central pattern generator circuits.

**Acknowledgements**

We acknowledge support from MINECO/FEDER DPI2015-65833-P and TIN2014-54580-R (http://www.mineco.gob.es/) and ONRG grant N62909-14-1-N279.

**References**

1. Marder E, Calabrese RL: Principles of rhythmic motor pattern generation. *Physiol Rev* 1996, **76**:687–717.

2. Selverston AI, Rabinovich MI, Abarbanel HDI, Elson R, Szücs A, Pinto RD, Huerta R, Varona P: Reliable circuits from irregular neurons: a dynamical approach to understanding central pattern generators. *J Physiol* 2000, **94**:357–374.

3. Elices I, Varona P: Closed-loop control of a minimal central pattern generator network. *Neurocomputing* 2015, **170**:55–62.

4 Elices I, Varona P: Asymmetry Factors Shaping Regular and Irregular Bursting Rhythms in Central Pattern Generators. *Front Comput Neurosci* 2017, **11**:9.

5. Elices I, Arroyo D, Levi R, Rodriguez FB, Varona P: Assessing irregularity and coordination of spiking-bursting rhythms in central pattern generators. *BMC Neuroscience* 2016, 17(Suppl 1):O1

## P283 Rivalry with irregular spiking: resolving mutual inhibition and the balanced state

### Ben Cohen, Carson Chow, Shashaank Vattikuti

#### Lab of Biological Modeling, NIDDK/NIH, Bethesda, MD, 20814, USA

##### Correspondence: Ben Cohen (benjapaulcohen@gmail.com)

*BMC Neuroscience* 2017, **18** (**Suppl 1**):P283

Perceptual rivalry is the subjective experience of alternations between competing percepts when an individual is presented with an ambiguous stimulus. Rivalry has been modeled extensively, and was recently proposed as a canonical neural computation [1]. Mutual inhibition, a network architecture where pools of neurons inhibit one another, has been the cornerstone of models of rivalry [2]. In such a network, inhibition dominates one pool, leading to sparse firing, while the opposing pool fires rigorously under net excitation. This difference in inputs is what drives rivalry, and yet it appears to conflict with balanced state theory, in which net excitation and inhibition approximately balance. Balanced state theory has been used to explain how neurons fire irregularly in response to stimuli, exhibiting Poisson-like inter-spike-interval histograms [3]. Therefore, we investigated rivalry with asynchronous irregular spiking in a ring of leaky integrate-and-fire neurons. We find that rivalry can exist in synchronous or asynchronous, as well as regular or irregular states, and we delineate parameter regimes for each.

**References**

1. Shashaank Vattikuti, Phyllis Thangaraj, Hua W. Xie, Stephen J. Gotts, Alex Martin, Carson C. Chow: Canonical Cortical Circuit Model Explains Rivalry, Intermittent Rivalry, and Rivalry Memory. *PLOS Computational Biology*, 2016, **12(5):**e1004903

2. Jeffery Seely, Carson C. Chow: Role of mutual inhibition in binocular rivalry*. J Neurophysiol*, 2011, **106:**2136 –2150

3. van Vreeswijk C, Sompolinsky H: Chaotic Balanced State in a Model of Cortical Circuits. *Neural Computation* 1998, **10:**1321–1371

## P284 Interplay between inhibition and connectivity structure in driving synchronization and functional properties of neural networks

### Elena Bertolotti^1,2^, Raffaella Burioni^1,2^, Matteo di Volo^3,4,5^, Alessandro Vezzani^1,6^

#### ^1^Department of Mathematical, Physical and Computer Sciences, University of Parma, Parma, Italy, 43124; ^2^INFN, Gruppo Collegato di Parma, Parma, Italy, 43124; ^3^Group for Neural Theory, Departément des Etudes Cognitives, Ecole Normale Supérieure, Paris, France; ^4^Centro Interdipartimentale per lo Studio delle Dinamiche Complesse, Sesto Fiorentino, Italy, 1-50019; ^5^Indiana University–Purdue University, Indianapolis, Indiana 46202, USA; ^6^IMEM-CNR, Parma, Italy, 43124

##### Correspondence: Elena Bertolotti (elena.bertolotti1@fis.unipr.it)

*BMC Neuroscience* 2017, **18** (**Suppl 1**):P284

We theoretically and numerically investigate the interplay between the presence of a fraction of inhibitory neurons in a neural network and their hub character (their relative connectivity with respect of the rest of the network units) in synchronization and input processing of a neural network. The starting point comes from a recent paper by Bonifazi et al. [1], which has put into evidence that hub neurons are typically inhibitory, suggesting a unifying view of cooperation between inhibition and connectivity structure as a driving of synchronization properties in neural networks. In our model, we consider a leaky-integrate-and-fire neural network composed by inhibitory and excitatory neurons with a short term synaptic plasticity mechanism. In order to emphasize the control role of highly connected neurons, both in input and in output direction, we build networks where input and output connectivities are the same for each neuron. Moreover, we apply a heterogeneous mean-field approach to the finite size network dynamics, that lets us speed up numerical computations and highlight the role of neuronal connections distributions. Then we can tune the fraction of inhibitory neurons *fI* and their connectivity level to study the cooperation between hub character and inhibition.

We show how the interplay of these two ingredients gives rise to a wide range of dynamical regimes and different ability to process external inputs. Depending on *f*_*I*_, highly connected inhibitory nodes strongly drive the synchronization properties of the overall network through dynamical transitions from partially synchronous to asynchronous regimes. Furthermore, a metastable regime with long-time memory of external inputs emerges for a specific fraction of hub inhibitory neurons, underlining the role of inhibition and connectivity also for input processing in neural networks.

**Reference**

1. Bonifazi P, Goldin M, Picardo MA, Jorquera I, Cattani A, Bianconi G, Represa A, Ben-Ari Y, Cossart R: GABAergic hub neurons orchestrate synchrony in developing hippocampal networks. *Science* 2009, **326:**1419–1424

## P285 A mechanistic model of dopamine modulated learning in the olfactory system of Drosophila

### Bayar Menzat, Tim P. Vogels

#### Centre for Neural Circuits and Behaviour, University of Oxford, Oxford, UK

##### Correspondence: Bayar Menzat (bayar.menzat@cncb.ox.ac.uk)

*BMC Neuroscience* 2017, **18** (**Suppl 1**):P285

The fruit fly memorises previous experiences of different odours along with information on whether the odour was associated with reward or punishment in an area called the mushroom body. The pairing of an odour with reward or shock process leads to odour specific modification in the responses of motor biasing output neurons (MBONs). MBONs receive excitatory input from odour identity coding Kenyon Cells (KCs). The connection between KCs and MBONs is targeted by dopaminergic neurons (DAs) [1]. The KC-MBON synapses change their efficacies when learning occurs, with experimental evidence proposing that the Spike-Timing-Dependent Plasticity synaptic change rule is present [2]. STDP changes the strength of a connection between two neurons depending on the precise timing of their action potentials [3]. When an odour is presented alone the firing rate of MBONs has been shown to increase, while paired odour and reinforcement presentation has shown to decrease the activity of the same MBONs [4, 5]. Here, we introduce a reward modulated STDP learning rule, where the learning rate of STDP is controlled by the firing rate of dopaminergic neurons. To test this learning rule, we simulate simultaneous presentation of odour and reward in a spiking model of the olfactory circuit. We find that our learning rule can create a stable memory of the value (positive, negative or neutral) of an odour in the excitatory weights of the KC-MBON synapses. Our model can reproduce experimentally observed bi-directional changes in the firing rates of MBONs after learning. Finally, we show that when we combined our learning rule with excitatory feedback from MBONs to DA neurons, dopamine modulated learning plasticity can provide a mechanism for learning the uncertainty of a reward. Building on this result, we make experimental predictions of which odour will be selected between two odours associated with uncertain reward. By proposing a simple mechanistic model of dopamine mediated learning our work has improved the understanding of the role of dopamine in the fruit fly olfactory learning.

**References**

1. Scott Waddell, Neural Plasticity: Dopamine Tunes the Mushroom Body Output Network, *Current Biology* 2016, **26**, no. 3, R109–12, doi:10.1016/j.cub.2015.12.023.

2. Stijn Cassenaer and Gilles Laurent: Conditional Modulation of Spike-Timing-Dependent Plasticity for Olfactory Learning. *Nature* 2012, **482**, no. 7383: 47–52, doi:10.1038/nature10776.

3. Guo-qiang Bi and Mu-ming Poo: Synaptic Modifications in Cultured Hippocampal Neurons: Dependence on Spike Timing, Synaptic Strength, and Postsynaptic Cell Type. *Journal of Neuroscience* 1998, **18**, no. 24: 10464–72, doi:10.1038/376074a0.

4. David Owald et al.: Activity of Defined Mushroom Body Output Neurons Underlies Learned Olfactory Behavior in Drosophila, *Neuron* 2015, **86**, no. 2: 417–27, doi:10.1016/j.neuron.2015.03.025.

5. Toshihide Hige et al.: Heterosynaptic Plasticity Underlies Aversive Olfactory Learning in Drosophila. *Neuron* 2015, **88**, no. 5: 985–98, doi:10.1016/j.neuron.2015.11.003.

## P286 Saliency-based Gaze Prediction based on the Neural Population for Integrating the Direction of Figure

### Nobuhiko Wagatsuma

#### School of Science and Engineering, Tokyo Denki University, Hiki, Saitama 350-0394, Japan

##### Correspondence: Nobuhiko Wagatsuma (nwagatsuma@rd.dendai.ac.jp)

*BMC Neuroscience* 2017, **18** (**Suppl 1**):P286

Selective attention is a function of the brain that allocates its computational resource to the momentarily most important subsets of a visual scene. Saliency models were used to predict the locations of selective attention and gaze [1]. I propose the biologically plausible saliency model based on the neural population for integrating activities in intermediate-level visual areas with neurons selective to the direction of figure (DOF). Russell et al. demonstrated that the DOF integration played an important role for computing saliency [2]. In addition, computational study hypothesized that a vast variety of surrounding organizations by connections from early- to intermediate-level visual areas were a basis for the neural selectivity of the DOF [3]. I extended the previous saliency model [2] by introducing a variety of spatial patterns of synaptic connectivity for integrating the neural responses to the DOF. In this work, a population of model neurons underlay the determination of saliency magnitude. I tested hundreds of DOF organizations, and found that my proposed saliency model not only reproduced the characteristics of perceptual organization but also captured object locations in natural images (Figure 1A). Furthermore, the gaze prediction accuracy shown by my saliency mechanism was significantly higher than that by previous models [1, 2] (Figure 1B). These results suggested a crucial role of various synaptic patterns in DOF integration and a neural population coding of saliency to determine selective attention and predict the locations of gaze.
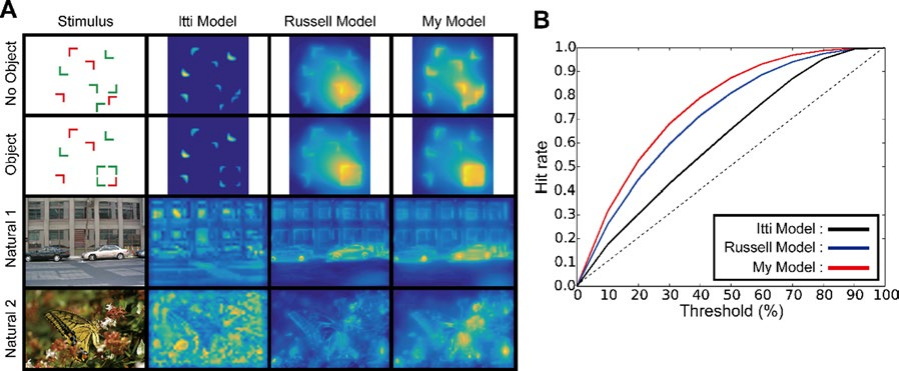


**Figure 1**. Simulation results. **A**. Examples of images and saliency maps calculated using previous models [1, 2] and my proposed model. **B.** Results of gaze estimation. I drew receive operating characteristics (ROC) curves with MIT data set (1003 images and fixation data) [4] for quantifying the responses of saliency models

**Acknowledgements**

This work was partly supported by KAKENHI (no.26880019) and Technology of Tokyo Denki University Grant Number Q16 J-04.

**References**

1. Itti L, Koch C, Niebur E: A model of saliency-based visual attention for rapid scene analysis. *IEEE Trans Pattern Analy Mach Intell* 1998, **20:**1254–1259.

2. Russell AF, Mihalas S, von der Heydt R, Niebur E, Etienne-Cummings R: A model of proto-object based saliency. *Vision Res* 2014, **94:**1–15.

3. Sakai K, Nishimura H: Surrounding suppression and facilitation in the dtermination of border ownership. *J Cogn Neurosci* 2006, **18:** 562–579.

4. Judd T, Ehinger K, Durand F, Torralba A: Learning to predict where human look. *IEEE International Conference on Computer Vision* 2009, 12: 2106–2113.

## P287 Investigating the differences in ion channel properties between ON and OFF ganglion cells: a combination of modelling and optimization approach

### Susmita Saha^1^, Reena Kapoor^1^, Robert Kerr^2^, John Wagner^1^

#### ^1^IBM Research Australia, VIC 3006, Melbourne, Australia; ^2^NeuroEngineering Laboratory, Electrical & Electronic Engineering, The University of Melbourne, Melbourne, Australia

##### Correspondence: Susmita Saha (susmitas@au1.ibm.com)

*BMC Neuroscience* 2017, **18** (**Suppl 1**):P287

Experimental studies have demonstrated differences in the intrinsic physiological responses between ON and OFF retinal ganglion cells (RGCs). OFF cells exhibit intrinsic spontaneous activity, subthreshold membrane potential oscillations, rebound excitation and burst firing. ON cells display none of the aforementioned intrinsic phenomena. Previous modeling studies [1, 2] showed how special properties of low-voltage-activated (T-type) calcium currents can explain the physiological differences between ON and OFF RGCs, while assuming that most of the other ion channel properties are similar. In our study, using a combination of computer simulations of single compartment, Hodgkin-Huxley type neurons and Bayesian optimisation, we optimised the leak reversal potential and all literature-reported ion channel conductance densities against experimental findings from mouse ON and OFF RGCs to estimate the potential contributions of other ion channels. Optimising the larger set of conductances suggested two distinct sets of parameters for ON and OFF cells (Table 1). In agreement with previous findings [1, 2], the low-voltage-activated calcium conductance (g_lva_) is indeed higher in OFF than ON cells, but our results (Figure 1) suggested further that g_lva_ is the main contributor to the differences between ON and OFF cells. In addition, we found that the voltage-gated sodium channel conductance may be very different in ON and OFF cells, as also suggested in [2]. In addition, our analysis (not shown here) also suggests that mainly the fast inactivating sodium current, not the persistent sodium current, play a significant role in generating distinct properties in ON and OFF GCs. Overall, our single neuron model with optimised ion channel parameters was able to demonstrate a major dependence of ON and OFF cell specific intrinsic activity on the sodium and calcium currents.

**Table:** **Table 1**. Optimized intrinsic parameters in ON and OFF GCs (% gap = 100*(((OFF value-ON value)/ON value))

Parameter	ON	OFF	% gap
Leak reversal potential (mV), V_L_	−60.4	−58.1	4
Leak (S/cm^2^), $\bar{g}_{L}$	4E−05	3E−05	20
A-type potassium (S/cm^2^), $\bar{g}_{K,A}$	0.035	0.036	5
Low-voltage-activated calcium (S/cm^2^), $\bar{g}_{lva}$	1E−04	8E−04	523
Persistent sodium (S/cm^2^),$\bar{g}_{Nap}$	1E−07	3E−08	73
Calcium (S/cm^2^), $\bar{g}_{Ca}$	0.008	0.009	11
Hyperpolarization-activated (S/cm^2^), $\bar{g}_{h}$	4E−06	3E−06	23
Potassium (S/cm^2^), $\bar{g}_{k}$	0.049	0.059	20
Ca-activated potassium (S/cm^2^), $\bar{g}_{k(Ca)}$	7E−05	9E−05	39
Fast inactivating sodium (S/cm^2^), $\bar{g}_{Na}$	0.025	0.077	207


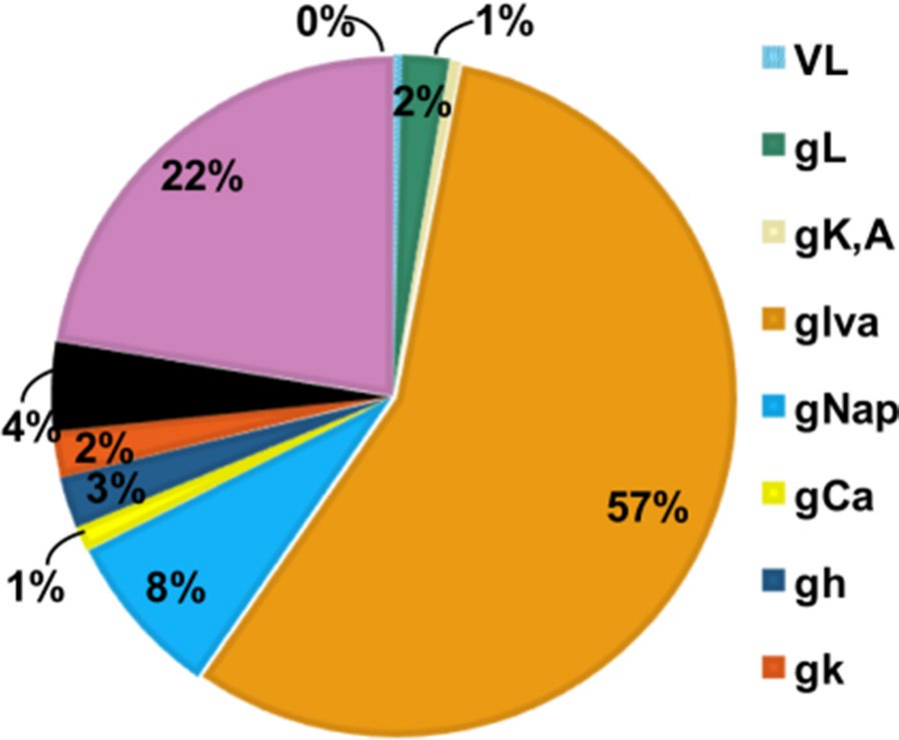


**Figure 1.** Relative  % gap (round((%gap/total_gap) *100) between two optimised parameter sets in ON and OFF RGC

**Conclusion:** The optimised cell-specific ion channel parameters imply that $\bar{g}_{lva}$ and $\bar{g}_{Na}$ exhibited the most important role in explaining intrinsic behavioural differences in ON and OFF retinal ganglion cells.

**References**

1. Kameneva T, Meffin H, Burkitt AN: Modelling intrinsic electrophysiological properties of ON and OFF retinal ganglion cells. *J. Comput. Neurosci* 2011; **31:**547–61.

2. Guo T, Tsai D, Morley JW, Suaning GJ, Kameneva T, Lovell NH, et al.: Electrical activity of ON and OFF retinal ganglion cells: a modelling study. *J. Neural Eng* 2016; **13:**25005.

## P288 Response reversal during top-down modulation in cortical circuits with multiple interneuron types

### Luis C. Garcia del Molino^1^, Guangyu Robert Yang^1^, Jorge F. Mejias^1^, and Xiao-Jing Wang^1,2^

#### ^1^Center for Neural Science, New York University, New York, NY 10003, USA; ^2^NYU-ECNU Institute of Brain and Cognitive Science, NYU Shanghai, Shanghai, 200122, China

##### Correspondence: Luis C. Garcia del Molino (garciadelmolino@nyu.edu)

*BMC Neuroscience* 2017, **18** (**Suppl 1**):P288

Three major non-overlapping classes of interneurons expressing parvalbumin, somatostatin, or vasoactive intestinal peptide (henceforth denoted PV, SST and VIP respectively) show cell type specific connectivity within themselves and with excitatory neurons leading to a canonical microcircuit across cortex [1, 2]. Dissecting the dynamics of this microcircuit is essential to our understanding of the mammalian cortex. However, experiments recording from this circuit often report counterintuitive and seemingly contradictory findings.

One particular example of complex behavior is the modulation of responses to visual stimuli during locomotion, when V1 activity significantly increases with respect to immobility [3]. VIP interneurons are known to be involved in such modulation because artificially activating (damaging) them mimics (blocks) the effect of running on visual response [4]. Since VIP cells inhibit SST cells which in turn inhibit excitatory, PV and VIP cells, a natural explanation for this phenomenon is disinhibition [5]: upon activation of VIP cells the SST population is inhibited and therefore neurons targeted by the SST population are disinhibited, raising the overall rate of the excitatory neurons. However recent experiments show that the network behavior might be more complex. In particular, in the absence of visual stimulation, the activation of VIP cells results in an average decrease of SST population activity [4, 6] whereas in the presence of visual stimulation the response of SST cells is reversed and its rate increases during locomotion [6, 7] which appears to challenge the disinhibition hypothesis. This observation suggests that the nature of the interaction between VIP and SST could be stimulus dependent.

We developed a general theoretical framework to explain such response reversal, and we showed how these complex dynamics can emerge in circuits that possess two key features: the presence of multiple interneuron populations and a non-linear dependence between the input and output of the populations. Furthermore, we built a cortical circuit model and the comparison of our simulations with real data shows that our model reproduces the complex dynamics observed experimentally in mouse V1. Our explicit calculations allowed us to pinpoint the connections critical to response reversal, and to predict the existence of more types of complex dynamics that could be experimentally tested.

**References**

1. Jiang X, Shen S, Cadwell CR, Berens P, Sinz F, Ecker AS, Patel S, Tolias AS: Principles of connectivity among morphologically defined cell types in adult neocortex, *Science* 2015, **350(6264):**p.aac9462.

2. Pfeffer CK, Xue M, He M, Huang ZJ, Scanziani M: Inhibition of inhibition in visual cortex: the logic of connections between molecularly distinct interneurons. *Nature Neuroscience* 2013, **16(8):**1068–1076.

3. Niell CM, Stryker MP: Modulation of Visual Responses by Behavioral State in Mouse Visual Cortex. *Neuron* 2010, **65(4):**472–479.

4. Fu Y, Tucciarone JM, Espinosa JS, Sheng N, Darcy DP, Nicoll RA, Huang ZJ, Stryker MP: A Cortical Circuit for Gain Control by Behavioral State. *Cell* 2014, **156(6):**1139–1152.

5. Wang XJ, Tegnér J, Constantinidis C, Goldman-Rakic PS: Division of labor among distinct subtypes of inhibitory neurons in a cortical microcircuit of working memory. *PNAS* 2004, **101(5):**1368–1373.

6. Dipoppa M, Ranson A, Krumin M, Pachitariu M, Carandini M, Harris KD: Vision and locomotion shape the interactions between neuron types in mouse visual cortex. *bioRxiv* 2016, p.058396

7. Pakan JM, Lowe SC, Dylda E, Keemink SW, Currie SP, Coutts CA, Rochefort NL: Behavioral-state modulation of inhibition is context-dependent and cell type specific in mouse visual cortex. *eLife.* 2016, 23; **5:**e14985.

## P289 Cellular and Network Modeling of the Striatal Microcircuit in a Mouse Model of Huntington’s Disease

### Hanbing Song^1^, Joseph Goodliffe^2^, Jennifer Luebke^2^, Christina M. Weaver^1^

#### ^1^Department of Mathematics, Franklin and Marshall College, Lancaster, PA, 17604, USA; ^2^Department of Anatomy and Neurobiology, Boston University School of Medicine, Boston, MA, 02118, USA

##### Correspondence: Hanbing Song (hsong1@fandm.edu)

*BMC Neuroscience* 2017, **18** (**Suppl 1**):P289

Huntington’s disease (HD) is a neurodegenerative disorder of the central nervous system characterized by movement, cognitive, and psychiatric disturbance. In HD, massive structural and functional neuropathology occurs in the striatum, particularly to spiny projection neurons (SPNs). SPNs are part of either direct or indirect pathways of the basal ganglia, and labeled either dSPNs or iSPNs respectively. The mechanisms underlying the vulnerability of SPNs in HD are unclear, and dSPNs and iSPNs are functionally distinct and differentially affected in HD [1]. A commonly employed transgenic mouse model of HD, Q175, exhibits changes of molecular phenotypes, specific neuronal dysfunction, and subtle but significant movement disorders [2]. Recent in vitro experiments on 12-month old animals showed that, compared to wildtype (WT), dSPNs in Q175 animals showed increased input resistance, reduced rheobase and reduced amplitude in action potentials. In addition, both dSPNs and iSPNs exhibited greater dendritic complexity and lower spine density, along with altered frequencies of spontaneous post-synaptic currents (reduced excitatory and increased inhibitory). This project models the SPNs of the striatum to further our understanding of these observed changes in Q175 vs. WT mice. First, we used morphoelectrotonic transforms of reconstructed SPNs to predict that dendritic signal attenuation is greater in SPNs from Q175 animals. Then, we used our parameter optimization method [3], implemented in NEURON (https://www.neuron.yale.edu/neuron/) on a published 189-compartment conductance-based model SPN [4], to acquire a set of parameters depicting the passive membrane properties and active channel gating (conductance and kinetics) of SPNs so that the difference of model output and empirical recording experimental data could be minimized. We found proper fits to the Q175 data only after increasing the branching complexity in the published morphology. Differences in reversal potential of the leak channel and inward-rectifying potassium channel (KIR) contributed to the increased excitability in Q175 dSPNs, consistent with empirical observations in mouse models of HD. Finally, we constructed a microcircuit network model of both dSPNs and iSPNs and fast spiking interneurons (FSIs) [5] that reflects the empirical findings and incorporates the optimized parameters in our single neuron study. In the WT network, dSPNs and iSPNs fired in nearly equal proportion in response to input from FSIs and the cortex and thalamus, predicting a balanced condition for motor movements. Perturbing our model network consistent with the Q175 experiments (e.g., altering the frequency of synaptic inputs received by SPNs) resulted in an imbalanced firing pattern among dSPNs and iSPNs, consistent with what is thought to occur in HD pathology. These models provide a novel way to explore how individual neuron and network properties contribute to functional pathology of the striatal microcircuit in Q175 mice, in order to better understand the deleterious effects of mutant Huntingtin in the human brain.

**Acknowledgements**

This research was supported by the CHDI Foundation, and used the Neuroscience Gateway and the Extreme Science and Engineering Discovery Environment (XSEDE), which is supported by National Science Foundation grant number ACI-1053575.

**References**

1. Galvan L, André VM, Wang EA, Cepeda C, Levine MS: Functional differences between direct and indirect striatal output pathways in Huntington’s disease. *J Huntingtons Dis* 2012, **1:**17

2. Rangel-Barajas C and Rebec GV: Dysregulation of corticostriatal connectivity in Huntington’s disease: a role for dopamine modulation. *J Huntingtons Dis* 2016, **5**:303.

3. Rumbell TH, Draguljić D, Yadav A, Hof PR, Luebke JI, Weaver CM: Automated evolutionary optimization of ion channel conductances and kinetics in models of young and aged rhesus monkey pyramidal neurons. *J Comput Neurosci* 2016, **41**:65

4. Evans RC, Morera-Herreras T, Cui Y, Du K, Sheehan T, Kotaleski JH, Venance L, Blackwell KT: The effects of NMDA subunit composition on calcium influx and spike timing-dependent plasticity in striatal medium spiny neurons. *PLoS Comput Biol* 2012, **8**:e1002493.

5. Damodaran S, Evans RC, Blackwell KT: Synchronicity of fast-spiking interneurons balances medium-spiny neurons. *J Neurophysiol* 2014, **111**:836.

## P290 Convolutional Neural Network-based Interictal Epileptiform Discharge Detection

### John Thomas^1^, Nishant Sinha^2,3^, Nikhita Shaju^1,4^, Tomasz Maszczyk^1^, Jing Jin^1^, Sydney S. Cash^5^, Justin Dauwels^1†^, M. Brandon Westover^5†^

#### ^1^School of Electrical and Electronic Engineering, Nanyang Technological University, Singapore 639798, Singapore; ^2^Institute of Neuroscience, Faculty of Medical Sciences, Newcastle University, Newcastle upon Tyne, UK; ^3^School of Computing Science, Newcastle University, Newcastle upon Tyne, UK; ^4^Department of Electrical and Electronics Engineering, Vellore Institute of Technology, Vellore, Tamil Nadu, India; ^5^Neurology Department, Massachusetts General Hospital and Harvard Medical School, Boston, MA, USA

##### Correspondence: Justin Dauwels (jdauwels@ntu.edu.sg)

^†^Contributed equally as co-senior authors

*BMC Neuroscience* 2017, **18** (**Suppl 1**):P290

Interictal epileptiform discharges (IEDs) are identified as one of the distinctive biomarkers of epilepsy. The clinical gold standard for IED detection is visual inspection by trained clinical neurophysiologists (CNs). Therefore, the diagnosis becomes a heavily expert-centered process. Automated or semi-automated IED detection systems could overcome this current problem. Convolutional neural networks (CNNs) are multilayer feed-forward deep neural networks that are widely applied for classification and prediction. In this study, we develop an efficient CNN-based IED detector and compare the performance with the traditional support vector machine (SVM)-based IED detector. A similar study has been performed in [1], but here we study the problem in greater depth and on a much larger dataset. We analyze 30-minute EEG recordings of 93 patients with epilepsy. The data was recorded according to the standard 10-20 electrode placement system at Massachusetts General Hospital (MGH), Boston. IEDs were independently annotated by two CNs. Each IED was extracted as a 500-millisecond waveform. A total of 18,164 IEDs were extracted. The CNN was developed using Tensorflow-r0.12 [2] with a Tesla K40 GPU. We developed the CNN model with five layers: an input layer, convolutional layer, pooling layer, fully connected layer, and output layer. Four convolutional filters, each of size (1 × 4), were applied in the convolutional layer. The Rectified linear unit (ReLU) activation function was applied with 100 neurons in the hidden layer. Training was performed until 99.99% training accuracy was obtained. To prevent over-fitting, weights to the output layer were dropped with a probability of 50% in each training epoch. 5-fold cross-validation results are presented in Table 1. For diagnostic purposes, CNs are most concerned with identifying the presence vs absence of IEDs in any given EEG recording, as opposed to detecting all instances of IEDs. False positives pose a major challenge, as there are typically many more background waveforms than IEDs (1000:1 imbalance). The CNN provides high sensitivity at very low false positive rates (see Figure 1), and thus is substantially less prone to false positives compared to the SVM.

**Table:** **Table 1**. Cross-validation results for CNN and SVM-based IED detector systems

Performance indices	CNN	SVM
Sensitivity	99.09%	32.18%
Specificity	93.22%	98.95%
BAC	96.15%	65.57%
AUC	0.966	0.829


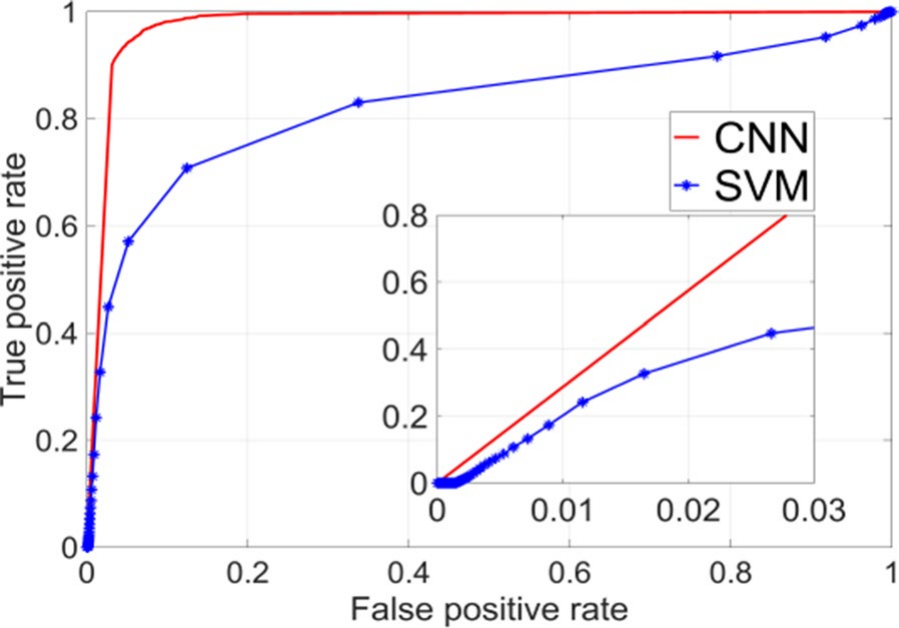


**Figure 1.** The ROC plots of CNN and SVM-based IED detector systems

**Conclusions:** The CNN-based IED detector outshines the traditional SVM-based system and methods proposed in the literature in terms of sensitivity and specificity. Moreover, this study considers a much larger data set than similar studies in the literature.

**References**

1. A. R. Johansen, J. Jin, T. Maszczyk, J. Dauwels, S. S. Cash, and M. B. Westover: Epileptiform Spike Detection via Convolutional Neural Networks, *Proc. IEEE ICASSP* 2016, pp. 754–758.

2. M. Abadi, A. Agarwal, P. Barham, E. Brevdo, Z. Chen, C. Citro, G. S. Corrado, A. Davis, J. Dean, M. Devin et al.: TensorFlow: Large-Scale Machine Learning on Heterogeneous Distributed Systems, 2015. Software available from tensorflow.org.

## P291 Effects of Hebbian learning on networks of Kuramoto phase oscillators with time delay

### Maryam Karimian^1^, Domenica Dibenedetto^1^, Michelle Moerel^1,2^, Peter De Weerd^1,2^, Thomas Burwick^4^, Ronald L. Westra^1,3^

#### ^1^Maastricht Centre for Systems Biology, Maastricht University, 6229ER, Maastricht, The Netherlands; ^2^Department of Cognitive Neuroscience, Maastricht University, 6229ER, Maastricht, The Netherlands; ^3^Department of Data Science and Knowledge Engineering, Maastricht University, 6211LH, Maastricht, The Netherlands; ^4^Frankfurt Institute for Advanced Studies, Goethe University Frankfurt, 60438 Frankfurt am Main, Germany

##### Correspondence: Maryam Karimian (maryam.karimian@maastrichtuniversity.nl)

*BMC Neuroscience* 2017, **18** (**Suppl 1**):P291

Neuronal oscillations are crucial for various cognitive functions, including learning. Among neuronal populations, patterns of synchronization can drive connectivity changes, in turn modifying oscillations and synchronization. To study changes in oscillation patterns with learning, we modeled brain processing using a directed random network of phase-coupled oscillators interacting according to the Kuramoto model [1]. We incorporated two extensions into the Kuramoto model: spatial embedding through coupling delays, and synaptic plasticity according to a Hebbian learning formulation containing learning associated parameters [2], i.e. learning rate (LR), which determines the speed of learning, and learning enhancement (LE), which limits the range of coupling weights. We investigated the structural and functional changes in the network with learning using graph theory and synchronization evaluating tools, respectively. To study the structural changes, we calculated the small-worldliness (SW) [3] of the network throughout the simulated time. Our preliminary results show that with learning the network is reweighted into a new structure with relatively high levels of SW (Fig. 1A), but a fully connected pattern. To study the functional changes, we measured the degree of synchronization for each combination of learning parameters. We observed that specific combinations of learning parameters led the network to show features of either single cluster synchronization, or split oscillators into two anti-phase synchronized clusters. These two synchronization features are evaluated by measuring global order parameters R_1_ and R_2_ [2], respectively. The introduction of time delay affected the network dynamics and structure changes especially in early stages of learning (Fig. 1A, C), but the global behavior observed (Fig. 1B, C) doesn’t change. In summary, this enhanced Kuramoto model seems promising as this induces a SW network topology. However, as the network obtained after learning is almost fully connected (unlike the efficiency typical for brain networks), crucial next steps include the exploration of biologically plausible ways to prune the network in order to increase the wiring cost efficiency, prior to the application of the model to neuroscientific data.
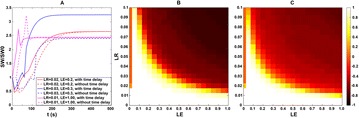


**Figure 1. A.** Small-worldliness changes over time for three combinations of (LE, LR) in different states of synchronization. Differences (R_1_ - R_2_) averaged over the last 100 time steps (0.1 s) of simulation time (500 s) for cases of having (**B**) and not having (**C**) time delay

**References**

1. Juan A. Acebrón, L. L. Bonilla, Conrad J. Pérez Vicente, Félix Ritort, and Renato Spigler: The Kuramoto model: A simple paradigm for synchronization phenomena. *Rev. Mod. Phys* 2005. **77**.

2. Niyogi, R. K. & English, L. Q.: Learning-rate-dependent clustering and self-development in a network of coupled phase oscillators. *Phys. Rev. E* 2009, **80**.

3. Bassett, D. S. & Bullmore, E. D.: Small-World Brain Networks. *Neuroscientist* 2006, **12:** 512–523.

## P292 Biophysical modelling of resting brain states and inhibitory synaptic plasticity

### Romesh Abeysuriya^1^, Jonathan Hadida^1,2^, Stamatios Sotiropoulos^2^, Saad Jbabdi^2^, Mark Woolrich^1,2^

#### ^1^Oxford Centre for Human Brain Activity, Oxford, United Kingdom; ^2^Oxford Centre for Functional MRI of the Brain, Oxford, United Kingdom

##### Correspondence: Romesh Abeysuriya (romesh.abeysuriya@psych.ox.ac.uk)

*BMC Neuroscience* 2017, **18** (**Suppl 1**):P292

Achieving realistic neural dynamics in biophysical models typically requires extremely fine tuning of parameters, whereas in the real brain dynamics are robust even to significant changes including sleep and wake. One possible explanation is that robust dynamics are facilitated by homeostatic mechanisms that are able to dynamically rebalance brain networks. In this study, we use one such mechanism, inhibitory synaptic plasticity (ISP), to achieve a local balance between excitation and inhibition, and investigate the effect this has on resting brain states. We simulated neural activity in 68 cortical brain regions using the relatively simple Wilson-Cowan neural mass model. Each brain region consists of an excitatory and an inhibitory population of neurons. Long-range white matter connections link excitatory populations with distance-dependent propagation delays, while inhibitory connections are purely local. Anatomical connectivity weights were estimated using pre-processed diffusion MRI data from the Human Connectome Project [1] using probabilistic tractography (40 subjects). ISP was incorporated in each brain region as a dynamic change in the local inhibitory connection depending on the difference between excitatory activity and a preselected target level of activity [2, 3]. For comparison to experimental data, we used resting state MEG recordings from 55 healthy controls. Source-space parcel timecourses were computed for the same brain regions as the model. Compared to previous work using coupled Kuramoto phase oscillators [4], we find the Wilson-Cowan model is even more sensitive to the network coupling strength because the amplitude of oscillations in neural activity can vary. ISP successfully adjusts local inhibition to balance excitatory activity across the network (Fig. 1A), reducing this sensitivity. As a result, at intermediate delays the network exhibits metastable dynamics and amplitude envelope functional connectivity that is well correlated with experimental data over a wide range of global coupling strengths (Fig. 1B-1D). Simple neural mass models are largely unable to predict frequency-specific connectivity, and we have focused primarily on alpha connectivity here. Future work will investigate simultaneous prediction of the different patterns of connectivity seen in experimental data across frequency bands.
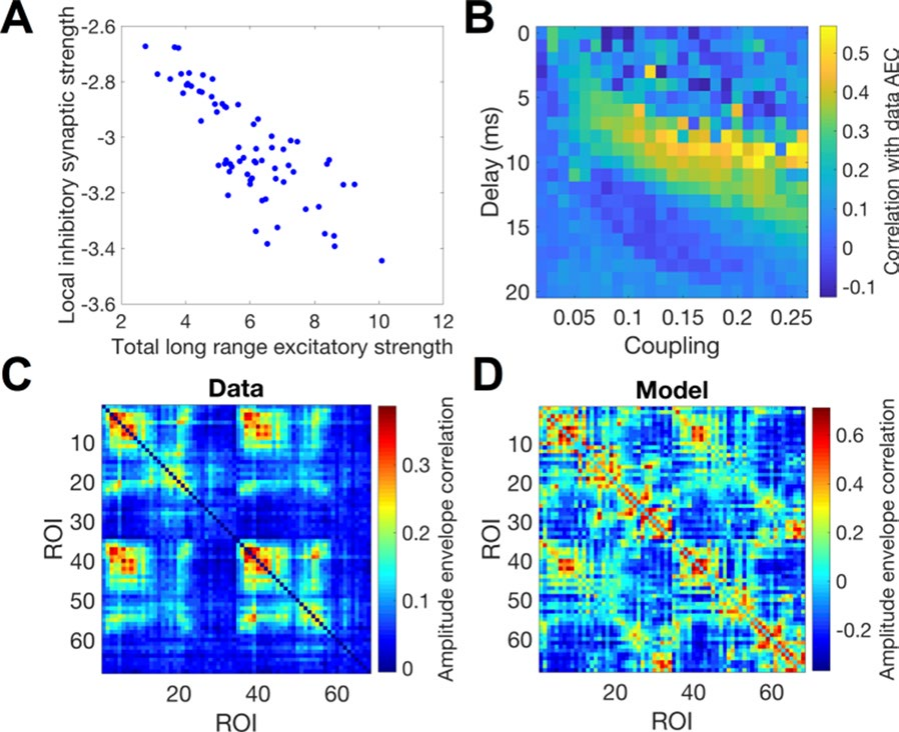


**Figure 1. A.** Local inhibition balances long-range excitation. **B.** Correlation between data and model alpha band functional connectivity (amplitude envelope correlation). Alpha band functional connectivity profiles **C.** in MEG data **D.** in the model

**References**

1. Sotiropoulos SN, Jbabdi S, Xu J, Andersson JL, Moeller S, Auerbach EJ, et al. Advances in diffusion MRI acquisition and processing in the Human Connectome Project. *Neuroimage* 2013, 80:125–143.

2. Vogels TP, Froemke RC, Doyon N, Gilson M, Haas JS, Liu R, et al. Inhibitory synaptic plasticity: spike timing-dependence and putative network function. *Front. Neural Circuits* 2013, 7:119.

3. Hellyer PJ, Jachs B, Clopath C, Leech R. Local inhibitory plasticity tunes macroscopic brain dynamics and allows the emergence of functional brain networks. *Neuroimage* 2016, 124:85–95.

4. Cabral J, Luckhoo H, Woolrich M, Joensson M, Mohseni H, Baker A, et al. Exploring mechanisms of spontaneous functional connectivity in MEG: How delayed network interactions lead to structured amplitude envelopes of band-pass filtered oscillations. *Neuroimage* 2014, 90:423–435.

## P293 Temporal pattern recognition and control of animat foraging by evolving small networks of adaptive exponential integrate-and-fire neurons

### Chama Bensmail^1,2^, Volker Steuber^1^, Borys Wrobel^2,3^

#### ^1^Centre for Computer Science and Informatics Research, University of Hertfordshire, Hatfield, AL10 9AB, UK; ^2^Biology, Adam Mickiewicz University, 61-712 Poznan, Poland; ^3^Systems Modelling IOPAN, 81-701 Sopot, Poland

##### Correspondence: Chama Bensmail (chamabens@evosys.org)

*BMC Neuroscience* 2017, **18** (**Suppl 1**):P293

Temporal pattern recognition is a common computational task that can be performed by neural networks. The networks investigated in this work are evolved with a biologically inspired artificial life platform [1], and consist of up to five adaptive exponential integrate-and-fire neurons with parameters producing tonic spiking with constant input current [2]. The animat forages in a 2D open world where it receives signals—we will describe them as 3-letter words, but the letters can be also seen as flashes of light (with 3 different colors) or musical notes (with 3 different frequencies). These words are emitted from two sources: a target, which emits the word **ABC**, and a distractor, which emits all the other 26 words consisting of a, b and c. The words and letters never overlap (see Figure 1). Each word lasts for 25-ms, with 2-ms intervals of silence between letters, and 20-ms intervals between words. When the animat touches an object (initially placed randomly), the object disappears, and another object of the same type reappears in another random position. The animat is equipped with 6 sensors; 2 per letter, which provide the input to the network (one input for the difference of signal intensity on two sides of the animat, and the other input for the average of signal intensity). The animat has 2 actuators, whose activity is driven by the number of spikes produced by the output neurons during the previous 120-ms. When the activity of one actuator (say, left) is higher, the animat turns (here, right); when both activities are equal, the animat moves straight, when both activities equal 0, the animat stops.

We used a genetic algorithm to obtain several small networks which discriminate efficiently the target pattern from all the other 3-letter words. Our results show that evolving in the presence of small amounts of noise on the duration of the letters results in a more efficient discriminator than evolution without such noise. The noisy pattern had letters with 5-, 7-, and 9-ms duration, with the duration ordered randomly, the pattern without noise had all letters with 7-ms duration. Only the networks evolved with a noisy pattern were robust to even noisier patterns (for example, they recognized patterns in which letters had 1-, 7-, and 13-ms duration, with random order of duration). Both the networks evolved with and without noise on the length of the letters were robust also to the change in the actuator forces and on silence intervals between words (up to 200-ms); number of objects in the world (up to 2 objects of each type).
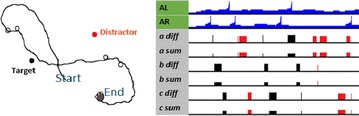


**Figure 1.** The champion environment and its highly noisy input vector (from left to right: **ABC**, **aac**, **ABC**, **ABC**, **aba**, **cab**). When a target is hit; it becomes a black circle. The green boxes are the actuator activities and the height of the bars corresponds to the sensory activity

**Acknowledgements**

This work was supported by the Polish National Science Center (project EvoSN, UMO-2013/08/M/ST6/00922).

**References**

1. Wróbel, B. Evolution of spiking neural networks robust to noise and damage for control of simple animats. *In:* *Proceedings of the 14th International Conference on Parallel Problem Solving from Nature* 2016.

2. Naud, Richard, et al. Firing patterns in the adaptive exponential integrate-and-fire model. *Biological cybernetics* 2008; **19.4-5**:335.

## P294 Learning Continuous Attractor Neural Networks from Continuously Morphed Patterns

### Xiaolong Zhou^1,2,†^, Zilong Ji^2,†^, Xiao Liu^2^, Yan Xia^2^, and Si Wu^2^

#### ^1^School of Systems Science, Beijing Normal University, Beijing 100875, China; ^2^State Key Laboratory of Cognitive Neuroscience & Learning, IDG/McGovern Institute for Brain Research, Beijing Normal University, Beijing 100875, China

##### Correspondence: Si Wu (wusi@bnu.edu.cn)

^†^Equal contribution

*BMC Neuroscience* 2017, **18** (**Suppl 1**):P294

Continuous attractor neural networks (CANNs) have been widely used as a canonical model for neural information representation [1]. They have been successfully applied to describe the encoding of a number of continuous features in neural systems, such as orientation, moving direction, head direction, and spatial location of objects [1]. It remains unclear, however, how a neural system acquires such a network structure in practice. Compared to the Hopfield network, the key property of a CANN is that it holds a continuous family of stationary states, which form an (*approximately*) flat subspace in the network states, rather than being isolated with each other with high-energy barriers. Hopfield network is learned by Hebbian learning from statistically independent memory patterns. It has been suggested that a CANN may be learned by Hebbian learning from correlated patterns, and in the ideal situation, from continuously morphed patterns. The challenge for memorizing correlated patterns is that the classical Hebb rule merges correlated patterns into a single attractor, corresponding to the pattern having the maximum overlap with others. To overcome this difficulty, two methods which modifies the Hebb rule were proposed. One considers the “popularity” of a neuron, i.e., the involvement of a neuron in all memory patterns [2]. If a neuron is very popular, then its contribution in Hebbian learning is decreased accordingly. By this, the network can store some correlated patterns, but requires that within a pattern, neuronal activities are statistically independent, a condition hardly satisfied in reality. The other approach considers the “novelty” of a newly presented memory pattern, measured by the Hamming distance between the new pattern and those already stored in the network [3]. If a new pattern is novel, then the pattern is learned by the Hebb rule; otherwise, the learning effect is diminished accordingly. This method works well in certain cases, but still has the shortcoming of that the learned result is rather sensitive to the presenting order of patterns. In this study, we propose a new method to learn a CANN from correlated patterns. The method applies the Hebb rule only after correlated patterns are orthogonalized by the Gram-Schmidt rule [4]. In effect, this method contains two operations, pattern separation and novelty detection, and these two operations appear to be biologically plausible and may happen in Dentate Gyrus and CA1, respectively. We apply this method to memorize continuous morphed patterns and learn a CANN successfully. The result is shown in Figure 1.
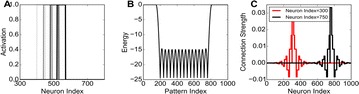


**Figure 1.** Learning a CANN from continuously morphed patterns. **A.** The morphed patterns. **B.** The learned network has an (*approximately*) flat subspace of low energy storing the morphed patterns, a key property of a CANN. **C.** The learned neuronal connection weights are translational invariant in space, a characteristic of a CANN

**References**

1. Wu S, Wong KM, Fung CA, Mi Y, Zhang W: Continuous attractor neural networks: candidate of a canonical model for neural information representation. *F1000Research* 2016, **5.**

2. Kropff E, TrevesA: Uninformative memories will prevail: the storage of correlated representations and its consequences. *HFSP journal* 2007, **1(4),** 249–262.

3. Blumenfeld B, Preminger S, Sagi D, Tsodyks M: Dynamics of memory representations in networks with novelty-facilitated synaptic plasticity. *Neuron* 2006, **52(2),** 383–394.

4. Srivastava V, Sampath S, Parker DJ: Overcoming Catastrophic Interference in Connectionist Networks Using Gram-Schmidt Orthogonalization. *PloS one* 2014, **9(9),** e105619.

## P295 Learning Peri-saccadic Receptive Field Remapping in Lateral Intraparietal Area from Visual Experience

### Xiao Wang, Mingsha Zhang, and Si Wu

#### State Key Laboratory of Cognitive Neuroscience & Learning, IDG/McGovern Institute for Brain Research, Beijing Normal University, Beijing 100875, China

##### Correspondence: Xiao Wang (wxbnu@mail.bnu.edu.cn), Mingsha Zhang (mingsha.zhang@bnu.edu.cn), Si Wu (wusi@bnu.edu.cn)

*BMC Neuroscience* 2017, **18** (**Suppl 1**):P295

Our eyes move constantly at a frequency of 3–5 times per second, called saccade. A saccade induces sweeping of visual images on the retina, yet we perceive the world to be stable. It has been suggested that the brain achieves this visual stability via predictive remapping of neuronal receptive field (RF), i.e., neurons respond to stimuli appearing in their future receptive fields (FRFs) before the eye actually moves [1]. Recently, Wang et al. unveiled the detailed time course of neuronal peri-saccadic remapping in the lateral intraparietal area (LIP) [2]. They found that around a saccade, the neuronal RF expands along the saccadic trajectory temporally, covering the current RF (CRF), the FRF, and the region the eye will sweep through during the saccade. A cortical wave (CW) model was also proposed, which attributes RF remapping as the consequence of neural activities propagated in LIP triggered by the joint effect of visual stimuli and the corollary discharge (CD) signal responsible for the saccade [2]. This CW model successfully reproduced the experimental data, however, its biological plausibility remains unresolved. In this study, we address this issue by building up a computational model to demonstrate that the CW model can be naturally learned from visual experiences at the developmental stage of the brain via the biologically plausible spiking-time-dependent-plasticity (STDP).

We build a two-layer network, with one layer consisting of LIP neurons and the other Superior Colliculus (SC) neurons. Initially, neuronal connections in LIP are bidirectional and weak, and the connections from SC to LIP are non-selective. An eye movement due to saccade causes a static visual image to “sweep” through the retina passively as if the visual stimulus is moving in the opposite direction of the saccade. This “moving” stimulus activates LIP neurons sequentially in the retinotopic map; meanwhile, SC neurons which convey the saccadic information also respond due to the CD signal. Suppose this process repeats many times, according to STDP, a connection path in the opposite direction of the saccade between LIP neurons will be learned, and a connection from SC to LIP matching the saccadic and remapping directions will be formed. Over many such visual experiences at different spatial locations and in different directions, a remapping network in LIP is completed. Consequently, a visual stimulus at FRF, combined with the CD signal from SC, can elicit a cortical wave in LIP which propagates from FRF to CRF of the neuron along the opposite direction of the saccade, exhibiting the peri-saccadic RF remapping phenomenon as observed in the experiment.

**References**

1. Duhamel JR, Colby CL, Goldberg ME: The updating of the representation of visual space in parietal cortex by intended eye movements [J]. *Science* 1992, **255(5040):** 90.

2. Wang X, Fung CA, Guan S, Wu S, Goldberg ME, Zhang M: Perisaccadic receptive field expansion in the lateral intraparietal area. *Neuron* 2016, **90(2):**400–409.

## P296 Axonal tree morphology dictates information coding

### Netanel Ofer^1,2^, Orit Shefi^1,2^, Gur Yaari^1^

#### ^1^Faculty of Engineering, Bar Ilan University, Ramat Gan, 52900, Israel; ^2^Institute for Nanotechnology and Advanced Materials, Bar Ilan University, Ramat Gan 5290002, Israel

##### Correspondence: Netanel Ofer (netanel.ofer@biu.ac.il)

*BMC Neuroscience* 2017, **18** (**Suppl 1**):P296

Neurons display various branching tree structures, and generate diverse activity patterns [1–3]. Studying the influence of morphology on electrical activity is of crucial importance for understanding brain functionality [4]. We have investigated the connection between tree structure and electrical activity by studying signal propagation and information coding along two basic morphological building blocks: unbranched axonal segments, and axonal branching points. We did it by solving numerically the Hodgkin Huxley model, as well as an adapted model for cortical neurons [5], that enable usage of parameter values that reflect better the conditions for the mammalian nervous system. In an unbranched axon, spike failures occur in high frequency trains. The effect on the propagated signal depends on the frequency of the spike train, axon diameter and axon length. In axonal branching points, signals are modified in a way that depends on the frequency of the spike train, and geometrical parameters of the branching point. Combined effects of these two elements can lead to asymmetric responses even between two sibling branches with identical diameters. These asymmetric conductions could be produced from geometrical properties alone. We have systematically characterized the firing patterns as a function of train frequency and morphometric parameters, revealing distinct patterns of activities such as trains, blockage, intermitted trains, single or several spikes, and stuttering. Adding up responses from many of these simple elements yields a rich repertoire of non-trivial activities that can be used as encoding mechanism for computational tasks. In light of these new theoretical results, we have extended our study to analyze real whole neuron structures, using reconstructed data obtained from publically available large data repositories from NeuroMorpho.Org [6] and the Blue Brain Project [7, 8]. We have examined the morphological parameters in all neuron types, and interneurons in particular. We clustered interneurons by their geometrical parameters and divided them into groups according to the signal modulations that their geometry dictates. Our results may advance interneurons classification by axonal tree morphology, suggesting that different cells generate different activity patterns. This detailed morphometric description of cells, together with understanding how geometry determines information flow, opens the door for deducing functionality from anatomical data.

**References**

1. DeFelipe J, López-Cruz PL, Benavides-Piccione R, Bielza C, Larrañaga P, Anderson S, et al. New insights into the classification and nomenclature of cortical GABAergic interneurons. *Nature Reviews Neuroscience.* 2013; **14:**202–216.

2. Kepecs A, Fishell G. Interneuron cell types are fit to function. *Nature.* 2014; **505:**318–326.

3. Polavaram S, Gillette TA, Parekh R, Ascoli GA. Statistical analysis and data mining of digital reconstructions of dendritic morphologies. *Frontiers in neuroanatomy*. 2014; **8:**138.

4. Ofer N, Shefi O. Axonal geometry as a tool for modulating firing patterns. *Applied Mathematical Modelling*. 2016; **40:**3175–84.

5. Mainen ZF, Sejnowski TJ. Influence of dendritic structure on firing pattern in model neocortical neurons. *Nature.* 1996;382:363.

6. Ascoli GA, Donohue DE, Halavi M. NeuroMorpho.Org: A Central Resource for Neuronal Morphologies. J. Neurosci. 2007; **27:**9247–51.

7. Markram H, Muller E, Ramaswamy S, Reimann MW, Abdellah M, Sanchez CA, et al. Reconstruction and simulation of neocortical microcircuitry. *Cell.* 2015; **163:**456–492.

8. Ramaswamy S, Courcol J-D, Abdellah M, Adaszewski SR, Antille N, Arsever S, et al. The neocortical microcircuit collaboration portal: a resource for rat somatosensory cortex. Frontiers in neural circuits [Internet]. 2015 [cited 2015 Dec 17];9. Available from: http://www.ncbi.nlm.nih.gov/pmc/articles/PMC4597797/

## P297 The Neuroscience Gateway Portal–High Performance Computing for Neuroscientists

### Ted Carnevale^1^, Amit Majumdar^2^, Subhashini Sivagnanam^2^, Kenneth Yoshimoto^2^

#### ^1^Neuroscience Department, Yale University, New Haven, CT 06510, USA; ^2^San Diego Supercomputer Center, University of California San Diego, San Diego, CA 92093-0505, USA

##### Correspondence: Ted Carnevale (ted.carnevale@yale.edu)

*BMC Neuroscience* 2017, **18** (**Suppl 1**):P297

The Neuroscience Gateway Portal [1] (NSG http://www.nsgportal.org) catalyzes computational neuroscience research that involves large scale simulations and/or data analysis by lowering or eliminating administrative and technical barriers to using High Performance Computing (HPC) resources. It does this by

(1) providing free and open access to supercomputers using time that is acquired via the peer reviewed allocation process managed by the Extreme Science and Engineering Discovery Environment (XSEDE), the virtual organization that coordinates US academic supercomputer centers

(2) offering a simple web-based user interface for accessing HPC resources and

(3) more recently, adding a RESTful interface that enables programmatic access to HPC resources.

NSG is enabling participation by the wider neuroscience community in research that would otherwise involve too great a computational burden, such as large scale and detailed models of cells and networks, parameter optimization, brain image processing, connectome pipelines etc. Since its inception in early 2013, it has provided about 10,000,000 core hours of supercomputer time to neuroscientists, and has enabled more than 50 publications and posters. In addition, many developers of new network modeling tools, data driven parameter optimization pipelines (e.g. BluePyOpt from the Human Brain Project), and data analysis tools are using the NSG to disseminate their results to the neuroscience community.

NSG currently has about 450 registered users. Total core hour usage, per-user core hour consumption rate, and the number of users have all been growing at a rapid rate; annual usage is projected to exceed 10,000,000 core hours in 2017.

Developing and operating the NSG has given us a unique opportunity to understand and analyze how a very diverse range of neuroscientists are using an environment like the NSG, and examine their growing need for supercomputer power, as well as associated issues and needs for collaboration, data sharing/management and various forms of computing.

**Acknowledgements**

Supported by NSF 1458840 (A.M., S.S., K.Y); NSF 1458495, NIH/NIDCD DC 009977, NIH/NINDS NS 011613 (T.C).

**Reference**

1. Sivagnanam S, Majumdar A, Yoshimoto K, Astakhov V, Bandrowski A, Martone ME, Carnevale NT: Introducing the Neuroscience Gateway, *Proceedings of the 5th International Workshop on Science Gateways*, volume 993, CEUR-WS.org, 2013. [http://ceur-ws.org/Vol-993/paper10.pdf]

## P298 Changes of electrophysiological neuronal properties in lithium-pilocarpine model of epilepsy

### Elena Y. Smirnova^1,2^, Dmitry V. Amakhin^2^, Sergey L. Malkin^2^, Anton V. Chizhov^1,2^, Aleksey V. Zaitsev^2^

#### ^1^Ioffe Institute, St.-Petersburg, 194021, Russia; ^2^Sechenov Institute of Evolutionary Physiology and Biochemistry of RAS, St.-Petersburg, 194223, Russia

##### Correspondence: Elena Y. Smirnova (elena.smirnova@mail.ioffe.ru)

*BMC Neuroscience* 2017, **18** (**Suppl 1**):P298

The lithium-pilocarpine (LP) model of epilepsy belongs to a group of animal models that replicate the general progression of events as observed in humans [1]. We aimed to determine, whether LP-induced status epilepticus (SE) affects electrophysiological properties of neurons and then, if does, what is the nature of the effect. Using whole-cell patch-clamp we compared passive properties, single-spike and spike-pattern attributes of pyramidal neurons in entorhinal cortex (ERC) slices of control (53 cells) and LP-treated rats (47 LP-cells, recorded in a day after SE), as well as in, presumably, less damaged prefrontal cortex (37 control vs 35 LP-neurons). LP-cells had reduced input resistance (*R*_*in*_), time constant (*τ*_*m*_), first instantaneous frequency (*IF*_*1*_) and amplitude of spikes (*RA*), and increased rheobase (*Rb*) and current inducing maximal firing rate (*I*_*max*_). Apart from the decrease of spike amplitude, all the effects were stronger in ERC. Because the decrease of *R*_*in*_ can explain all the other effects, we then aimed to clarify whether *R*_*in*_ change is caused by the disturbances of synaptic, passive or active channels.

First, we compared electrophysiological properties before and after the blockade of synaptic currents by APV, DNQX and bicuculline. Analysis showed that all of the parameters changed by SE sustain after the blockade of synaptic currents. Thus, the main source of SE-induced changes is not synaptic.

Next, we used the dynamic-clamp which allowed us to simulate additional potassium and nonspecific currents [2]. We attempted to clarify which of the neuronal properties are affected by the leak current (*I*_*L*_) and which ones are by the potassium current which induces spike adaptation (*I*_*ad*_). The effect of *I*_*L*_ in control rats was similar to the effect of SE. Addition of the leak current led to statistically significant decreases of *R*_*in*_, *τ*_*m*_, *I*_*max*_, *IF*_*1*_, *RA*, magnitude of sag, and increases Rb, stationary *IF*, and gain (only in ERC). We then mimicked *I*_*ad*_ by using the approximation from [3]. Addition of *I*_*ad*_ did not change *R*_*in*_, increases *IF*_*1*_, and decreases firing rate, as well as time to the first spike.

Thus, the decrease of *R*_*in*_ after SE is more likely to be induced by *I*_*L*_ as well as the decrease of *τ*_*m*_, time to the first spike, *RA*, *IF*_*1*_ and increase of Rb. Overall, our results suggest that LP-induced SE mainly increases the leak conductance and keeps other factors intact.

**Acknowledgements**

This work was supported by the Russian Science Foundation (project 16-15-10202).

**References**

1. Furman M: Seizure Initiation and Propagation in the Pilocarpine Rat Model of Temporal Lobe Epilepsy. *J Neurosci* 2013, **33(42)**:16409 –16411.

2. Smirnova EY, Zaitsev AV, Kim KK, Chizhov AV: The domain of neuronal firing on a plane of input current and conductance. *J Comput Neurosci* 2015, **39(2)**: 217–233.

3. Kopell N, Ermentrout GB, Whittington MA, Traub RD: Gamma rhythms and beta rhythms have different synchronization properties. *Proc Nat Acad Sci USA 15* 2000, **97(4):**1867–1872.

## P299 Depolarizing GABA leads to interneuron-based interictal discharges: experimental and mathematical models

### Anton V. Chizhov^1,2^, Dmitry V. Amakhin^2^, Aleksey V. Zaitsev^2^

#### ^1^Ioffe Institute, St.-Petersburg, 194021, Russia; ^2^Sechenov Institute of Evolutionary Physiology and Biochemistry of RAS, St.-Petersburg, 194223, Russia

##### Correspondence: Anton V. Chizhov (anton.chizhov@mail.ioffe.ru)

*BMC Neuroscience* 2017, **18** (**Suppl 1**):P299

In in vitro experimental model of temporal lobe epilepsy, we observe the repeating sequences of interictal discharge (IID) regimes and seizure-like events, where IID are initiated by interneurons. We used an extracellular medium with high potassium/low magnesium concentration with the addition of 4-AP in order to provoke epileptiform activity in combined hippocampus/entorhinal cortex slices of the rat brain [1]. Two types of IID were observed. For each type, AMPA, NMDA, and GABA-A synaptic components have been estimated by means of multiple recordings on different voltage levels in the voltage-clamp whole cell configuration. As found, IIDs of the first type (IID1) reflect synchronization in the network of interneurons, thus they are characterized by a pure GABAergic current recorded in an excitatory neuron. IIDs of the second type (IID2) are composed of early GABAergic and later glutamatergic components.

We have reproduced the IIDs in our mathematical model, using the conductance-based refractory density approach [2] which provides both a biophysically detailed description of neuronal populations in terms of ionic channel conductances for one- or two-compartment neurons and good precision for statistically equilibrium and non-equilibrium regimes of ensemble activity. Coupled excitatory and inhibitory neurons interact via glutamatergic and GABAergic plastic synapses. IID1 s and IID2 s were well reproduced in the model. In simulations, the only parameter that controlled the regimes was the reversal potential of GABA-A current, *V*_GABA_. Switching from the control silent state to IID1 s and then to intermittent IID2 s and IID1 s, and finally, only IID2 s occurs with depolarization of *V*_GABA_. We hypothesize that in the experiments *V*_GABA_ was depolarized because of depressed action of potassium-chloride cotransporters in the conditions with high extracellular potassium concentration. We have also found the synaptic depression to be a crucial factor, which provides ceasing of each of the discharges and determines their duration. Overall, our study reveals the mechanisms of pathological synchronization with the primary role of excitatory GABA receptors in the interneuronal network.

**Acknowledgements**

This work was supported by the Russian Science Foundation (project 16-15-10201).

**References**

1. Amakhin DV, Ergina JL, Chizhov AV, Zaitsev AV: Synaptic Conductances during Interictal Discharges in Pyramidal Neurons of Rat Entorhinal Cortex. *Front. in Cell. Neuroscience* 2016, **10: 233.**

2. Chizhov AV, Graham LJ: Population model of hippocampal pyramidal neurons, linking a refractory density approach to conductance-based neurons. *Phys Rev E* 2007, **75: 011924.**

## P300 Neural activity in distinct navigation modes of flying pigeons

### Margarita Zaleshina, Alexander Zaleshin

#### Moscow Institute of Physics and Technology, Moscow, 117303, Russia

##### Correspondence: Margarita Zaleshina (zaleshina@gmail.com)

*BMC Neuroscience* 2017, **18** (**Suppl 1**):P300

In spatial navigation, there are certain tasks of choosing a route or correcting a route depending on external conditions. It is necessary to respond to changes in the visual environment, to compare the expected and observed landscape. Quick reaction is required in case of unexpected obstacles on the pathway. Perception is detailed as the target is approached. Orientation awareness is accompanied by various types of neuronal activities. It can be observed in brain cells associated with navigation (including place cells, grid cells, head-direction cells) [1], and in combinations of rhythms of neural ensembles [2]. Contributions of different band oscillations during route selection can be independent [3]. Observed brain activities are different in tasks with the specified position of the visual cue or with underspecified movement goal [4].

This work proposes a model to identify the characteristics of brain activity of flying pigeons with different modes of space perception. Pigeons fly home based on familiar landmarks and landscape features [5], solar, stellar and magnetic cues, polarized light patterns [6], and other references to geographical location. Pigeons have color and ultraviolet vision, their eyes distinguish the 75 frames per second, field of view is 340 degrees. Comparison of EEG responses to visual landmarks in flying pigeons was described [7].

The work considers pigeon flight on known route in three modes: 1. Stationary flight at an altitude of 100-300 meters, speed of 60 km/h. For flight in a given direction it is necessary to take into account the influence of wind (drift angle). 2. Response to danger or sudden changes. Pigeons are more sensitive to radial motion when there is an acceleration as opposed to a constant velocity [8]. 3. Descent and landing. Birds begin to fly in circles at an altitude of 30-50 meters.

In the computational model, it is assumed that each mode is accompanied by a characteristic set of rhythms of neural ensembles (for quiet flight, for alarm and for approaching to visible goal). Representation of brain activity as sets of rhythms depends on the type of mode. In model, recognition of textures and borders in the mode “stationary flight” is additionally encoded by the phase of rhythms with lower frequency. Interactions between cortical rhythms may generate a third frequency [9]. Route reference points are additionally encoded by the amplitude of rhythms in all modes. QGIS (http://www.qgis.org) allows to integrate data received from various sources simultaneously. In the work, GPS track of flights and landscape maps are performed in QGIS (similarly, QGIS was applied in [6]). In addition, the program allows to combine results of EEG data processing with the spatial characteristics of pigeon flight. In the spatial representation of the model takes into account the distances between the reference points on the ground.

**References**

1. Spiers HJ, Barry C: Neural systems supporting navigation. *Curr Opin Behav Sci Elsevier Ltd* 2015, **1:**47–55.

2. Watrous AJ, Fell J, Ekstrom AD, Axmacher N: More than spikes: Common oscillatory mechanisms for content specific neural representations during perception and memory. *Curr Opin Neurobiol Elsevier Ltd* 2015, **31:**33–39.

3. Brinkman L, Stolk A, Marshall TR, Esterer S, Sharp P, Dijkerman HC, et al.: Independent Causal Contributions of Alpha- and Beta-Band Oscillations during Movement Selection. *J Neurosci* 2016, **36:**8726–8733.

4. Gertz H, Lingnau A, Fiehler K: Decoding Movement Goals from the Fronto-Parietal Reach Network. *Front Hum Neurosci* 2017, **11:**84.

5. Gagliardo A, Ioale P, Savini M, Dell’Omo G, Bingman VP: Hippocampal-dependent familiar area map supports corrective re-orientation following navigational error during pigeon homing: A GPS-tracking study. *Eur J Neurosci* 2009, **29:**2389–2400.

6. Blaser N, Guskov SI, Meskenaite V, Kanevskyi VA, Lipp HP: Altered Orientation and Flight Paths of Pigeons Reared on Gravity Anomalies: A GPS Tracking Study. *PLoS One* 2013, **8(10):**e77102.

7. Vyssotski AL, Dell’Omo G, Dell’Ariccia G, Abramchuk AN, Serkov AN, Latanov AV., et al.: EEG Responses to Visual Landmarks in Flying Pigeons. *Curr Biol Elsevier Ltd* 2009, **19:**1159–1166.

8. Nankoo JF, Madan CR, Spetch ML: Wylie DR: Perception of complex motion in humans and pigeons (Columba livia). *Exp Brain Res* 2014, **232:**1843–1853.

9. Roopun AK: Temporal interactions between cortical rhythms. *Front Neurosci* 2008, **2:**145–154.

## P301 The Role of the Receptive Field Structure in Neuronal Compressive Sensing Signal Processing

### Victor J. Barranca, George Zhu

#### Department of Mathematics and Statistics, Swarthmore College, Swarthmore, PA 19081, USA

##### Correspondence: Victor J. Barranca (vbarran1@swarthmore.edu)

*BMC Neuroscience* 2017, **18** (**Suppl 1**):P301

The receptive field structure ubiquitous in the visual system is believed to play a crucial role in encoding stimulus characteristics, such as contrast and spectral composition. However, receptive field architecture may also result in unforeseen difficulties in processing particular classes of images. We explore the potential functional benefits and shortcomings of localization and center-surround paradigms in the context of an integrate-and-fire neuronal network model. Utilizing the sparsity of natural scenes, we derive a compressive-sensing based theoretical framework for network input reconstructions based on neuronal firing rate dynamics [1, 2]. This formalism underlines a potential mechanism for efficiently transmitting sparse stimulus information, and further suggests sensory pathways may have evolved to take advantage of the sparsity of visual stimuli [3, 4]. Using this methodology, we investigate how the accuracy of image encoding depends on the network architecture.

We demonstrate that the receptive field structure does indeed facilitate marked improvements in natural stimulus encoding at the price of yielding erroneous information about specific classes of stimuli. Relative to uniformly random sampling, we show that localized random sampling yields robust improvements in image reconstructions, which are most pronounced for natural stimuli containing a relatively large spread of dominant low frequency components. This suggests a novel direction for compressive sensing theory and sampling methodology in engineered devices. However, for images with specific gray-scale patterning, such as the Hermann grid depicted in Fig. 1, we show that localization in sampling produces systematic errors in image encoding that may underlie several optical illusions. We expect that these connections between input characteristics, network topology, and neuronal dynamics will give new insights into the structure-function relationship of the visual system.
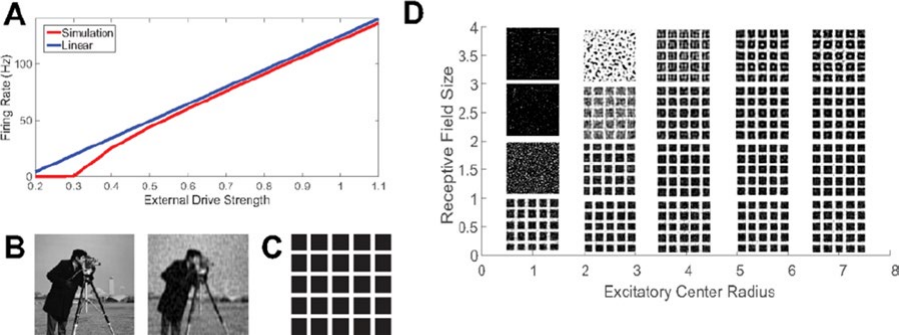


**Figure 1. A.** The network-averaged firing rate dependence on the external-drive strength scaling, computed using model simulation and theoretical linear input-output mapping. **B.** Original image (left) and CS reconstruction (right) using localized random sampling of the network dynamics. **C.** Hermann grid illusion. **D.** Reconstructions of (C) for various choices of receptive field size scaling and excitatory center region radius

**References**

1. Field DJ: What is the Goal of Sensory Coding? *Neural Comput.* 1994, **6**: 559–601.

2. Candes EJ, Romberg JK, Tao T: Stable Signal Recovery from Incomplete and Inaccurate Measurements. *Commun Pur Appl Math.* 2006, **59(8):** 1207–1223.

3. Barranca VJ, Kovacic G, Zhou D, Cai D: Sparsity and Compressed Coding in Sensory Systems. *PLoS Comp Biol.* 2014, **10(8):**e1003793.

4. Barranca VJ, Kovacic G, Zhou D, Cai D: Improved Compressive Sensing of Natural Scenes using Localized Random Sampling. *Sci Rep.* 2016, **6:**31976.

## P302 FuNS with E/I balance: critical dynamics maximize stability of neural networks

### Quinton M. Skilling^1^, Daniel Maruyama^2^, Nicolette Ognjanovski^3^, Sara J. Aton^3^, and Michal Zochowski^1,2^

#### ^1^Biophysics Program, University of Michigan, Ann Arbor, MI 48109, United States; ^2^Department of Physics, University of Michigan, Ann Arbor, MI 48109, United States; ^3^Department of Cellular, Molecular, and Developmental Biology, Ann Arbor, MI 48109, United States

##### Correspondence: Quinton M. Skilling (qmskill@umich.edu)

*BMC Neuroscience* 2017, **18** (**Suppl 1**):P302

The mammalian brain naturally balances excitation and inhibition. This results in complex dynamics vital for important cognitive functions such as the formation of new memories. Excitatory/inhibitory (E/I) balance has been shown to result in scale-free distributions of population behavior known as neuronal avalanches, a hallmark of self-organized criticality in the brain. Recently, we have shown using models well-rooted in physics that new memories are stored only when the system dynamics reside near a critical point and are characterized by enhanced stability of spiking activity which we refer to as functional network stability (FuNS) [1]. Here, we expand on this work through direct modeling of neuronal networks where E/I balance is tightly controlled. Proximity to criticality at E/I balance is verified via calculation of neuronal avalanches as well as through calculating functional connectivity correlation between neurons for increasing separation distance between them. Introducing a region of increased coupling, such as the synaptic potentiation involved in learning, increases FuNS in networks exhibiting E/I balance significantly over networks whose dynamics arise primarily through excitatory or inhibitory inputs. Our results indicate that networks with balanced excitation and inhibition have an increased ability to store memories through increased functional network stability, a phenomenon due in part to critical dynamics in neural systems.

**Reference**

1. Skilling QM et al. 24 Feb 2017. “Criticality, stability, competition, and consolidation of new representations in brain networks.” *arXiv: 1702.07649.*

## P303 Neural oscillations modulate the network dynamics around E-I balance in memory consolidation

### Jiaxing Wu^1^, Nicolette Ognjanovski^2^, Sara Aton^2^, Michal Zochowski^3^

#### ^1^Applied Physics Program, University of Michigan, Ann Arbor, MI, 48109, USA; ^2^Department of Molecular, Cellular, and Developmental Biology, University of Michigan, Ann Arbor, MI, 48109, USA; ^3^Biophysics Program and Department of Physics, University of Michigan, Ann Arbor, MI, 48109, USA

##### Correspondence: Jiaxing Wu (jxwu@umich.edu)

*BMC Neuroscience* 2017, **18** (**Suppl 1**):P303

Rhythmic activities of different frequency bands have been observed universally in the brain and are thought to play important roles in various cognitive processes [1]. However, the fundamental mechanism of how these neural oscillations contribute to brain activities is still an open question. Recently, via both computational simulations and in vivo experiments, we found that oscillations are essential for memory consolidation as they mediate network functional stability. We have shown computationally, that various network properties such as firing rate, synchrony, mean phase coherence are enhanced in the presence of external oscillations around Excitatory-Inhibitory (E-I) balance, where E-I ratio is calculated based on the excitatory and inhibitory synaptic strength and neuronal firing frequency. We have investigated this effect for both type 1 (integrator) neurons as well as type 2 (resonator) cells. The networks composed of resonator neurons are more sensitive to the oscillatory drive than the networks composed of integrator neurons, however both show significant changes in firing patterns. We show that global oscillations causally organize firing patterns between heterogeneous networks composed of dense neuronal clusters that are loosely connected with each other, facilitating communication and information transfer between spatially distributed brain regions. Most importantly, near Excitatory-Inhibitory (E-I) balance, oscillations increase both functional connectivity between neurons and coherence between spikes and local field potential (LFP), as well as enhance network functional stability, thus leading to faster changes in network structural connectivity patterns thought to underlie learning and memory consolidation. These in silico observations are supported by our experimental data [2]. In summary, our results show that neural oscillations together with network state near E/I balance coordinate the network dynamics and contribute to memory consolidation.

**References**

1. Buzsaki G: Rhythms of the Brain, *Oxford University Press* 2006.

2. Ognjanovski N, Schaeer S, Wu J, Mofakham S, Maruyama D, Zochowski M, Aton SJ: Parvalbumin-expressing interneurons coordinate hippocampal network dynamics required for memory consolidation. *Nature Communications* (In Press).

## P304 Cellular and network properties of interneuron networks dictate variable clustering patterns in both strictly inhibitory and E-I neural networks

### Scott Rich^1^, Victoria Booth^2^, Michal Zochowski^3^

#### ^1^Applied and Interdisciplinary Mathematics Program, University of Michigan, Ann Arbor, MI 48104, USA; ^2^Departments of Anesthesiology and Mathematics, University of Michigan, Ann Arbor, MI 48104, USA; ^3^Departments of Physics and Biophysics, University of Michigan, Ann Arbor, MI 48104, USA

##### Correspondence: Scott Rich (sbrich@umich.edu)

*BMC Neuroscience* 2017, **18** (**Suppl 1**):P304

The diverse population of interneurons in the hippocampus is pivotal to the formation of oscillatory electrical activity that contributes to memory processing [1], while in the cortex such interneurons and rhythms are implicated in potential mechanisms underlying selective attention [2]. Computational research has shown that these rhythms can be generated in purely inhibitory networks or networks with both excitatory and inhibitory neurons (E-I networks). However, the dynamics and mechanisms generating them depend on properties of the inhibitory network.

Simulations of strictly inhibitory networks in our recently published work [3] demonstrate that the intrinsic cellular properties and connectivity density alter the bursting properties exhibited in randomly connected, heterogeneous inhibitory networks. We analyze networks with three types of model neurons that are classified into the classical Type I or Type II categories using their current-frequency relation (IF curve) and Phase Response Curve (PRC), while additionally investigating the role of an M-type adaptation current adaptation current. Across simulations we vary the degree of cellular heterogeneity, the intrinsic firing frequency of neurons, and the time scale of decay of synaptic inhibition. The observed dynamics often differ from those predicted by the Interneuron Network Gamma (ING) mechanism [4], as well as from results in all-to-all connected networks. While the networks studied here synchronize into a single cluster of active neurons when said neurons are Type I, analogous networks of Type II neurons without adaptation segregate into two mutually exclusive clusters organized by the cells’ intrinsic firing frequencies. When the neurons are modeled as Type II with adaptation, we observe dynamics similar to those seen in networks of either Type I or Type II neurons depending upon network parameters, although the adaptation current does imbue these networks with additional unique behaviors. The mechanisms underlying this variety of dynamics is explained by changes in profiles of the PRCs across the different neuron types.

One additional property of Type I inhibitory networks is the different synchrony patterns exhibited when the inhibitory synapses are strong or weak. By expanding our research to E-I networks, we have shown that this property plays an important role in the dynamics of excitatory neurons in these larger networks. When inhibitory to inhibitory synapses (I-I) in an E-I network are sufficiently strong, the dynamics match those predicted by the PING mechanism [5]. When these synapses are weakened, networks exhibit rhythmic bursting for weaker excitatory to inhibitory connectivity and can exhibit rhythms slower than the typical gamma frequency, two features that expand upon the types of dynamics typically described by PING theory. However, with weak I-I synapses, the dynamics of the excitatory cell bursts tend to become disorganized and aperiodic for stronger excitatory to inhibitory connectivity, due to more variable activity patterns in the inhibitory network. These results indicate that the strength of I-I connectivity plays a crucial role in dictating the type, strength and robustness of excitatory bursting patterns in an E-I network, analogously to how cell type dictates the type of dynamics seen in strictly inhibitory networks.

**References**

1. Bartos M, Vida I, Jonas P: Synaptic mechanisms of synchronized gamma oscillations in inhibitory interneuron networks. *Nat. Rev. Neurosci.* 2007, **8:** 45–56.

2. Fries P: A mechanism for cognitive dynamics: neuronal communication through neuronal coherence. *Trends Cogn. Sci.* 2005, **9:** 474–480.

3. Rich S, Booth V, Zochowski M: Intrinsic cellular properties and connectivity density determine variable clustering patterns in randomly connected inhibitory neural networks. *Front. Neural Circuits* 2016, **10.**

4. Whittington M, Traub RD, Kopell N, Ermentrout B, Buhl E. Inhibition-based rhythms: experimental and mathematical observations on network dynamics. *Int. J. Psychophysiol.* 2000: **38**, 315–336.

5. Kopell N, Borgers C, Pervouchine D, Malerba P, Tort A. Gamma and theta rhythms in biophysical models of hippocampal circuits in *Microcircuits, A Computational Modeler’s Resource Book, Springer Series in Computational Neuroscience*. 2010: 423–457.

## P305 Synaptic failure in functional network activity

### Maral Budak^1^, Michal Zochowski^1,2^

#### ^1^Department of Biophysics, University of Michigan, Ann Arbor, Michigan, 48109, USA; ^2^Department of Physics, University of Michigan, Ann Arbor, Michigan, 48109, USA

##### Correspondence: Maral Budak (mbudak@umich.edu)

*BMC Neuroscience* 2017, **18** (**Suppl 1**):P305

Human brain is a complex network including 10^11^ neurons connected by 10^14^ synapses. Information processing occurs in separate, functionally specialized regions, which also coordinate and integrate efficiently for the brain to function in a coherent way. To exhibit these features, brain networks are considered to have small-world and scale-free network structure properties [1].

Neurons in the brain communicate with each other via connections called synapses in order to be able to synchronize and perform certain tasks. Thus, failures at these synaptic connections have detrimental effects such as loss of consciousness or neurodegenerative diseases. Our objective is understanding the effect of decreased synaptic transmission on brain networks as a whole as well as on the formation of globally coherent states. The results may be applied to understand how anesthetics brings the loss of consciousness by changing brain dynamics and early diagnosis of some neurodegenerative diseases such as Alzheimer’s and ALS, in which cases synaptic failure is the earliest symptom [2, 3]. For this purpose, both small-world and scale-free networks, which are prevalent in brain, are modeled with integrate- and-fire excitatory neurons. Synaptic failure is introduced to the model by a parameter which randomly determines whether neurons get signals from the others they’re connected to or not. This parameter also depends on the spiking history of the neurons, i.e. synapses are more likely to fail if the presynaptic neuron is more recently fired.

After various simulations, quantitative measures are done with neurons’ spike times in order to determine how synchronous and coherent the networks behave. As a result, we demonstrate that more local connections favor more coherent and synchronous behavior with increasing synaptic transmission. Moreover, we show that scale-free networks with different directionalities respond to synaptic failure in different ways. However, neurons with moderate degrees are more coherent than other neurons in all scale-free network structures. Also, when hubs in scale-free networks are disconnected, the effect is bigger than the disconnection of lower-degree neurons on the network. We also observed that the dependence on spiking history affects synchronization and coherent state formation in different ways for different network structures.

**References**

1. Bocaletti S, Latora V, Moreno Y, Chavez M, Hwang D: Complex networks: Structure and dynamics. *Physics Reports*. 2006;**424(4-5):**175–308.

2. Wishart T, Parson S, Gillingwater T: Synaptic Vulnerability in Neurodegenerative Disease. *Journal of Neuropathology & Experimental Neurology.* 2006;**65(8):**733–739.

3. Alkire M, Hudetz A, Tononi G: Consciousness and Anesthesia. *Science.* 2008;**322(5903):**876–880.

## P306 Data-driven multiscale model of mouse M1 microcircuits

### Salvador Dura-Bernal^1^, Samuel A. Neymotin^1,3^, Benjamin A. Suter^4^, Gordon M. G. Shepherd^2^, William W. Lytton^1,5^

#### ^1^Department Physiology & Pharmacology, SUNY Downstate, Brooklyn, NY 11203, USA; ^2^Department Physiology, Northwestern University, Chicago, IL 60611, USA; ^3^Department Neuroscience, Brown University, Providence, RI 02912, USA; ^4^Institute of Science and Technology (IST) Austria, Klosterneburg, 3400, Austria; ^5^Kings County Hospital Center, Brooklyn, NY 11203, USA

##### Correspondence: Salvador Dura-Bernal (salvadordura@gmail.com)

*BMC Neuroscience* 2017, **18** (**Suppl 1**):P306

We developed a detailed multiscale computational model of mouse primary motor cortex (M1) microcircuits, based on novel data provided by experimentalist collaborators. The model simulates at scale a cylindrical volume with a diameter of 300 μm and cortical depth 1350 μm of M1. It includes over 10,000 cells distributed across cortical layers based on measured cell densities, with more than 40 million synaptic connections. Neuron models were optimized to reproduce the current-clamp electrophysiological properties of major classes of M1 neurons, with a special emphasis on layer 5 corticospinal (SPI) and corticostriatal (STR) neurons. Cell ionic channel distributions were constrained by literature and optimized to reproduce with high precision in vitro recordings for these two cell types, which used detailed cell morphologies with 700 + compartments from morphological reconstructions. The network was driven by the main long-range inputs to M1: thalamus, primary and secondary somatosensory cortex (S1, S2), contralateral M1, secondary motor cortex (M2), and orbital cortex (OC). Local and long-range network connectivity was based on optogenetic circuit mapping studies which have demonstrated that connection strength cannot be fully defined by layer identification but depends strongly on cortical depth and on the subtype of pyramidal cell. Therefore, highly specific synaptic input positional distribution along dendritic trees of these different types were incorporated. We hypothesize that these distinct patterns of dendritic innervation will have different effects that reflect roles in multiple co-existing neural coding patterns.

The model was developed using NetPyNE, a novel Python package that facilitates the development of biological neuronal networks in the NEURON simulator, and emphasizes the incorporation of multiscale anatomical and physiological data at varying levels of detail. Parallel simulations were executed on the XSEDE San Diego Supercomputer Center. Our M1 model incorporates quantitative experimental data from 14 publications, therefore accumulating previously isolated knowledge into a unified framework.

We studied the output of the 15 local M1 populations in response to increased input from each of the seven long-range inputs modeled. Sensory information from S1, S2 and sensory thalamus primarily modulated M1 superficial layers, which projected unidirectionally to layer 5B corticospinal neurons. Secondary motor cortical areas, as well as basal ganglia-relaying thalamic direct inputs, also modulated the same layer 5B circuits. Firing rates, oscillations, and information transfer (measured using Granger causality and normalized transfer entropy) demonstrated differences in dynamics and information flow along the two parallel pathways is encoded, transformed and integrated. At the dendritic scale, the distinct innervation patterns in corticospinal neurons facilitated the integration of information from distinct regions, and the modulatory role of HCN (H current) ion channels, which has been hypothesized to provide a mechanism to translate action planning into action execution.

**Acknowledgements**

Research supported by NIH grant U01EB017695, NIH R01EB022903 and NIH R01MH086638.

## P307 Membrane resonance and oscillation preferences of a multi-compartment model pyramidal neuron

### Melvin A. Felton Jr^1^, Alfred B. Yu^2^, David L. Boothe^2^, Kelvin S. Oie^2^, Piotr J. Franaszczuk^2, 3^

#### ^1^U. S. Army Research Laboratory, Adelphi, MD 20783, USA; ^2^U. S. Army Research Laboratory, Aberdeen Proving Ground, MD 21005, USA; ^3^Department of Neurology, Johns Hopkins University School of Medicine, Baltimore, MD 21287, USA

##### Correspondence: Melvin A. Felton Jr (melvin.a.felton.civ@mail.mil)

*BMC Neuroscience* 2017, **18** (**Suppl 1**):P307

In the soma of neocortical neurons, near-threshold depolarizations have been shown to induce subthreshold membrane potential oscillations that contribute to network oscillations by enhancing or hindering neuronal responses to synaptic inputs in specific frequency bands [1]. The frequency of these subthreshold membrane potential oscillations coincides with the peak resonance of the neuronal membrane. While differential responses within the soma of neocortical neurons to inputs of varying frequency have been well studied, the dendritic contribution within these same neurons is less clear [2], [3]. In addition, the differential impact on neuronal response properties of afferent inputs to different areas or “zones” of pyramidal neurons is not well understood.

We characterize resonance and membrane potential oscillation characteristics of a biophysically-realistic compartmental model of a neocortical layer 5 pyramidal neuron [4]. We simulated injected currents with varying temporal properties, including both step currents and sinusoidal currents with linearly increasing frequency (chirp currents), to determine the resonant properties of individual model compartments that were parameterized to reflect known differences in the properties of functionally distinct zones. We computed changes in membrane potential under different conditions of input current, and calculated the input and transfer impedance in the soma, initial axon segment, and along the dendrites. In addition, we calculated resonance strength and phase relationship between input current and output membrane potential.

The model showed that preferred oscillation frequency depends critically on parameters defining the ionic conductance of neuronal compartments that are active in the subthreshold range, which drive currents that contribute to the total membrane potential. These ionic currents include: hyperpolarization-activated anomalous rectifier, low-threshold inactivating calcium, persistent sodium, transient inactivating potassium, and muscarinic potassium (M current). For instance, resonance at low frequencies (1-4 Hz) can be produced by low-threshold calcium at near-rest/hyperpolarized potentials, resonance at intermediate frequencies (4-10 Hz) can be produced by anomalous rectifier at near-rest/hyperpolarized potentials, and resonance at intermediate and faster frequencies (4-30 Hz) is produced by M current from resting to more depolarized potentials.

The existence of perisomatic and distal dendritic zones whose intrinsic properties preferentially enhance or impede resonance in specific frequency ranges coincides with differential afferent connectivity [5]. Understanding the relationship between the intrinsic properties of these zones and the areas of the brain that target them could reveal additional insight about functional connectivity. Furthermore, the oscillatory behavior of the zones may modulate action potential initiation at the soma/axon hillock of a neuron which results in variability in overall network activity.

**References**

1. Erchova I, Kreck G, Heinemann U, Herz AVM: Dynamics of rat entorhinal cortex layer II and III cells: characteristics of membrane potential resonance at rest predict oscillation properties near threshold, J Physiol 2004, **560.1**: 89–110.

2. Yoshida M, Giocomo LM, Boardman I, Hasselmo ME: Frequency of subthreshold oscillations at different membrane potential voltages in neurons at different anatomical positions on the dorso-ventral axis in the rat medial entorhinal cortex, J Neurosci 2011, **31**(35): 12683–12694.

3. Remme MWH, Lengyel M, Gutkin BS: The role of ongoing dendritic oscillations in single-neuron dynamics, PLoS Comput Biol 2009, **5**(9): e1000493.

4. Bower JM, Beeman D: *The Book of Genesis, 2nd Edition.* New York: Springer-Verlag; 1998.

5. Zhuchkova E, Remme MWH, Schreiber S: Somatic versus dendritic resonance: differential filtering of inputs through non-uniform distributions of active conductances, PLOS One 2013, **8**(11): e78908.

## P308 Network cloning using DNA barcodes

### Sergey A. Shuvaev, Batuhan Başerdem, Anthony Zador, Alexei A. Koulakov

#### Cold Spring Harbor Laboratory, Cold Spring Harbor, NY 11724, USA

##### Correspondence: Alexei A. Koulakov (koulakov@cshl.edu)

*BMC Neuroscience* 2017, **18** (**Suppl 1**):P308

The ability to measure or manipulate network connectivity is the main challenge in the field of connectomics. Recently, a set of approaches has been developed that takes advantage of next generation DNA sequencing to scan connections between neurons into a set of DNA barcodes. Individual DNA sequences called markers represent single neurons, while pairs of markers, called barcodes contain information about connections. Here we propose a strategy for ‘copying’ or ‘cloning’ connectivity contained in barcodes into a clean slate *tabula rasa* network. We show that a one marker one cell (OMOC) rule, which forces all markers with the same sequence to condense into the same neuron, leads to fast and reliable formation of desired connectivity in a new network. We show that OMOC rule yields convergence in a number of steps given by a power law function of the network size. We thus propose that copying network connectivity from one network to another is theoretically possible. Most current implementations of artificial neural networks are on digital computers and GPUs [1]. On these architectures, connections are stored explicitly and therefore straightforward to extract and copy into a new network. However, in biological networks, there is no central repository for connections, so reading out the connections of a network and copying them into a new network represents a difficult challenge.
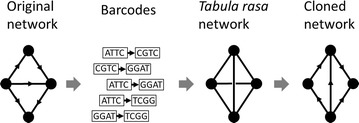


**Figure 1.** Network cloning as a way to copy connectivity from one network to another. The original network is read out into a set of barcodes carrying information about connections. Each half of the barcode (marker) represents one of the cells that are connected, while the link represents the direction of the connections. These barcodes are then introduced into a *tabula rasa* network that has no structure. Barcodes are capable to shape the *tabula rasa* network to match the target connectivity

We have recently proposed a new way to read out neuronal connections using DNA barcodes [2, 3]. In this strategy, individual neurons produce distinguishable pseudo-random DNA identifiers called markers. Pairs of markers, called here barcodes, represent individual synaptic connections (Figure 1). Barcodes are read out using high-throughput sequencing technology, either in situ [4] or ex vivo after individual neurons are disassociated. This strategy allows to convert connections between neurons into an ensemble of DNA barcodes that can be identified using sequencing methods. Here we formulate a different question: Given an ensemble of connections represented by barcodes, can we copy them into a new network? In other words, can original network be cloned? We explore a computational model that simulates the behavior of barcodes introduced into a *tabula rasa* network with unstructured connectivity and test its ability to recreate target connectivity in such networks (Fig. 1). We require that the underlying mechanisms be purely local, i.e. the behavior of each cell and barcode is based on the information available in this cell or in its synapses only. The particular form of dynamics that we considered is described by one marker one cell rule (OMOC), which favors positioning of a single type of marker DNA sequence in a single neuron. We showed that OMOC dynamics leads to fast and reliable recreation of desired connectivity in the new network. The formation of new connectivity is achieved in a number of steps given by a power law of the network size. Thus, copying connectivity from one neural network to another using DNA barcodes is theoretically possible.

**References**

1. LeCun, Y., Y. Bengio, and G. Hinton: Deep learning. Nature 2015, **521**: 436–44.

2. Kebschull, J.M., et al.: High-Throughput Mapping of Single-Neuron Projections by Sequencing of Barcoded RNA. Neuron 2016, **91**: 975–87.

3. Zador, A.M., et al.: Sequencing the connectome. PLoS Biol 2012, **10**: e1001411.

4. Lee, J.H., et al.: Fluorescent in situ sequencing (FISSEQ) of RNA for gene expression profiling in intact cells and tissues. Nat Protoc 2015, **10**: 442–58.

## P309 Triads of synchronized theta cycles boost Cross-Frequency Coupling during novelty exploration

### Víctor J. López-Madrona^1^, Ernesto Pereda^2^, Claudio R. Mirasso^3^, and Santiago Canals^1^

#### ^1^Instituto de Neurociencias, Consejo Superior de Investigaciones Científicas, Universidad Miguel Hernández, Sant Joan d’Alacant 03550, Spain; ^2^Departamento de Ingeniería Industrial, Escuela Superior de Ingeniería y Tecnología, Universidad de La Laguna Avda. Astrofísico Fco. Sanchez, s/n, La Laguna, Tenerife 38205, Spain; ^3^Instituto de Física Interdisciplinar y Sistemas Complejos, CSIC-UIB, Campus Universitat de les Illes Balears E-07122, Palma de Mallorca, Spain

##### Correspondence: Víctor J. López-Madrona (v.lopez@umh.es)

*BMC Neuroscience* 2017, **18** (**Suppl 1**):P309

**Introduction:** A prominent feature of brain activity is the presence of oscillations in neuronal recordings. Interactions between brain rhythms have been demonstrated at different space and time scales, and are believed to play an important role in neuronal communication. One such interactions is the cross-frequency coupling (CFC) between the phase of the theta rhythm and the amplitude of gamma oscillations in the hippocampus. However, neither the cellular mechanisms supporting this interaction, nor its behavioral correlates, are well understood. In the present work, we study the causal interactions between both frequency bands and the features maximizing this interaction in the hippocampus of behaving animals.

**Methods:** Sprague-Dawley rats (n = 5) were used for multi-site, multichannel electrophysiological recordings in freely moving conditions. Local field potentials (LFP) were recorded across the CA1 and dentate gyrus (DG) regions of the dorsal hippocampus. We applied an independent component analysis to dissect the local generators of the LFP signals. CFC between theta phase and gamma amplitude was computed and the directionality of this modulation measured with phase transfer entropy (PhTE). Alterations on the level of coupling were estimated as a function of the synchronization between the theta phases of the generators and in relation to the exploration of new vs. known environments (novelty test) or new vs. known object locations in a familiar environment (novel object location task or NOL).

**Results:** Phase differences between theta oscillations across hippocampal subfields were patent, as expected from previous literature. PhTE analysis indicates a causal link between theta and gamma bands in each component, suggesting that theta phases modulate gamma amplitudes and no otherwise. We show that CFC in each LFP generator is predominantly found when theta phases are synchronized (locked phase-differences) across hippocampal subfields, at least during three consecutive cycles. However, maximal gamma amplitude is found from the first cycle in the triad. The transitions between synchronization states were analyzed through Markov chains and found a significantly higher probability of phase-locking in CA1 and CA3 previous to a global synchronization state. Importantly, quantification of the modulation index during behavior demonstrates maximal theta-gamma coupling when the subject is exposed to a novel environment or when the animal explores a new object location in the NOL task.

**Conclusions:** Our findings suggest that CFC is a communication mechanism in the hippocampus for encoding new information into memory. Efficient coupling requires precise and sustained synchronization across all subfields, suggesting global integration.

## P310 Simulations of synaptic integration in a detailed Purkinje cell model

### Stefano Masoli^1^, Egidio D’Angelo^1,2^

#### ^1^Department of Brain and Behavioural Science, Neurophysiology and Neurocomputation Unit, University of Pavia, Via Forlanini 6, I-27100, Pavia, Italy; ^2^Brain Connectivity Center, Istituto Neurologico IRCCS C. Mondino, Pavia, I-27100, Italy

##### Correspondence: Stefano Masoli (stefano.masoli@unipv.it)

*BMC Neuroscience* 2017, **18** (**Suppl 1**):P310

The Purkinje cell (PC) is one of the most complex neurons of the brain and integrates more than 100000 synaptic inputs coming from granule cell (GrC) ascending axons (aa), parallel fibers (pf), climbing fibers (cf) and molecular layer interneurons (mli). The synapses are distributed, with zone specific limitations, on a large dendritic tree and exploit different neurotransmission mechanisms to modulate the PC discharge in way that remains largely unknown. Here we have explored this wide synaptic computational space using a detailed PC model built in Python - NEURON [1]. The GrC inputs generated the characteristic burst-pause in the simple spike (SS) responses that were accentuated by MLI inhibition [2]. The cf inputs elicited complex spikes (CS) followed by pauses in SS firing that inversely correlated with the number of spikes in cf bursts [3]. Ephaptic coupling between MLIs and PCs depressed SS firing, especially when associated to GABA-A receptor-mediated synaptic inputs to the PC soma. The activation of GABA-B receptors and Kir channels generated a permanent downstate [4]. The PC discharge patterns depended on the excitatory/inhibitory balance, efficacy, dendritic location, Zebrin (Z + vs. Z-) phenotype, and input patterns of the synapses in a way that matched a large set of experimental observations [5]. The model thus anticipates how a large set of electroresponsive patterns could emerge from the complexity of PCs synaptic organization generating the specific outputs to be transmitted to DCN [6].

**References**

1. Masoli S, Solinas S, D’Angelo E. Action potential processing in a detailed Purkinje cell model reveals a critical role for axonal compartmentalization. *Front. Cell. Neurosci.* 2015; **9:**1–22.

2. Steuber V, Mittmann W, Hoebeek FE, Silver RA, De Zeeuw CI, Häusser M, et al. Cerebellar LTD and pattern recognition by Purkinje cells. *Neuron.* 2007; **54:**121–36.

3. Yartsev MM, Givon-Mayo R, Maller M, Donchin O. Pausing purkinje cells in the cerebellum of the awake cat. *Front. Syst. Neurosci.* 2009; **3:**2.

4. Loewenstein Y, Mahon S, Chadderton P, Kitamura K, Sompolinsky H, Yarom Y, et al. Bistability of cerebellar Purkinje cells modulated by sensory stimulation. *Nat. Neurosci.* 2005; 202–11.

5. Zhou H, Voges K, Lin Z, Ju C, Schonewille M. Differential Purkinje cell simple spike activity and pausing behavior related to cerebellar modules. *J. Neurophysiol.* 2015; **113:**2524–36.

6. Dykstra S, Turner RW. Determinants of rebound burst responses in rat cerebellar nuclear neurons to physiological stimuli *Steven. Mol. Microbiol.* 2015; **82:**1496–514.

## P311 A model for tactile stimuli processing in cuneate nucleus

### Udaya B. Rongala^1^, Alberto Mazzoni^1†^, Anton Spanne^2^, Henrik Jorntell^2†^, Calogero M. Oddo^1†^

#### ^1^The BioRobotics Institute, Scuola Superiore Sant’Anna, Pontedera, Pisa 56025, Italy; ^2^Neural Basis of Sensorimotor Control, Department of Experimental Medical Science, Lund University, Lund, Sweden

##### Correspondence: Alberto Mazzoni (alberto.mazzoni@santannapisa.it)

^†^equal last author contribution

*BMC Neuroscience* 2017, **18** (**Suppl 1**):P311

Our understanding of tactile information processing in humans made critical advancements in the recent years [1] These studies fruitfully interacted with those aiming at the development of neuromorphic devices [2]. Here we tackle this issue combining computational neuroscience and neuroengineering. We modelled tactile sensors and neurons from both the periphery and the cuneate nucleus taking advantage of the existing neurophysiology knowledge. We injected them with inputs coming from electronic hardware sensors presented with a variety of artificial and naturalistic textures. This approach offers rewards in making robots efficient [3], and contributing for better understanding of neural mechanisms of sensory processing [4].

First, we generated artificial mechanoreceptor-like output injecting the output of our biomimetic tactile sensor into an Izhikevich regular spiking neuron. We injected the normalized output and its derivative to reproduce the dynamics of Slowly Adaptive and Fast Adaptive neurons respectively [1]. We mimicked the rich information content in primary afferent sensors by presenting 10 naturalistic textures (Glass, BioSkin, Textiles, etc.) to our tactile sensor, in a passive touch protocol. We have achieved accuracy as high as 97% in classifying these 10 textures using a kNN decoding based on Victor-Purpura distances [5]. As a second step, we have simulated a second layer of neurons receiving the output of mechanosensors with conduction delays mimicking the peripheral nerve fibers that transmit primary afferent signals onto the cuneate neurons (CNs). The CNs are the second order neuron structure present in the brain stem [1, 2], that is responsible in segregating the PA information based on different tactile input features [6]. The conduction delays generated a structure of coincident inputs that could encode stimuli orientation [7]. We modeled then the learning of stimuli segregation in CNs, based on recent neurophysiology studies [6, 8]. We developed a model of synaptic learning plasticity able to reproduce the sparseness of CNs encoding of information from primary afferent. We tested this model by presenting a broad spectrum of high & low frequency inputs (textures & shape stimuli, using sliding & indentation protocol respectively) in a pseudo random fashion to induce a realistic rearrangement of synaptic weights, studying the evolution of connectivity with stimulation history. We found that highly specialized CNs tended to pick up diverse features in the input spike patterns and hence in tactile stimuli. Our model provides a candidate mechanism for feature extraction in CNs and might pave the way to neuromorphic algorithms able to learn to segregate tactile inputs.

**Acknowledgements**

This work was supported by institutional funds from Scuola Superiore Sant’Anna, and by the Ministero degli Affari Esteri e della Cooperazione Internazionale, via the Italy-Sweden bilateral research projectSE14GR4.

**References**

1. Johansson RS, Flanagan JR: Coding and use of tactile signals from the fingertips in object manipulation tasks. *Nature Reviews Neuroscience*2009, **10**: 345–359.

2. Bologna LL, Pinoteau J, Passot JB, Garrido JA, Vogel J, Vidal ER, Arleo A:A closed-loop neurobotic system for fine touch sensing. *Journal of neural engineering* 2013, **10(4)**:046019.

3. ServiceRF: Minds of their own. *Science*2014, **346.6206**:182-183.

4. Oddo CM, Raspopovic S, Artoni F, Mazzoni A, Spigler G, Petrini F, Giambattistelli F, Vecchio F, Miraglia F, Zollo L, et al.: Intraneural stimulation elicits discrimination of textural features by artificial fingertip in intact and amputee humans. *Elife* 2016, **5**:e09148.

5. Rongala UB, Mazzoni A, Oddo CM: Neuromorphic artificial touch for categorization of naturalistic textures. *IEEE transactions on neural networks and learning systems* 2015, **PP(99)**: 1–1

6. Jörntell H, Bengtsson F, Geborek P, Spanne A, Terekhov AV, Hayward V: Segregation of tactile input features in neurons of the cuneate nucleus. *Neuron* 2014, **83(6)**:1444–52.

7. Rongala UB, Mazzoni A, Camboni D, Carrozza MC, Oddo CM: Neuromorphic artificial sense of touch: Bridging robotics and neuroscience. *Robotics Research, Springer Proceedings in Advanced Robotics* 2017.

8. Bengtsson F, Brasselet R, Johansson RS, Arleo A, Jörntell H: Integration of sensory quanta in cuneate nucleus neurons in vivo. *PloS one* 2013, **8(2)**:e56630.

## P312 A new method of system visualization of cognitive functioning for fMRI

### Alexander V. Vartanov^1^, Anastasia K. Neklyudova^1^, Stanislav A. Kozlovskiy^1^, Andrey A. Kiselnikov^1^, Julia A. Marakshina^1,2^

#### ^1^Department of Psychology, Lomonosov Moscow State University, Moscow, Russia; ^2^Psychological Institute of Russian Academy of Education, Moscow, Russia

##### Correspondence: Alexander V. Vartanov (a_v_vartanov@mail.ru)

*BMC Neuroscience* 2017, **18** (**Suppl 1**):P312

There are two main ways of neural networks identification in human brain using fMRI: based on searching for dependency of voxel activity on the task performance and based on finding interdependency of voxel activity among each other (i.e., resting state) [1]. The disadvantage of the first method is that it is practically impossible to select an experimental task which only one cognitive process is involved in, and that is why it is difficult to talk about specificity of the obtained network to some particular function. Because of this item, it is necessary to conduct a system of tasks. Brain activity which was obtained using the second way also connects not only with certain cognitive processes, but reflects primarily default state of the brain. Furthermore, another serious problem is artifacts and physiological noise [2]. A new method of system visualization of cognitive functioning for fMRI (Russian Federation #2016149614 patent pending) and corresponding software (Fact-fMRI) for complex factor analysis for several individual datasets is presented (Fig. 1). Compared to the ICA method, the new method allows to estimate the number of functional systems by orthogonal factors which can be additionally rotated. This method allows to formalize identification of brain systems which are involved in executing of different cognitive tasks. A proposed method includes the following stages:

1. Normalization of voxel activity in a given time slice in a specific task;
2. Obtaining a matrix of voxel activity in time slices in each of the tasks;
3. Calculating cross-correlation between different time slices within one task and among the others and the following factorization (Q factor analysis);
4. Dimensionality estimation of this matrix based on eigenvalues;
5. Obtaining the orthogonal system of factors (or factor loadings) as the result of different types of rotation;
6. Computing a corresponding matrix of factor scores.

The dimensionality of the obtained matrix is interpreted as an amount of functional systems involved in all conducted cognitive tasks. Factor loadings are interpreted as characteristic of dynamics of each system as a whole in different tasks. Factor scores reveal the localization of each brain system.
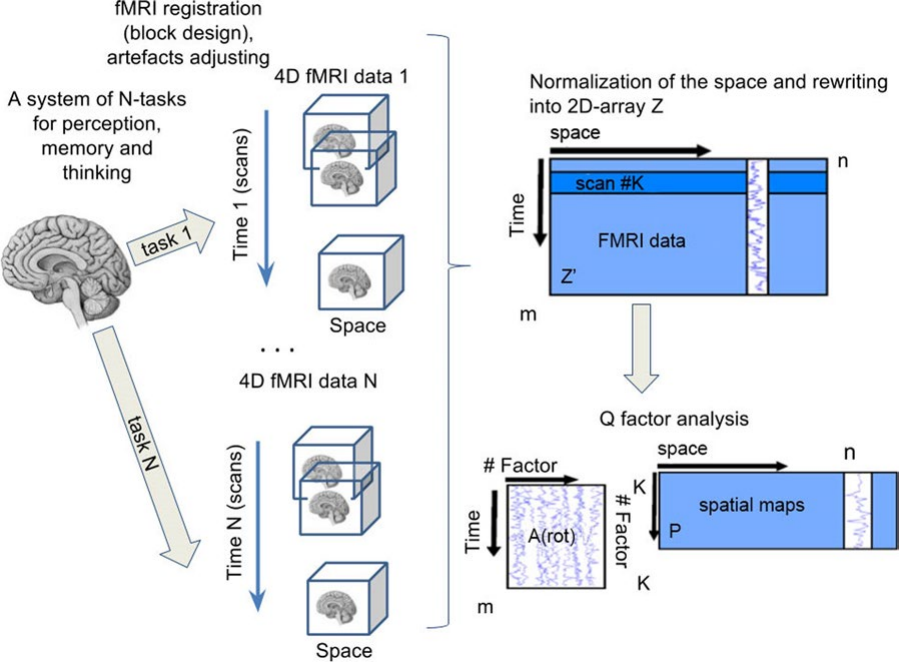


**Figure 1.** A scheme of the Fact-fMRI method. N - a number of cognitive tasks; m - a general number of scans within N tasks; n - a number of analyzing voxels in the brain; Z - a normalized matrix of initial data; K - a number of obtained factors, which interpreted as an amount of functional systems included in all cognitive tasks; A (rot) - a matrix of factor loadings after rotation, describing dynamics of brain activity for each of functional systems; P - factor scores, revealing the localization of each brain system

Thus, the proposed method allows to combine data which show conducting several cognitive tasks by a person in a single model. Furthermore, it allows to identify and characterize corresponding functional brain systems.

**Acknowledgements**

The research was supported by the Russian Science Foundation, project № 16-18-00066.

**References**

1. Biswal BB: Resting state fMRI: a personal history. *Neuroimage*, 2012, **62(2):** 938–944.

2. Birn RM, Murphy K, Bandettini PA: The effect of respiration variations on independent component analysis results of resting state functional connectivity. *Human brain mapping*, 2008, **29(7):** 740–750.

## P313 Synaptic distribution predicts unitary LFP fields in the hippocampus and in the neocortex

### Maria Teleńczuk^1,2^, Bartosz Teleńczuk^1,2^, Alain Destexhe^1,2^

#### ^1^European Institute for Theoretical Neuroscience, Paris, France; ^2^Unité de Neurosciences, Information et Complexité, Gif-sur-Yvette, France

##### Correspondence: Maria Teleńczuk (maria@telenczuk.pl)

*BMC Neuroscience* 2017, **18** (**Suppl 1**):P313

The local field potential (LFP) is a widely-used signal to monitor the activity of neural populations. It is usually considered to be generated by the synaptic currents triggered by pre-synaptic action potentials. Nevertheless, the magnitude and spatial distribution of LFP depends greatly on the anatomy of recorded region of the brain, including the neuron morphology, arrangement of different neuron types and the distribution of excitatory and inhibitory synapses.

In hippocampus, the pyramidal cells are aligned with somas placed in the stratum pyramidale (s.p), basal dendrites in stratum oriens (s.o) and apical dendrites stretching through stratum lucidum (s.l), stratum radiatum (s.r) and stratum lacunosum moleculare (s.lm). Distribution of synapses terminating on the pyramidal cells is also framed. Synapses of the basket cells terminate mostly on or near to the soma of pyramidal cells forming basket-like-looking dense axonal structures [1, 2]. Pyramidal cells, on the other hand, stretch their axons much further from the cell body and terminate their synapses in the s.r and s.o. Extracellular recordings from the pyramidal cell layer, besides spiking activity, show very distinct inhibitory fields generated by single interneurons [3, 4].

In neocortex, on the other hand, neurons are positioned in parallel, they are shifted in the vertical axis. Although, inhibitory synapses are still placed on nearby somas, they generate effectively closed-field symmetry, which does not produce large far-field potentials. Recently, we have shown, however, that the electric field following a single interneuron spike dominates the on-going LFP. Excitatory neurons contribute to the LFP with longer latencies suggesting that their contribution is di-synaptic, mediated by an intermediary interneuron [5].

In the present study, we are investigating this discrepancy using computational modelling. We place pyramidal neurons according to their distribution in the hippocampus and neocortex and activate inhibitory or excitatory synapses on them. We follow the distributions of neurons and synapses found in the literature and realistic morphology downloaded from online databases (neuromorpho.org). The simulation of the model is performed in NEURON simulator through its Python interface and the extracellular field is calculated using the NeuroEAP library [6]. We reproduce the findings of Bazelot et al. [4] in the hippocampus and Telenczuk et al. [5] in the neocortex and show that the difference in the field magnitude originate from the differences in the distribution of synaptic target of inhibitory and excitatory neurons. Importantly, we find that the magnitude of the LFP generated by the synapses and the relative contribution of excitatory vs. inhibitory pre-synaptic neurons depend on the cortical layer and the source of feedforward activation.

**Acknowledgements**

We acknowledge support from the European Commission (The Human Brain Project, grant number: H2020-720270) and Centre National de la Recherche Scientifique (CNRS, France).

**References**

1. Miles R, Tóth K, Gulyás AI, Hájos N, Freund TF. Differences between somatic and dendritic inhibition in the hippocampus. *Neuron* 1996; **16:**815–23.

2. Le Duigou C, Simonnet J, Telenczuk M, Fricker D, Miles RM. Recurrent synapses and circuits in the CA3 region of the hippocampus: an associative network. *Front. Cell. Neurosci.* 2014; **7:**262.

3. Glickfeld LL, Roberts JD, Somogyi P, Scanziani M. Interneurons hyperpolarize pyramidal cells along their entire somatodendritic axis. *Nat. Neurosci.* 2009; **12:**21–3.

4. Bazelot M, Dinocourt C, Cohen I, Miles R. Unitary inhibitory field potentials in the CA3 region of rat hippocampus. *J. Physiol.* 2010; **588:**2077–90.

5. Teleńczuk B, Dehghani N, Quyen MLV, Cash SS, Halgren E, Hatsopoulos NG, et al. Local field potentials primarily reflect inhibitory neuron activity in human and monkey cortex. *Sci. Rep.* 2017; **7:**40211.

6. Telenczuk B, Telenczuk M. NeuronEAP library. Zenodo. 2016.

## P314 Differential tuning of the low- and high-frequency components of the neurophonic spectrum reveals the spike contribution of barn owl’s nucleus laminaris neurons

### Paula T. Kuokkanen^1,2,3^, Anna Kraemer^3^, Thomas McColgan^1^, Catherine E. Carr^3^, Richard Kempter^1,2^

#### ^1^Inst. For Theoretical Biology, Humboldt-Universitaet Zu Berlin, 10115 Berlin, Germany; ^2^Bernstein Center for Computational Neuroscience Berlin, 10115 Berlin, Germany; ^3^Dept. of Biol., Univ. of Maryland, College Park, MD 20742, USA

##### Correspondence: Paula T. Kuokkanen (p.kuokkanen@biologie.hu-berlin.de)

*BMC Neuroscience* 2017, **18** (**Suppl 1**):P314

In-vivo neural activity gives rise to transmembrane currents that can be recorded as an extracellular field potential. These potentials are often challenging to interpret due to thousands of contributing sources. We aim at revealing the neural sources of the “neurophonic”. The neurophonic is a frequency-following extracellular potential that can be recorded in the network formed by the nucleus magnocellularis (NM) and the nucleus laminaris (NL) in the brainstem of the barn owl. NL anatomy is well understood, and putative generators of the neurophonic are the activity of afferent axons from NM, the synaptic activation onto NL neurons, and spikes of NL neurons.

We recorded the neurophonic in response to binaural high-frequency tones (3-7 kHz) close to the recording site’s best frequency, and we varied the interaural time difference (ITD). The mean activity of the monaural inputs to NL does not change with ITD. However, their relative phase does, causing cancellation or summation of input signals. The activity of the binaurally sensitive output of NL, i.e., firing rate of NL neurons, strongly depends on ITD. Our recordings contained both of these signals, and we analysed the broadband power spectrum of the response (0-18 kHz).

The low-frequency component (LFc, 200-700 Hz) of the neurophonic spectrum depended on ITD. The spectrum of extracellularly recorded NL neurons’ action potentials closely resembled this component. Thus, the LFc reflects the contribution of action potentials initiated in NL neurons. The spectral component at the stimulus frequency (SFc) was much stronger than the LFc. The SFc also depended on ITD, reflecting the activity of the inputs and their relative phase change with ITD. The power spectrum at other frequencies did not depend on ITD. We used the LFc as a proxy for NL neurons’ local population activity, and the SFc as a proxy for NM axons’ local population activity. We compared the ITD and frequency tunings of these proxies at each recording site. The best ITDs of the LFc and the SFc were independent. Also, the tuning to stimulus frequency was different: LFcs showed typically a 400 Hz lower best frequency than SFcs. Both findings indicate that the LFc might originate from NL neurons’ axons in the vicinity of the electrode. Related NL neurons can be located tens to hundreds of micrometers away. The findings are consistent with the known anatomy of NL. Our analysis thus reveals the small contribution of NL neurons to the neurophonic, improving our understanding of the extracellular field potential in the auditory brainstem.

###### Publisher’s Note

Springer Nature remains neutral with regard to jurisdictional claims in published maps and institutional affiliations.

